# Implication of asymptomatic and clinical *Plasmodium falciparum* infections on biomarkers of iron status among school-aged children in Malawi

**DOI:** 10.1186/s12936-022-04297-1

**Published:** 2022-10-01

**Authors:** Peter A. M. Ntenda, Angeziwa C. Chirambo, Owen Nkoka, Walaa M. El-Meidany, Jessy Goupeyou-Youmsi

**Affiliations:** 1 Malaria Alert Centre, Kamuzu University of Health Sciences, Private Bag 360, Chichiri, Blantyre 3, Malawi; 2 Malawi-Liverpool-Wellcome Trust Clinical Research Programme, P.O. Box 30096, Mahatma Ghandhi Road, Chichiri, Blantyre, Malawi; 3 Institute of Health and Wellbeing, University of Glasgow, Glasgow, G12 8QQ UK; 4 Department of Nutrition, High Institute of Public Health, Alexandria University, Hiph 65 El-Horreya Avenue, El-Ibrahimia, Alexandria, Egypt

**Keywords:** Parasitemia, Clinical malaria, Inflammation, Iron status, Anaemia, Malawi

## Abstract

**Background:**

Iron status is considered as a continuum from an iron deficiency with anaemia, without anaemia, varying amounts of stored iron to iron overload. The burden of *Plasmodium falciparum* infections is typically high among school-aged children (SAC). Nonetheless, SAC are often less likely to be covered by malaria interventions, making them a group with an untreated reservoir of parasite transmission. This study aimed to assess the effects of asymptomatic and clinical malaria infections on biochemical markers of iron status among SAC in Malawi.

**Methods:**

Data from the 2015–2016 Malawi Micronutrient Survey (MNS) was used and multivariable logistic regression models using a generalized estimating equation to account for the complex cluster survey design were constructed. Blood samples of 684 children aged 5 to 14 years old were evaluated for clinical and asymptomatic malaria infections. Furthermore, blood samples were used to estimate haemoglobin (Hb), serum ferritin (SF) and, soluble transferrin receptors (sTfR) concentrations.

**Results:**

Of the 684 SAC analysed, approximately 42% had asymptomatic malaria, while 41.0% had clinical malaria. Anaemia (low Hb levels), iron deficiency (low SF concentration), and functional iron deficiency (high sTfR levels) were found in 20%, 5%, and 30% of the children, respectively. School-aged children with asymptomatic malaria had increased odds of being anaemic (adjusted odds ratio [aOR]: 3.71, 95% confidence interval [CI]: 2.29–5.99) and increased levels of sTfR (aOR: 3.00, 95% CI 2.01–4.47). Similarly, SAC with clinical malaria had increased odds of being anaemic (aOR: 3.54, 95% CI 2.19–5.72) and increased levels of sTfR (aOR: 3.02, 95% CI 2.02–4.52).

**Conclusions:**

Both asymptomatic and clinical malaria were independent risk factors for anaemia and functional iron deficiency (FID). The notion that asymptomatic and clinical malaria were associated with both anaemia and FID underscores the need for public health programmers to consider adding mass screening and treatment for malaria to existing school-based health programmes.

**Supplementary Information:**

The online version contains supplementary material available at 10.1186/s12936-022-04297-1.

## Background

Iron status is considered as a variety of conditions ranging from iron deficiency (ID) with anaemia [i.e., reduced haemoglobin (Hb) in red blood cells—RBCs], to ID without anaemia (i.e., depleted iron stores), to normal iron status with varying amounts of stored iron, to iron overload [1–4]. Iron deficiency is a condition in which there are no mobilizable iron stores and in which signs of a compromised supply of iron to the tissues, including the erythron, are noted [4, 5]. Globally, ID, specifically iron deficiency anaemia (IDA), remains one of the most public health nutritional problems [6, 7] and constitutes a wide range of poor health outcomes [6, 8]. Worldwide, ID is considered the most common cause of anaemia and is responsible for 50% of all anaemias [9, 10], although other factors such as malnutrition [11, 12], inflammation [12, 13], and haemoglobinopathies [12, 14] that affect Hb synthesis, can all be the cause of anaemia. Iron deficiency may lead to impaired cognitive development in children from infancy through adolescence [15, 16]. Further, ID may damage immune mechanisms, and increase rates of morbidity [17, 18]. Additionally, when IDA occurs in pregnancy, it is associated with multiple adverse outcomes for both mother and infant [19, 20]. These outcomes include an increased risk of haemorrhage, sepsis, maternal mortality, perinatal mortality, low birth weight, and premature delivery [19, 20].

Indicators of iron status encompass a continuum of parameters and can be confounded by factors varying from inflammation to analytical challenges [3]. Generally, inflammation may prevent body from using stored iron to make enough healthy RBCs, also may impair iron status by decreasing intake of food and reducing intestinal absorption [21, 22]**.** On the other hand, analytical challenges may affect iron status interpretation due to the lack of assay standardization, common reference ranges, and common cutoffs [23]. The World Health Organization (WHO) and the Centers for Disease Control and Prevention (CDC) technical consultation on the assessment of iron status at the population level, selected five measures namely haemoglobin (Hb), zinc protoporphyrin (ZPP), mean cell volume (MCV), soluble transferrin receptor (sTfR) and serum ferritin (SF), as indicators of iron status [24, 25]. Some markers of iron status such as haemoglobin and serum ferritin are affected by inflammation and infection. Thus, to deal with the inflammation-induced biases such as those from infectious diseases, chronic diseases, and tissue injury, the WHO and CDC recommended that the measurement of iron status should be corrected for acute-phase protein (APP) concentrations, in particular, C-reactive protein (CRP) and/or alpha-1-acid glycoprotein (AGP) [26]. They advised further that Hb should be used as the measure of anaemia [3], ZPP was suggested to reflect the systematic deficiency of iron supply to erythrocytes in bone marrow [27]. At the same time, MCV should be assessed to indicate whether RBCs are either macrocytic or microcytic [28]. Further, sTfR levels were suggested to reflect the magnitude of erythropoiesis and the demand for iron [28], and SF concentration as an indicator of body iron stores [29].

Malaria is a life-threatening disease caused by five *Plasmodium* species; namely, *Plasmodium falciparum*, *Plasmodium ovale*, *Plasmodium malariae*, *Plasmodium vivax* and *Plasmodium knowlesi* [30]. Globally, by the end of 2020, an estimated 241 million cases of malaria were recorded, of which 627,000 cases resulted in deaths [31], while one-thirds of these deaths were a result of disruptions to health services during the COVID-19 pandemic [32]. Despite the extensive coverage of malaria control strategies in the last decade, malaria remains a leading cause of morbidity and mortality (especially among children under 5 years of age and pregnant women) in sub-Saharan Africa [33, 34]. Universal malaria interventions, such as bed nets (i.e. insecticide-treated nets and long-lasting insecticidal nets) access and usage, and access to prompt diagnosis and treatment (use of artemisinin-based combination therapy), have targeted these two groups at the highest risk for malaria disease [35, 36]. However, previous researchers have demonstrated that school-aged children (SAC) bear an under-appreciated burden of malaria, yet this age group is often not prioritized [37, 38]. Hence, SAC constitutes a group with an untreated reservoir of parasite transmission [37, 39]. Several health consequences for malaria chronically infected children have been recognized in previous studies [40, 41]. Specifically, *P. falciparum* infections in SAC are associated with compromised health, diminished cognitive function, and lower educational achievement [39, 42, 43]. Furthermore, it is known that *P. falciparum* infections involve an increased removal of parasitized and unparasitized RBCs through several mechanisms that may result in anaemia in under-five children [44, 45]. There is limited information on whether malaria infection has effects on the markers of iron status among SAC since most studies among this age group have focused on prevalence and risk factors. Most SAC with malaria parasitaemia do not exhibit any symptoms owing to some immunity they have acquired [37, 39]. However, asymptomatic and clinical infections may contribute to poor iron status including anaemia, and other poor health outcomes. The findings of this study may help public health programme designers to formulate new policies and approaches for the prevention of malaria in SAC with the view to improve their nutrition status. Therefore, the current study examined the implication of asymptomatic and clinical *P. falciparum* infections on biomarkers of iron status among this under-appreciated age group in Malawi.

## Methods

### Design, data source, and sampling methods

**Fig. 1:**
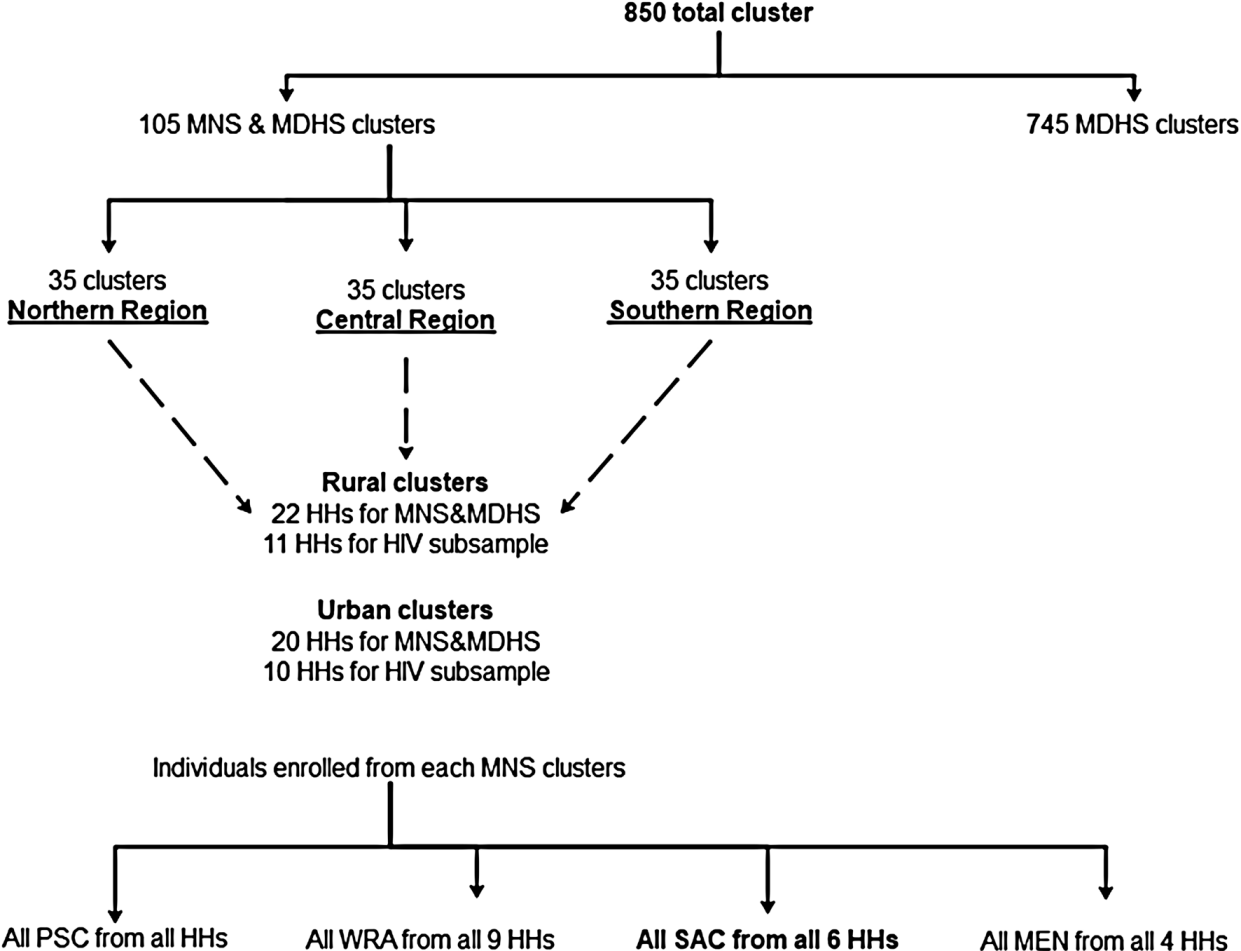
Describes the survey sampling design

This was a cross-sectional study that used data from the 2015–2016 Malawi Micronutrient Survey (MNS). The MNS was conducted jointly with the 2015–2016 Malawi Demographic and Health Survey (MDHS) between December 2015 and February 2016. The comprehensive methods used in this study can be obtained from elsewhere [46, 47]. In brief, the 2015–2016 MDHS employed a two-stage cluster sampling design to produce a nationally representative sample. The first stage selected 850 clusters proportional to population. The second stage selected 27 516 households from the clusters with an equal probability of systematic selection. The 2015–2016 MNS was selected as a subsample from the MDHS to produce estimates of the key indicators of population health for the country as a whole. A subsample of 105 clusters for MNS (35 clusters in each of the three regions) was randomly selected from the 850 MDHS clusters (Fig. 1). However, 20 per urban cluster and 22 per rural cluster were finally included in the MNS after 10 per urban cluster and 11 per rural cluster were excluded since households from these clusters were selected for the MDHS human immunodeficiency virus (HIV) subsample (Fig. 1). In each selected household, all eligible participants were invited to take part in the survey as demonstrated in the Fig. 1.

### Survey implementation

The MDHS teams collected survey data electronically using tablets, while the MNS teams used paper-based questionnaires pre-translated into two common Malawian languages, i.e. Chichewa and Tumbuka. Further, the MDHS teams completed data collection in each cluster before the arrival of the MNS team. The MDHS teams pre-selected the MNS participants through an algorithm pre-programmed into tablets. After finalizing data collection in each selected household, the MDHS supervisor completed the cover sheet of the MNS questionnaire booklet with the names and ages of all eligible individuals selected for the MNS. The MDHS enumerators placed a household label with a unique barcode on the questionnaire and entered the barcode number into the tablet for each eligible household. Eventually, the barcode number allowed the data collected separately by the MDHS and the MNS teams to be linked (Additional files **s**1, **s**2).

### Sample size determination

The sample size calculation provided in the current study was adapted from the original survey, and that a subset of these was used for the current study. Basically, the estimates for MNS were calculated based on a predicted change in the prevalence of vitamin A deficiency in preschool-aged children (PSC), i.e. from 22% in 2009 to 16% in 2015–16. Considering a confidence level of 95%, power of 80%, design effect of 2.0, and 90% household and individual response rates, data had to be collected on a minimum of 1452 PSC. Calculations assumed a 90% household-response rate, 90% individual-response rate, and an average household size of 4.3 persons. The 2015–16 MNS was conducted in 2262 residential households, including 480 households in urban areas and 1782 households in rural areas. Thus, the sample size calculated was expected to result in data collected from about 750 eligible women of reproductive age, 252 eligible men, 700 eligible SAC.

### Inclusion and exclusion criteria

The present study focused on SAC (children aged 5 to 14 years old). In order to achieve quality data, the MNS excluded those deemed too ill to participate and those with a physical disability that would prevent them from having accurate height and/or weight measurements. All SAC with missing information in any of the variables (*n* = 6) were also excluded from this study. A final sample of 684 was analysed.

### Data collection

#### Sociodemographic

Data were collected from women aged 15–49 years who had children aged between 5 and 14 years prior to the survey. Information on sociodemographic, household and economic factors, and clinical characteristics were collected using semi-structured questionnaires.

#### Field and laboratory procedures

**Fig. 2:**
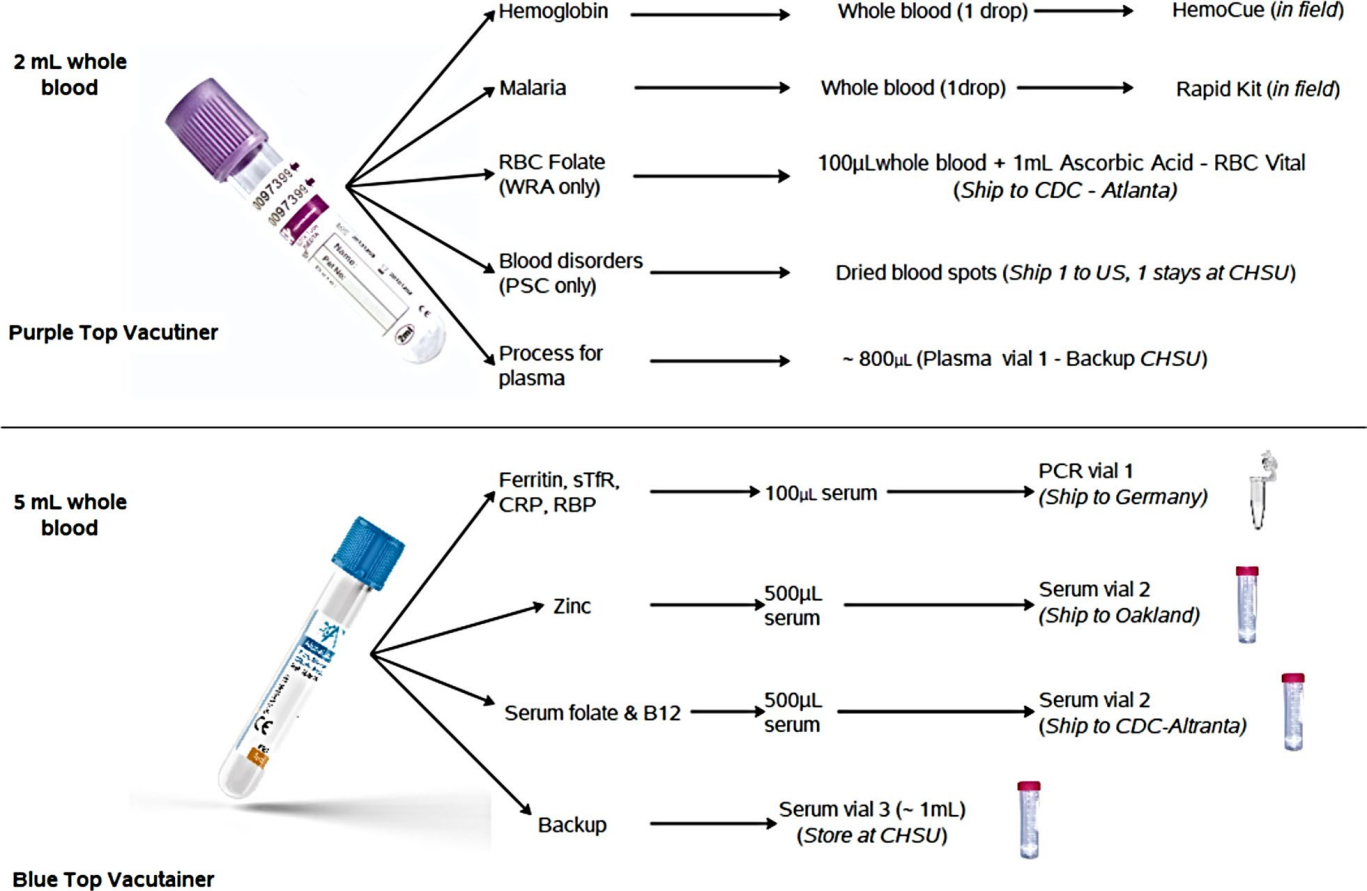
Describes the specimen volumes and testing approaches

An axillary temperature of every SAC was documented where a temperature equal to or greater than 37.5 °C was defined as fever. Approximately 7 mL of blood samples were collected from children for biochemical and hematological assays (Fig. 2). About 5 mL of the blood sample was transferred into trace free elements test tube and 2 mL into the ethylenediaminetetraacetic acid (EDTA) test tube [46].

#### Haematological tests

Approximately 10 μL of the whole blood sample from the EDTA vacutainer (Fig. 2) was used to examine Hb concentration using HemoCue® Hb 301 system (Ängelholm, Sweden) and recorded in g/dL [48, 49]. The HemoCue® 301 blood Hb system comes with a set of an analyzer and microcuvettes. The microcuvette acts both as a pipette as well as a measuring cuvette [50]. A blood sample of about 10 µL is drawn into the cavity by capillary action. The measurement takes place in the analyzer, which measures the absorbance of whole blood at an Hb/HbO_2_ isobestic point [50, 51]. The analyzer measures at two wavelengths (506 and 880 nm) in order to compensate for turbidity [51]. The HemoCue Hb 301 System is calibrated against the haemoglobin cyanide (HiCN) method, the international reference method for the determination of the haemoglobin concentration in blood [51].

#### Parasitology tests

Another 10 μL of the blood sample from the EDTA vacutainer was used to test for malaria (Fig. 2). The Standard Diagnostic BIOLINE Malaria Ag *P.f*/Pan histamine-rich protein (HRP-II) rapid diagnostic test (RDT) was used [52].

#### Biochemical tests

Regarding biochemical tests, 100 μL of serum from the trace free elements test tube (Fig. 2) was transferred into polymerize chain reaction vials and were shipped to Germany for the biochemical examination of SF and sTfR along with other parameters such as alpha-1-acid glycoprotein (AGP) and C-reactive protein (CRP) [46]. A combined measurement of the biochemical tests was performed using an inexpensive, sensitive, and simple sandwich enzyme-linked immunosorbent assay technique in the VitMin laboratories in Germany [53].

### Measures

#### Dependent variable

The dependent variable of the current study was iron status measured using three domains namely; anaemia, ID, and FID. Using the WHO recommendations, anaemia was defined as Hb concentration < 11.5 g/dL for children 5–11 years, and < 12.0 g/dL for children 12–14 years. Hb concentration was adjusted for altitude [46]. Serum ferritin adjusted for inflammation using internal regression approach [54] was used to define the ID (depleted iron stores). Children were classified as having depleted iron stores if their SF concentration was < 15 μg/L [55, 56]. Finally, sTfR levels of ≥ 8.3 mg/L were considered elevated and represented a FID in this populations [46, 55–57]. The FID is defined as a state in which there is inadequate iron incorporation into erythroid precursors in the face of apparently adequate body iron stores, as defined by the presence of stainable iron in the bone marrow together with a SF value within normal limits [58].

#### Main independent variables

Asymptomatic and clinical malaria were the two main independent variables considered in this study. Clinical malaria is defined as *P. falciparum* parasitaemia of any density and axillary temperature ≥ 37.5 °C [59]. On the other hand, asymptomatic malaria is defined as *P. falciparum* parasitaemia of any density with an axillary temperature < 37.5 °C or without any apparent clinical manifestations at the time of collecting the blood [59]. Usually, clinical malaria is defined when an individual has malaria-related symptoms such as fever (an axillary temperature ≥ 37.5 °C), chills, severe malaise, headache or vomiting at the time of examination or 48 h prior to the examination in the presence of a *P. falciparum* positive blood smear [60]. However, in the current study, *P. falciparum* infection for both clinical malaria and asymptomatic malaria was confirmed using the RDT [44]. Table 1 shows the definition of the dependent and main independent variables as well as other biochemical markers.

**Table 1:** Definition of clinical and biochemical variables

Variable	Definition
Clinical malaria	*Plasmodium falciparum* parasitemia of any density and axillary temperature ≥ 37.5 °C. Positive *P. falciparum* parasitemia was confirmed using the rapid diagnostic test (RDT)
Asymptomatic malaria	*P. falciparum* parasitemia of any density with an axillary temperature < 37.5 °C. Positive *P. falciparum* parasitemia was confirmed using the RDT
Anemia	*Hb* levels < 11.5 g/dL for SAC 5–11 years, and < 12.0 g/dL for SAC 12–14 years adjusted for altitude
Iron deficiency	*Serum ferritin* level < 15 µg/L adjusted for inflammation using internal regression approach
Functional iron and deficiency	*Serum soluble transferrin receptor* levels ≥ 8.3 mg/L
Acute inflammation	*Serum C-reactive protein* (CRP) > 5 mg/dL
Chronic inflammation	*Serum alpha-1-acid glycoprotein* (AGP) 1 g/L
Any inflammation	Elevated AGP or CRP (AGP > 1 or CRP > 5)

#### Covariates

The covariates included in this study were, sex of the child, age of the child, fever, and diarrhoea in the last 2 weeks, acute and chronic inflammation, any inflammation, household hunger, type of place of residence, and region of residence. Sex (male/female), the age of the children was categorized as 5–10 years and 11–14 years. Fever (Yes/No), the respondents were asked if their child had a fever in the last 2 weeks. Similarly, diarrhoea was also reported for episodes that occurred in the last 2 weeks. Basically, diarrhoea is defined as the passage of three or more loose or liquid stools per day (or more frequent passage than is normal for the individual) [61]. Acute and chronic inflammation were measured using AGP and CRP levels, respectively [21]. Chronic inflammation was defined as alpha-1-acid glycoprotein (AGP) levels greater than 1 g/L, while acute inflammation was defined as C-reactive protein (CRP) levels greater than 5 μg/L [21, 46]. Additionally, any inflammation was defined as elevated AGP or CRP [21, 46]. The household hunger scale was categorized as little to no hunger and moderate to severe hunger using the recommendations from the Food and Nutrition Technical Assistance III Project [62]. The type of place of residence (rural and urban), and region of residence (northern, central, and southern) assessed the area of residence and region, respectively.

### Statistical analyses

All statistical analyses were conducted using SAS software version 9.4 (SAS Institute, Cary, NC, USA). The survey-specific SAS procedures for weighting, clustering, and stratification were used to account for the complex survey design. Baseline characteristics were reported as weighted frequency and percentages stratified by sex of the child. The differences in sociodemographic, clinical outcomes, biochemical markers, morbidity outcomes, and community factors according to the sex of the child were compared using Rao-Scott Chi-Square test. The effects of asymptomatic and clinical malaria on the markers of iron status were performed using univariate and multivariable logistic regression analyses. Analyses were adjusted for clustering effects of individuals from the same households by use of generalized estimating equations. The results of multivariable analyses were reported as adjusted odds ratio (aOR) with their p-values and 95% confidence intervals (CI). P-values < 0.05 were considered to indicate statistical significance.

### Ethical statement

A permission was obtained from the Monitoring and Evaluation to Assess and Use Results Demographic and Health Surveys (MEASURE) and/or the Demographic and Health Survey (DHS) program for the use MNS 2015–16 datasets before the primary purpose. The MNS survey protocol was approved by the Institutional Review Board of ICF Macro and the Malawi National Health Sciences Research Committee (NHSRC). The survey was implemented by the National Statistics Office, the Community Health Sciences Unit of the Ministry of Health, Department of Nutrition, HIV, and AIDS, and jointly with the 2015–16 MDHS. Informed consent for MNS took place at several levels. Firstly, community leaders from each cluster were informed regarding the MDHS and MNS, and communal consent was sought prior to the arrival of the MDHS teams. Secondly, the MDHS enumerators asked each MNS-eligible household for permission to participate in the MNS where consent for each household was documented on the MNS paper-based questionnaires. Finally, the paper-based questionnaires were then handed over to the MNS team. The nurse asked for informed consent from each individual for anthropometry measurements and biological samples.

## Results

### Baseline characteristics of the study sample

**Fig. 3:**
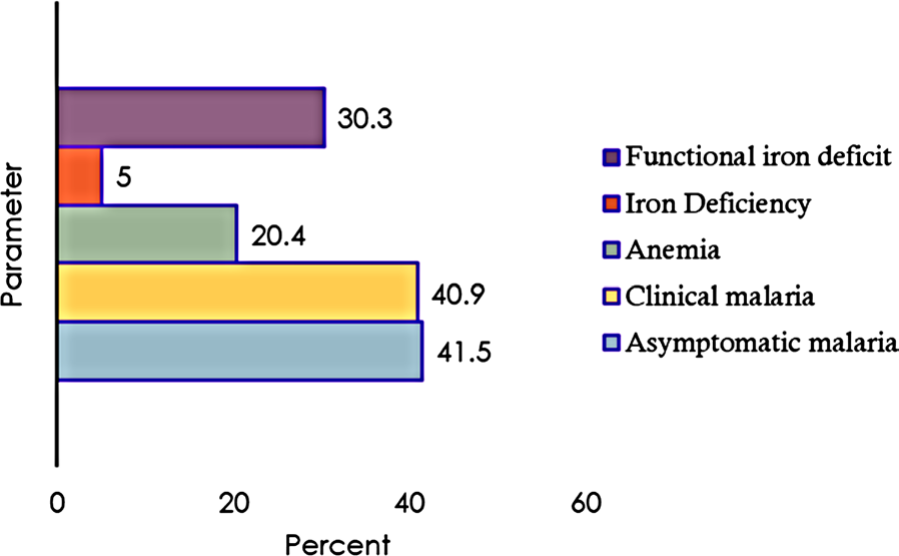
Descriptive results of the outcome and main explanatory variables

Of the 684 SAC analysed, about 42% had asymptomatic malaria while 41.0% had clinical malaria. Furthermore, anaemia, ID, and FID were found in 20%, 5%, and 30% of children, respectively. Figure 3 displays the descriptive results of the outcome and main explanatory variables. Approximately, 52% of the children were female and nearly two-thirds (62%) of children were in the 5–10 years age group. Nearly 25% and 6% of children had a history of fever and diarrhea in the last 2 weeks, respectively. About 17% of children had acute inflammation, nearly 32% of children had chronic inflammation, and 34% of children had elevated levels of both AGP and CRP. Approximately 60% of children resided in households with moderate to severe hunger and more than three-quarters (95%) of the children were rural dwellers. Table 2 presents the participants’ characteristics by sex of the child. Apart from malaria infection outcomes, sociodemographic, clinical, and biochemical characteristics considered in this study were comparable between these two groups.

**Table 2:** Sociodemographic, clinical, and biochemical characteristics of the study sample

Characteristics	Total 684 (100)	Female 335 (51.9)	Male 329 (48.1)	*P*-value*
	*n* (%)	*n* (%)	*n* (%)	
Child age (years)				0.2567
5–10	437 (62.1)	230 (32.8)	125 (17.8)	
11–14	247 (37.7)	207 (29.3)	122 (20.1)	
Fever in last 2 weeks				0.4187
Yes	163 (24.8)	83 (13.4)	272 (37.2)	
No	521 (75.2)	80 (11.4)	249 (38.0)	
Diarrhea in last 2 weeks				0.4187
Yes	37 (6.0)	15 (2.7)	338 (47.9)	
No	643 (94.0)	22 (3.3)	305 (46.1)	
Asymptomatic malaria				0.0098
Yes	257 (41.5)	144 (24.1)	211 (26.5)	
No	427 (58.5)	113 (174)	216 (32.0)	
Clinical malaria				
Yes	253 (40.9)	141 (23.8)	213 (26.8)	0.0104
No	4331 (59.1)	111 (17.1)	218 (32.3)	
C-reactive protein (CRP)				0.2943
≤ 5 mg/dL	573 (83.1)	291 (41.1)	64 (9.5)	
> 5 mg/dL	111 (16.9)	282 (42.0)	47 (7.4)	
Alpha-1-acid glycoprotein (AGP)				0.1713
≤ 1 g/L	459 (68.2)	230 (32.9)	125 (17.7)	
> 1 g/L	225 (31.8)	229 (35.2)	100 (14.2)	
Elevated CRP or AGP				0.0952
No	447 (65.9)	223 (31.4)	132 (19.2)	
Yes	237 (34.1)	224 (34.5)	105 (14.9)	
Household hunger scale				0.3788
Little to none	341 (40.2)	175 (19.3)	180 (31.3)	
Moderate to severe	355 (59.8)	166 (20.9)	163 (28.5)	
Place of residence				
Urban	89 (5.2)	47 (2.0)	308 (48.6)	0.2418
Rural	595 (94.8)	42 (3.2)	287 (46.2)	
Geographical region				
North	230 (13.8)	117 (6.3)	113 (7.5)	0.6552
Central	250 (41.8)	113 (21.6)	117 (20.2)	
South	204 (44.4)	105 (22.7)	99 (21.7)	
Anemia				0.3050
< 12 g/dL	129 (20.4)	270 (40.3)	59 (9.1)	
≥ 12 g/dL	555 (79.6)	285 (39.3)	70 (11.3)	
Serum ferritin				0.5665
< 15 µg/L	30 (5.0)	311 (46.6)	18 (2.8)	
≥ 15 µg/L	654 (95.0)	343 (48.4)	12 (2.2)	
Soluble transferrin receptors (sTfR)				0.0797
< 8.3 mg/L	490 (69.7)	249 (33.3)	106 (17.3)	
≥ 8.3 mg/L	194 (30.3)	241 (36.5)	88 (13.0)	

*P-values from Rao-Scott Chi-Square test

### Univariate analyses of asymptomatic and clinical malaria on biochemical markers of iron

Tables 3 and 4 list the results of univariate analyses of asymptomatic and clinical malaria on biochemical markers of iron status. Compared to SAC without asymptomatic malaria, SAC with asymptomatic malaria had increased odds of being anaemic (adjusted odds ratio [aOR]: 4.92, 95% confidence interval [CI] 3.18–7.61), *P* < 0.0001 and increased odds of developing functional iron deficit—high of sTfR levels (aOR: 3.46, 95% CI 2.39–5.01), *P* < 0.0001. Similarly, compared to children without clinical malaria, SAC with clinical malaria had increased odds of being anaemic (aOR: 4.70, 95% CI 3.04–7.27), *P* < 0.0001, and increased odds of developing functional iron deficit (aOR: 3.50, 95% confidence interval [CI] 2.41–5.08), *P* < 0.0001.

**Table 3:** Univariate analysis of asymptomatic malaria and its effect on anemia, iron deficiency, and soluble transferrin receptors among school aged children

Variables	Low Hb levels			Low ferritin concentration			High sTfR levels		
	OR	95% CI	*P*-value	OR	95% CI	*P*-value	OR	95% CI	*P*-value
Asymptomatic malaria									
Yes	**4.92**	**(3.18–7.61)**	**< 0.0001**	1.21	(0.56–2.64)	0.6245	**3.46**	**(2.39–5.01)**	**< 0.0001**
No	1.00			1.00			1.00		
Sex									
Female	1.16	(0.77–1.75)	0.4782	1.65	(0.78–3.50)	0.1867	1.18	(0.83–1.69)	0.3628
Male	1.00			1.00			1.00		
Age									
5–10	**2.14**	**(1.35–3.41)**	**0.0013**	1.19	(0.56–2.51)	0.6507	1.00	(0.69–1.45)	0.9815
11–14	1.00			1.00			1.00		
Fever in last 2 weeks									
Yes	**1.65**	**(1.04–2.65)**	**0.0342**	1.26	(0.51–3.15)	0.6155	**1.90**	**(1.26–2.87)**	**0.0023**
No	1.00			1.00			1.00		
Diarrhea in last 2 weeks									
Yes	1.83	(0.80–4.20)	0.1535	1.70	(0.22–12.9)	0.6070	1.76	(0.84–3.69)	0.1359
No	1.00			1.00			1.00		
Elevated CRP or AGP									
Yes	**4.00**	**(2.58–6.19)**	**< 0.0001**	1.48	(0.65–3.39)	0.3506	**1.93**	**(1.33–2.80)**	**0.0005**
No	1.00			1.00			1.00		
Household hunger scale									
Little to none	0.78	(0.50–1.21)	0.2652	0.86	(0.41–1.80)	0.6970	0.84	(0.58–1.23)	0.3798
Moderate to severe	1.00			1.00			1.00		
Place of residence									
Urban	**0.31**	**(0.12–0.78)**	**0.0125**	0.97	(0.33–2.86)	0.9573	**0.49**	**(0.25–0.99)**	**0.0482**
Rural	1.00			1.00			1.00		
Geographical region									
Northern	0.87	(0.46–1.67)	0.6782	1.89	(0.76–4.66)	0.1674	0.82	(0.47–1.45)	0.5016
Central	1.01	(0.54–1.90)	0.9806	1.82	(0.76–4.36)	0.1772	1.09	(0.63–1.88)	0.7616
South	1.00			1.00			1.00		

Bold font indicates statistical significance

*CRP* C-reactive protein, *AGP* Alpha-1-acid glycoprotein, *Hb* hemoglobin, *sTfR* soluble transferrin receptors, *OR* odds ratio, *CI* confidence interval

**Table 4:** Univariate analysis of clinical malaria and its effects on anemia, iron deficiency, and soluble transferrin receptors among school aged children

Variables	Low Hb levels			Low ferritin concentration			High sTfR levels		
	OR	95% CI	*P*-value	OR	95% CI	*P*-value	OR	95% CI	*P*-value
Clinical malaria									
Positive	**4.70**	**(3.04–7.27)**	**< 0.0001**	1.49	(0.63–3.09)	0.4197	**3.50**	**(2.41–5.08)**	**< 0.0001**
Negative	1.00			1.00			1.00		
Sex									
Female	1.16	(0.77–1.75)	0.4782	1.65	(0.78–3.50)	0.1867	1.18	(0.83–1.69)	0.3628
Male	1.00			1.00			1.00		
Age									
5–10	**2.14**	**(1.35–3.41)**	**0.0013**	1.19	(0.56–2.51)	0.6507	1.00	(0.69–1.45)	0.9815
11–14	1.00			1.00			1.00		
Fever in last 2 weeks									
Yes	**1.65**	**(1.04–2.65)**	**0.0342**	1.26	(0.51–3.15)	0.6155	**1.90**	**(1.26–2.87)**	**0.0023**
No	1.00			1.00			1.00		
Diarrhea in last 2 weeks									
Yes	1.83	(0.80–4.20)	0.1535	1.70	(0.22–12.9)	0.6070	1.76	(0.84–3.69)	0.1359
No	1.00			1.00			1.00		
Elevated CRP or AGP									
Yes	**4.00**	**(2.58–6.19)**	**< 0.0001**	1.48	(0.65–3.39)	0.3506	**1.93**	**(1.33–2.80)**	**0.0005**
No	1.00			1.00			1.00		
Household hunger scale									
Little to none	0.78	(0.50–1.21)	0.2652	0.86	(0.41–1.80)	0.6970	0.84	(0.58–1.23)	0.3798
Moderate to severe	1.00			1.00			1.00		
Place of residence									
Urban	**0.31**	**(0.12–0.78)**	**0.0125**	0.97	(0.33–2.86)	0.9573	**0.49**	**(0.25–0.99)**	**0.0482**
Rural	1.00			1.00			1.00		
Geographical region									
Northern	0.87	(0.46–1.67)	0.6782	1.89	(0.76–4.66)	0.1674	0.82	(0.47–1.45)	0.5016
Central	1.01	(0.54–1.90)	0.9806	1.82	(0.76–4.36)	0.1772	1.09	(0.63–1.88)	0.7616
South	1.00			1.00			1.00		

Bold font indicates statistical significance

*CRP* C-reactive protein, *AGP* Alpha-1-acid glycoprotein, *Hb* hemoglobin, *sTfR* soluble transferrin receptors, *OR* odds ratio, *CI* confidence interval

### Multivariate analyses of asymptomatic and clinical malaria on biochemical markers of iron

Tables 5 and 6 show the results of multivariate analyses of asymptomatic and clinical malaria on biochemical markers of iron status. Compared to SAC without asymptomatic malaria, SAC with asymptomatic malaria had increased odds of being anaemic (aOR: 3.71, 95% CI 2.29–5.99), *P* < 0.0001 and increased odds of developing functional iron deficit (aOR: 3.00, 95% CI 2.01–4.47), *P* < 0.0001. Similarly, compared to children without clinical malaria, SAC with clinical malaria had increased odds of being anaemic (aOR: 3.54, 95% CI 2.19–5.72), *P* < 0.0001, and increased odds of developing functional iron deficit (aOR: 3.02, 95% confidence interval [CI] 2.02–4.52), *P* < 0.0001.

**Table 5:** Multivariate analysis of asymptomatic malaria and its effect on anemia, iron deficiency, and soluble transferrin receptors among school aged children

Variables	Low Hb levels			Low Ferritin concentration			High sTfR levels		
	aOR	95% CI	*P*-value	aOR	95% CI	*P*-value	aOR	95% CI	*P*-value
Asymptomatic malaria									
Yes	**3.71**	**(2.29–5.99)**	**< 0.0001**	1.03	(0.44–2.37)	0.9491	**3.00**	**(2.01–4.47)**	**< 0.0001**
No	1.00			1.00			1.00		
Sex									
Female	1.02	(0.66–1.59)	0.9286	1.63	(0.77–3.46)	0.2057	1.11	(0.77–1.61)	0.5723
Male	1.00			1.00			1.00		
Age									
5–10	**1.70**	**(1.03–2.79)**	**0.0378**	1.11	(0.51–2.39)	0.7932	0.89	(0.61–1.32)	0.5746
11–14	1.00			1.00			1.00		
Fever in last 2 weeks									
Yes	1.08	(0.64–1.80)	0.7780	1.17	(0.46–3.01)	0.7428	**1.62**	**(1.05–2.48)**	**0.0289**
No	1.00			1.00			1.00		
Diarrhea in last 2 weeks									
Yes	1.32	(0.53–3.29)	0.5498	1.69	(0.22–13.2)	0.6157	1.39	(0.64–3.02)	0.4032
No	1.00			1.00			1.00		
Elevated CRP or AGP									
Yes	**2.63**	**(1.64–4.24)**	**< 0.0001**	1.33	(0.55–3.24)	0.5249	1.37	(0.91–2.05)	0.1344
No	1.00			1.00			1.00		
Household hunger scale									
Little to none	0.85	(0.53–1.38)	0.5122	0.81	(0.37–1.79)	0.6085	1.05	(0.70–1.58)	0.8028
Moderate to severe	1.00			1.00			1.00		
Place of residence									
Urban	0.53	(0.20–1.34)	0.1966	0.93	(0.29–2.99)	0.8995	0.73	(0.36–1.51)	0.3965
Rural	1.00			1.00			1.00		
Geographical region									
Northern	1.03	(0.53–1.97)	0.9379	2.04	(0.78–5.30)	0.1418	0.97	(0.56–1.69)	0.9114
Central	0.85	(0.46–1.57)	0.5987	1.79	(0.74–4.31)	0.1974	1.11	(0.66–1.85)	0.7036
South	1.00			1.00			1.00		

Bold font indicates statistical significance

*CRP* C-reactive protein, *AGP* Alpha-1-acid glycoprotein, *Hb* hemoglobin, *sTfR* soluble transferrin receptors, *aOR* adjusted odds ratio, *CI* confidence interval

**Table 6:** Multivariate analysis of clinical malaria and its effects on anemia, iron deficiency, and soluble transferrin receptors among school aged children

Variables	Low Hb levels			Low ferritin concentration			High sTfR levels		
	aOR	95% CI	*P*-value	aOR	95% CI	*P*-value	aOR	95% CI	*P*-value
Clinical malaria									
Yes	**3.54**	**(2.19–5.72)**	**< 0.0001**	1.17	(0.50–2.75)	0.7225	**3.02**	**(2.02–4.52)**	**< 0.0001**
No	1.00			1.00			1.00		
Sex									
Female	1.02	(0.65–1.58)	0.9470	1.61	(0.76–3.43)	0.2136	1.11	(0.77–1.60)	0.5919
Male	1.00			1.00			1.00		
Age									
5–10	**1.72**	**(1.05–2.83)**	**0.0320**	1.11	(0.52–2.40)	0.7887	0.91	(0.62–1.35)	0.6374
11–14	1.00			1.00			1.00		
Fever in last 2 weeks									
Yes	1.09	(0.65–1.96)	0.7314	1.16	(0.45–2.90)	0.7580	**1.63**	**(1.06–2.50)**	**0.0268**
No	1.00			1.00			1.00		
Diarrhea in last 2 weeks									
Yes	1.19	(0.48–2.96)	0.7151	1.68	(0.22–13.1)	0.6185	1.29	(0.59–2.79)	0.5244
No	1.00			1.00			1.00		
Elevated CRP or AGP									
Yes	**2.65**	**(1.65–4.26)**	**< 0.0001**	1.29	(0.53–3.13)	0.5688	1.35	(0.90–2.053)	0.1461
No	1.00			1.00			1.00		
Household hunger scale									
Little to none	0.86	(0.53–1.38)	0.5280	0.83	(0.38–1.82)	0.6378	1.07	(0.71–1.60)	0.7482
Moderate to severe	1.00			1.00			1.00		
Place of residence									
Urban	0.51	(0.19–1.32)	0.1643	0.95	(0.30–3.06)	0.9343	0.72	(0.35–1.49)	0.3750
Rural	1.00			1.00			1.00		
Geographical region									
Northern	0.98	(0.51–1.88)	0.9472	2.04	(0.77–5.29)	0.1433	0.93	(0.54–1.62)	0.8087
Central	0.80	(0.43–1.49)	0.4840	1.77	(0.73–4.28)	0.2050	1.05	(0.63–1.77)	0.8463
South	1.00			1.00			1.00		

Bold font indicates statistical significance

*CRP* C-reactive protein, *AGP* Alpha-1-acid glycoprotein, *Hb* hemoglobin, *sTfR* soluble transferrin receptors, *aOR* adjusted odds ratio, *CI* confidence interval

## Discussion

The current study aimed to assess the implications of asymptomatic and clinical malaria on biochemical markers of iron status among SAC in Malawi. The study found that the prevalence’s of both asymptomatic and clinical malaria were high and reported at 42% and 41%, respectively. After adjusting for various background characteristics, the current study found that both asymptomatic and clinical malaria infections were significantly associated with anaemia (low Hb levels) and functional ID (high levels of sTfR) but not ID, which is characterized by low SF.

Increased levels of sTfR could be exclusively explained by the elevated rate of RBCs haemolysis associated with *P. falciparum* infections and thus increased demand for iron for erythropoiesis [63]. sTfR are proteins found in the blood that are cleaved from the membrane-bound TfRs found on nearly all cells [64]. Generally, sTfR is considered a useful marker of ID independent of concurrent inflammation or infection [63]. It is known that in the course of RBCs haemolysis, erythroblasts in the bone marrow would increase the presentation of membrane transferrin receptor (TfR) [65]. Increased serum levels of sTfR are associated with tissue receptor expression and provide a clinical measure of the potential for cell proliferation [66]. Thus, when iron supply to the tissues progressively declines, the expression of TfRs increases [67]. Even though the mechanisms underlying the increased levels of sTfR in response to malaria are poorly understood, however, the hepcidin pathway is thought to provide a plausible explanation [68]. In the course of malaria infection, elevated lysis of RBCs, and the subsequent discharge of malaria parasites into the bloodstream, trigger the production of pro-inflammatory cytokines by macrophages, and as result, activate the hepatic synthesis of hepcidin [69]. Hepcidin activates a state of intracellular iron sequestration by internalizing ferroportin, and upregulating the synthesis of SF [70, 71]. Hence, the transient extracellular ID brings about an increase in the membrane-bound TfR [72]. Prior researchers have demonstrated that the value of sTfR remains in proportional association to the concentration of TfR which is expressed on almost all body cells [73]. Thus, sTfR shows the amount of iron that is available for erythropoiesis and constitutes a biomarker of the functional iron compartment in the body [74]. The results of this study are in line with the findings reported in Zambia [56] and Tanzania [59], where malaria parasitemia was associated with a significant increase in plasma sTfR levels. In both studies, it was reported that malaria substantially increased sTfR concentrations, but with modest effects on ID and IDA, results which are consistent with the current study.

In line with previous studies conducted in the other age groups [44, 45, 75], the current study found that both asymptomatic and clinical malaria appeared to decrease Hb concentration significantly, and subsequently increased the odds of anaemia among SAC. As part of the *Plasmodium* life cycle, infection with malaria parasite increases the rate of haemolysis of RBCs directly while suppressing the erythropoiesis process, or in part due to inflammatory cytokines [76, 77]. Eventually, this phenomenon may lead individuals with malaria to exhibit low Hb concentration and also be unable to replace the haemolyzed RBCs with new ones [75]. Further, it is reported that infection with *P. falciparum* may also induce loss of functional body iron and Hb through haemozoin and through urinary excretion [78, 79]. Previous studies have also demonstrated that *P. falciparum* infection may induce anaemia and a decline in Hb levels through altered cytokines balance and inflammation [79]. An imbalance of cytokines such as tumor necrosis factor (TNF), interleukin-6 (IL-6), IL-10, and interferon-gamma (IFN-γ) are implicated in malaria related-inflammation that induce changes in iron absorption and delocalization [44, 80, 81]. As such, iron delocalization may compromise the release of iron from the reticuloendothelial system and increase the uptake of iron from the reticuloendothelial system, resulting in an iron increase in tissues/secretions and ID in blood [82, 83].

Other studies have reported that infection with *P. falciparum* brings about an extensive deformity in the RBC population [84, 85]. The mechanisms that lead to erythrocyte deformability have not been elucidated clearly. However, there is evidence in acute malaria for increased oxidative destruction, which might alter the red cell membrane physiologically and diminish deformability [86–88]. Furthermore, other studies have reported the role of antibody and complement binding in the occurrence of malarial anaemia. For example, a study on severe anaemia showed elevated surface IgG and immune complexes and deficiencies in the complement regulatory proteins CR1 and CD55. As such, the circulating erythrocytes from these subjects were more susceptible to phagocytosis [89, 90]. Additionally, the role of the spleen in anaemia associated with malaria has been recognized and reported. In the course of an acute phase of *P. falciparum* infection, the spleen reconstitutes and expands rapidly. As such, this phenomenon results in elevated clearance capability and decreased splenic activities for the removal of abnormal erythrocytes [91].

In this situation, the spleen eliminates great numbers of relatively rigid red cells, which is a healthy uninfected subject that would be allowed to remain in the circulation [92, 93]. Thus, this process reduces the number of RBCs drastically in the circulation, and subsequent reduces Hb concentration and induces anaemia.

## Strengths and limitations

The use of the nationally-representative sample could permit these results to be generalized among SAC in Malawi. Assessments of Hb, sTfR, and SF objectively minimized the risk of measurement bias. However, the results of the current study should be interpreted with caution. Firstly, the use of a cross-sectional study design cannot provide a causal relationship between asymptomatic, clinical *P. falciparum* infections and the outcomes of interest of the current study. Secondly, the use of RDT to diagnose asymptomatic and clinical *P. falciparum* infections may not be able to detect some infections with lower numbers of malaria parasites circulating in the patient’s bloodstream. Thirdly, the use of the self-reported nature of collecting clinical information, such as fever and diarrhea in the last 2 weeks can lead to recall bias. Fourthly, participants that deemed too ill and those with a physical disability were excluded from the survey. As a result, this would have introduced selection and measurement biases. Lastly, it is documented that indicators for iron status comprise the concentrations of Hb, SF, sTfR, ZPP, reticulocyte, serum iron, and hepcidin and total iron [74, 94]. However, the current study addressed only three of the indicators mentioned above, namely Hb, SF, and sTfR since the MNS had analysed only these parameters.

## Conclusion

The current study found that both asymptomatic and clinical malaria were independent risk factors for anaemia and FID but not ID. The notion that asymptomatic malaria was associated with both anaemia and FID underscores the need for public health programmers to consider adding mass screening and treatment for malaria to existing school-based health programmes in order to improve nutrition status especially iron status among SAC.

## Supplementary Information

**Additional file 1.** Questionnaire for micronutrients survey module.

**Additional file 2.** Questionnaire for the main Malawi Demographic and Health Survey.

## Data Availability

The datasets generated and/or analysed during the current study are available in the MEASURE DHS repository; https://dhsprogram.com/data/dataset/Malawi_Standard-DHS_2015.cfm?flag=1.

## Abbreviations

ACT: Artemisinin-based combination therapy
AGP: Alpha-1-acid glycoprotein
aOR: Adjusted odds ratio
CDC: Centers for Disease Control and Prevention
CI: Confidence interval
CRP: C-reactive protein
DHS: Demographic and Health Survey
EDTA: Ethylenediaminetetraacetic acid
FID: Functional iron deficiency
Hb: Haemoglobin
HIV: Human immunodeficiency virus
ID: Iron deficiency
IDA: Iron deficiency anaemia
MCV: Mean cell volume
MDHS: Malawi Demographic and Health Survey
MEASURE: Monitoring and Evaluation to Assess and Use Results
MNS: Malawi Micronutrient Survey
NHSRC: National Health Sciences Research Committee
PSC: Preschool-aged children
RBCs: Red blood cells
RDT: Rapid Malaria Test
SAC: School-aged children
SF: Serum ferritin
STfR: Soluble transferrin receptors
WHO: World Health Organization
ZPP: Zinc protoporphyrin

## References

[1] Daru J, Colman K, Stanworth SJ, De La Salle B, Wood EM, Pasricha S-R (2017). Serum ferritin as an indicator of iron status: what do we need to know?. Am J Clin Nutr.

[2] Ramachandran N (2014). Persisting undernutrition in India. Causes, consequences and possible solutions.

[3] World Health Organization (WHO) (2001). Iron deficiency anaemia assessment, prevention, and control: a guide for programme managers.

[4] Jeremiah ZA, Uko EK, Buseri FI, Adias TC (2007). Baseline iron status of apparently healthy children in Port Harcourt, Nigeria. Eur J Gen Med.

[5] Kloub MN, Yassin MA (2020). Oral iron therapy-induced neutropenia in patient with iron deficiency anemia. Case Rep Oncol.

[6] Pasricha S-R, Tye-Din J, Muckenthaler MU, Swinkels DW (2021). Iron deficiency. Lancet.

[7] Stoltzfus RJ (2003). Iron deficiency: global prevalence and consequences. Food Nutr Bull.

[8] Uijterschout L, Domellöf M, Vloemans J, Vos R, Hudig C, Bubbers S (2014). The value of Ret-Hb and sTfR in the diagnosis of iron depletion in healthy, young children. Eur J Clin Nutr.

[9] Warner MJ, Kamran MT. Iron deficiency anemia. StatPearls; 2020. 28846348

[10] Ntenda PAM, Chuang K-Y, Tiruneh FN, Chuang Y-C (2017). Multilevel analysis of the effects of individual- and community-level factors on childhood anemia, severe anemia, and hemoglobin concentration in Malawi. J Trop Pediatr.

[11] Semba RD, Bloem MW (2002). The anemia of vitamin A deficiency: epidemiology and pathogenesis. Eur J Clin Nutr.

[12] Calis JCJ, Phiri KS, Faragher EB, Brabin BJ, Bates I, Cuevas LE (2008). Severe anemia in Malawian children. N Engl J Med.

[13] Crawley J. Reducing the burden of anemia in infants and young children in malaria-endemic countries of Africa: from evidence to action. Am J Trop Med Hyg. 2004;25–34. 15331816

[14] Morris CR, Singer ST, Walters MC (2006). Clinical hemoglobinopathies: iron, lungs and new blood. Curr Opin Hematol.

[15] Osendarp SJM, Eilander A, Benton D (2011). Iron deficiency and cognitive development. Lifetime nutritional influences on cognition, behaviour and psychiatric illness.

[16] Halterman JS, Kaczorowski JM, Aligne CA, Auinger P, Szilagyi PG (2001). Iron deficiency and cognitive achievement among school-aged children and adolescents in the United States. Pediatrics.

[17] Cherayil BJ (2010). Iron and immunity: immunological consequences of iron deficiency and overload. Arch Immunol Ther Exp (Warsz).

[18] Oppenheimer SJ (2001). Iron and its relation to immunity and infectious disease. J Nutr.

[19] Abu-Ouf NM, Jan MM (2015). The impact of maternal iron deficiency and iron deficiency anemia on child’s health. Saudi Med J.

[20] Koura GK, Ouedraogo S, Le Port A, Watier L, Cottrell G, Guerra J (2012). Anaemia during pregnancy: impact on birth outcome and infant haemoglobin level during the first 18months of life. Trop Med Int Health.

[21] Suchdev PS, Williams AM, Mei Z, Flores-Ayala R, Pasricha SR, Rogers LM (2017). Assessment of iron status in settings of inflammation: challenges and potential approaches. Am J Clin Nutr.

[22] Hurrell RF (2012). Influence of inflammatory disorders and infection on iron absorption and efficacy of iron-fortified foods. Nestle Nutr Inst Workshop Ser.

[23] Pfeiffer CM, Looker AC (2017). Laboratory methodologies for indicators of iron status: strengths, limitations, and analytical challenges. Am J Clin Nutr.

[24] Northrop-Clewes CA (2008). Interpreting indicators of iron status during an acute phase response—lessons from malaria and human immunodeficiency virus. Ann Clin Biochem.

[25] Joint World Health Organization/Centers for Disease Control and Prevention Technical Consultation on the Assessment of Iron Status at the Population Level. Assessing the iron status of populations: including literature reviews: report of a Joint World Health Organization/Centers for Disease Control and Prevention Technical Consultation on the Assessment of Iron Status at the Population Level, Geneva, Switzerland, 2nd edition. Geneva: World Health Organization; 2004.

[26] Thurnham DI, McCabe GP. Influence of infection and inflammation on biomarkers of nutritional status with an emphasis on vitamin A and iron. Report: priorities in the assessment of vitamin A and iron status and in populations Panama City, Panama. World Health Organization. 2010;15.

[27] Teshome EM, Prentice AM, Demir AY, Andango PEA, Verhoef H (2017). Diagnostic utility of zinc protoporphyrin to detect iron deficiency in Kenyan preschool children: a community-based survey. BMC Hematol.

[28] Davidson RJ, Hamilton PJ (1978). High mean red cell volume: its incidence and significance in routine haematology. J Clin Pathol.

[29] Knovich MA, Storey JA, Coffman LG, Torti SV, Torti FM (2009). Ferritin for the clinician. Blood Rev.

[30] Kwenti TE, Kwenti TDB, Njunda LA, Latz A, Tufon KA, Nkuo-Akenji T (2017). Identification of the *Plasmodium* species in clinical samples from children residing in five epidemiological strata of malaria in Cameroon. Trop Med Health.

[31] World Health Organization (WHO) (2020). World malaria report 2020: 20 years of global progress and challenges.

[32] World Health Organization (WHO). Malaria. Geneva: WHO; 2020. https://www.who.int/news-room/fact-sheets/detail/malaria. Accessed 16 Mar 2021.

[33] Tizifa TA, Kabaghe AN, McCann RS, van den Berg H, Van Vugt M, Phiri KS (2018). Prevention efforts for malaria. Curr Trop Med Rep.

[34] Dhiman S (2019). Are malaria elimination efforts on right track? An analysis of gains achieved and challenges ahead. Infect Dis Poverty.

[35] Zamawe COF, Nakamura K, Shibanuma A, Jimba M (2016). The effectiveness of a nationwide universal coverage campaign of insecticide-treated bed nets on childhood malaria in Malawi. Malar J.

[36] World Health Organization (2014). WHO policy brief for the implementation of intermittent preventive treatment of malaria in pregnancy using sulfadoxine–pyrimethamine (IPTp-SP).

[37] Walldorf JA, Cohee LM, Coalson JE, Bauleni A, Nkanaunena K, Kapito-Tembo A (2015). School-age children are a reservoir of malaria infection in Malawi. PLoS ONE.

[38] Coalson JE, Cohee LM, Buchwald AG, Nyambalo A, Kubale J, Seydel KB (2018). Simulation models predict that school-age children are responsible for most human-to-mosquito *Plasmodium falciparum* transmission in southern Malawi. Malar J.

[39] Cohee LM, Opondo C, Clarke SE, Halliday KE, Cano J, Shipper AG (2020). Preventive malaria treatment among school-aged children in sub-Saharan Africa: a systematic review and meta-analyses. Lancet Glob Health.

[40] Vorasan N, Pan-Ngum W, Jittamala P, Maneeboonyang W, Rukmanee P, Lawpoolsri S (2015). Long-term impact of childhood malaria infection on school performance among school children in a malaria endemic area along the Thai-Myanmar border. Malar J.

[41] Brooker SJ, Clarke S, Fernando D, Gitonga CW, Nankabirwa J, Schellenberg D (2017). Malaria in middle childhood and adolescence. Disease control priorities, third edition (volume 8): child and adolescent health and development.

[42] Mathanga DP, Halliday KE, Jawati M, Verney A, Bauleni A, Sande J (2015). The high burden of malaria in primary school children in southern Malawi. Am J Trop Med Hyg.

[43] Nankabirwa J, Wandera B, Kiwanuka N, Staedke SG, Kamya MR, Brooker SJ (2013). Asymptomatic *Plasmodium* infection and cognition among primary schoolchildren in a high malaria transmission setting in Uganda. Am J Trop Med Hyg.

[44] Ntenda PAM, Chilumpha S, Mwenyenkulu ET, Kazambwe JF, El-Meidany W (2019). Clinical malaria and the potential risk of anaemia among preschool-aged children: a population-based study of the 2015–2016 Malawi micronutrient survey. Infect Dis Poverty.

[45] Autino B, Corbett Y, Castelli F, Taramelli D (2012). Pathogenesis of malaria in tissues and blood. Mediterr J Hematol Infect Dis.

[46] National Statistical Office (NSO), Community Health Sciences Unit (CHSU) [Malawi], Centers for Disease Control and Prevention (CDC), and University Emory. Malawi micronutrient survey 2015–16. Atlanta GA, USA NSO, CHSU, CDC Univ Emory; 2017.

[47] National Statistical Office, Community Health Sciences (CHSU), Center for Disease Control and Prevention (CDC) and EU. Malawi demographic and health survey 2015–16. Antlanta, GA, USA, NSO, CHISU, CDC Emory Univ; 2017.

[48] Ängelhol Sweden. HemoCue® Hb 301 system. HemoCue AB, Ängelhol Sweden 2021. http://www.hemocue.com/en/solutions. Accessed 16 May 2021.

[49] HemoCue. Anemia screening from the pioneers HemoCue ® Hb 301 system. Sweden 2015. https://www.hemocue.com/-/media/hemocue-images/hemocuedotcom-images/product-images/hb/pdf-folders-etc/web-update-01092015.pdf. Accessed 9 May 2019.

[50] HemoCue AB. HemoCue® Hb 301 Microcuvettes. Kuvettgatan 1, SE-262 71, Ängelholm, Sweden n.d. https://www.hemocue.us/wp-content/uploads/2020/06/Product_Insert_Hb_301.pdf. Accessed 6 June 2022.

[51] HemoCue AB. HemoCue® Hb 301; operating manual. Kuvettgatan, SE-262 71, Ängelholm, Sweden; 2015. https://www.michigan.gov/-/media/Project/Websites/mdhhs/Folder3/Folder69/Folder2/Folder169/Folder1/Folder269/HB301_Operator_manual.pdf?rev=9dc20799e9a04979a25c08e5050e3cdb.

[52] Obeagu EI, Chijioke U, Stella EI (2019). Malaria rapid diagnostic test (RDTs). Ann Clin Lab Res.

[53] Erhardt JG, Estes JE, Pfeiffer CM, Biesalski HK, Craft NE (2004). Combined measurement of ferritin, soluble transferrin receptor, retinol binding protein, and C-reactive protein by an inexpensive, sensitive and simple sandwich enzyme-linked immunosorbent assay technique. J Nutr.

[54] Namaste SM, Rohner F, Huang J, Bhushan NL, Flores-Ayala R, Kupka R (2017). Adjusting ferritin concentrations for inflammation: biomarkers reflecting inflammation and nutritional determinants of anemia (BRINDA) project. Am J Clin Nutr.

[55] Gebreegziabher T, Stoecker BJ (2017). Iron deficiency was not the major cause of anemia in rural women of reproductive age in Sidama zone, southern Ethiopia: a cross-sectional study. PLoS ONE.

[56] Barffour MA, Schulze KJ, Coles CL, Chileshe J, Kalungwana N, Siamusantu W (2018). Malaria exacerbates inflammation-associated elevation in ferritin and soluble transferrin receptor with only modest effects on iron deficiency and iron deficiency anaemia among rural Zambian children. Trop Med Int Heal.

[57] Phiri KS, Calis JCJ, Siyasiya A, Bates I, Brabin B, van Hensbroek MB (2009). New cut-off values for ferritin and soluble transferrin receptor for the assessment of iron deficiency in children in a high infection pressure area. J Clin Pathol.

[58] Thomas DW, Hinchliffe RF, Briggs C, Macdougall IC, Littlewood T, Cavill I (2013). Guideline for the laboratory diagnosis of functional iron deficiency. Br J Haematol.

[59] Menendez C, Quinto LL, Kahigwa E, Alvarez L, Fernandez R, Gimenez N (2001). Effect of malaria on soluble transferrin receptor levels in Tanzanian infants. Am J Trop Med Hyg.

[60] Mutanda AL, Cheruiyot P, Hodges JS, Ayodo G, Odero W, John CC (2014). Sensitivity of fever for diagnosis of clinical malaria in a Kenyan area of unstable, low malaria transmission. Malar J.

[61] World Health Organization (WHO). Diarrhoea. Geneva: WHO Press; 2031. https://www.who.int/topics/diarrhoea/en/. Accessed 19 Mar 2019.

[62] Ballard T, Coates J, Swindale A, Deitchler M. Household hunger scale: indicator definition and measurement guide. 2011. Washington, DC: Food and nutrition technical assistance II project, FHI; 2016. p. 360

[63] Mockenhaupt FP, May J, Stark K, Falusi AG, Meyer CG, Bienzle U (1999). Serum transferrin receptor levels are increased in asymptomatic and mild *Plasmodium falciparum*-infection. Haematologica.

[64] Kundrapu S, Noguez J (2018). Laboratory assessment of anemia. Adv Clin Chem.

[65] Verhoef H, West CE, Ndeto P, Burema J, Beguin Y, Kok FJ (2001). Serum transferrin receptor concentration indicates increased erythropoiesis in Kenyan children with asymptomatic malaria. Am J Clin Nutr.

[66] Alarcón B, Fresno M, Delves PJ, Roitt IM (1998). Transferrin receptor (CD71). Encyclopedia of immunology.

[67] World Health Organization (WHO). Serum transferrin receptor levels for the assessment of iron status and iron deficiency in populations. WHO/NMH/NHD/EPG/14.6. Geneva: World Health Organization; 2014.

[68] Menendez C, Fleming AF, Alonso PL (2000). Malaria-related anaemia. Parasitol Today.

[69] Othoro C, Lal AA, Nahlen B, Koech D, Orago ASS, Udhayakumar V (1999). A low interleukin-10 tumor necrosis factor-α ratio is associated with malaria anemia in children residing in a holoendemic malaria region in western Kenya. J Infect Dis.

[70] Ganz T, Nemeth E (2006). Regulation of iron acquisition and iron distribution in mammals. Biochim Biophys Acta (BBA) Mol Cell Res.

[71] Ganz T, Nemeth E (2009). Iron sequestration and anemia of inflammation. Seminars in hematology.

[72] Nemeth E, Ganz T (2006). Regulation of iron metabolism by hepcidin. Annu Rev Nutr.

[73] Oustamanolakis P, Koutroubakis IE, Kouroumalis EA (2011). Diagnosing anemia in inflammatory bowel disease: beyond the established markers. J Crohn’s Colitis.

[74] Krawiec P, Pac-Kożuchowska E (2020). Biomarkers and hematological indices in the diagnosis of iron deficiency in children with inflammatory bowel disease. Nutrients.

[75] Green HK, Sousa-Figueiredo JC, Basáñez MG, Betson M, Kabatereine NB, Fenwick A (2011). Anaemia in Ugandan preschool-aged children: the relative contribution of intestinal parasites and malaria. Parasitology.

[76] Schofield L, Grau GE (2005). Immunological processes in malaria pathogenesis. Nat Rev Immunol.

[77] Awandare GA, Kempaiah P, Ochiel DO, Piazza P, Keller CC, Perkins DJ (2011). Mechanisms of erythropoiesis inhibition by malarial pigment and malaria-induced proinflammatory mediators in an in vitro model. Am J Hematol.

[78] Stoltzfus RJ, Chwaya HM, Albonico M, Schulze KJ, Savioli L, Tielsch JM (1997). Serum ferritin, erythrocyte protoporphyrin and hemoglobin are valid indicators of iron status of school children in a malaria-holoendemic population. J Nutr.

[79] Perkins DJ, Were T, Davenport GC, Kempaiah P, Hittner JB, Ong’echa JM (2011). Severe malarial anemia: innate immunity and pathogenesis. Int J Biol Sci.

[80] Raza A, Khan MS, Ghanchi NK, Raheem A, Beg MA (2014). Tumour necrosis factor, interleukin-6 and interleukin-10 are possibly involved in *Plasmodium vivax*-associated thrombocytopaenia in southern Pakistani population. Malar J.

[81] Kany S, Vollrath JT, Relja B (2019). Cytokines in inflammatory disease. Int J Mol Sci.

[82] Paesano R, Natalizi T, Berlutti F, Valenti P (2012). Body iron delocalization: the serious drawback in iron disorders in both developing and developed countries. Pathog Glob Health.

[83] Yiannikourides A, Latunde-Dada G (2019). A short review of iron metabolism and pathophysiology of iron disorders. Medicines.

[84] Mohandas N, An X (2012). Malaria and human red blood cells. Med Microbiol Immunol.

[85] Moxon CA, Grau GE, Craig AG (2011). Malaria: modification of the red blood cell and consequences in the human host. Br J Haematol.

[86] Hosseini SM, Feng JJ (2012). How malaria parasites reduce the deformability of infected red blood cells. Biophys J.

[87] Percário S, Moreira DR, Gomes BAQ, Ferreira MES, Gonçalves ACM, Laurindo PSOC (2012). Oxidative stress in malaria. Int J Mol Sci.

[88] Becker K, Tilley L, Vennerstrom JL, Roberts D, Rogerson S, Ginsburg H (2004). Oxidative stress in malaria parasite-infected erythrocytes: host–parasite interactions. Int J Parasitol.

[89] Stoute JA, Odindo AO, Owuor BO, Mibei EK, Opollo MO, Waitumbi JN (2003). Loss of red blood cell-complement regulatory proteins and increased levels of circulating immune complexes are associated with severe malarial anemia. J Infect Dis.

[90] Kai OK, Roberts DJ (2008). The pathophysiology of malarial anaemia: where have all the red cells gone?. BMC Med.

[91] White NJ (2017). Malaria parasite clearance. Malar J.

[92] Chotivanich K, Udomsangpetch R, McGready R, Proux S, Newton P, Pukrittayakamee S (2002). Central role of the spleen in malaria parasite clearance. J Infect Dis.

[93] Wang H, Li S, Cui Z, Qin T, Shi H, Ma J (2021). Analysis of spleen histopathology, splenocyte composition and haematological parameters in four strains of mice infected with *Plasmodium **berghei* K173. Malar J.

[94] Aimone-Gastin I (2006). Biochemical markers of iron status. Nephrol Ther.

