# Supplementary material for: Implication of asymptomatic and clinical Plasmodium falciparum infections on biomarkers of iron status among school-aged children in Malawi
Source: Malar J. 2022 Oct 1;21:278. doi: 10.1186/s12936-022-04297-1 (PMC9526385; doi:10.1186/s12936-022-04297-1)
Supplement: Supplementary file 1 — Additional file 1. Questionnaire for micronutrients survey module. [file 12936_2022_4297_MOESM1_ESM.pdf]

FORMATTING DATE: 06 Dec 2015
ENGLISH LANGUAGE: 06 Dec 2015

2015-2016 MALAWI DEMOGRAPHIC AND HEALTH SURVEY
MALAWI GOVERNMENT - NATIONAL STATISTICAL OFFICE
MALAWI MICRONUTRIENT MODULE

IDENTIFICATION

PLACE NAME ______________________________

NAME OF HOUSEHOLD HEAD ______________________________

CLUSTER NUMBER . . . . [ ][ ][ ][ ]

HOUSEHOLD NUMBER . . . . [ ][ ][ ][ ]

PLACE BAR CODE LABEL. . . . . PUT THE HOUSEHOLD QUESTIONNAIRE BAR CODE LABEL HERE.

PERMISSION FOR REVISIT (1=GRANTED, 2=NOT GRANTED) . . . . [ ]

HOUSEHOLD SELECTED FOR MRDR TESTING? (1=YES, 2=NO) . . . . [ ]

FIELDWORKER VISITS

	1	2	3	FINAL VISIT
DATE	______	______	______	DAY [ ][ ]
				MONTH [ ][ ]
	______	______	______	YEAR [2][0][ ][ ]
FIELDWORKER'S NAME	______	______	______	FIELDW. NO. [ ][ ]
RESULT*	______	______	______	RESULT* [ ][ ]
NEXT VISIT: DATE	______	______		TOTAL NUMBER OF VISITS [ ]
TIME	______	______		

*RESULT CODES:
1 COMPLETED
2 NO HOUSEHOLD MEMBER AT HOME OR NO COMPETENT RESPONDENT AT HOME AT TIME OF VISIT
3 ENTIRE HOUSEHOLD ABSENT FOR EXTENDED PERIOD OF TIME
4 POSTPONED
5 REFUSED
6 PARTLY COMPLETED
7 DWELLING VACANT OR ADDRESS NOT A DWELLING
8 DWELLING DESTROYED
9 DWELLING NOT FOUND
10 OTHER ______________________________ (SPECIFY)

TOTAL ELIGIBLE WOMEN 15-49 YEARS [ ][ ]

TOTAL ELIGIBLE MEN 20-54 YEARS [ ][ ]

TOTAL ELIGIBLE CHILDREN 0-4 YEARS [ ][ ]

TOTAL ELIGIBLE CHILDREN 5-14 YEARS [ ][ ]

LANGUAGE OF QUESTIONNAIRE** [0][1]   LANGUAGE OF INTERVIEW** [ ][ ]   NATIVE LANGUAGE OF RESPONDENT** [ ][ ]   TRANSLATOR (YES = 1, NO = 2) [ ]

LANGUAGE OF QUESTIONNAIRE** ENGLISH

**LANGUAGE CODES:
01 ENGLISH
02 CHICHEWA
03 TUMBUKA
04 OTHER ______________ (SPECIFY)

SUPERVISOR		OFFICE EDITOR	KEYED BY
______________ NAME	[ ][ ] NUMBER	[ ][ ] NUMBER	[ ][ ] NUMBER

NO.	QUESTIONS AND FILTERS	CODING CATEGORIES	SKIP
100	ASK CONSENT FOR FOOD FORTIFICATION COVERAGE INFORMATION FROM HEAD OF HOUSEHOLD/OTHER ADULT.	Hello. My name is ______________________________________. I am working with The National Statistical Office (NSO). As part of the Demographic and Health Survey we would like to ask you some questions about some of the foods that you may have in your home. We are particularly interested in learning more about salt, sugar and oil. If you agree we would like to take a very small sample of any of these foods that you have, so that we can test whether or not they have been fortified with vitamin A or iodine. In exchange we will replace any items you have given us. This information will help the Ministry of Health understand better what foods people have in their homes and the quality of the foods.<br><br>Do you have any questions?<br>You can say yes or no. It is up to you to decide.<br>Will you allow us to talk to you about some foods you may have in your home?	
101	CIRCLE THE CODE AND SIGN YOUR NAME.	GRANTED . . . . 1<br>______________ (SIGN)<br>REFUSED . . . . 2<br>OTHER ______________ 6 (SPECIFY)	 <br>2, 6 → 200
102	Do you have salt in your household today?<br><br>IF YES: Please can we see the salt you have in the household?	YES . . . . 1<br>NO 2	 → 106
103	OBSERVE THE BRAND OF THE SALT<br><br>RECORD OBSERVATION.	BOTSALT . . . . 11<br>MALAWI . . . . 12<br>RAB'S . . . . 13<br>FA RAHIMA . . . . 14<br>SEAFRESH . . . . 15<br>FAMILY PRIDE . . . . 16<br><br>NOT LABELED . . . . 95<br><br>OTHER ______________ 96 (SPECIFY)	 <br><br><br><br><br><br>95 → 105
104	RECORD IF THE SALT IS LABELLED AS IODIZED	YES, LABELLED AS IODIZED . . . . 1<br>NO, NOT LABELLED AS IODIZED . . . . 2<br>DON'T KNOW 8	
105	Please may we take a small sample of your salt so that we can test it for iodine?<br><br>PUT THE SALT SPECIMEN BAR CODE LABEL ON THE SALT CONTAINER AND THE SALT FORM LABEL ON THE FOOD CONTROL FORM [B]	PUT THE SALT QUESTIONNAIRE LABEL HERE<br><br>SALT NOT COLLECTED . . . . 9995<br><br>OTHER ______________ 9996 (SPECIFY REASON)	
` 106	Do you have any sugar in your household today?<br><br>IF YES: Please can we see the main type of sugar you have in the household?	YES . . . . 1<br>NO 2	 → 110
107	OBSERVE THE BRAND OF THE SUGAR<br><br>RECORD OBSERVATION.	ILOVO . . . . 11<br><br>NOT LABELED . . . . 95<br><br>OTHER ______________ 96 (SPECIFY)	 <br>95 → 109

FOOD FORTIFICATION

NO.	QUESTIONS AND FILTERS	CODING CATEGORIES	SKIP
108	RECORD IF THE SUGAR IS LABELLED AS FORTIFIED WITH VITAMIN A	YES, FORTIFIED WITH VITAMIN A .... 1<br>NO, NOT LABELLED AS FORTIFIED WITH VIT. A 2<br>DON'T KNOW 8	
109	Please may we take a small sample of your sugar so that we can test it for vitamin A?<br><br>PUT THE SUGAR SPECIMEN BAR CODE LABEL ON THE SUGAR CONTAINER AND THE SUGAR FORM LABEL ON THE FOOD CONTROL FORM [B]	PUT THE SUGAR QUESTIONNAIRE BAR CODE LABEL HERE.<br><br>SUGAR NOT COLLECTED .... 9995<br><br>OTHER ______________ 9996<br>(SPECIFY REASON)	
110	Do you have any oil in your household today?<br><br>IF YES: Please can we see the main type of oil you have in the household?	YES .... 1<br>NO 2	 → 114
111	OBSERVE THE BRAND OF THE OIL<br><br>RECORD OBSERVATION.	KAZINGA .... 11<br>KUKOMA .... 12<br>SUPERSTAR .... 13<br>MULAWE .... 14<br>DELIGHT .... 15<br>SUNFOIL .... 16<br>RINA .... 17<br><br>NOT LABELED .... 95<br><br>OTHER ______________ 96<br>(SPECIFY)	 <br><br><br><br><br><br><br>→ 113
112	RECORD IF THE OIL IS LABELLED AS FORTIFIED WITH VITMAIN A	YES, FORTIFIED WITH VITAMIN A .... 1<br>NO, NOT LABELLED AS FORTIFIED WITH VIT. A 2<br>DON'T KNOW 8	
113	Please may we take a small sample of your oil so that we can test it for vitamin A?<br><br>PUT THE OIL SPECIMEN BAR CODE LABEL ON THE OIL TUBE CONTAINER AND THE OIL FORM LABEL ON THE FOOD CONTROL FORM [B]	PUT THE OIL QUESTIONNAIRE BAR CODE LABEL HERE.<br><br>OIL NOT COLLECTED .... 9995<br><br>OTHER ______________ 9996<br>(SPECIFY REASON)	
114	Do you have Blue Band Margarine in your house currently?	YES .... 1<br>NO 2	
115	In the past 7 days, did anyone in your household purchase:<br><br>a) Wheat flour?<br>b) Pasta/Spaghetti?<br>c) Bread?<br>d) Biscuits/Cookies?<br>e) Mandazi?<br>f) Cakes<br>g) Maize flour?	YES NO<br><br>a) WHEAT FLOUR .... 1 2<br>b) PASTA/SPAGHETTI .... 1 2<br>c) BREAD .... 1 2<br>d) BISCUITS/COOKIES .... 1 2<br>e) MANDAZI .... 1 2<br>f) CAKES .... 1 2<br>g) MAIZE FLOUR 1 2	

FOOD FORTIFICATION

NO.	QUESTIONS AND FILTERS	CODING CATEGORIES	SKIP
116	In the past 4 weeks (30 days), was there ever no food to eat of any kind in your house because of lack of resources to get food?	YES . . . . 1<br>NO 2	 → 118
117	How often did this happen in the past 4 weeks (30 days)?	RARELY (1–2 TIMES) . . . . 1<br>SOMETIMES (3–10 TIMES) . . . . 2<br>OFTEN (MORE THAN 10 TIMES) 3	
118	In the past 4 weeks (30 days), did you or any household member go to sleep at night hungry because there was not enough food?	YES . . . . 1<br>NO 2	 → 120
119	How often did this happen in the past 4 weeks (30 days)?	RARELY (1–2 TIMES) . . . . 1<br>SOMETIMES (3–10 TIMES) . . . . 2<br>OFTEN (MORE THAN 10 TIMES) 3	
120	In the past 4 weeks (30 days), did you or any household member go a whole day and night without eating anything at all because there was not enough food?	YES . . . . 1<br>NO 2	 → 122
121	How often did this happen in the past 4 weeks (30 days)?	RARELY (1–2 TIMES) . . . . 1<br>SOMETIMES (3–10 TIMES) . . . . 2<br>OFTEN (MORE THAN 10 TIMES) 3	
122	Has your household received coupons for the Farm Input Subsidy Program (FISP) for this season (2015-2016)?	YES . . . . 1<br>NO . . . . 2<br>DON'T KNOW…………… 8	
123	Does your household participate in the social cash transfer programme?	YES . . . . 1<br>NO . . . . 2<br>DON'T KNOW…………… 8	
124	Is your household on the Malawian Vulnerability Assessment Committee (MVAC) list this season (2015-2016)?	YES . . . . 1<br>NO . . . . 2<br>DON'T KNOW…………… 8	
125	Did your household receive food or cash support during last year's (2014-2015) drought and flood response from the Malawian Vulnerability Assessment Committee (MVAC)?	YES . . . . 1<br>NO . . . . 2<br>DON'T KNOW…………… 8	
126	RECORD IF REPLACEMENT ITEMS WERE PROVIDED TO HOUSEHOLD	YES, REPLACEMENT ITEMS PROVIDED . . . . 1<br>NO, REPLACEMENT ITEMS NOT PROVIDED . . 2	

BIOLOGICAL INFORMATION FOR CHILDREN AGE 6-59 MONTHS

200	CHECK COLUMN 7 IN HOUSEHOLD QUESTIONNAIRE. RECORD THE LINE NUMBER AND NAME FOR ALL CHILDREN 0-4 YEARS IN QUESTION 201; IF MORE THAN SIX CHILDREN, USE ADDITIONAL QUESTIONNAIRE BOOKLET AND USE THE DUPLICATE HH LABEL(S).			
		CHILD 1	CHILD 2	CHILD 3
201	CHECK HOUSEHOLD QUESTIONNAIRE:<br>LINE NUMBER FROM COLUMN 1.<br><br>NAME FROM COLUMN 2.	LINE NUMBER . . . . ☐☐<br><br>NAME ________	LINE NUMBER . . . . ☐☐<br><br>NAME ________	LINE NUMBER . . . . ☐☐<br><br>NAME ________
202	What is (NAME)'s date of birth?	DAY . . . . ☐☐<br>MONTH . . . . ☐☐<br>YEAR . . . . ☐☐☐☐	DAY . . . . ☐☐<br>MONTH . . . . ☐☐<br>YEAR . . . . ☐☐☐☐	DAY . . . . ☐☐<br>MONTH . . . . ☐☐<br>YEAR . . . . ☐☐☐☐
203	PRESCHOOL CHILD LABEL	PUT THE PRESCHOOL CHILD QUESTIONNAIRE BAR CODE LABEL HERE.	PUT THE PRESCHOOL CHILD QUESTIONNAIRE BAR CODE LABEL HERE.	PUT THE PRESCHOOL CHILD QUESTIONNAIRE BAR CODE LABEL HERE.
204	CHECK 202: CHILD BORN IN 2010-2015?	YES . . . . 1<br>NO . . . . 2<br>(SKIP TO 253)	YES . . . . 1<br>NO . . . . 2<br>(SKIP TO 253)	YES . . . . 1<br>NO . . . . 2<br>(SKIP TO 253)
205	CHECK 202: CHILD AGE 0-5 MONTHS, I.E., WAS CHILD BORN IN MONTH OF INTERVIEW OR 5 PREVIOUS MONTHS?	0-5 MONTHS . . . . 1<br>(SKIP TO 253)<br><br>6 MONTHS-4 YEARS 2	0-5 MONTHS . . . . 1<br>(SKIP TO 253)<br><br>6 MONTHS-4 YEARS 2	0-5 MONTHS . . . . 1<br>(SKIP TO 253)<br><br>6 MONTHS-4 YEARS 2
206	CHILD'S SEX	FEMALE . . . . 1<br>MALE 2	FEMALE . . . . 1<br>MALE 2	FEMALE . . . . 1<br>MALE 2
207	ASK CONSENT FOR ANTHROPOMETRY AND BIOLOGICAL TESTING<br>FROM PARENT/OTHER ADULT.	As part of this survey we are asking a parent of some children to allow us to weigh and measure their children and check them for Oedma. If your child has severe acute malnutrition we will refer your child to the nearest facility that can help you. In addition to weighing and measuring your child we would like to take a sample of his/her blood and urine. The tests are safe. Some tests may cause your child slight discomfort, such as taking a blood sample. For all tests, there will be a brand new set of equipment used to take your child's blood and collect their urine, which is clean and completely safe. The equipment will be thrown away after it has been used on your child.<br>With the blood we will test your child for anemia and malaria. Anemia is a serious health problem that usually results from poor nutrition, infection, or chronic disease. Malaria can also be serious and can lead to your child becoming anemic or making the anemia worse. You will be given these results immediately. If needed your child will be referred to a local health facility for treatment. The rest of the blood will be sent to a laboratory to be tested for other vitamins and minerals, such as vitamin A and iron. The results from these tests will not be reported back to you as it will take some time to process the blood.The results will be kept strictly confidential.<br>This information will help the Ministry of Health understand better what problems children in Malawi are experiencing and help them to improve the health and nutrition programs here, which will benefit all children in Malawi.<br><br>Do you have any questions?<br>You can say yes or no. It is up to you to decide.<br>Will you allow (NAME OF CHILD) to participate in these tests?		
208	CIRCLE THE CODE AND SIGN YOUR NAME.	AGREED, ANTHROPOM. MEASURES ONLY . . . . 1<br>AGREED, BLOOD TEST ONLY . 2<br>AGREED, URINE TEST ONLY . 3<br>AGREED, ANTHROPO& BLOOD TEST ONLY 4<br>AGREED, ANTHROPO& URINE TEST ONLY . . . . 5<br>AGREED, BLOOD& URINE TESTS ONLY . . . . 6<br>AGREED **ALL**, ANTHROPO & BLOOD&URINE TESTS . 7<br>REFUSED . . . . 8<br>________<br>(SIGN)<br>NOT PRESENT/OTHER . 9<br>(SKIP TO 253)	AGREED, ANTHROPOM. MEASURES ONLY . . . . 1<br>AGREED, BLOOD TEST ONLY . 2<br>AGREED, URINE TEST ONLY . 3<br>AGREED, ANTHROPO& BLOOD TEST ONLY 4<br>AGREED, ANTHROPO& URINE TEST ONLY . . . . 5<br>AGREED, BLOOD& URINE TESTS ONLY . . . . 6<br>AGREED **ALL**, ANTHROPO & BLOOD&URINE TESTS . 7<br>REFUSED . . . . 8<br>________<br>(SIGN)<br>NOT PRESENT/OTHER . 9<br>(SKIP TO 253)	AGREED, ANTHROPOM. MEASURES ONLY . . . . 1<br>AGREED, BLOOD TEST ONLY . 2<br>AGREED, URINE TEST ONLY . 3<br>AGREED, ANTHROPO& BLOOD TEST ONLY 4<br>AGREED, ANTHROPO& URINE TEST ONLY . . . . 5<br>AGREED, BLOOD& URINE TESTS ONLY . . . . 6<br>AGREED **ALL**, ANTHROPO & BLOOD&URINE TESTS . 7<br>REFUSED . . . . 8<br>________<br>(SIGN)<br>NOT PRESENT/OTHER . 9<br>(SKIP TO 253)
209	NURSE: ENTER YOUR ID NUMBER	☐☐<br>ID NUMBER	☐☐<br>ID NUMBER	☐☐<br>ID NUMBER

200	CHECK COLUMN 7 IN HOUSEHOLD QUESTIONNAIRE. RECORD THE LINE NUMBER AND NAME FOR ALL CHILDREN 0-4 YEARS IN QUESTION 201; IF MORE THAN SIX CHILDREN, USE ADDITIONAL QUESTIONNAIRE BOOKLET AND USE THE DUPLICATE HH LABEL(S).			
		CHILD 1	CHILD 2	CHILD 3
201	CHECK HOUSEHOLD QUESTIONNAIRE: LINE NUMBER FROM COLUMN 1.<br><br>NAME FROM COLUMN 2.	LINE NUMBER . . . .<br>NAME ___	LINE NUMBER . . . .<br>NAME ___	LINE NUMBER . . . .<br>NAME ___
210A	In the last month, has (NAME OF CHILD) taken iron tablets/ syrups/ Multiple micronutrient powders?<br><br>SHOW COMMON IRON TABLETS/ SYRUP/ MNP IN MALAWI.	YES . . . . 1<br>NO 2	YES . . . . 1<br>NO 2	YES . . . . 1<br>NO 2
210	In the last six months, has (NAME OF CHILD) received deworming treatment?	YES . . . . 1<br>NO 2	YES . . . . 1<br>NO 2	YES . . . . 1<br>NO 2
211	In the last month, has (NAME OF CHILD) received any therapeutic foods, such as PLUMPY NUT [CHIPONDE]?<br><br>SHOW SACHET.	YES . . . . 1<br>NO 2	YES . . . . 1<br>NO 2	YES . . . . 1<br>NO 2
212	In the last month, has (NAME OF CHILD) received a vitamin A capsule?	YES . . . . 1<br>NO 2	YES . . . . 1<br>NO 2	YES . . . . 1<br>NO 2
213	Has (NAME OF CHILD) had a fever in the last 2 weeks?	YES . . . . 1<br>NO 2	YES . . . . 1<br>NO 2	YES . . . . 1<br>NO 2
214	Has (NAME OF CHILD) had a fever in the last 24 hours?	YES . . . . 1<br>NO 2	YES . . . . 1<br>NO 2	YES . . . . 1<br>NO 2
215	Has (NAME OF CHILD) had diarrhea in the last 2 weeks?	YES . . . . 1<br>NO 2	YES . . . . 1<br>NO 2	YES . . . . 1<br>NO 2
216	Has (NAME OF CHILD) had a cough or breathing problems in the last 2 weeks?	YES . . . . 1<br>NO 2	YES . . . . 1<br>NO 2	YES . . . . 1<br>NO 2
217	Has (NAME OF CHILD) been ill with malaria in the last 2 weeks?	YES . . . . 1<br>NO 2	YES . . . . 1<br>NO 2	YES . . . . 1<br>NO 2
218	Have you noticed blood in (NAME OF CHILD)'s urine in the past 2 weeks?	YES . . . . 1<br>NO 2	YES . . . . 1<br>NO 2	YES . . . . 1<br>NO 2
219	In the last six months, has (NAME OF CHILD) received a blood transfusion?	YES . . . . 1<br>NO 2	YES . . . . 1<br>NO 2	YES . . . . 1<br>NO 2
220	At what time approximately did (NAME OF CHILD) eat her/his most recent meal or was breastfed?	HOURS . . . .<br>MINUTES	HOURS . . . .<br>MINUTES	HOURS . . . .<br>MINUTES . . . .
221	CHECK 208:<br>AGREED FOR BLOOD TEST	CODE '2', '4', '6' OR '7' CIRCLED<br>CODE '2', '4', 6' OR '7' NOT CIRCLED (SKIP TO 230)	CODE '2', '4', '6' OR '7' CIRCLED<br>CODE '2', '4', 6' OR '7' NOT CIRCLED (SKIP TO 230)	CODE '2', '4', '6' OR '7' CIRCLED<br>CODE '2', '4', 6' OR '7' NOT CIRCLED (SKIP TO 230)
222	**PURPLE TOP TUBE (EDTA)**<br>RECORD THE RESULT OF THE PURPLE TOP TUBE BLOOD SAMPLE COLLECTION	PURPLE TOP TUBE COLLECTED 1<br>INSUFFICIENT SAMPLE . . . . . . 2<br>REFUSED . . . . 3<br>OTHER 6	PURPLE TOP TUBE COLLECTED 1<br>INSUFFICIENT SAMPLE . . . . . . 2<br>REFUSED . . . . 3<br>OTHER 6	PURPLE TOP TUBE COLLECTED 1<br>INSUFFICIENT SAMPLE . . . . . . 2<br>REFUSED . . . . 3<br>OTHER 6
223	**BLUE TOP TUBE (METAL FREE)**<br>RECORD THE RESULT OF THE BLUE TOP TUBE BLOOD SAMPLE COLLECTION	BLUE TOP TUBE COLLECTED . 1<br>INSUFFICIENT SAMPLE . . . . . . 2<br>REFUSED . . . . 3<br>OTHER 6	BLUE TOP TUBE COLLECTED . 1<br>INSUFFICIENT SAMPLE . . . . . . 2<br>REFUSED . . . . 3<br>OTHER 6	BLUE TOP TUBE COLLECTED . 1<br>INSUFFICIENT SAMPLE . . . . . . 2<br>REFUSED . . . . 3<br>OTHER 6

200	CHECK COLUMN 7 IN HOUSEHOLD QUESTIONNAIRE. RECORD THE LINE NUMBER AND NAME FOR ALL CHILDREN 0-4 YEARS IN QUESTION 201; IF MORE THAN SIX CHILDREN, USE ADDITIONAL QUESTIONNAIRE BOOKLET AND USE THE DUPLICATE HH LABEL(S).			
		CHILD 1	CHILD 2	CHILD 3
201	CHECK HOUSEHOLD QUESTIONNAIRE:<br>LINE NUMBER FROM COLUMN 1.<br><br>NAME FROM COLUMN 2.	LINE NUMBER . . . .<br>NAME ____	LINE NUMBER . . . .<br>NAME ____	LINE NUMBER . . . .<br>NAME ____
224	DATE BLOOD SAMPLE TAKEN<br>(DAY/MONTH/YEAR)	DAY . . . .<br>MONTH . . . .<br>YEAR	DAY . . . .<br>MONTH . . . .<br>YEAR	DAY . . . .<br>MONTH . . . .<br>YEAR . . . .
225	TIME BLOOD DRAWN	HOURS . . . .<br>MINUTES	HOURS . . . .<br>MINUTES	HOURS . . . .<br>MINUTES . . . .
226	**DBS**<br>RECORD THE RESULT OF DBS SAMPLE COLLECTION	DBS SAMPLE COLLECTED .... 1<br>INSUFFICIENT SAMPLE . . . . . . 2<br>REFUSED . . . . 3<br>OTHER 6	DBS SAMPLE COLLECTED .... 1<br>INSUFFICIENT SAMPLE . . . . . . 2<br>REFUSED . . . . 3<br>OTHER 6	DBS SAMPLE COLLECTED .... 1<br>INSUFFICIENT SAMPLE . . . . . . 2<br>REFUSED . . . . 3<br>OTHER 6
227	RECORD MALARIA TEST RESULT	POSITIVE . . . . 1<br>NEGATIVE . . . . 2<br>INVALID . . . . 3<br>REFUSED . . . . 4<br>NOT PRESENT . . . . 5<br>OTHER 6	POSITIVE . . . . 1<br>NEGATIVE . . . . 2<br>INVALID . . . . 3<br>REFUSED . . . . 4<br>NOT PRESENT . . . . 5<br>OTHER 6	POSITIVE . . . . 1<br>NEGATIVE . . . . 2<br>INVALID . . . . 3<br>REFUSED . . . . 4<br>NOT PRESENT . . . . 5<br>OTHER 6
228	RECORD HEMOGLOBIN LEVEL HERE	G/DL . . . . . . __ __ . __<br>INSUFFICIENT SAMPLE . . . . 99.3<br>REFUSED . . . . 99.4<br>NOT PRESENT . . . . 99.5<br>OTHER 99.6	G/DL . . . . . . __ __ . __<br>INSUFFICIENT SAMPLE . . . . 99.3<br>REFUSED . . . . 99.4<br>NOT PRESENT . . . . 99.5<br>OTHER 99.6	G/DL . . . . . . __ __ . __<br>INSUFFICIENT SAMPLE . . . . 99.3<br>REFUSED . . . . 99.4<br>NOT PRESENT . . . . 99.5<br>OTHER 99.6
229	RECORD POC HEMOGLOBIN LEVEL HERE	VISUAL G/DL . . . . . . __ __ . __<br>APP G/DL . . . . . . __ __ . __<br>BLUE . . . . 99.3<br>GREEN . . . . 99.4<br>YELLOW . . . . 99.5<br>ORANGE . . . . 99.6<br>RED 99.7	VISUAL G/DL . . . . . . __ __ . __<br>APP G/DL . . . . . . __ __ . __<br>BLUE . . . . 99.3<br>GREEN . . . . 99.4<br>YELLOW . . . . 99.5<br>ORANGE . . . . 99.6<br>RED 99.7	VISUAL G/DL . . . . . . __ __ . __<br>APP G/DL . . . . . . __ __ . __<br>BLUE . . . . 99.3<br>GREEN . . . . 99.4<br>YELLOW . . . . 99.5<br>ORANGE . . . . 99.6<br>RED 99.7
230	CHECK 208:<br>AGREED FOR ANTROPOMETRIC MEASUREMENTS	CODE '1', '4', '5' OR '7' CIRCLED<br>CODE '1', '4', '5' OR '7' NOT CIRCLED (SKIP TO 236)	CODE '1', '4', '5' OR '7' CIRCLED<br>CODE '1', '4', '5' OR '7' NOT CIRCLED (SKIP TO 236)	CODE '1', '4', '5' OR '7' CIRCLED<br>CODE '1', '4', '5' OR '7' NOT CIRCLED (SKIP TO 236)
231	WEIGHT IN KILOGRAMS.	KG. . . . . __ __ . __ __<br>REFUSED . . . . 99.94<br>NOT PRESENT . . . . 99.95<br>OTHER 99.96	KG. . . . . __ __ . __ __<br>REFUSED . . . . 99.94<br>NOT PRESENT . . . . 99.95<br>OTHER 99.96	KG. . . . . __ __ . __ __<br>REFUSED . . . . 99.94<br>NOT PRESENT . . . . 99.95<br>OTHER 99.96
232	HEIGHT/LENGTH IN CENTIMETERS.	CM. . . . . __ __ __ . __<br>REFUSED . . . . 999.4<br>NOT PRESENT . . . . 999.5<br>OTHER . . . . 999.6<br>(SKIP TO 234)	CM. . . . . __ __ __ . __<br>REFUSED . . . . 999.4<br>NOT PRESENT . . . . 999.5<br>OTHER . . . . 999.6<br>(SKIP TO 234)	CM. . . . . __ __ __ . __<br>REFUSED . . . . 999.4<br>NOT PRESENT . . . . 999.5<br>OTHER . . . . 999.6<br>(SKIP TO 234)
233	MEASURED LYING DOWN OR STANDING UP?	LYING DOWN . . . . 1<br>STANDING UP 2	LYING DOWN . . . . 1<br>STANDING UP 2	LYING DOWN . . . . 1<br>STANDING UP 2
234	RECORD THE RESULT OF OEDEMA TESTING	HAS OEDEMA . . . . 1<br>NO OEDEMA 2	HAS OEDEMA . . . . 1<br>NO OEDEMA 2	HAS OEDEMA . . . . 1<br>NO OEDEMA 2
235	MID-UPPER ARM CIRCUMFERENCE (MUAC) IN CENTIMETERS.	CM . . . . __ __ . __ __<br>REFUSED . . . . 99.95<br>OTHER 99.96	CM . . . . __ __ . __ __<br>REFUSED . . . . 99.95<br>OTHER 99.96	CM . . . . __ __ . __ __<br>REFUSED . . . . 99.95<br>OTHER 99.96
236	LAB TECH: ENTER YOUR ID NUMBER.	ID NUMBER	ID NUMBER	ID NUMBER

200	CHECK COLUMN 7 IN HOUSEHOLD QUESTIONNAIRE. RECORD THE LINE NUMBER AND NAME FOR ALL CHILDREN 0-4 YEARS IN QUESTION 201; IF MORE THAN SIX CHILDREN, USE ADDITIONAL QUESTIONNAIRE BOOKLET AND USE THE DUPLICATE HH LABEL(S).			
		CHILD 1	CHILD 2	CHILD 3
201	CHECK HOUSEHOLD QUESTIONNAIRE: LINE NUMBER FROM COLUMN 1.<br>NAME FROM COLUMN 2.	LINE NUMBER . . . .<br>NAME ______	LINE NUMBER . . . .<br>NAME ______	LINE NUMBER . . . .<br>NAME ______
237	TIME BLOOD CENTRIFUGED	HOURS . . . .<br>MINUTES	HOURS . . . .<br>MINUTES	HOURS . . . .<br>MINUTES . . . .
238	CHECK 208:<br>AGREED FOR URINE TEST	CODE '3', '5', '6' OR '7' CIRCLED<br>CODE '3', '5', 6' OR '7' NOT CIRCLED (SKIP TO 243)	CODE '3', '5', '6' OR '7' CIRCLED<br>CODE '3', '5', 6' OR '7' NOT CIRCLED (SKIP TO 243)	CODE '3', '5', '6' OR '7' CIRCLED<br>CODE '3', '5', 6' OR '7' NOT CIRCLED (SKIP TO 243)
239	In order to determine if your child has blood in their urine, which might suggest that they have schistosomiasis, we would like to collect a urine sample from your child. If you can provide this now, we appreciate it. If not now, we can come back to pick up the sample at a later time.<br>INSTRUCTIONS IF UNABLE TO PRODUCE AT WILL:<br>FOR URINE: We will return tomorrow to pick up your child's urine. We would like the freshest urine you can give us. Please use this cup to collect your child's urine.			
240	**URINE SPECIMEN**<br>RECORD THE RESULT OF URINE SPECIMEN COLLECTION	URINE SPECIMEN COLLECTE ... 1<br>INSUFFICIENT SAMPLE . . . . . . 2<br>REFUSED . . . . 3<br>OTHER 6	URINE SPECIMEN COLLECTE ... 1<br>INSUFFICIENT SAMPLE . . . . . . 2<br>REFUSED . . . . 3<br>OTHER 6	URINE SPECIMEN COLLECTE ... 1<br>INSUFFICIENT SAMPLE . . . . . . 2<br>REFUSED . . . . 3<br>OTHER 6
241	DATE URINE SAMPLE COLLECTED (DAY/MONTH/YEAR)	DAY . . . .<br>MONTH . . . .<br>YEAR	DAY . . . .<br>MONTH . . . .<br>YEAR	DAY . . . .<br>MONTH . . . .<br>YEAR . . . .
242	RECORD RESULTS OF DIPSTICK FOR HEMATURIA	POSITIVE . . . . 1<br>NEGATIVE . . . . 2<br>INVALID . . . . 3<br>NOT PRESENT . . . . 4<br>OTHER 6	POSITIVE . . . . 1<br>NEGATIVE . . . . 2<br>INVALID . . . . 3<br>NOT PRESENT . . . . 4<br>OTHER 6	POSITIVE . . . . 1<br>NEGATIVE . . . . 2<br>INVALID . . . . 3<br>NOT PRESENT . . . . 4<br>OTHER 6
243	CHECK FRONT COVER<br>HOUSEHOLD SELECTED FOR MRDR TEST<br>HOUSEHOLD NOT SELECTED FOR MRDR TEST → 249			
244	CHECK 222:<br>WAS THE FIRST BLOOD SAMPLE COLLECTED?	YES<br>NO (SKIP TO 249)	YES<br>NO (SKIP TO 249)	YES<br>NO (SKIP TO 249)
245	As part of this survey we are asking some people to participate in an additional test. We would also like to include your child in an additional test to find out more information about vitamin A in the body. This test will involve giving your child a small amount of liquid to swallow with a snack. We will then have to wait about 4 hours and then take an additional small blood sample.<br>The results from this test will help the Ministry of Health understand better how well the food fortification program in Malawi is working and if other improvements are necessary.<br>Do you have any questions?<br>You can say yes or no. It is up to you to decide.<br>Will you allow (NAME OF CHILD) to participate in these tests?			
245A	CONSENT TO MRDR	CONSENT TO MRDR TEST GRANTED<br>CONSENT TO MRDR TEST NOT GRANTED (SKIP TO 249)	CONSENT TO MRDR TEST GRANTED<br>CONSENT TO MRDR TEST NOT GRANTED (SKIP TO 249)	CONSENT TO MRDR TEST GRANTED<br>CONSENT TO MRDR TEST NOT GRANTED (SKIP TO 249)
246	TIME OF INGESTING VITAMIN A2	HOURS . . . .<br>MINUTES	HOURS . . . .<br>MINUTES	HOURS . . . .<br>MINUTES . . . .
247	**MRDR TEST - BLOOD SAMPLE**<br>RECORD THE RESULT OF MRDR TEST BLOOD SAMPLE COLLECTION	MRDR TEST-SAMPLE COLLECTI 1<br>INSUFFICIENT SAMPLE . . . . . . 2<br>REFUSED . . . . 3<br>OTHER 6	MRDR TEST-SAMPLE COLLECTI 1<br>INSUFFICIENT SAMPLE . . . . . . 2<br>REFUSED . . . . 3<br>OTHER 6	MRDR TEST-SAMPLE COLLECTI 1<br>INSUFFICIENT SAMPLE . . . . . . 2<br>REFUSED . . . . 3<br>OTHER 6
248	TIME SECOND BLOOD DRAWN FOR MRDR TESTING	HOURS . . . .<br>MINUTES	HOURS . . . .<br>MINUTES	HOURS . . . .<br>MINUTES . . . .

200	CHECK COLUMN 7 IN HOUSEHOLD QUESTIONNAIRE. RECORD THE LINE NUMBER AND NAME FOR ALL CHILDREN 0-4 YEARS IN QUESTION 201; IF MORE THAN SIX CHILDREN, USE ADDITIONAL QUESTIONNAIRE BOOKLET AND USE THE DUPLICATE HH LABEL(S).			
		CHILD 1	CHILD 2	CHILD 3
201	CHECK HOUSEHOLD QUESTIONNAIRE:<br>LINE NUMBER FROM COLUMN 1.<br><br>NAME FROM COLUMN 2.	LINE NUMBER . . . . ☐☐<br><br>NAME ______	LINE NUMBER . . . . ☐☐<br><br>NAME ______	LINE NUMBER . . . . ☐☐<br><br>NAME ______
249	**REFERRAL CLINICAL MALARIA**<br><br>CHECK 227:<br>REFER IF RDT POSITIVE (227=1)	REFERRED . . . . 1<br>NOT REFERRED 2	REFERRED . . . . 1<br>NOT REFERRED 2	REFERRED . . . . 1<br>NOT REFERRED 2
250	**REFERRAL SEVERE ANEMIA**<br><br>CHECK 228:<br>REFER IF Hb <7 G/DL	REFERRED . . . . 1<br>NOT REFERRED 2	REFERRED . . . . 1<br>NOT REFERRED 2	REFERRED . . . . 1<br>NOT REFERRED 2
251	**REFERRAL MALNUTRITION**<br><br>CHECK 234 AND 235:<br>REFER IF OEDEMA PRESENT (234=1) AND/OR MUAC <11.5 CM	REFERRED . . . . 1<br>NOT REFERRED 2	REFERRED . . . . 1<br>NOT REFERRED 2	REFERRED . . . . 1<br>NOT REFERRED 2
252	**REFERRAL PRESUMED SHISTOSOMIASIS**<br><br>CHECK 242:<br>REFER IF HEMATURIA POSITIVE (242=1)	REFERRED . . . . 1<br>NOT REFERRED 2	REFERRED . . . . 1<br>NOT REFERRED 2	REFERRED . . . . 1<br>NOT REFERRED 2
253	GO BACK TO 202 IN NEXT COLUMN OR IN THE FIRST COLUMN OF THE NEXT PAGE OF THIS QUESTIONNAIRE;<br>IF NO MORE CHILDREN 0-4 YEARS, GO TO 300.			

200	CHECK COLUMN 7 IN HOUSEHOLD QUESTIONNAIRE. RECORD THE LINE NUMBER AND NAME FOR ALL CHILDREN 0-4 YEARS IN QUESTION 201; IF MORE THAN SIX CHILDREN, USE ADDITIONAL QUESTIONNAIRE BOOKLET AND USE THE DUPLICATE HH LABEL(S).			
		CHILD 4	CHILD 5	CHILD 6
201	CHECK HOUSEHOLD QUESTIONNAIRE:<br>LINE NUMBER FROM COLUMN 1.<br><br>NAME FROM COLUMN 2.	LINE NUMBER . . . .<br>NAME ________	LINE NUMBER . . . .<br>NAME ________	LINE NUMBER . . . .<br>NAME ________
202	What is (NAME)'s date of birth?	DAY . . . .<br>MONTH . . . .<br>YEAR	DAY . . . .<br>MONTH . . . .<br>YEAR	DAY . . . .<br>MONTH . . . .<br>YEAR . . . .
203	PRESCHOOL CHILD LABEL	PUT THE PRESCHOOL CHILD QUESTIONNAIRE BAR CODE LABEL HERE.	PUT THE PRESCHOOL CHILD QUESTIONNAIRE BAR CODE LABEL HERE.	PUT THE PRESCHOOL CHILD QUESTIONNAIRE BAR CODE LABEL HERE.
204	CHECK 202: CHILD BORN IN 2010-2015?	YES . . . . 1<br>NO . . . . 2<br>(SKIP TO 253)	YES . . . . 1<br>NO . . . . 2<br>(SKIP TO 253)	YES . . . . 1<br>NO . . . . 2<br>(SKIP TO 253)
205	CHECK 202: CHILD AGE 0-5 MONTHS, I.E., WAS CHILD BORN IN MONTH OF INTERVIEW OR 5 PREVIOUS MONTHS?	0-5 MONTHS . . . . 1<br>(SKIP TO 253)<br>6 MONTHS-4 YEARS 2	0-5 MONTHS . . . . 1<br>(SKIP TO 253)<br>6 MONTHS-4 YEARS 2	0-5 MONTHS . . . . 1<br>(SKIP TO 253)<br>6 MONTHS-4 YEARS 2
206	CHILD'S SEX	FEMALE . . . . 1<br>MALE 2	FEMALE . . . . 1<br>MALE 2	FEMALE . . . . 1<br>MALE 2
207	ASK CONSENT FOR ANTHROPOMETRY AND BIOLOGICAL TESTING<br>FROM PARENT/OTHER ADULT.	As part of this survey we are asking a parent of some children to allow us to weigh and measure their children and check them for Oedma. If your child has severe acute malnutrition we will refer your child to the nearest facility that can help you.<br>In addition to weighing and measuring your child we would like to take a sample of his/her blood and urine. The tests are safe. Some tests may cause your child slight discomfort, such as taking a blood sample. For all tests, there will be a brand new set of equipment used to take your child's blood and collect their urine, which is clean and completely safe. The equipment will be thrown away after it has been used on your child. With the blood we will test your child for anemia and malaria. Anemia is a serious health problem that usually results from poor nutrition, infection, or chronic disease. Malaria can also be serious and can lead to your child becoming anemic or making the anemia worse. You will be given these results immediately. If needed your child will be referred to a local health facility for treatment. The rest of the blood will be sent to a laboratory to be tested for other vitamins and minerals, such as vitamin A and iron. The results from these tests will not be reported back to you as it will take some time to process the blood.The results will be kept strictly confidential.<br>This information will help the Ministry of Health understand better what problems children in Malawi are experiencing and help them to improve the health and nutrition programs here, which will benefit all children in Malawi.<br><br>Do you have any questions?<br>You can say yes or no. It is up to you to decide.<br>Will you allow (NAME OF CHILD) to participate in these tests?		
208	CIRCLE THE CODE AND SIGN YOUR NAME.	AGREED, ANTHROPOM. MEASURES ONLY . . . . 1<br>AGREED, BLOOD TEST ONLY . 2<br>AGREED, URINE TEST ONLY . 3<br>AGREED, ANTHROPO& BLOOD TEST ONLY 4<br>AGREED, ANTHROPO& URINE TEST ONLY . . . . 5<br>AGREED, BLOOD& URINE TESTS ONLY . . . . 6<br>AGREED **ALL**, ANTHROPO & BLOOD&URINE TESTS . 7<br>REFUSED . . . . 8<br>________ (SIGN)<br>NOT PRESENT/OTHER . 9<br>(SKIP TO 253)	AGREED, ANTHROPOM. MEASURES ONLY . . . . 1<br>AGREED, BLOOD TEST ONLY . 2<br>AGREED, URINE TEST ONLY . 3<br>AGREED, ANTHROPO& BLOOD TEST ONLY 4<br>AGREED, ANTHROPO& URINE TEST ONLY . . . . 5<br>AGREED, BLOOD& URINE TESTS ONLY . . . . 6<br>AGREED **ALL**, ANTHROPO & BLOOD&URINE TESTS . 7<br>REFUSED . . . . 8<br>________ (SIGN)<br>NOT PRESENT/OTHER . 9<br>(SKIP TO 253)	AGREED, ANTHROPOM. MEASURES ONLY . . . . 1<br>AGREED, BLOOD TEST ONLY . 2<br>AGREED, URINE TEST ONLY . 3<br>AGREED, ANTHROPO& BLOOD TEST ONLY 4<br>AGREED, ANTHROPO& URINE TEST ONLY . . . . 5<br>AGREED, BLOOD& URINE TESTS ONLY . . . . 6<br>AGREED **ALL**, ANTHROPO & BLOOD&URINE TESTS . 7<br>REFUSED . . . . 8<br>________ (SIGN)<br>NOT PRESENT/OTHER . 9<br>(SKIP TO 253)
209	NURSE: ENTER YOUR ID NUMBER	ID NUMBER	ID NUMBER	ID NUMBER

200	CHECK COLUMN 7 IN HOUSEHOLD QUESTIONNAIRE. RECORD THE LINE NUMBER AND NAME FOR ALL CHILDREN 0-4 YEARS IN QUESTION 201; IF MORE THAN SIX CHILDREN, USE ADDITIONAL QUESTIONNAIRE BOOKLET AND USE THE DUPLICATE HH LABEL(S).			
		CHILD 4	CHILD 5	CHILD 6
201	CHECK HOUSEHOLD QUESTIONNAIRE:<br>LINE NUMBER FROM COLUMN 1.<br><br>NAME FROM COLUMN 2.	LINE NUMBER . . . . ☐☐<br><br>NAME ______	LINE NUMBER . . . . ☐☐<br><br>NAME ______	LINE NUMBER . . . . ☐☐<br><br>NAME ______
210A	In the last month, has (NAME OF CHILD) taken iron tablets/ syrups/ Multiple micronutrient powders?<br><br>SHOW COMMON IRON TABLETS/ SYRUP/ MNP IN MALAWI.	YES . . . . 1<br>NO 2	YES . . . . 1<br>NO 2	YES . . . . 1<br>NO 2
210	In the last six months, has (NAME OF CHILD) received deworming treatment?	YES . . . . 1<br>NO 2	YES . . . . 1<br>NO 2	YES . . . . 1<br>NO 2
211	In the last month, has (NAME OF CHILD) received any therapeutic foods, such as PLUMPY NUT [CHIPONDE]?<br><br>SHOW SACHET.	YES . . . . 1<br>NO 2	YES . . . . 1<br>NO 2	YES . . . . 1<br>NO 2
212	In the last month, has (NAME OF CHILD) received a vitamin A capsule?	YES . . . . 1<br>NO 2	YES . . . . 1<br>NO 2	YES . . . . 1<br>NO 2
213	Has (NAME OF CHILD) had a fever in the last 2 weeks?	YES . . . . 1<br>NO 2	YES . . . . 1<br>NO 2	YES . . . . 1<br>NO 2
214	Has (NAME OF CHILD) had a fever in the last 24 hours?	YES . . . . 1<br>NO 2	YES . . . . 1<br>NO 2	YES . . . . 1<br>NO 2
215	Has (NAME OF CHILD) had diarrhea in the last 2 weeks?	YES . . . . 1<br>NO 2	YES . . . . 1<br>NO 2	YES . . . . 1<br>NO 2
216	Has (NAME OF CHILD) had a cough or breathing problems in the last 2 weeks?	YES . . . . 1<br>NO 2	YES . . . . 1<br>NO 2	YES . . . . 1<br>NO 2
217	Has (NAME OF CHILD) been ill with malaria in the last 2 weeks?	YES . . . . 1<br>NO 2	YES . . . . 1<br>NO 2	YES . . . . 1<br>NO 2
218	Have you noticed blood in (NAME OF CHILD)'s urine in the past 2 weeks?	YES . . . . 1<br>NO 2	YES . . . . 1<br>NO 2	YES . . . . 1<br>NO 2
219	In the last six months, has (NAME OF CHILD) received a blood transfusion?	YES . . . . 1<br>NO 2	YES . . . . 1<br>NO 2	YES . . . . 1<br>NO 2
220	At what time approximately did (NAME OF CHILD) eat her/his most recent meal or was breastfed?	HOURS . . . . ☐☐<br>MINUTES . . . . ☐☐	HOURS . . . . ☐☐<br>MINUTES . . . . ☐☐	HOURS . . . . ☐☐<br>MINUTES . . . . ☐☐
221	CHECK 208:<br>AGREED FOR BLOOD TEST	CODE '2', '4', '6' OR '7' CIRCLED ☐<br>CODE '2', '4', 6' OR '7' NOT CIRCLED ☐ (SKIP TO 230)	CODE '2', '4', '6' OR '7' CIRCLED ☐<br>CODE '2', '4', 6' OR '7' NOT CIRCLED ☐ (SKIP TO 230)	CODE '2', '4', '6' OR '7' CIRCLED ☐<br>CODE '2', '4', 6' OR '7' NOT CIRCLED ☐ (SKIP TO 230)
222	**PURPLE TOP TUBE (EDTA)**<br>RECORD THE RESULT OF THE PURPLE TOP TUBE BLOOD SAMPLE COLLECTION	PURPLE TOP TUBE COLLECTED 1<br>INSUFFICIENT SAMPLE . . . . . . 2<br>REFUSED . . . . 3<br>OTHER 6	PURPLE TOP TUBE COLLECTED 1<br>INSUFFICIENT SAMPLE . . . . . . 2<br>REFUSED . . . . 3<br>OTHER 6	PURPLE TOP TUBE COLLECTED 1<br>INSUFFICIENT SAMPLE . . . . . . 2<br>REFUSED . . . . 3<br>OTHER 6
223	**BLUE TOP TUBE (METAL FREE)**<br>RECORD THE RESULT OF THE BLUE TOP TUBE BLOOD SAMPLE COLLECTION	BLUE TOP TUBE COLLECTED . 1<br>INSUFFICIENT SAMPLE . . . . . . 2<br>REFUSED . . . . 3<br>OTHER 6	BLUE TOP TUBE COLLECTED . 1<br>INSUFFICIENT SAMPLE . . . . . . 2<br>REFUSED . . . . 3<br>OTHER 6	BLUE TOP TUBE COLLECTED . 1<br>INSUFFICIENT SAMPLE . . . . . . 2<br>REFUSED . . . . 3<br>OTHER 6

BIOLOGICAL INFORMATION FOR CHILDREN AGE 6-59 MONTHS

200	CHECK COLUMN 7 IN HOUSEHOLD QUESTIONNAIRE. RECORD THE LINE NUMBER AND NAME FOR ALL CHILDREN 0-4 YEARS IN QUESTION 201; IF MORE THAN SIX CHILDREN, USE ADDITIONAL QUESTIONNAIRE BOOKLET AND USE THE DUPLICATE HH LABEL(S).			
		CHILD 4	CHILD 5	CHILD 6
201	CHECK HOUSEHOLD QUESTIONNAIRE:<br>LINE NUMBER FROM COLUMN 1.<br><br>NAME FROM COLUMN 2.	LINE NUMBER . . . . [ ][ ]<br>NAME ______	LINE NUMBER . . . . [ ][ ]<br>NAME ______	LINE NUMBER . . . . [ ][ ]<br>NAME ______
224	DATE BLOOD SAMPLE TAKEN<br>(DAY/MONTH/YEAR)	DAY . . . . [ ][ ]<br>MONTH . . . . [ ][ ]<br>YEAR . . . . [ ][ ][ ][ ]	DAY . . . . [ ][ ]<br>MONTH . . . . [ ][ ]<br>YEAR . . . . [ ][ ][ ][ ]	DAY . . . . [ ][ ]<br>MONTH . . . . [ ][ ]<br>YEAR . . . . [ ][ ][ ][ ]
225	TIME BLOOD DRAWN	HOURS . . . . [ ][ ]<br>MINUTES . . . . [ ][ ]	HOURS . . . . [ ][ ]<br>MINUTES . . . . [ ][ ]	HOURS . . . . [ ][ ]<br>MINUTES . . . . [ ][ ]
226	**DBS**<br>RECORD THE RESULT OF DBS SAMPLE COLLECTION	DBS SAMPLE COLLECTED .... 1<br>INSUFFICIENT SAMPLE . . . . . . 2<br>REFUSED . . . . 3<br>OTHER 6	DBS SAMPLE COLLECTED .... 1<br>INSUFFICIENT SAMPLE . . . . . . 2<br>REFUSED . . . . 3<br>OTHER 6	DBS SAMPLE COLLECTED .... 1<br>INSUFFICIENT SAMPLE . . . . . . 2<br>REFUSED . . . . 3<br>OTHER 6
227	RECORD MALARIA TEST RESULT	POSITIVE . . . . 1<br>NEGATIVE . . . . 2<br>INVALID . . . . 3<br>REFUSED . . . . 4<br>NOT PRESENT . . . . 5<br>OTHER 6	POSITIVE . . . . 1<br>NEGATIVE . . . . 2<br>INVALID . . . . 3<br>REFUSED . . . . 4<br>NOT PRESENT . . . . 5<br>OTHER 6	POSITIVE . . . . 1<br>NEGATIVE . . . . 2<br>INVALID . . . . 3<br>REFUSED . . . . 4<br>NOT PRESENT . . . . 5<br>OTHER 6
228	RECORD HEMOGLOBIN LEVEL HERE	G/DL . . . . . . [ ][ ].[ ]<br>INSUFFICIENT SAMPLE . . . . 99.3<br>REFUSED . . . . 99.4<br>NOT PRESENT . . . . 99.5<br>OTHER 99.6	G/DL . . . . . . [ ][ ].[ ]<br>INSUFFICIENT SAMPLE . . . . 99.3<br>REFUSED . . . . 99.4<br>NOT PRESENT . . . . 99.5<br>OTHER 99.6	G/DL . . . . . . [ ][ ].[ ]<br>INSUFFICIENT SAMPLE . . . . 99.3<br>REFUSED . . . . 99.4<br>NOT PRESENT . . . . 99.5<br>OTHER 99.6
229	RECORD POC HEMOGLOBIN LEVEL HERE	VISUAL<br>G/DL . . . . . . [ ][ ].[ ]<br>APP<br>G/DL . . . . . . [ ][ ].[ ]<br>BLUE . . . . 99.3<br>GREEN . . . . 99.4<br>YELLOW . . . . 99.5<br>ORANGE . . . . 99.6<br>RED 99.7	VISUAL<br>G/DL . . . . . . [ ][ ].[ ]<br>APP<br>G/DL . . . . . . [ ][ ].[ ]<br>BLUE . . . . 99.3<br>GREEN . . . . 99.4<br>YELLOW . . . . 99.5<br>ORANGE . . . . 99.6<br>RED 99.7	VISUAL<br>G/DL . . . . . . [ ][ ].[ ]<br>APP<br>G/DL . . . . . . [ ][ ].[ ]<br>BLUE . . . . 99.3<br>GREEN . . . . 99.4<br>YELLOW . . . . 99.5<br>ORANGE . . . . 99.6<br>RED 99.7
230	CHECK 208:<br>AGREED FOR ANTROPOMETRIC MEASUREMENTS	CODE '1', '4', '5' OR '7' CIRCLED [ ] ↓<br>CODE '1', '4', '5' OR '7' NOT CIRCLED [ ] (SKIP TO 236)	CODE '1', '4', '5' OR '7' CIRCLED [ ] ↓<br>CODE '1', '4', '5' OR '7' NOT CIRCLED [ ] (SKIP TO 236)	CODE '1', '4', '5' OR '7' CIRCLED [ ] ↓<br>CODE '1', '4', '5' OR '7' NOT CIRCLED [ ] (SKIP TO 236)
231	WEIGHT IN KILOGRAMS.	KG. . . . . [ ][ ].[ ][ ]<br>REFUSED . . . . 99.94<br>NOT PRESENT . . . . 99.95<br>OTHER 99.96	KG. . . . . [ ][ ].[ ][ ]<br>REFUSED . . . . 99.94<br>NOT PRESENT . . . . 99.95<br>OTHER 99.96	KG. . . . . [ ][ ].[ ][ ]<br>REFUSED . . . . 99.94<br>NOT PRESENT . . . . 99.95<br>OTHER 99.96
232	HEIGHT/LENGTH IN CENTIMETERS.	CM. . . . . [ ][ ][ ].[ ]<br>REFUSED . . . . 999.4<br>NOT PRESENT . . . . 999.5<br>OTHER . . . . 999.6<br>(SKIP TO 234)	CM. . . . . [ ][ ][ ].[ ]<br>REFUSED . . . . 999.4<br>NOT PRESENT . . . . 999.5<br>OTHER . . . . 999.6<br>(SKIP TO 234)	CM. . . . . [ ][ ][ ].[ ]<br>REFUSED . . . . 999.4<br>NOT PRESENT . . . . 999.5<br>OTHER . . . . 999.6<br>(SKIP TO 234)
233	MEASURED LYING DOWN OR STANDING UP?	LYING DOWN . . . . 1<br>STANDING UP 2	LYING DOWN . . . . 1<br>STANDING UP 2	LYING DOWN . . . . 1<br>STANDING UP 2
234	RECORD THE RESULT OF OEDEMA TESTING	HAS OEDEMA . . . . 1<br>NO OEDEMA 2	HAS OEDEMA . . . . 1<br>NO OEDEMA 2	HAS OEDEMA . . . . 1<br>NO OEDEMA 2
235	MID-UPPER ARM CIRCUMFERENCE (MUAC) IN CENTIMETERS.	CM . . . . [ ][ ].[ ][ ]<br>REFUSED . . . . 99.95<br>OTHER 99.96	CM . . . . [ ][ ].[ ][ ]<br>REFUSED . . . . 99.95<br>OTHER 99.96	CM . . . . [ ][ ].[ ][ ]<br>REFUSED . . . . 99.95<br>OTHER 99.96
236	LAB TECH: ENTER YOUR ID NUMBER.	[ ][ ]<br>ID NUMBER	[ ][ ]<br>ID NUMBER	[ ][ ]<br>ID NUMBER

200	CHECK COLUMN 7 IN HOUSEHOLD QUESTIONNAIRE. RECORD THE LINE NUMBER AND NAME FOR ALL CHILDREN 0-4 YEARS IN QUESTION 201; IF MORE THAN SIX CHILDREN, USE ADDITIONAL QUESTIONNAIRE BOOKLET AND USE THE DUPLICATE HH LABEL(S).			
		CHILD 4	CHILD 5	CHILD 6
201	CHECK HOUSEHOLD QUESTIONNAIRE:<br>LINE NUMBER FROM COLUMN 1.<br><br>NAME FROM COLUMN 2.	LINE NUMBER . . . . [ ][ ]<br><br>NAME ________	LINE NUMBER . . . . [ ][ ]<br><br>NAME ________	LINE NUMBER . . . . [ ][ ]<br><br>NAME ________
237	TIME BLOOD CENTRIFUGED	HOURS . . . . [ ][ ]<br>MINUTES . . . . [ ][ ]	HOURS . . . . [ ][ ]<br>MINUTES . . . . [ ][ ]	HOURS . . . . [ ][ ]<br>MINUTES . . . . [ ][ ]
238	CHECK 208:<br>AGREED FOR URINE TEST	CODE '3', '5', '6' OR '7' CIRCLED [ ]<br>CODE '3', '5', 6' OR '7' NOT CIRCLED [ ] (SKIP TO 243)	CODE '3', '5', '6' OR '7' CIRCLED [ ]<br>CODE '3', '5', 6' OR '7' NOT CIRCLED [ ] (SKIP TO 243)	CODE '3', '5', '6' OR '7' CIRCLED [ ]<br>CODE '3', '5', 6' OR '7' NOT CIRCLED [ ] (SKIP TO 243)
239	In order to determine if your child has blood in their urine, which might suggest that they have schistosomiasis, we would like to collect a urine sample from your child. If you can provide this now, we appreciate it. If not now, we can come back to pick up the sample at a later time.<br><br>INSTRUCTIONS IF UNABLE TO PRODUCE AT WILL:<br><br>FOR URINE: We will return tomorrow to pick up your child's urine. We would like the freshest urine you can give us. Please use this cup to collect your child's urine.			
240	**URINE SPECIMEN**<br>RECORD THE RESULT OF URINE SPECIMEN COLLECTION	URINE SPECIMEN COLLECTE ... 1<br>INSUFFICIENT SAMPLE . . . . . . 2<br>REFUSED . . . . 3<br>OTHER 6	URINE SPECIMEN COLLECTE ... 1<br>INSUFFICIENT SAMPLE . . . . . . 2<br>REFUSED . . . . 3<br>OTHER 6	URINE SPECIMEN COLLECTE ... 1<br>INSUFFICIENT SAMPLE . . . . . . 2<br>REFUSED . . . . 3<br>OTHER 6
241	DATE URINE SAMPLE COLLECTED<br>(DAY/MONTH/YEAR)	DAY . . . . [ ][ ]<br>MONTH . . . . [ ][ ]<br>YEAR . . . . [ ][ ][ ][ ]	DAY . . . . [ ][ ]<br>MONTH . . . . [ ][ ]<br>YEAR . . . . [ ][ ][ ][ ]	DAY . . . . [ ][ ]<br>MONTH . . . . [ ][ ]<br>YEAR . . . . [ ][ ][ ][ ]
242	RECORD RESULTS OF DIPSTICK FOR HEMATURIA	POSITIVE . . . . 1<br>NEGATIVE . . . . 2<br>INVALID . . . . 3<br>NOT PRESENT . . . . 4<br>OTHER 6	POSITIVE . . . . 1<br>NEGATIVE . . . . 2<br>INVALID . . . . 3<br>NOT PRESENT . . . . 4<br>OTHER 6	POSITIVE . . . . 1<br>NEGATIVE . . . . 2<br>INVALID . . . . 3<br>NOT PRESENT . . . . 4<br>OTHER 6
243	CHECK FRONT COVER<br>HOUSEHOLD SELECTED FOR MRDR TEST [ ]<br>HOUSEHOLD NOT SELECTED FOR MRDR TEST [ ] → 249			
244	CHECK 222:<br>WAS THE FIRST BLOOD SAMPLE COLLECTED?	YES [ ]<br>NO [ ] (SKIP TO 249)	YES [ ]<br>NO [ ] (SKIP TO 249)	YES [ ]<br>NO [ ] (SKIP TO 249)
245	As part of this survey we are asking some people to participate in an additional test. We would also like to include your child in an additional test to find out more information about vitamin A in the body. This test will involve giving your child a small amount of liquid to swallow with a snack. We will then have to wait about 4 hours and then take an additional small blood sample.<br>The results from this test will help the Ministry of Health understand better how well the food fortification program in Malawi is working and if other improvements are necessary.<br><br>Do you have any questions?<br>You can say yes or no. It is up to you to decide.<br>Will you allow (NAME OF CHILD) to participate in these tests?			
245A	CONSENT TO MRDR	CONSENT TO MRDR TEST GRANTED [ ]<br>CONSENT TO MRDR TEST NOT GRANTED [ ] (SKIP TO 249)	CONSENT TO MRDR TEST GRANTED [ ]<br>CONSENT TO MRDR TEST NOT GRANTED [ ] (SKIP TO 249)	CONSENT TO MRDR TEST GRANTED [ ]<br>CONSENT TO MRDR TEST NOT GRANTED [ ] (SKIP TO 249)
246	TIME OF INGESTING VITAMIN A2	HOURS . . . . [ ][ ]<br>MINUTES . . . . . . [ ][ ]	HOURS . . . . [ ][ ]<br>MINUTES . . . . [ ][ ]	HOURS . . . . [ ][ ]<br>MINUTES . . . . [ ][ ]
247	**MRDR TEST - BLOOD SAMPLE**<br>RECORD THE RESULT OF MRDR TEST BLOOD SAMPLE COLLECTION	MRDR TEST-SAMPLE COLLECTI 1<br>INSUFFICIENT SAMPLE . . . . . . 2<br>REFUSED . . . . 3<br>OTHER 6	MRDR TEST-SAMPLE COLLECTI 1<br>INSUFFICIENT SAMPLE . . . . . . 2<br>REFUSED . . . . 3<br>OTHER 6	MRDR TEST-SAMPLE COLLECTI 1<br>INSUFFICIENT SAMPLE . . . . . . 2<br>REFUSED . . . . 3<br>OTHER 6
248	TIME SECOND BLOOD DRAWN FOR MRDR TESTING	HOURS . . . . [ ][ ]<br>MINUTES . . . . [ ][ ]	HOURS . . . . [ ][ ]<br>MINUTES . . . . [ ][ ]	HOURS . . . . [ ][ ]<br>MINUTES . . . . [ ][ ]

200	CHECK COLUMN 7 IN HOUSEHOLD QUESTIONNAIRE. RECORD THE LINE NUMBER AND NAME FOR ALL CHILDREN 0-4 YEARS IN QUESTION 201; IF MORE THAN SIX CHILDREN, USE ADDITIONAL QUESTIONNAIRE BOOKLET AND USE THE DUPLICATE HH LABEL(S).			
		CHILD 4	CHILD 5	CHILD 6
201	CHECK HOUSEHOLD QUESTIONNAIRE:<br>LINE NUMBER FROM COLUMN 1.<br><br>NAME FROM COLUMN 2.	LINE NUMBER . . . . ☐☐<br><br>NAME ________	LINE NUMBER . . . . ☐☐<br><br>NAME ________	LINE NUMBER . . . . ☐☐<br><br>NAME ________
249	**REFERRAL CLINICAL MALARIA**<br><br>CHECK 227:<br>REFER IF RDT POSITIVE (227=1)	REFERRED . . . . 1<br>NOT REFERRED 2	REFERRED . . . . 1<br>NOT REFERRED 2	REFERRED . . . . 1<br>NOT REFERRED 2
250	**REFERRAL SEVERE ANEMIA**<br><br>CHECK 228:<br>REFER IF Hb <7 G/DL	REFERRED . . . . 1<br>NOT REFERRED 2	REFERRED . . . . 1<br>NOT REFERRED 2	REFERRED . . . . 1<br>NOT REFERRED 2
251	**REFERRAL MALNUTRITION**<br><br>CHECK 234 AND 235:<br>REFER IF OEDEMA PRESENT (234=1) AND/OR MUAC <11.5 CM	REFERRED . . . . 1<br>NOT REFERRED 2	REFERRED . . . . 1<br>NOT REFERRED 2	REFERRED . . . . 1<br>NOT REFERRED 2
252	**REFERRAL PRESUMED SHISTOSOMIASIS**<br><br>CHECK 242:<br>REFER IF HEMATURIA POSITIVE (242=1)	REFERRED . . . . 1<br>NOT REFERRED 2	REFERRED . . . . 1<br>NOT REFERRED 2	REFERRED . . . . 1<br>NOT REFERRED 2
253	GO BACK TO 202 IN NEXT COLUMN OR IN THE FIRST COLUMN OF THE NEXT PAGE OF THIS QUESTIONNAIRE;<br>IF NO MORE CHILDREN 0-4 YEARS, GO TO 300.			

BIOLOGICAL INFORMATION FOR CHILDREN AGE 5-14 YEARS

300	CHECK COLUMN 7 IN HOUSEHOLD QUESTIONNAIRE. RECORD THE LINE NUMBER AND NAME FOR ALL CHILDREN 5-14 YEARS IN QUESTION 301; IF MORE THAN SIX CHILDREN, USE ADDITIONAL QUESTIONNAIRE BOOKLET AND USE THE DUPLICATE HH LABEL(S).			
		CHILD 1	CHILD 2	CHILD 3
301	CHECK HOUSEHOLD QUESTIONNAIRE:<br>LINE NUMBER FROM COLUMN 1.<br><br>NAME FROM COLUMN 2.	LINE NUMBER . . . .<br><br>NAME ____________	LINE NUMBER . . . .<br><br>NAME ____________	LINE NUMBER . . . .<br><br>NAME ____________
302	What is (NAME)'s date of birth?	DAY . . . .<br>MONTH . . . .<br>YEAR	DAY . . . .<br>MONTH . . . .<br>YEAR	DAY . . . .<br>MONTH . . . .<br>YEAR . . . .
303	SCHOOL-AGED CHILD LABEL	PUT THE SCHOOL AGED CHILD QUESTIONNAIRE BAR CODE LABEL HERE.	PUT THE SCHOOL AGED CHILD QUESTIONNAIRE BAR CODE LABEL HERE.	PUT THE SCHOOL AGED CHILD QUESTIONNAIRE BAR CODE LABEL HERE.
304	CHECK 302: CHILD BORN IN 2000-2010	YES . . . . 1<br>NO . . . . 2<br>(SKIP TO 351)	YES . . . . 1<br>NO . . . . 2<br>(SKIP TO 351)	YES . . . . 1<br>NO . . . . 2<br>(SKIP TO 351)
305	CHILD'S SEX	FEMALE . . . . 1<br>MALE 2	FEMALE . . . . 1<br>MALE 2	FEMALE . . . . 1<br>MALE 2
306	ASK CONSENT FOR ANTHROPOMETRY AND BIOLOGICAL TESTING FROM PARENT/OTHER ADULT.	As part of this survey we are asking a parent of some children to allow us to weigh and measure their children and check them for Oedma. If your child has severe acute malnutrition we will refer your child to the nearest facility that can help you.<br>In addition to weighing and measuring your child we would like to take a sample of his/her blood and urine. The tests are safe. Some tests may cause your child slight discomfort, such as taking a blood sample. For all tests, there will be a brand new set of equipment used to take your child's blood and collect their urine, which is clean and completely safe. The equipment will be thrown away after it has been used on your child.<br>With the blood we will test your child for anemia and malaria. Anemia is a serious health problem that usually results from poor nutrition, infection, or chronic disease. Malaria can also be serious and can lead to your child becoming anemic or making the anemia worse. You will be given these results immediately. If needed your child will be referred to a local health facility for treatment. The rest of the blood will be sent to a laboratory to be tested for other vitamins and minerals, such as vitamin A and iron. The results from these tests will not be reported back to you as it will take some time to process the blood.The results will be kept strictly confidential and will not be shared with anyone other than members of our survey team.<br>This information will help the Ministry of Health understand better what problems children in Malawi are experiencing and help them to improve the health and nutrition programs here, which will benefit all children in Malawi.<br><br>Do you have any questions?<br>You can say yes or no. It is up to you to decide.<br>Will you allow (NAME OF CHILD) to participate in these tests?		
307	CIRCLE THE CODE AND SIGN YOUR NAME.	AGREED, ANTHROPOM. MEASURES ONLY . . . . 1<br>AGREED, BLOOD TEST ONLY . 2<br>AGREED, URINE TEST ONLY . 3<br>AGREED, ANTHROPO& BLOOD TEST ONLY 4<br>AGREED, ANTHROPO& URINE TEST ONLY . . . . 5<br>AGREED, BLOOD& URINE TESTS ONLY . . . . 6<br>AGREED **ALL**, ANTHROPO & BLOOD&URINE TESTS . 7<br>REFUSED . . . . 8<br>____________ (SIGN)<br>NOT PRESENT/OTHER . 9<br>(SKIP TO 351)	AGREED, ANTHROPOM. MEASURES ONLY . . . . 1<br>AGREED, BLOOD TEST ONLY . 2<br>AGREED, URINE TEST ONLY . 3<br>AGREED, ANTHROPO& BLOOD TEST ONLY 4<br>AGREED, ANTHROPO& URINE TEST ONLY . . . . 5<br>AGREED, BLOOD& URINE TESTS ONLY . . . . 6<br>AGREED **ALL**, ANTHROPO & BLOOD&URINE TESTS . 7<br>REFUSED . . . . 8<br>____________ (SIGN)<br>NOT PRESENT/OTHER . 9<br>(SKIP TO 351)	AGREED, ANTHROPOM. MEASURES ONLY . . . . 1<br>AGREED, BLOOD TEST ONLY . 2<br>AGREED, URINE TEST ONLY . 3<br>AGREED, ANTHROPO& BLOOD TEST ONLY 4<br>AGREED, ANTHROPO& URINE TEST ONLY . . . . 5<br>AGREED, BLOOD& URINE TESTS ONLY . . . . 6<br>AGREED **ALL**, ANTHROPO & BLOOD&URINE TESTS . 7<br>REFUSED . . . . 8<br>____________ (SIGN)<br>NOT PRESENT/OTHER . 9<br>(SKIP TO 351)
308	NURSE: ENTER YOUR ID NUMBER	ID NUMBER	ID NUMBER	ID NUMBER

300	CHECK COLUMN 7 IN HOUSEHOLD QUESTIONNAIRE. RECORD THE LINE NUMBER AND NAME FOR ALL CHILDREN 5-14 YEARS IN QUESTION 301; IF MORE THAN SIX CHILDREN, USE ADDITIONAL QUESTIONNAIRE BOOKLET AND USE THE DUPLICATE HH LABEL(S).			
		CHILD 1	CHILD 2	CHILD 3
301	CHECK HOUSEHOLD QUESTIONNAIRE:<br>LINE NUMBER FROM COLUMN 1.<br><br>NAME FROM COLUMN 2.	LINE NUMBER . . . .<br>NAME	LINE NUMBER . . . .<br>NAME	LINE NUMBER . . . .<br>NAME
309A	In the last month, has (NAME OF CHILD) taken iron tablets or syrups?<br><br>SHOW COMMON IRON TABLETS IN MALAWI.	YES . . . . 1<br>NO 2	YES . . . . 1<br>NO 2	YES . . . . 1<br>NO 2
309	In the last six months, has (NAME OF CHILD) received deworming treatment?	YES . . . . 1<br>NO 2	YES . . . . 1<br>NO 2	YES . . . . 1<br>NO 2
310	In the last month, has (NAME OF CHILD) received any therapeutic foods, such as PLUMPY NUT [CHIPONDE]?<br><br>SHOW SACHET.	YES . . . . 1<br>NO 2	YES . . . . 1<br>NO 2	YES . . . . 1<br>NO 2
311	In the last month, has (NAME OF CHILD) received a vitamin A capsule?	YES . . . . 1<br>NO 2	YES . . . . 1<br>NO 2	YES . . . . 1<br>NO 2
312	Has (NAME OF CHILD) had a fever in the last 2 weeks?	YES . . . . 1<br>NO 2	YES . . . . 1<br>NO 2	YES . . . . 1<br>NO 2
313	Has (NAME OF CHILD) had a fever in the last 24 hours?	YES . . . . 1<br>NO 2	YES . . . . 1<br>NO 2	YES . . . . 1<br>NO 2
314	Has (NAME OF CHILD) had diarrhea in the last 2 weeks?	YES . . . . 1<br>NO 2	YES . . . . 1<br>NO 2	YES . . . . 1<br>NO 2
315	Has (NAME OF CHILD) had a cough or breathing problems in the last 2 weeks?	YES . . . . 1<br>NO 2	YES . . . . 1<br>NO 2	YES . . . . 1<br>NO 2
316	Has (NAME OF CHILD) been ill with malaria in the last 2 weeks?	YES . . . . 1<br>NO 2	YES . . . . 1<br>NO 2	YES . . . . 1<br>NO 2
317	Have you noticed blood in (NAME OF CHILD)'s urine in the past 2 weeks?	YES . . . . 1<br>NO 2	YES . . . . 1<br>NO 2	YES . . . . 1<br>NO 2
318	In the last six months, has (NAME OF CHILD) received a blood transfusion?	YES . . . . 1<br>NO 2	YES . . . . 1<br>NO 2	YES . . . . 1<br>NO 2
319	At what time approximately did (NAME OF CHILD) eat her/his most recent meal?	HOURS . . . .<br>MINUTES	HOURS . . . .<br>MINUTES	HOURS . . . .<br>MINUTES . . . .
320	CHECK 307:<br>AGREED FOR BLOOD TEST	CODE '2', '4', '6' OR '7' CIRCLED<br>CODE '2', '4', 6' OR '7' NOT CIRCLED (SKIP TO 328)	CODE '2', '4', '6' OR '7' CIRCLED<br>CODE '2', '4', 6' OR '7' NOT CIRCLED (SKIP TO 328)	CODE '2', '4', '6' OR '7' CIRCLED<br>CODE '2', '4', 6' OR '7' NOT CIRCLED (SKIP TO 328)
321	**PURPLE TOP TUBE (EDTA)**<br>RECORD THE RESULT OF THE PURPLE TOP TUBE BLOOD SAMPLE COLLECTION	PURPLE TOP TUBE COLLECTED 1<br>INSUFFICIENT SAMPLE . . . . . . 2<br>REFUSED . . . . 3<br>OTHER 6	PURPLE TOP TUBE COLLECTED 1<br>INSUFFICIENT SAMPLE . . . . . . 2<br>REFUSED . . . . 3<br>OTHER 6	PURPLE TOP TUBE COLLECTED 1<br>INSUFFICIENT SAMPLE . . . . . . 2<br>REFUSED . . . . 3<br>OTHER 6
322	**BLUE TOP TUBE (METAL FREE)**<br>RECORD THE RESULT OF THE BLUE TOP TUBE BLOOD SAMPLE COLLECTION	BLUE TOP TUBE COLLECTED . 1<br>INSUFFICIENT SAMPLE . . . . . . 2<br>REFUSED . . . . 3<br>OTHER 6	BLUE TOP TUBE COLLECTED . 1<br>INSUFFICIENT SAMPLE . . . . . . 2<br>REFUSED . . . . 3<br>OTHER 6	BLUE TOP TUBE COLLECTED . 1<br>INSUFFICIENT SAMPLE . . . . . . 2<br>REFUSED . . . . 3<br>OTHER 6

BIOLOGICAL INFORMATION FOR CHILDREN AGE 5-14 YEARS

300	CHECK COLUMN 7 IN HOUSEHOLD QUESTIONNAIRE. RECORD THE LINE NUMBER AND NAME FOR ALL CHILDREN 5-14 YEARS IN QUESTION 301; IF MORE THAN SIX CHILDREN, USE ADDITIONAL QUESTIONNAIRE BOOKLET AND USE THE DUPLICATE HH LABEL(S).			
		CHILD 1	CHILD 2	CHILD 3
301	CHECK HOUSEHOLD QUESTIONNAIRE:<br>LINE NUMBER FROM COLUMN 1.<br><br>NAME FROM COLUMN 2.	LINE NUMBER . . . . [ ][ ]<br>NAME ________	LINE NUMBER . . . . [ ][ ]<br>NAME ________	LINE NUMBER . . . . [ ][ ]<br>NAME ________
323	DATE BLOOD SAMPLE TAKEN (DAY/MONTH/YEAR)	DAY . . . . [ ][ ]<br>MONTH . . . . [ ][ ]<br>YEAR . . . . [ ][ ][ ][ ]	DAY . . . . [ ][ ]<br>MONTH . . . . [ ][ ]<br>YEAR . . . . [ ][ ][ ][ ]	DAY . . . . [ ][ ]<br>MONTH . . . . [ ][ ]<br>YEAR . . . . [ ][ ][ ][ ]
324	TIME BLOOD DRAWN	HOURS . . . . [ ][ ]<br>MINUTES . . . . [ ][ ]	HOURS . . . . [ ][ ]<br>MINUTES . . . . [ ][ ]	HOURS . . . . [ ][ ]<br>MINUTES . . . . [ ][ ]
325	RECORD MALARIA TEST RESULT	POSITIVE . . . . 1<br>NEGATIVE . . . . 2<br>INVALID . . . . 3<br>REFUSED . . . . 4<br>NOT PRESENT . . . . 5<br>OTHER 6	POSITIVE . . . . 1<br>NEGATIVE . . . . 2<br>INVALID . . . . 3<br>REFUSED . . . . 4<br>NOT PRESENT . . . . 5<br>OTHER 6	POSITIVE . . . . 1<br>NEGATIVE . . . . 2<br>INVALID . . . . 3<br>REFUSED . . . . 4<br>NOT PRESENT . . . . 5<br>OTHER 6
326	RECORD HEMOGLOBIN LEVEL HERE	G/DL . . . . . . [ ][ ].[ ]<br>INSUFFICIENT SAMPLE . . . . 99.3<br>REFUSED . . . . 99.4<br>NOT PRESENT . . . . 99.5<br>OTHER 99.6	G/DL . . . . . . [ ][ ].[ ]<br>INSUFFICIENT SAMPLE . . . . 99.3<br>REFUSED . . . . 99.4<br>NOT PRESENT . . . . 99.5<br>OTHER 99.6	G/DL . . . . . . [ ][ ].[ ]<br>INSUFFICIENT SAMPLE . . . . 99.3<br>REFUSED . . . . 99.4<br>NOT PRESENT . . . . 99.5<br>OTHER 99.6
327	RECORD POC HEMOGLOBIN LEVEL HERE	VISUAL G/DL . . . . . . [ ][ ].[ ]<br>APP G/DL . . . . . . [ ][ ].[ ]<br>BLUE . . . . 99.3<br>GREEN . . . . 99.4<br>YELLOW . . . . 99.5<br>ORANGE . . . . 99.6<br>RED 99.7	VISUAL G/DL . . . . . . [ ][ ].[ ]<br>APP G/DL . . . . . . [ ][ ].[ ]<br>BLUE . . . . 99.3<br>GREEN . . . . 99.4<br>YELLOW . . . . 99.5<br>ORANGE . . . . 99.6<br>RED 99.7	VISUAL G/DL . . . . . . [ ][ ].[ ]<br>APP G/DL . . . . . . [ ][ ].[ ]<br>BLUE . . . . 99.3<br>GREEN . . . . 99.4<br>YELLOW . . . . 99.5<br>ORANGE . . . . 99.6<br>RED 99.7
328	CHECK 307:<br>AGREED FOR ANTROPOMETRIC MEASUREMENTS	CODE '1', '4', '5' OR '7' CIRCLED [ ]<br>CODE '1', '4', '5' OR '7' NOT CIRCLED [ ]<br>(SKIP TO 334)	CODE '1', '4', '5' OR '7' CIRCLED [ ]<br>CODE '1', '4', '5' OR '7' NOT CIRCLED [ ]<br>(SKIP TO 334)	CODE '1', '4', '5' OR '7' CIRCLED [ ]<br>CODE '1', '4', '5' OR '7' NOT CIRCLED [ ]<br>(SKIP TO 334)
329	WEIGHT IN KILOGRAMS.	KG. . . . . [ ][ ].[ ][ ]<br>REFUSED . . . . 99.94<br>NOT PRESENT . . . . 99.95<br>OTHER 99.96	KG. . . . . [ ][ ].[ ][ ]<br>REFUSED . . . . 99.94<br>NOT PRESENT . . . . 99.95<br>OTHER 99.96	KG. . . . . [ ][ ].[ ][ ]<br>REFUSED . . . . 99.94<br>NOT PRESENT . . . . 99.95<br>OTHER 99.96
330	HEIGHT/LENGTH IN CENTIMETERS.	CM. . . . . [ ][ ][ ].[ ]<br>REFUSED . . . . 999.4<br>NOT PRESENT . . . . 999.5<br>OTHER 999.6	CM. . . . . [ ][ ][ ].[ ]<br>REFUSED . . . . 999.4<br>NOT PRESENT . . . . 999.5<br>OTHER 999.6	CM. . . . . [ ][ ][ ].[ ]<br>REFUSED . . . . 999.4<br>NOT PRESENT . . . . 999.5<br>OTHER 999.6
333	MID-UPPER ARM CIRCUMFERENCE (MUAC) IN CENTIMETERS.	CM . . . . [ ][ ].[ ][ ]<br>REFUSED . . . . 99.95<br>OTHER 99.96	CM . . . . [ ][ ].[ ][ ]<br>REFUSED . . . . 99.95<br>OTHER 99.96	CM . . . . [ ][ ].[ ][ ]<br>REFUSED . . . . 99.95<br>OTHER 99.96
334	LAB TECH: ENTER YOUR ID NUMBER.	[ ][ ]<br>ID NUMBER	[ ][ ]<br>ID NUMBER	[ ][ ]<br>ID NUMBER

BIOLOGICAL INFORMATION FOR CHILDREN AGE 5-14 YEARS

300	CHECK COLUMN 7 IN HOUSEHOLD QUESTIONNAIRE. RECORD THE LINE NUMBER AND NAME FOR ALL CHILDREN 5-14 YEARS IN QUESTION 301; IF MORE THAN SIX CHILDREN, USE ADDITIONAL QUESTIONNAIRE BOOKLET AND USE THE DUPLICATE HH LABEL(S).			
		CHILD 1	CHILD 2	CHILD 3
301	CHECK HOUSEHOLD QUESTIONNAIRE:<br>LINE NUMBER FROM COLUMN 1.<br><br>NAME FROM COLUMN 2.	LINE NUMBER . . . . ☐☐<br><br>NAME ______	LINE NUMBER . . . . ☐☐<br><br>NAME ______	LINE NUMBER . . . . ☐☐<br><br>NAME ______
335	TIME BLOOD CENTRIFUGED	HOURS . . . . ☐☐<br>MINUTES . . . . ☐☐	HOURS . . . . ☐☐<br>MINUTES . . . . ☐☐	HOURS . . . . ☐☐<br>MINUTES . . . . ☐☐
336	CHECK 307:<br>AGREED FOR URINE TEST	CODE '3', '5', '6' OR '7' CIRCLED ☐<br>CODE '3', '5', 6' OR '7' NOT CIRCLED ☐ (SKIP TO 341)	CODE '3', '5', '6' OR '7' CIRCLED ☐<br>CODE '3', '5', 6' OR '7' NOT CIRCLED ☐ (SKIP TO 341)	CODE '3', '5', '6' OR '7' CIRCLED ☐<br>CODE '3', '5', 6' OR '7' NOT CIRCLED ☐ (SKIP TO 341)
337	In order to determine if your child has blood in their urine, which might suggest that they have schistosomiasis, we would like to collect a urine sample from your child. If you can provide this now, we appreciate it. If not now, we can come back to pick up the sample at a later time.<br><br>INSTRUCTIONS IF UNABLE TO PRODUCE AT WILL:<br><br>FOR URINE: We will return tomorrow to pick up your child's urine. We would like the freshest urine you can give us. Please use this cup to collect your child's urine .			
338	**URINE SPECIMEN**<br>RECORD THE RESULT OF URINE SPECIMEN COLLECTION	URINE SPECIMEN COLLECTE ... 1<br>INSUFFICIENT SAMPLE . . . . . . 2<br>REFUSED . . . . 3<br>OTHER 6	URINE SPECIMEN COLLECTE ... 1<br>INSUFFICIENT SAMPLE . . . . . . 2<br>REFUSED . . . . 3<br>OTHER 6	URINE SPECIMEN COLLECTE ... 1<br>INSUFFICIENT SAMPLE . . . . . . 2<br>REFUSED . . . . 3<br>OTHER 6
339	DATE URINE SAMPLE COLLECTED (DAY/MONTH/YEAR)	DAY . . . . ☐☐<br>MONTH . . . . ☐☐<br>YEAR . . . . ☐☐☐☐	DAY . . . . ☐☐<br>MONTH . . . . ☐☐<br>YEAR . . . . ☐☐☐☐	DAY . . . . ☐☐<br>MONTH . . . . ☐☐<br>YEAR . . . . ☐☐☐☐
340	RECORD RESULTS OF DIPSTICK FOR HEMATURIA	POSITIVE . . . . 1<br>NEGATIVE . . . . 2<br>INVALID . . . . 3<br>REFUSED . . . . 4<br>NOT PRESENT . . . . 5<br>OTHER 6	POSITIVE . . . . 1<br>NEGATIVE . . . . 2<br>INVALID . . . . 3<br>REFUSED . . . . 4<br>NOT PRESENT . . . . 5<br>OTHER 6	POSITIVE . . . . 1<br>NEGATIVE . . . . 2<br>INVALID . . . . 3<br>REFUSED . . . . 4<br>NOT PRESENT . . . . 5<br>OTHER 6
341	CHECK FRONT COVER<br>HOUSEHOLD SELECTED FOR MRDR TEST ☐<br>HOUSEHOLD NOT SELECTED FOR MRDR TEST ☐ → 347			
342	CHECK 322:<br>WAS THE FIRST BLOOD SAMPLE COLLECTED?	YES ☐<br>NO ☐ (SKIP TO 347)	YES ☐<br>NO ☐ (SKIP TO 347)	YES ☐<br>NO ☐ (SKIP TO 347)
343	As part of this survey we are asking some people to participate in an additional test. We would also like to include your child in an additional test to find out more information about vitamin A in the body. This test will involve giving your child a small amount of liquid to swallow with a snack. We will then have to wait about 4 hours and then take an additional small blood sample. The results from this test will help the Ministry of Health understand better how well the food fortification program in Malawi is working and if other improvements are necessary.<br><br>Do you have any questions?<br>You can say yes or no. It is up to you to decide.<br>Will you allow (NAME OF CHILD) to participate in these tests?			
343A	CONSENT TO MRDR	CONSENT TO MRDR TEST GRANTED ☐<br>CONSENT TO MRDR TEST NOT GRANTED ☐ (SKIP TO 347)	CONSENT TO MRDR TEST GRANTED ☐<br>CONSENT TO MRDR TEST NOT GRANTED ☐ (SKIP TO 347)	CONSENT TO MRDR TEST GRANTED ☐<br>CONSENT TO MRDR TEST NOT GRANTED ☐ (SKIP TO 347)
344	TIME OF INGESTING VITAMIN A2	HOURS . . . . ☐☐<br>MINUTES . . . . . . ☐☐	HOURS . . . . ☐☐<br>MINUTES . . . . ☐☐	HOURS . . . . ☐☐<br>MINUTES . . . . ☐☐
345	**MRDR TEST - BLOOD SAMPLE**<br>RECORD THE RESULT OF MRDR TEST BLOOD SAMPLE COLLECTION	MRDR TEST-SAMPLE COLLECTI 1<br>INSUFFICIENT SAMPLE . . . . . . 2<br>REFUSED . . . . 3<br>OTHER 6	MRDR TEST-SAMPLE COLLECTI 1<br>INSUFFICIENT SAMPLE . . . . . . 2<br>REFUSED . . . . 3<br>OTHER 6	MRDR TEST-SAMPLE COLLECTI 1<br>INSUFFICIENT SAMPLE . . . . . . 2<br>REFUSED . . . . 3<br>OTHER 6
346	TIME SECOND BLOOD DRAWN FOR MRDR TESTING	HOURS . . . . ☐☐<br>MINUTES . . . . ☐☐	HOURS . . . . ☐☐<br>MINUTES . . . . ☐☐	HOURS . . . . ☐☐<br>MINUTES . . . . ☐☐

300	CHECK COLUMN 7 IN HOUSEHOLD QUESTIONNAIRE. RECORD THE LINE NUMBER AND NAME FOR ALL CHILDREN 5-14 YEARS IN QUESTION 301; IF MORE THAN SIX CHILDREN, USE ADDITIONAL QUESTIONNAIRE BOOKLET AND USE THE DUPLICATE HH LABEL(S).			
		CHILD 1	CHILD 2	CHILD 3
301	CHECK HOUSEHOLD QUESTIONNAIRE:<br>LINE NUMBER FROM COLUMN 1.<br><br>NAME FROM COLUMN 2.	LINE NUMBER . . . . [ ][ ]<br><br>NAME ________	LINE NUMBER . . . . [ ][ ]<br><br>NAME ________	LINE NUMBER . . . . [ ][ ]<br><br>NAME ________
347	**REFERRAL CLINICAL MALARIA**<br><br>CHECK 325:<br>REFER IF RDT POSITIVE (325=1)	REFERRED . . . . 1<br>NOT REFERRED 2	REFERRED . . . . 1<br>NOT REFERRED 2	REFERRED . . . . 1<br>NOT REFERRED 2
348	**REFERRAL SEVERE ANEMIA**<br><br>CHECK 326:<br>REFER IF Hb <7 G/DL	REFERRED . . . . 1<br>NOT REFERRED 2	REFERRED . . . . 1<br>NOT REFERRED 2	REFERRED . . . . 1<br>NOT REFERRED 2
349	**REFERRAL MALNUTRITION**<br><br>CHECK 333:<br>REFER IF MUAC <14.0 CM	REFERRED . . . . 1<br>NOT REFERRED 2	REFERRED . . . . 1<br>NOT REFERRED 2	REFERRED . . . . 1<br>NOT REFERRED 2
350	**REFERRAL PRESUMED SHISTOSOMIASIS**<br><br>CHECK 340:<br>REFER IF HEMATURIA POSITIVE (340=1)	REFERRED . . . . 1<br>NOT REFERRED 2	REFERRED . . . . 1<br>NOT REFERRED 2	REFERRED . . . . 1<br>NOT REFERRED 2
351	GO BACK TO 302 IN NEXT COLUMN OR IN THE FIRST COLUMN OF THE NEXT PAGE OF THIS QUESTIONNAIRE;<br>IF NO MORE CHILDREN 5-14 YEARS, GO TO 300.			

300	CHECK COLUMN 7 IN HOUSEHOLD QUESTIONNAIRE. RECORD THE LINE NUMBER AND NAME FOR ALL CHILDREN 5-14 YEARS IN QUESTION 301; IF MORE THAN SIX CHILDREN, USE ADDITIONAL QUESTIONNAIRE BOOKLET AND USE THE DUPLICATE HH LABEL(S).			
		CHILD 4	CHILD 5	CHILD 6
301	CHECK HOUSEHOLD QUESTIONNAIRE: LINE NUMBER FROM COLUMN 1.<br>NAME FROM COLUMN 2.	LINE NUMBER . . . .<br>NAME ______	LINE NUMBER . . . .<br>NAME ______	LINE NUMBER . . . .<br>NAME ______
302	What is (NAME)'s date of birth?	DAY . . . .<br>MONTH . . . .<br>YEAR	DAY . . . .<br>MONTH . . . .<br>YEAR	DAY . . . .<br>MONTH . . . .<br>YEAR . . . .
303	SCHOOL-AGED CHILD LABEL	PUT THE SCHOOL AGED CHILD QUESTIONNAIRE BAR CODE LABEL HERE.	PUT THE SCHOOL AGED CHILD QUESTIONNAIRE BAR CODE LABEL HERE.	PUT THE SCHOOL AGED CHILD QUESTIONNAIRE BAR CODE LABEL HERE.
304	CHECK 302: CHILD BORN IN 2000-2010	YES . . . . 1<br>NO . . . . 2<br>(SKIP TO 351)	YES . . . . 1<br>NO . . . . 2<br>(SKIP TO 351)	YES . . . . 1<br>NO . . . . 2<br>(SKIP TO 351)
305	CHILD'S SEX	FEMALE . . . . 1<br>MALE 2	FEMALE . . . . 1<br>MALE 2	FEMALE . . . . 1<br>MALE 2
306	ASK CONSENT FOR ANTHROPOMETRY AND BIOLOGICAL TESTING FROM PARENT/OTHER ADULT.	As part of this survey we are asking a parent of some children to allow us to weigh and measure their children and check them for Oedma. If your child has severe acute malnutrition we will refer your child to the nearest facility that can help you.<br>In addition to weighing and measuring your child we would like to take a sample of his/her blood and urine. The tests are safe. Some tests may cause your child slight discomfort, such as taking a blood sample. For all tests, there will be a brand new set of equipment used to take your child's blood and collect their urine, which is clean and completely safe. The equipment will be thrown away after it has been used on your child.<br>With the blood we will test your child for anemia and malaria. Anemia is a serious health problem that usually results from poor nutrition, infection, or chronic disease. Malaria can also be serious and can lead to your child becoming anemic or making the anemia worse. You will be given these results immediately. If needed your child will be referred to a local health facility for treatment. The rest of the blood will be sent to a laboratory to be tested for other vitamins and minerals, such as vitamin A and iron. The results from these tests will not be reported back to you as it will take some time to process the blood.The results will be kept strictly confidential and will not be shared with anyone other than members of our survey team.<br>This information will help the Ministry of Health understand better what problems children in Malawi are experiencing and help them to improve the health and nutrition programs here, which will benefit all children in Malawi.<br><br>Do you have any questions?<br>You can say yes or no. It is up to you to decide.<br>Will you allow (NAME OF CHILD) to participate in these tests?		
307	CIRCLE THE CODE AND SIGN YOUR NAME.	AGREED, ANTHROPOM. MEASURES ONLY . . . . 1<br>AGREED, BLOOD TEST ONLY . 2<br>AGREED, URINE TEST ONLY . 3<br>AGREED, ANTHROPO& BLOOD TEST ONLY 4<br>AGREED, ANTHROPO& URINE TEST ONLY . . . . 5<br>AGREED, BLOOD& URINE TESTS ONLY . . . . 6<br>AGREED **ALL**, ANTHROPO & BLOOD&URINE TESTS . 7<br>REFUSED . . . . 8<br>______ (SIGN)<br>NOT PRESENT/OTHER . 9<br>(SKIP TO 351)	AGREED, ANTHROPOM. MEASURES ONLY . . . . 1<br>AGREED, BLOOD TEST ONLY . 2<br>AGREED, URINE TEST ONLY . 3<br>AGREED, ANTHROPO& BLOOD TEST ONLY 4<br>AGREED, ANTHROPO& URINE TEST ONLY . . . . 5<br>AGREED, BLOOD& URINE TESTS ONLY . . . . 6<br>AGREED **ALL**, ANTHROPO & BLOOD&URINE TESTS . 7<br>REFUSED . . . . 8<br>______ (SIGN)<br>NOT PRESENT/OTHER . 9<br>(SKIP TO 351)	AGREED, ANTHROPOM. MEASURES ONLY . . . . 1<br>AGREED, BLOOD TEST ONLY . 2<br>AGREED, URINE TEST ONLY . 3<br>AGREED, ANTHROPO& BLOOD TEST ONLY 4<br>AGREED, ANTHROPO& URINE TEST ONLY . . . . 5<br>AGREED, BLOOD& URINE TESTS ONLY . . . . 6<br>AGREED **ALL**, ANTHROPO & BLOOD&URINE TESTS . 7<br>REFUSED . . . . 8<br>______ (SIGN)<br>NOT PRESENT/OTHER . 9<br>(SKIP TO 351)
308	NURSE: ENTER YOUR ID NUMBER	ID NUMBER	ID NUMBER	ID NUMBER

300	CHECK COLUMN 7 IN HOUSEHOLD QUESTIONNAIRE. RECORD THE LINE NUMBER AND NAME FOR ALL CHILDREN 5-14 YEARS IN QUESTION 301; IF MORE THAN SIX CHILDREN, USE ADDITIONAL QUESTIONNAIRE BOOKLET AND USE THE DUPLICATE HH LABEL(S).			
		CHILD 4	CHILD 5	CHILD 6
301	CHECK HOUSEHOLD QUESTIONNAIRE:<br>LINE NUMBER FROM COLUMN 1.<br><br>NAME FROM COLUMN 2.	LINE NUMBER . . . .<br><br>NAME ______	LINE NUMBER . . . .<br><br>NAME ______	LINE NUMBER . . . .<br><br>NAME ______
309A	In the last month, has (NAME OF CHILD) taken iron tablets or syrups?<br><br>SHOW COMMON IRON TABLETS IN MALAWI.	YES . . . . 1<br>NO 2	YES . . . . 1<br>NO 2	YES . . . . 1<br>NO 2
309	In the last six months, has (NAME OF CHILD) received deworming treatment?	YES . . . . 1<br>NO 2	YES . . . . 1<br>NO 2	YES . . . . 1<br>NO 2
310	In the last month, has (NAME OF CHILD) received any therapeutic foods, such as PLUMPY NUT [CHIPONDE]?<br><br>SHOW SACHET.	YES . . . . 1<br>NO 2	YES . . . . 1<br>NO 2	YES . . . . 1<br>NO 2
311	In the last month, has (NAME OF CHILD) received a vitamin A capsule?	YES . . . . 1<br>NO 2	YES . . . . 1<br>NO 2	YES . . . . 1<br>NO 2
312	Has (NAME OF CHILD) had a fever in the last 2 weeks?	YES . . . . 1<br>NO 2	YES . . . . 1<br>NO 2	YES . . . . 1<br>NO 2
313	Has (NAME OF CHILD) had a fever in the last 24 hours?	YES . . . . 1<br>NO 2	YES . . . . 1<br>NO 2	YES . . . . 1<br>NO 2
314	Has (NAME OF CHILD) had diarrhea in the last 2 weeks?	YES . . . . 1<br>NO 2	YES . . . . 1<br>NO 2	YES . . . . 1<br>NO 2
315	Has (NAME OF CHILD) had a cough or breathing problems in the last 2 weeks?	YES . . . . 1<br>NO 2	YES . . . . 1<br>NO 2	YES . . . . 1<br>NO 2
316	Has (NAME OF CHILD) been ill with malaria in the last 2 weeks?	YES . . . . 1<br>NO 2	YES . . . . 1<br>NO 2	YES . . . . 1<br>NO 2
317	Have you noticed blood in (NAME OF CHILD)'s urine in the past 2 weeks?	YES . . . . 1<br>NO 2	YES . . . . 1<br>NO 2	YES . . . . 1<br>NO 2
318	In the last six months, has (NAME OF CHILD) received a blood transfusion?	YES . . . . 1<br>NO 2	YES . . . . 1<br>NO 2	YES . . . . 1<br>NO 2
319	At what time approximately did (NAME OF CHILD) eat her/his most recent meal?	HOURS . . . .<br>MINUTES	HOURS . . . .<br>MINUTES	HOURS . . . .<br>MINUTES . . . .
320	CHECK 307:<br>AGREED FOR BLOOD TEST	CODE '2', '4', '6' OR '7' CIRCLED<br>CODE '2', '4', 6' OR '7' NOT CIRCLED (SKIP TO 328)	CODE '2', '4', '6' OR '7' CIRCLED<br>CODE '2', '4', 6' OR '7' NOT CIRCLED (SKIP TO 328)	CODE '2', '4', '6' OR '7' CIRCLED<br>CODE '2', '4', 6' OR '7' NOT CIRCLED (SKIP TO 328)
321	**PURPLE TOP TUBE (EDTA)**<br>RECORD THE RESULT OF THE PURPLE TOP TUBE BLOOD SAMPLE COLLECTION	PURPLE TOP TUBE COLLECTED 1<br>INSUFFICIENT SAMPLE . . . . . . 2<br>REFUSED . . . . 3<br>OTHER 6	PURPLE TOP TUBE COLLECTED 1<br>INSUFFICIENT SAMPLE . . . . . . 2<br>REFUSED . . . . 3<br>OTHER 6	PURPLE TOP TUBE COLLECTED 1<br>INSUFFICIENT SAMPLE . . . . . . 2<br>REFUSED . . . . 3<br>OTHER 6
322	**BLUE TOP TUBE (METAL FREE)**<br>RECORD THE RESULT OF THE BLUE TOP TUBE BLOOD SAMPLE COLLECTION	BLUE TOP TUBE COLLECTED . 1<br>INSUFFICIENT SAMPLE . . . . . . 2<br>REFUSED . . . . 3<br>OTHER 6	BLUE TOP TUBE COLLECTED . 1<br>INSUFFICIENT SAMPLE . . . . . . 2<br>REFUSED . . . . 3<br>OTHER 6	BLUE TOP TUBE COLLECTED . 1<br>INSUFFICIENT SAMPLE . . . . . . 2<br>REFUSED . . . . 3<br>OTHER 6

300	CHECK COLUMN 7 IN HOUSEHOLD QUESTIONNAIRE. RECORD THE LINE NUMBER AND NAME FOR ALL CHILDREN 5-14 YEARS IN QUESTION 301; IF MORE THAN SIX CHILDREN, USE ADDITIONAL QUESTIONNAIRE BOOKLET AND USE THE DUPLICATE HH LABEL(S).			
		CHILD 4	CHILD 5	CHILD 6
301	CHECK HOUSEHOLD QUESTIONNAIRE:<br>LINE NUMBER FROM COLUMN 1.<br><br>NAME FROM COLUMN 2.	LINE NUMBER . . . .<br>NAME ________	LINE NUMBER . . . .<br>NAME ________	LINE NUMBER . . . .<br>NAME ________
323	DATE BLOOD SAMPLE TAKEN<br>(DAY/MONTH/YEAR)	DAY . . . .<br>MONTH . . . .<br>YEAR	DAY . . . .<br>MONTH . . . .<br>YEAR	DAY . . . .<br>MONTH . . . .<br>YEAR . . . .
324	TIME BLOOD DRAWN	HOURS . . . .<br>MINUTES	HOURS . . . .<br>MINUTES	HOURS . . . .<br>MINUTES . . . .
325	RECORD MALARIA TEST RESULT	POSITIVE . . . . 1<br>NEGATIVE . . . . 2<br>INVALID . . . . 3<br>REFUSED . . . . 4<br>NOT PRESENT . . . . 5<br>OTHER 6	POSITIVE . . . . 1<br>NEGATIVE . . . . 2<br>INVALID . . . . 3<br>REFUSED . . . . 4<br>NOT PRESENT . . . . 5<br>OTHER 6	POSITIVE . . . . 1<br>NEGATIVE . . . . 2<br>INVALID . . . . 3<br>REFUSED . . . . 4<br>NOT PRESENT . . . . 5<br>OTHER 6
326	RECORD HEMOGLOBIN LEVEL HERE	G/DL . . . . . . __ __ . __<br>INSUFFICIENT SAMPLE . . . . 99.3<br>REFUSED . . . . 99.4<br>NOT PRESENT . . . . 99.5<br>OTHER 99.6	G/DL . . . . . . __ __ . __<br>INSUFFICIENT SAMPLE . . . . 99.3<br>REFUSED . . . . 99.4<br>NOT PRESENT . . . . 99.5<br>OTHER 99.6	G/DL . . . . . . __ __ . __<br>INSUFFICIENT SAMPLE . . . . 99.3<br>REFUSED . . . . 99.4<br>NOT PRESENT . . . . 99.5<br>OTHER 99.6
327	RECORD POC HEMOGLOBIN LEVEL HERE	VISUAL G/DL . . . . . . __ __ . __<br>APP G/DL . . . . . . __ __ . __<br>BLUE . . . . 99.3<br>GREEN . . . . 99.4<br>YELLOW . . . . 99.5<br>ORANGE . . . . 99.6<br>RED 99.7	VISUAL G/DL . . . . . . __ __ . __<br>APP G/DL . . . . . . __ __ . __<br>BLUE . . . . 99.3<br>GREEN . . . . 99.4<br>YELLOW . . . . 99.5<br>ORANGE . . . . 99.6<br>RED 99.7	VISUAL G/DL . . . . . . __ __ . __<br>APP G/DL . . . . . . __ __ . __<br>BLUE . . . . 99.3<br>GREEN . . . . 99.4<br>YELLOW . . . . 99.5<br>ORANGE . . . . 99.6<br>RED 99.7
328	CHECK 307:<br>AGREED FOR ANTROPOMETRIC MEASUREMENTS	CODE '1', '4', '5' OR '7' CIRCLED<br>CODE '1', '4', '5' OR '7' NOT CIRCLED (SKIP TO 334)	CODE '1', '4', '5' OR '7' CIRCLED<br>CODE '1', '4', '5' OR '7' NOT CIRCLED (SKIP TO 334)	CODE '1', '4', '5' OR '7' CIRCLED<br>CODE '1', '4', '5' OR '7' NOT CIRCLED (SKIP TO 334)
329	WEIGHT IN KILOGRAMS.	KG. . . . . __ __ . __ __<br>REFUSED . . . . 99.94<br>NOT PRESENT . . . . 99.95<br>OTHER 99.96	KG. . . . . __ __ . __ __<br>REFUSED . . . . 99.94<br>NOT PRESENT . . . . 99.95<br>OTHER 99.96	KG. . . . . __ __ . __ __<br>REFUSED . . . . 99.94<br>NOT PRESENT . . . . 99.95<br>OTHER 99.96
330	HEIGHT/LENGTH IN CENTIMETERS.	CM. . . . . __ __ __ . __<br>REFUSED . . . . 999.4<br>NOT PRESENT . . . . 999.5<br>OTHER 999.6	CM. . . . . __ __ __ . __<br>REFUSED . . . . 999.4<br>NOT PRESENT . . . . 999.5<br>OTHER 999.6	CM. . . . . __ __ __ . __<br>REFUSED . . . . 999.4<br>NOT PRESENT . . . . 999.5<br>OTHER 999.6
333	MID-UPPER ARM CIRCUMFERENCE (MUAC) IN CENTIMETERS.	CM . . . . __ __ . __ __<br>REFUSED . . . . 99.95<br>OTHER 99.96	CM . . . . __ __ . __ __<br>REFUSED . . . . 99.95<br>OTHER 99.96	CM . . . . __ __ . __ __<br>REFUSED . . . . 99.95<br>OTHER 99.96
334	LAB TECH: ENTER YOUR ID NUMBER.	ID NUMBER	ID NUMBER	ID NUMBER

300	CHECK COLUMN 7 IN HOUSEHOLD QUESTIONNAIRE. RECORD THE LINE NUMBER AND NAME FOR ALL CHILDREN 5-14 YEARS IN QUESTION 301; IF MORE THAN SIX CHILDREN, USE ADDITIONAL QUESTIONNAIRE BOOKLET AND USE THE DUPLICATE HH LABEL(S).			
		CHILD 4	CHILD 5	CHILD 6
301	CHECK HOUSEHOLD QUESTIONNAIRE:<br>LINE NUMBER FROM COLUMN 1.<br><br>NAME FROM COLUMN 2.	LINE NUMBER . . . .<br>NAME ______	LINE NUMBER . . . .<br>NAME ______	LINE NUMBER . . . .<br>NAME ______
335	TIME BLOOD CENTRIFUGED	HOURS . . . .<br>MINUTES	HOURS . . . .<br>MINUTES	HOURS . . . .<br>MINUTES . . . .
336	CHECK 307:<br>AGREED FOR URINE TEST	CODE '3', '5', '6' OR '7' CIRCLED<br>CODE '3', '5', 6' OR '7' NOT CIRCLED (SKIP TO 341)	CODE '3', '5', '6' OR '7' CIRCLED<br>CODE '3', '5', 6' OR '7' NOT CIRCLED (SKIP TO 341)	CODE '3', '5', '6' OR '7' CIRCLED<br>CODE '3', '5', 6' OR '7' NOT CIRCLED (SKIP TO 341)
337	In order to determine if your child has blood in their urine, which might suggest that they have schistosomiasis, we would like to collect a urine sample from your child. If you can provide this now, we appreciate it. If not now, we can come back to pick up the sample at a later time.<br><br>INSTRUCTIONS IF UNABLE TO PRODUCE AT WILL:<br><br>FOR URINE: We will return tomorrow to pick up your child's urine. We would like the freshest urine you can give us. Please use this cup to collect your child's urine .			
338	**URINE SPECIMEN**<br>RECORD THE RESULT OF URINE SPECIMEN COLLECTION	URINE SPECIMEN COLLECTE ... 1<br>INSUFFICIENT SAMPLE . . . . . . 2<br>REFUSED . . . . 3<br>OTHER 6	URINE SPECIMEN COLLECTE ... 1<br>INSUFFICIENT SAMPLE . . . . . . 2<br>REFUSED . . . . 3<br>OTHER 6	URINE SPECIMEN COLLECTE ... 1<br>INSUFFICIENT SAMPLE . . . . . . 2<br>REFUSED . . . . 3<br>OTHER 6
339	DATE URINE SAMPLE COLLECTED<br>(DAY/MONTH/YEAR)	DAY . . . .<br>MONTH . . . .<br>YEAR	DAY . . . .<br>MONTH . . . .<br>YEAR	DAY . . . .<br>MONTH . . . .<br>YEAR . . . .
340	RECORD RESULTS OF DIPSTICK FOR HEMATURIA	POSITIVE . . . . 1<br>NEGATIVE . . . . 2<br>INVALID . . . . 3<br>REFUSED . . . . 4<br>NOT PRESENT . . . . 5<br>OTHER 6	POSITIVE . . . . 1<br>NEGATIVE . . . . 2<br>INVALID . . . . 3<br>REFUSED . . . . 4<br>NOT PRESENT . . . . 5<br>OTHER 6	POSITIVE . . . . 1<br>NEGATIVE . . . . 2<br>INVALID . . . . 3<br>REFUSED . . . . 4<br>NOT PRESENT . . . . 5<br>OTHER 6
341	CHECK FRONT COVER<br>HOUSEHOLD SELECTED FOR MRDR TEST<br>HOUSEHOLD NOT SELECTED FOR MRDR TEST → 347			
342	CHECK 322:<br>WAS THE FIRST BLOOD SAMPLE COLLECTED?	YES<br>NO (SKIP TO 347)	YES<br>NO (SKIP TO 347)	YES<br>NO (SKIP TO 347)
343	As part of this survey we are asking some people to participate in an additional test. We would also like to include your child in an additional test to find out more information about vitamin A in the body. This test will involve giving your child a small amount of liquid to swallow with a snack. We will then have to wait about 4 hours and then take an additional small blood sample.<br>The results from this test will help the Ministry of Health understand better how well the food fortification program in Malawi is working and if other improvements are necessary.<br><br>Do you have any questions?<br>You can say yes or no. It is up to you to decide.<br>Will you allow (NAME OF CHILD) to participate in these tests?			
343A	CONSENT TO MRDR	CONSENT TO MRDR TEST GRANTED<br>CONSENT TO MRDR TEST NOT GRANTED (SKIP TO 347)	CONSENT TO MRDR TEST GRANTED<br>CONSENT TO MRDR TEST NOT GRANTED (SKIP TO 347)	CONSENT TO MRDR TEST GRANTED<br>CONSENT TO MRDR TEST NOT GRANTED (SKIP TO 347)
344	TIME OF INGESTING VITAMIN A2	HOURS . . . .<br>MINUTES	HOURS . . . .<br>MINUTES	HOURS . . . .<br>MINUTES . . . .
345	**MRDR TEST - BLOOD SAMPLE**<br>RECORD THE RESULT OF MRDR TEST BLOOD SAMPLE COLLECTION	MRDR TEST-SAMPLE COLLECTI 1<br>INSUFFICIENT SAMPLE . . . . . . 2<br>REFUSED . . . . 3<br>OTHER 6	MRDR TEST-SAMPLE COLLECTI 1<br>INSUFFICIENT SAMPLE . . . . . . 2<br>REFUSED . . . . 3<br>OTHER 6	MRDR TEST-SAMPLE COLLECTI 1<br>INSUFFICIENT SAMPLE . . . . . . 2<br>REFUSED . . . . 3<br>OTHER 6
346	TIME SECOND BLOOD DRAWN FOR MRDR TESTING	HOURS . . . .<br>MINUTES	HOURS . . . .<br>MINUTES	HOURS . . . .<br>MINUTES . . . .

300	CHECK COLUMN 7 IN HOUSEHOLD QUESTIONNAIRE. RECORD THE LINE NUMBER AND NAME FOR ALL CHILDREN 5-14 YEARS IN QUESTION 301; IF MORE THAN SIX CHILDREN, USE ADDITIONAL QUESTIONNAIRE BOOKLET AND USE THE DUPLICATE HH LABEL(S).			
		CHILD 4	CHILD 5	CHILD 6
301	CHECK HOUSEHOLD QUESTIONNAIRE:<br>LINE NUMBER FROM COLUMN 1.<br><br>NAME FROM COLUMN 2.	LINE NUMBER . . . . [ ][ ]<br><br>NAME ____________	LINE NUMBER . . . . [ ][ ]<br><br>NAME ____________	LINE NUMBER . . . . [ ][ ]<br><br>NAME ____________
347	**REFERRAL CLINICAL MALARIA**<br><br>CHECK 325:<br>REFER IF RDT POSITIVE (325=1)	REFERRED . . . . 1<br>NOT REFERRED 2	REFERRED . . . . 1<br>NOT REFERRED 2	REFERRED . . . . 1<br>NOT REFERRED 2
348	**REFERRAL SEVERE ANEMIA**<br><br>CHECK 326:<br>REFER IF Hb <7 G/DL	REFERRED . . . . 1<br>NOT REFERRED 2	REFERRED . . . . 1<br>NOT REFERRED 2	REFERRED . . . . 1<br>NOT REFERRED 2
349	**REFERRAL MALNUTRITION**<br><br>CHECK 333:<br>REFER IF MUAC <14.0 CM	REFERRED . . . . 1<br>NOT REFERRED 2	REFERRED . . . . 1<br>NOT REFERRED 2	REFERRED . . . . 1<br>NOT REFERRED 2
350	**REFERRAL PRESUMED SHISTOSOMIASIS**<br><br>CHECK 340:<br>REFER IF HEMATURIA POSITIVE (340=1)	REFERRED . . . . 1<br>NOT REFERRED 2	REFERRED . . . . 1<br>NOT REFERRED 2	REFERRED . . . . 1<br>NOT REFERRED 2
351	GO BACK TO 302 IN NEXT COLUMN OR IN THE FIRST COLUMN OF THE NEXT PAGE OF THIS QUESTIONNAIRE;<br>IF NO MORE CHILDREN 5-14 YEARS, GO TO 300.			

<u>BIOLOGICAL INFORMATION FOR WOMEN AGE 15-49 YEARS</u>

400	CHECK COLUMN 1 IN HOUSEHOLD QUESTIONNAIRE. RECORD THE LINE NUMBER, NAME, AGE, AND MARITAL STATUS FOR ALL ELIGIBLE WOMEN IN 401, 402, AND 403.<br>IF THERE ARE MORE THAN THREE WOMEN, USE ADDITIONAL QUESTIONNAIRE(S) BOOKLET AND USE THE DUPLICATE HH LABEL(S).			
		WOMAN 1	WOMAN 2	WOMAN 3
401	CHECK HOUSEHOLD QUESTIONNAIRE:<br><br>LINE NUMBER FROM COLUMN 1.<br><br>NAME FROM COLUMN 2.	LINE NUMBER . . . . [ ][ ]<br><br>NAME ______________	LINE NUMBER . . . . [ ][ ]<br><br>NAME ______________	LINE NUMBER . . . . [ ][ ]<br><br>NAME ______________
402	CHECK HOUSEHOLD QUESTIONNAIRE COLUMN 7 (AGE):	15-17 YEARS . . . . 1<br>18-49 YEARS 2	15-17 YEARS . . . . 1<br>18-49 YEARS 2	15-17 YEARS . . . . 1<br>18-49 YEARS 2
403	CHECK HOUSEHOLD QUESTIONNAIRE COLUMN 8 (MARITAL	CODE 4 (NEVER IN UNION) . 1<br>OTHER 2	CODE 4 (NEVER IN UNION) . 1<br>OTHER 2	CODE 4 (NEVER IN UNION) . 1<br>OTHER 2
404	WOMAN LABEL	PUT THE WOMAN QUESTIONNAIRE BAR CODE LABEL HERE.	PUT THE WOMAN QUESTIONNAIRE BAR CODE LABEL HERE.	PUT THE WOMAN QUESTIONNAIRE BAR CODE LABEL HERE.
405	CHECK 402: AGE	15-17 YEARS . . . . 1<br>18-49 YEARS . . . . 2<br>(SKIP TO 407)	15-17 YEARS . . . . 1<br>18-49 YEARS . . . . 2<br>(SKIP TO 407)	15-17 YEARS . . . . 1<br>18-49 YEARS . . . . 2<br>(SKIP TO 407)
406	CHECK 403: MARITAL STATUS	CODE 4 (NEVER IN UNION) . 1<br>(SKIP TO 409)<br>OTHER 2	CODE 4 (NEVER IN UNION) . 1<br>(SKIP TO 409)<br>OTHER 2	CODE 4 (NEVER IN UNION) . 1<br>(SKIP TO 409)<br>OTHER 2

		WOMAN 1	WOMAN 2	WOMAN 3
	LINE NUMBER FROM COLUMN 1.<br><br>NAME FROM COLUMN 2.	LINE NUMBER . . . . [ ][ ]<br><br>NAME ________	LINE NUMBER . . . . [ ][ ]<br><br>NAME ________	LINE NUMBER . . . . [ ][ ]<br><br>NAME ________
**ADULT RESPONDENT CONSENT**	**ADULT RESPONDENT CONSENT FOR ANTHROPOMETRY AND BIOLOGICAL TESTING FROM RESPONDENT**			
407	ASK CONSENT FOR ANTHROPOMETRY AND BIOLOGICAL TESTING FROM RESPONDENT	As part of this survey we are asking women from all over this country to allow us to weigh and measure you. In addition to weigh and measuring you we would like to take a sample of your blood and urine. The tests are safe. Some tests may cause you slight discomfort, such as taking a blood sample. For all tests, there will be a brand new set of equipment used to take your blood and collect your urine, which is clean and completely safe. The equipment will be thrown away after it has been used on you.<br>With the blood we will test you for anemia and malaria. Anemia is a serious health problem that usually results from poor nutrition, infection, or chronic disease. Malaria can also be serious and can lead to you becoming anemic or making the anemia worse. You will be given these results immediately. If needed you will be referred to a local health facility for treatment. The rest of the blood will be sent to a laboratory to be tested for other vitamins and minerals, such as vitamin A and iron. The results from these tests will not be reported back to you as it will take some time to process the blood. The results will be kept strictly confidential and will not be shared with anyone other than members of our survey team.<br>This information will help the Ministry of Health understand better what problems women in Malawi are experiencing and help them to improve the health and nutrition programs here, which will benefit all women in Malawi.<br>Do you have any questions?<br>You can say yes or no. It is up to you to decide.<br>Will you participate in these tests?		
408	CIRCLE THE CODE AND SIGN YOUR NAME.	AGREED, ANTHROPOM. MEASURES ONLY . . . . 1<br>AGREED, BLOOD TEST ONLY . . . . 2<br>AGREED, URINE TEST ONLY . . . . 3<br>AGREED, ANTHROPO& BLOOD TEST ONLY . . . . 4<br>AGREED, ANTHROPO& URINE TEST ONLY . . . . 5<br>AGREED, BLOOD& URINE TESTS ONLY . . . . 6<br>AGREED, ANTHROPO & BLOOD&URINE TESTS . . . . . . 7<br>RESPONDENT REFUSED . . . . 8<br>________<br>(SIGN AND ENTER YOUR ID NUMBER)<br>[ ][ ]<br>(SKIP TO 413)<br><br>NOT PRESENT/OTHER . . . . . . 9<br>(SKIP TO 413)	AGREED, ANTHROPOM. MEASURES ONLY . . . . 1<br>AGREED, BLOOD TEST ONLY . . . . 2<br>AGREED, URINE TEST ONLY . . . . 3<br>AGREED, ANTHROPO& BLOOD TEST ONLY . . . . 4<br>AGREED, ANTHROPO& URINE TEST ONLY . . . . 5<br>AGREED, BLOOD& URINE TESTS ONLY . . . . 6<br>AGREED, ANTHROPO & BLOOD&URINE TESTS . . . . . . 7<br>RESPONDENT REFUSED . . . . 8<br>________<br>(SIGN AND ENTER YOUR ID NUMBER)<br>[ ][ ]<br>(SKIP TO 413)<br><br>NOT PRESENT/OTHER . . . . . . 9<br>(SKIP TO 413)	AGREED, ANTHROPOM. MEASURES ONLY . . . . 1<br>AGREED, BLOOD TEST ONLY . . . . 2<br>AGREED, URINE TEST ONLY . . . . 3<br>AGREED, ANTHROPO& BLOOD TEST ONLY . . . . 4<br>AGREED, ANTHROPO& URINE TEST ONLY . . . . 5<br>AGREED, BLOOD& URINE TESTS ONLY . . . . 6<br>AGREED, ANTHROPO & BLOOD&URINE TESTS . . . . . . 7<br>RESPONDENT REFUSED . . . . 8<br>________<br>(SIGN AND ENTER YOUR ID NUMBER)<br>[ ][ ]<br>(SKIP TO 413)<br><br>NOT PRESENT/OTHER . . . . . . 9<br>(SKIP TO 413)

		WOMAN 1	WOMAN 2	WOMAN 3
	LINE NUMBER FROM COLUMN 1.<br><br>NAME FROM COLUMN 2.	LINE NUMBER . . . . [ ][ ]<br><br>NAME ________	LINE NUMBER . . . . [ ][ ]<br><br>NAME ________	LINE NUMBER . . . . [ ][ ]<br><br>NAME ________

PARENT / RESP ADULT CONSENT

PARENTAL/RESPONSIBLE ADULT CONSENT FOR ANTHROPOMETRY AND BIOLOGICAL TESTING OF A MINOR

409	ASK CONSENT FOR ANTHROPOMETRY AND BIOLOGICAL TESTING FROM PARENT/ADULT.	As part of this survey we are asking women from all over this country to allow us to weigh and measure you. In addition to weigh and measuring (NAME OF MINOR) we would like to take a sample of (NAME OF MINOR) blood and urine. The tests are safe. Some tests may cause you slight discomfort, such as taking a blood sample. For all tests, there will be a brand new set of equipment used to take HER/HIS blood and collect your urine, which is clean and completely safe. The equipment will be thrown away after it has been used on you.<br>With the blood we will test (NAME OF MINOR) for anemia and malaria. Anemia is a serious health problem that usually results from poor nutrition, infection, or chronic disease. Malaria can also be serious and can lead to HER/HIM becoming anemic or making the anemia worse. You and (NAME OF MINOR) will be given these results immediately. If needed you (NAME OF MINOR) will be referred to a local health facility for treatment. The rest of the blood will be sent to a laboratory to be tested for other vitamins and minerals, such as vitamin A and iron. The results from these tests will not be reported back to you as it will take some time to process the blood. The results will be kept strictly confidential and will not be shared with anyone other than members of our survey team.<br>This information will help the Ministry of Health understand better what problems women in Malawi are experiencing and help them to improve the health and nutrition programs here, which will benefit all women in Malawi.<br><br>Do you have any questions?<br>You can say yes or no. It is up to you to decide.<br>Will you allow (NAME OF MINOR) to participate in these tests?		
410	CIRCLE THE CODE AND SIGN YOUR NAME.	AGREED, ANTHROPOM. MEASURES ONLY . . . . 1<br>AGREED, BLOOD TEST ONLY . . . . 2<br>AGREED, URINE TEST ONLY . . . . 3<br>AGREED, ANTHROPO& BLOOD TEST ONLY . . . . 4<br>AGREED, ANTHROPO& URINE TEST ONLY . . . . 5<br>AGREED, BLOOD& URINE TESTS ONLY . . . . 6<br>AGREED, ANTHROPO & BLOOD&URINE TESTS . . . . . . 7<br>RESPONDENT REFUSED . . . . 8<br>________ (SIGN)<br>(IF REFUSED, SKIP TO 413)<br><br>NOT PRESENT/OTHER . . . . . . 9<br>(SKIP TO 413)	AGREED, ANTHROPOM. MEASURES ONLY . . . . 1<br>AGREED, BLOOD TEST ONLY . . . . 2<br>AGREED, URINE TEST ONLY . . . . 3<br>AGREED, ANTHROPO& BLOOD TEST ONLY . . . . 4<br>AGREED, ANTHROPO& URINE TEST ONLY . . . . 5<br>AGREED, BLOOD& URINE TESTS ONLY . . . . 6<br>AGREED, ANTHROPO & BLOOD&URINE TESTS . . . . . . 7<br>RESPONDENT REFUSED . . . . 8<br>________ (SIGN)<br>(IF REFUSED, SKIP TO 413)<br><br>NOT PRESENT/OTHER . . . . . . 9<br>(SKIP TO 413)	AGREED, ANTHROPOM. MEASURES ONLY . . . . 1<br>AGREED, BLOOD TEST ONLY . . . . 2<br>AGREED, URINE TEST ONLY . . . . 3<br>AGREED, ANTHROPO& BLOOD TEST ONLY . . . . 4<br>AGREED, ANTHROPO& URINE TEST ONLY . . . . 5<br>AGREED, BLOOD& URINE TESTS ONLY . . . . 6<br>AGREED, ANTHROPO & BLOOD&URINE TESTS . . . . . . 7<br>RESPONDENT REFUSED . . . . 8<br>________ (SIGN)<br>(IF REFUSED, SKIP TO 413)<br><br>NOT PRESENT/OTHER . . . . . . 9<br>(SKIP TO 413)

		WOMAN 1	WOMAN 2	WOMAN 3
	LINE NUMBER FROM COLUMN 1.<br><br>NAME FROM COLUMN 2.	LINE NUMBER . . . . [ ][ ]<br><br>NAME ____________	LINE NUMBER . . . . [ ][ ]<br><br>NAME ____________	LINE NUMBER . . . . [ ][ ]<br><br>NAME ____________
	**MINOR RESPONDENT CONSENT FOR ANTHROPOMETRY AND BIOLOGICAL TESTING**			
411	ASK CONSENT FOR ANTHROPOMETRY AND BIOLOGICAL TESTING FROM MINOR RESPONDENT.	As part of this survey we are asking women from all over this country to allow us to weigh and measure you. In addition to weigh and measuring you we would like to take a sample of your blood and urine. The tests are safe. Some tests may cause you slight discomfort, such as taking a blood sample. For all tests, there will be a brand new set of equipment used to take your blood and collect your urine, which is clean and completely safe. The equipment will be thrown away after it has been used on you.<br>With the blood we will test you for anemia and malaria. Anemia is a serious health problem that usually results from poor nutrition, infection, or chronic disease. Malaria can also be serious and can lead to you becoming anemic or making the anemia worse. You will be given these results immediately. If needed you will be referred to a local health facility for treatment. The rest of the blood will be sent to a laboratory to be tested for other vitamins and minerals, such as vitamin A and iron. The results from these tests will not be reported back to you as it will take some time to process the blood. The results will be kept strictly confidential and will not be shared with anyone other than members of our survey team.<br>This information will help the Ministry of Health understand better what problems women in Malawi are experiencing and help them to improve the health and nutrition programs here, which will benefit all women in Malawi.<br><br>Do you have any questions?<br>You can say yes or no. It is up to you to decide.<br>Will you participate in these tests?		
412	CIRCLE THE CODE AND SIGN YOUR NAME.	AGREED, ANTHROPOM. MEASURES ONLY . . . . 1<br>AGREED, BLOOD TEST ONLY . . . . 2<br>AGREED, URINE TEST ONLY . . . . 3<br>AGREED, ANTHROPO& BLOOD TEST ONLY . . . . 4<br>AGREED, ANTHROPO& URINE TEST ONLY . . . . 5<br>AGREED, BLOOD& URINE TESTS ONLY . . . . 6<br>AGREED, ANTHROPO & BLOOD&URINE TESTS . . . . . . 7<br>RESPONDENT REFUSED . . . . 8<br>(SIGN AND ENTER YOUR ID NUMBER) [ ][ ]<br>NOT PRESENT/OTHER 9	AGREED, ANTHROPOM. MEASURES ONLY . . . . 1<br>AGREED, BLOOD TEST ONLY . . . . 2<br>AGREED, URINE TEST ONLY . . . . 3<br>AGREED, ANTHROPO& BLOOD TEST ONLY . . . . 4<br>AGREED, ANTHROPO& URINE TEST ONLY . . . . 5<br>AGREED, BLOOD& URINE TESTS ONLY . . . . 6<br>AGREED, ANTHROPO & BLOOD&URINE TESTS . . . . . . 7<br>RESPONDENT REFUSED . . . . 8<br>(SIGN AND ENTER YOUR ID NUMBER) [ ][ ]<br>NOT PRESENT/OTHER 9	AGREED, ANTHROPOM. MEASURES ONLY . . . . 1<br>AGREED, BLOOD TEST ONLY . . . . 2<br>AGREED, URINE TEST ONLY . . . . 3<br>AGREED, ANTHROPO& BLOOD TEST ONLY . . . . 4<br>AGREED, ANTHROPO& URINE TEST ONLY . . . . 5<br>AGREED, BLOOD& URINE TESTS ONLY . . . . 6<br>AGREED, ANTHROPO & BLOOD&URINE TESTS . . . . . . 7<br>RESPONDENT REFUSED . . . . 8<br>(SIGN AND ENTER YOUR ID NUMBER) [ ][ ]<br>NOT PRESENT/OTHER 9
413	NURSE: ENTER YOUR ID NUMBER.	[ ][ ]<br>ID NUMBER	[ ][ ]<br>ID NUMBER	[ ][ ]<br>ID NUMBER
414	In the last week, have you taken iron tablets or iron syrup?<br><br>SHOW COMMON IRON TABLETS IN MALAWI.	YES . . . . 1<br>NO . . . . 2<br>DON'T KNOW 8	YES . . . . 1<br>NO . . . . 2<br>DON'T KNOW 8	YES . . . . 1<br>NO . . . . 2<br>DON'T KNOW 8
415	In the last month, have you taken any other kind of vitamin or mineral tablet/syrup/powder?	YES . . . . 1<br>NO 2	YES . . . . 1<br>NO 2	YES . . . . 1<br>NO 2

MINOR RESPONDENT CONSENT

BIOLOGICAL INFORMATION FOR WOMEN AGE 15-49 YEARS

		WOMAN 1	WOMAN 2	WOMAN 3
	LINE NUMBER FROM COLUMN 1.<br><br>NAME FROM COLUMN 2.	LINE NUMBER . . . . [ ][ ]<br><br>NAME ______	LINE NUMBER . . . . [ ][ ]<br><br>NAME ______	LINE NUMBER . . . . [ ][ ]<br><br>NAME ______
416	Have you had a fever in the last 2 weeks?	YES . . . . . 1<br>NO 2	YES . . . . . 1<br>NO 2	YES . . . . . 1<br>NO 2
417	Have you had a fever in the last 24 hours?	YES . . . . . 1<br>NO 2	YES . . . . . 1<br>NO 2	YES . . . . . 1<br>NO 2
418	Have you had a cough or breathing problems in the last 2 weeks?	YES . . . . . 1<br>NO 2	YES . . . . . 1<br>NO 2	YES . . . . . 1<br>NO 2
419	Have you had diarrhea in the last 2 weeks?	YES . . . . . 1<br>NO 2	YES . . . . . 1<br>NO 2	YES . . . . . 1<br>NO 2
420	Have you been ill with malaria in the last 2 weeks?	YES . . . . . 1<br>NO 2	YES . . . . . 1<br>NO 2	YES . . . . . 1<br>NO 2
421	In the past 2 weeks did you notice blood, other than menstrual blood, in your urine?	YES . . . . . 1<br>NO 2	YES . . . . . 1<br>NO 2	YES . . . . . 1<br>NO 2
422	In the last six months, have you received a blood transfusion?	YES . . . . . 1<br>NO 2	YES . . . . . 1<br>NO 2	YES . . . . . 1<br>NO 2
423	Are you pregnant?	YES . . . . . 1<br>NO 2	YES . . . . . 1<br>NO 2	YES . . . . . 1<br>NO 2
424	At what time approximately did you eat your most recent meal?	HOURS . . . . . [ ][ ]<br>MINUTES . . . . . [ ][ ]	HOURS . . . . . [ ][ ]<br>MINUTES . . . . . [ ][ ]	HOURS . . . . . [ ][ ]<br>MINUTES . . . . . [ ][ ]
425	PROCEED ONLY WITH MEASUREMENTS AND/OR TESTS (S) FOR WHICH CONSENT HAS BEEN OBTAINED. IF ADULT RESPONDENT, CHECK 408; IF MINOR RESPONDENT, CHECK 410 AND 412.			
426	CHECK 408, 410 or 412 AGREED FOR BLOOD TEST	CODE '2', '4', '6' OR '7' CIRCLED [ ]<br>CODE '2', '4', 6' OR '7' NOT CIRCLED [ ] (SKIP TO 434)	CODE '2', '4', '6' OR '7' CIRCLED [ ]<br>CODE '2', '4', 6' OR '7' NOT CIRCLED [ ] (SKIP TO 434)	CODE '2', '4', '6' OR '7' CIRCLED [ ]<br>CODE '2', '4', 6' OR '7' NOT CIRCLED [ ] (SKIP TO 434)
427	**PURPLE TOP TUBE (EDTA)**<br>RECORD THE RESULT OF THE PURPLE TOP TUBE BLOOD SAMPLE COLLECTION	PURPLE TOP TUBE COLLECTED 1<br>INSUFFICIENT SAMPLE . . . . . 2<br>REFUSED . . . . . 3<br>OTHER 6	PURPLE TOP TUBE COLLECTED 1<br>INSUFFICIENT SAMPLE . . . . . 2<br>REFUSED . . . . . 3<br>OTHER 6	PURPLE TOP TUBE COLLECTED 1<br>INSUFFICIENT SAMPLE . . . . . 2<br>REFUSED . . . . . 3<br>OTHER 6
428	**BLUE TOP TUBE (METAL**<br>RECORD THE RESULT OF THE BLUE TOP TUBE BLOOD SAMPLE COLLECTION	BLUE TOP TUBE COLLECTED . 1<br>INSUFFICIENT SAMPLE . . . . . 2<br>REFUSED . . . . . 3<br>OTHER 6	BLUE TOP TUBE COLLECTED . 1<br>INSUFFICIENT SAMPLE . . . . . 2<br>REFUSED . . . . . 3<br>OTHER 6	BLUE TOP TUBE COLLECTED . 1<br>INSUFFICIENT SAMPLE . . . . . 2<br>REFUSED . . . . . 3<br>OTHER 6

BIOLOGICAL INFORMATION FOR WOMEN AGE 15-49 YEARS

		WOMAN 1	WOMAN 2	WOMAN 3
	LINE NUMBER FROM COLUMN 1.<br>NAME FROM COLUMN 2.	LINE NUMBER . . . . [ ][ ]<br>NAME ______	LINE NUMBER . . . . [ ][ ]<br>NAME ______	LINE NUMBER . . . . [ ][ ]<br>NAME ______
429	DATE BLOOD SAMPLE TAKEN	DAY . . . . [ ][ ]<br>MONTH . . . . [ ][ ]<br>YEAR . . . . [ ][ ][ ][ ]	DAY . . . . [ ][ ]<br>MONTH . . . . [ ][ ]<br>YEAR . . . . [ ][ ][ ][ ]	DAY . . . . [ ][ ]<br>MONTH . . . . [ ][ ]<br>YEAR . . . . [ ][ ][ ][ ]
430	TIME BLOOD DRAWN	HOURS . . . . [ ][ ]<br>MINUTES . . . . [ ][ ]	HOURS . . . . [ ][ ]<br>MINUTES . . . . [ ][ ]	HOURS . . . . [ ][ ]<br>MINUTES . . . . [ ][ ]
431	RECORD MALARIA TEST RESULT	POSITIVE . . . . 1<br>NEGATIVE . . . . 2<br>INVALID . . . . 3<br>REFUSED . . . . 4<br>NOT PRESENT . . . . 5<br>OTHER 6	POSITIVE . . . . 1<br>NEGATIVE . . . . 2<br>INVALID . . . . 3<br>REFUSED . . . . 4<br>NOT PRESENT . . . . 5<br>OTHER 6	POSITIVE . . . . 1<br>NEGATIVE . . . . 2<br>INVALID . . . . 3<br>REFUSED . . . . 4<br>NOT PRESENT . . . . 5<br>OTHER 6
432	RECORD HEMOGLOBIN LEVEL HERE	G/DL . . . . . . [ ][ ].[ ]<br>INSUFFICIENT . . . . 99.3<br>REFUSED . . . . 99.4<br>NOT PRESENT . . . . 99.5<br>OTHER 99.6	G/DL . . . . . . [ ][ ].[ ]<br>INSUFFICIENT . . . . 99.3<br>REFUSED . . . . 99.4<br>NOT PRESENT . . . . 99.5<br>OTHER 99.6	G/DL . . . . . . [ ][ ].[ ]<br>INSUFFICIENT . . . . 99.3<br>REFUSED . . . . 99.4<br>NOT PRESENT . . . . 99.5<br>OTHER 99.6
433	RECORD POC HEMOGLOBIN LEVEL HERE	VISUAL G/DL . . . . . . [ ][ ].[ ]<br>APP G/DL . . . . . . [ ][ ].[ ]<br>BLUE . . . . 99.3<br>GREEN . . . . 99.4<br>YELLOW . . . . 99.5<br>ORANGE . . . . 99.6<br>RED 99.7	VISUAL G/DL . . . . . . [ ][ ].[ ]<br>APP G/DL . . . . . . [ ][ ].[ ]<br>BLUE . . . . 99.3<br>GREEN . . . . 99.4<br>YELLOW . . . . 99.5<br>ORANGE . . . . 99.6<br>RED 99.7	VISUAL G/DL . . . . . . [ ][ ].[ ]<br>APP G/DL . . . . . . [ ][ ].[ ]<br>BLUE . . . . 99.3<br>GREEN . . . . 99.4<br>YELLOW . . . . 99.5<br>ORANGE . . . . 99.6<br>RED 99.7
434	CHECK 408, 410, 412: AGREED FOR ANTROPOMETRIC MEASUREMENTS	CODE '1', '4', '5' OR '7' CIRCLED [ ]<br>CODE '1', '4', '5' OR '7' NOT CIRCLED [ ] (SKIP TO 439)	CODE '1', '4', '5' OR '7' CIRCLED [ ]<br>CODE '1', '4', '5' OR '7' NOT CIRCLED [ ] (SKIP TO 439)	CODE '1', '4', '5' OR '7' CIRCLED [ ]<br>CODE '1', '4', '5' OR '7' NOT CIRCLED [ ] (SKIP TO 439)
435	WEIGHT IN KILOGRAMS.	KG. . . . . [ ][ ][ ].[ ][ ]<br>REFUSED . . . . 999.94<br>NOT PRESENT . . . . . 999.95<br>OTHER 999.96	KG. . . . . [ ][ ][ ].[ ][ ]<br>REFUSED . . . . 999.94<br>NOT PRESENT . . . . . 999.95<br>OTHER 999.96	KG. . . . . [ ][ ][ ].[ ][ ]<br>REFUSED . . . . 999.94<br>NOT PRESENT . . . . . 999.95<br>OTHER 999.96
436	HEIGHT IN CENTIMETERS.	CM. . . . . [ ][ ][ ].[ ]<br>REFUSED . . . . 999.4<br>NOT PRESENT . . . . . . 999.5<br>OTHER 999.6	CM. . . . . [ ][ ][ ].[ ]<br>REFUSED . . . . 999.4<br>NOT PRESENT . . . . . . 999.5<br>OTHER 999.6	CM. . . . . [ ][ ][ ].[ ]<br>REFUSED . . . . 999.4<br>NOT PRESENT . . . . . . 999.5<br>OTHER 999.6
438	MID-UPPER ARM CIRCUMFERENCE (MUAC) IN CENTIMETERS.	CM . . . . [ ][ ].[ ][ ]<br>REFUSED . . . . 9995<br>OTHER 9996	CM . . . . [ ][ ].[ ][ ]<br>REFUSED . . . . 9995<br>OTHER 9996	CM . [ ][ ].[ ][ ]<br>REFUSED . . . . 9995<br>OTHER 9996

BIOLOGICAL INFORMATION FOR WOMEN AGE 15-49 YEARS

		WOMAN 1	WOMAN 2	WOMAN 3
	LINE NUMBER FROM COLUMN 1.<br><br>NAME FROM COLUMN 2.	LINE NUMBER . . . .<br><br>NAME ____________	LINE NUMBER . . . .<br><br>NAME ____________	LINE NUMBER . . . .<br><br>NAME ____________
439	LAB TECH: ENTER YOUR ID NUMBER.	ID NUMBER	ID NUMBER	ID NUMBER
440	TIME BLOOD CENTRIFUGED	HOURS . . . .<br>MINUTES	HOURS . . . .<br>MINUTES	HOURS . . . .<br>MINUTES . . . .
441	CHECK 408, 410, 412: AGREED FOR URINE TEST	CODE '3', '5', '6' OR '7' CIRCLED<br>CODE '3', '5', 6' OR '7' NOT CIRCLED (SKIP TO 446)	CODE '3', '5', '6' OR '7' CIRCLED<br>CODE '3', '5', 6' OR '7' NOT CIRCLED (SKIP TO 446)	CODE '3', '5', '6' OR '7' CIRCLED<br>CODE '3', '5', 6' OR '7' NOT CIRCLED (SKIP TO 446)
442	In order to determine if you have blood in your urine, which might suggest that you have schistosomiasis, we would like to collect a urine sample from you. If you can provide this now, we appreciate it. If not now, we can come back to pick up the sample at a later time.<br><br>INSTRUCTIONS IF UNABLE TO PRODUCE AT WILL:<br><br>FOR URINE: We will return tomorrow to pick up your urine. We would like the freshest urine you can give us. Please use this cup to collect your urine.			
443	**URINE SPECIMEN**<br>RECORD THE RESULT OF URINE SPECIMEN COLLECTION	URINE SPECIMEN COLLECTED ... 1<br>INSUFFICIENT SAMPLE . . . . 2<br>REFUSED . . . . 3<br>OTHER 6	URINE SPECIMEN COLLECTED ... 1<br>INSUFFICIENT SAMPLE . . . . 2<br>REFUSED . . . . 3<br>OTHER 6	URINE SPECIMEN COLLECTED ... 1<br>INSUFFICIENT SAMPLE . . . . 2<br>REFUSED . . . . 3<br>OTHER 6
444	DATE URINE SAMPLE COLLECTED (DAY/MONTH/YEAR)	DAY . . . .<br>MONTH . . . .<br>YEAR	DAY . . . .<br>MONTH . . . .<br>YEAR	DAY . . . .<br>MONTH . . . .<br>YEAR . . . .
445	RECORD RESULTS OF DIPSTICK FOR HEMATURIA	POSITIVE . . . . 1<br>NEGATIVE . . . . 2<br>INVALID . . . . 3<br>REFUSED . . . . 4<br>NOT PRESENT . . . . 5<br>OTHER 6	POSITIVE . . . . 1<br>NEGATIVE . . . . 2<br>INVALID . . . . 3<br>REFUSED . . . . 4<br>NOT PRESENT . . . . 5<br>OTHER 6	POSITIVE . . . . 1<br>NEGATIVE . . . . 2<br>INVALID . . . . 3<br>REFUSED . . . . 4<br>NOT PRESENT . . . . 5<br>OTHER 6
446	CHECK FRONT COVER<br><br>HOUSEHOLD SELECTED FOR MRDR TEST<br><br>HOUSEHOLD NOT SELECTED FOR MRDR TEST → 452			
447	CHECK 427:<br>WAS THE FIRST BLOOD SAMPLE COLLECTED?	YES<br>NO (SKIP TO 452)	YES<br>NO (SKIP TO 452)	YES<br>NO (SKIP TO 452)

BIOLOGICAL INFORMATION FOR WOMEN AGE 15-49 YEARS

		WOMAN 1	WOMAN 2	WOMAN 3
	LINE NUMBER FROM COLUMN 1.<br><br>NAME FROM COLUMN 2.	LINE NUMBER . . . .<br><br>NAME ____________	LINE NUMBER . . . .<br><br>NAME ____________	LINE NUMBER . . . .<br><br>NAME ____________
448	As part of this survey we are asking some people to participate in an additional test. We would also like to include you in an additional test to find out more information about vitamin A in the body. This test will involve giving you a small amount of liquid to swallow with a snack. We will then have to wait about 4 hours and then take an additional small blood sample.<br>The results from this test will help the Ministry of Health understand better how well the food fortification program in Malawi is working and if other improvements are necessary.<br><br>Do you have any questions?<br>You can say yes or no. It is up to you to decide.<br>Will you participate in these tests?			
448A	CONSENT TO MRDR	CONSENT TO MRDR TEST GRANTED<br>CONSENT TO MRDR TEST NOT GRANTED (SKIP TO 452)	CONSENT TO MRDR TEST GRANTED<br>CONSENT TO MRDR TEST NOT GRANTED (SKIP TO 452)	CONSENT TO MRDR TEST GRANTED<br>CONSENT TO MRDR TEST NOT GRANTED (SKIP TO 452)
449	TIME OF INGESTING VITAMIN A2	HOURS . . . .<br>MINUTES	HOURS . . . .<br>MINUTES	HOURS . . . .<br>MINUTES . . . .
450	**MRDR TEST - BLOOD SAMPLE**<br>RECORD THE RESULT OF MRDR TEST BLOOD SAMPLE COLLECTION	MRDR TEST-SAMPLE COLLECTED 1<br>INSUFFICIENT SAMPLE . 2<br>REFUSED . . . . 3<br>OTHER 6	MRDR TEST-SAMPLE COLLECTED 1<br>INSUFFICIENT SAMPLE . 2<br>REFUSED . . . . 3<br>OTHER 6	MRDR TEST-SAMPLE COLLECTED 1<br>INSUFFICIENT SAMPLE . 2<br>REFUSED . . . . 3<br>OTHER 6
451	TIME SECOND BLOOD DRAWN FOR MRDR TESTING	HOURS . . . .<br>MINUTES	HOURS . . . .<br>MINUTES	HOURS . . . .<br>MINUTES . . . .
452	**REFERRAL CLINICAL MALARIA**<br><br>CHECK 431:<br>REFER IF RDT POSITIVE (431=1)	REFERRED . . . . 1<br>NOT REFERRED 2	REFERRED . . . . 1<br>NOT REFERRED 2	REFERRED . . . . 1<br>NOT REFERRED 2
453	**REFERRAL SEVERE ANEMIA**<br><br>CHECK 432:<br>REFER IF Hb <7 G/DL	REFERRED . . . . 1<br>NOT REFERRED 2	REFERRED . . . . 1<br>NOT REFERRED 2	REFERRED . . . . 1<br>NOT REFERRED 2
454	**REFERRAL MALNUTRITION**<br><br>CHECK 438:<br>REFER IF MUAC<19.0CM.	REFERRED . . . . 1<br>NOT REFERRED 2	REFERRED . . . . 1<br>NOT REFERRED 2	REFERRED . . . . 1<br>NOT REFERRED 2
456	**REFERRAL PRESUMED SHISTO**<br><br>CHECK 423 AND 445:<br>REFER IF PREGNANT (423=1) AND HEMATURIA POSITIVE (445=1)	REFERRED . . . . 1<br>NOT REFERRED 2	REFERRED . . . . 1<br>NOT REFERRED 2	REFERRED . . . . 1<br>NOT REFERRED 2
457	GO BACK TO 404 IN NEXT COLUMN OF THIS QUESTIONNAIRE OR IN THE FIRST COLUMN OF AN ADDITIONAL QUESTIONNAIRE;<br>IF NO MORE WOMEN 15-49 YEARS, GO TO 500.			

500	CHECK COLUMN 1 IN THE HOUSEHOLD QUESTIONNAIRE. RECORD THE LINE NUMBER AND NAME FOR ALL MEN AGE 20-54 IN 501.<br>IF THERE ARE MORE THAN THREE MEN, USE ADDITIONAL QUESTIONNAIRE(S) BOOKLET AND USE THE DUPLICATE HH LABEL(S).			
		MAN 1	MAN 2	MAN 3
501	CHECK HOUSEHOLD QUESTIONNAIRE:			
	LINE NUMBER FROM COLUMN 10.<br><br>NAME FROM COLUMN 2.	LINE NUMBER . . . . ☐☐<br><br>NAME ____________	LINE NUMBER . . . . ☐☐<br><br>NAME ____________	LINE NUMBER . . . . ☐☐<br><br>NAME ____________
502	MAN LABEL	PUT THE MAN QUESTIONNAIRE BAR CODE LABEL HERE.	PUT THE MAN QUESTIONNAIRE BAR CODE LABEL HERE.	PUT THE MAN QUESTIONNAIRE BAR CODE LABEL HERE.
503	ASK CONSENT FOR FOR ANTHROPOMETRY AND BIOLOGICAL TESTING	As part of this survey we are asking men from all over this country to allow us to weigh and measure you. In addition to weigh and measuring you we would like to take a sample of your blood and urine. The tests are safe. Some tests may cause you slight discomfort, such as taking a blood sample. For all tests, there will be a brand new set of equipment used to take your blood and collect your urine, which is clean and completely safe. The equipment will be thrown away after it has been used on you.<br>With the blood we will test you for anemia and malaria. Anemia is a serious health problem that usually results from poor nutrition, infection, or chronic disease. Malaria can also be serious and can lead to you becoming anemic or making the anemia worse. You will be given these results immediately. If needed you will be referred to a local health facility for treatment. The rest of the blood will be sent to a laboratory to be tested for other vitamins and minerals, such as vitamin A and iron. The results from these tests will not be reported back to you as it will take some time to process the blood. The results will be kept strictly confidential and will not be shared with anyone other than members of our survey team.<br>This information will help the Ministry of Health understand better what problems men in Malawi are experiencing and help them to improve the health and nutrition programs here, which will benefit all men in Malawi.<br><br>Do you have any questions?<br>You can say yes or no. It is up to you to decide.<br>Will you participate in these tests?		
504	CIRCLE THE CODE AND SIGN YOUR NAME.	AGREED, ANTHROPOM. MEASURES ONLY . . . . 1<br>AGREED, BLOOD TEST ONLY . . . . 2<br>AGREED, URINE TEST ONLY . . . . 3<br>AGREED, ANTHROPO& BLOOD TEST ONLY . . . . 4<br>AGREED, ANTHROPO& URINE TEST ONLY . . . . 5<br>AGREED, BLOOD& URINE TESTS ONLY . . . . 6<br>AGREED **ALL**, ANTHROPO & BLOOD&URINE TESTS . . . . . . 7<br>RESPONDENT REFUSED . . . . 8<br><br>____________<br>(SIGN AND ENTER YOUR ID NUMBER) ☐☐☐<br><br>NOT PRESENT/OTHER 9	AGREED, ANTHROPOM. MEASURES ONLY . . . . 1<br>AGREED, BLOOD TEST ONLY . . . . 2<br>AGREED, URINE TEST ONLY . . . . 3<br>AGREED, ANTHROPO& BLOOD TEST ONLY . . . . 4<br>AGREED, ANTHROPO& URINE TEST ONLY . . . . 5<br>AGREED, BLOOD& URINE TESTS ONLY . . . . 6<br>AGREED **ALL**, ANTHROPO & BLOOD&URINE TESTS . . . . . . 7<br>RESPONDENT REFUSED . . . . 8<br><br>____________<br>(SIGN AND ENTER YOUR ID NUMBER) ☐☐☐<br><br>NOT PRESENT/OTHER 9	AGREED, ANTHROPOM. MEASURES ONLY . . . . 1<br>AGREED, BLOOD TEST ONLY . . . . 2<br>AGREED, URINE TEST ONLY . . . . 3<br>AGREED, ANTHROPO& BLOOD TEST ONLY . . . . 4<br>AGREED, ANTHROPO& URINE TEST ONLY . . . . 5<br>AGREED, BLOOD& URINE TESTS ONLY . . . . 6<br>AGREED **ALL**, ANTHROPO & BLOOD&URINE TESTS . . . . . . 7<br>RESPONDENT REFUSED . . . . 8<br><br>____________<br>(SIGN AND ENTER YOUR ID NUMBER) ☐☐☐<br><br>NOT PRESENT/OTHER 9
505	NURSE: ENTER YOUR ID NUMBER.	☐☐<br>ID NUMBER	☐☐<br>ID NUMBER	☐☐<br>ID NUMBER
506	In the last week, have you taken iron tablets or iron syrup?<br><br>SHOW COMMON IRON TABLETS IN MALAWI.	YES . . . . 1<br>NO 2	YES . . . . 1<br>NO 2	YES . . . . 1<br>NO 2
506A	In the last month, have you taken any other kind of vitamin or mineral tablet/syrup/powder?	YES . . . . 1<br>NO 2	YES . . . . 1<br>NO 2	YES . . . . 1<br>NO 2
507	Have you had a fever in the last 2 weeks?	YES . . . . 1<br>NO 2	YES . . . . 1<br>NO 2	YES . . . . 1<br>NO 2
508	Have you had a fever in the last 24 hours?	YES . . . . 1<br>NO 2	YES . . . . 1<br>NO 2	YES . . . . 1<br>NO 2
509	Have you had a cough or breathing problem in the last 2 weeks?	YES . . . . 1<br>NO 2	YES . . . . 1<br>NO 2	YES . . . . 1<br>NO 2
510	Have you had diarrhea in the last 2 weeks?	YES . . . . 1<br>NO 2	YES . . . . 1<br>NO 2	YES . . . . 1<br>NO 2
511	Have you been ill with malaria in the last 2 weeks?	YES . . . . 1<br>NO 2	YES . . . . 1<br>NO 2	YES . . . . 1<br>NO 2
512	In the past 2 weeks did you notice blood in your urine?	YES . . . . 1<br>NO 2	YES . . . . 1<br>NO 2	YES . . . . 1<br>NO 2

	LINE NUMBER FROM COLUMN 10.<br><br>NAME FROM COLUMN 2.	LINE NUMBER .... ☐☐<br><br>NAME ________	LINE NUMBER .... ☐☐<br><br>NAME ________	LINE NUMBER .... ☐☐<br><br>NAME ________
513	In the last six months, have you received a blood transfusion?	YES .... 1<br>NO 2	YES .... 1<br>NO 2	YES .... 1<br>NO 2
514	At what time approximately did you eat your most recent meal?	HOURS .... ☐☐<br>MINUTES .... ☐☐	HOURS .... ☐☐<br>MINUTES .... ☐☐	HOURS .... ☐☐<br>MINUTES .... ☐☐
515	CHECK 504<br>AGREED FOR BLOOD TEST	CODE '2', '4', '6' OR '7' CIRCLED ☐ ↓<br>CODE '2', '4', 6' OR '7' NOT CIRCLED ☐<br>(SKIP TO 523)	CODE '2', '4', '6' OR '7' CIRCLED ☐ ↓<br>CODE '2', '4', 6' OR '7' NOT CIRCLED ☐<br>(SKIP TO 523)	CODE '2', '4', '6' OR '7' CIRCLED ☐ ↓<br>CODE '2', '4', 6' OR '7' NOT CIRCLED ☐<br>(SKIP TO 523)
516	**PURPLE TOP TUBE**<br>RECORD THE RESULT OF THE PURPLE TOP TUBE BLOOD SAMPLE COLLECTION	PURPLE TOP TUBE COLLECTED .... 1<br>INSUFFICIENT SAMPLE .... 2<br>REFUSED .... 3<br>OTHER 6	PURPLE TOP TUBE COLLECTED .... 1<br>INSUFFICIENT SAMPLE .... 2<br>REFUSED .... 3<br>OTHER 6	PURPLE TOP TUBE COLLECTED .... 1<br>INSUFFICIENT SAMPLE .... 2<br>REFUSED .... 3<br>OTHER 6
517	**BLUE TOP TUBE (METAL FREE)**<br>RECORD THE RESULT OF THE BLUE TOP TUBE BLOOD SAMPLE COLLECTION	BLUE TOP TUBE COLLECTED .... 1<br>INSUFFICIENT SAMPLE .... 2<br>REFUSED .... 3<br>OTHER 6	BLUE TOP TUBE COLLECTED .... 1<br>INSUFFICIENT SAMPLE .... 2<br>REFUSED .... 3<br>OTHER 6	BLUE TOP TUBE COLLECTED .... 1<br>INSUFFICIENT SAMPLE .... 2<br>REFUSED .... 3<br>OTHER 6
518	DATE BLOOD SAMPLE TAKEN (DAY/MONTH/YEAR)	DAY .... ☐☐<br>MONTH .... ☐☐<br>YEAR .... ☐☐☐☐	DAY .... ☐☐<br>MONTH .... ☐☐<br>YEAR .... ☐☐☐☐	DAY .... ☐☐<br>MONTH .... ☐☐<br>YEAR .... ☐☐☐☐
519	TIME BLOOD DRAWN	HOURS .... ☐☐<br>MINUTES .... ☐☐	HOURS .... ☐☐<br>MINUTES .... ☐☐	HOURS .... ☐☐<br>MINUTES .... ☐☐
520	RECORD MALARIA TEST RESULT	POSITIVE .... 1<br>NEGATIVE .... 2<br>INVALID .... 3<br>REFUSED .... 4<br>NOT PRESENT .... 5<br>OTHER 6	POSITIVE .... 1<br>NEGATIVE .... 2<br>INVALID .... 3<br>REFUSED .... 4<br>NOT PRESENT .... 5<br>OTHER 6	POSITIVE .... 1<br>NEGATIVE .... 2<br>INVALID .... 3<br>REFUSED .... 4<br>NOT PRESENT .... 5<br>OTHER 6
521	RECORD HEMOGLOBIN LEVEL HERE	G/DL .... ☐☐.☐<br>INSUFFICIENT .... 99.3<br>REFUSED .... 99.4<br>NOT PRESENT .... 99.5<br>OTHER 99.6	G/DL .... ☐☐.☐<br>INSUFFICIENT .... 99.3<br>REFUSED .... 99.4<br>NOT PRESENT .... 99.5<br>OTHER 99.6	G/DL .... ☐☐.☐<br>INSUFFICIENT .... 99.3<br>REFUSED .... 99.4<br>NOT PRESENT .... 99.5<br>OTHER 99.6
522	RECORD POC HEMOGLOBIN LEVEL HERE	VISUAL G/DL .... ☐☐.☐<br>APP G/DL .... ☐☐.☐<br>BLUE .... 99.3<br>GREEN .... 99.4<br>YELLOW .... 99.5<br>ORANGE .... 99.6<br>RED 99.7	VISUAL G/DL .... ☐☐.☐<br>APP G/DL .... ☐☐.☐<br>BLUE .... 99.3<br>GREEN .... 99.4<br>YELLOW .... 99.5<br>ORANGE .... 99.6<br>RED 99.7	VISUAL G/DL .... ☐☐.☐<br>APP G/DL .... ☐☐.☐<br>BLUE .... 99.3<br>GREEN .... 99.4<br>YELLOW .... 99.5<br>ORANGE .... 99.6<br>RED 99.7
523	CHECK 504:<br>AGREED FOR ANTROPOMETRIC MEASUREMENTS	CODE '1', '4', '5' OR '7' CIRCLED ☐ ↓<br>CODE '1', '4', '5' OR '7' NOT CIRCLED ☐<br>(SKIP TO 528)	CODE '1', '4', '5' OR '7' CIRCLED ☐ ↓<br>CODE '1', '4', '5' OR '7' NOT CIRCLED ☐<br>(SKIP TO 528)	CODE '1', '4', '5' OR '7' CIRCLED ☐ ↓<br>CODE '1', '4', '5' OR '7' NOT CIRCLED ☐<br>(SKIP TO 528)
524	WEIGHT IN KILOGRAMS.	KG. .... ☐☐☐.☐☐<br>REFUSED .... 999.94<br>NOT PRESENT .... 999.95<br>OTHER 999.96	KG. .... ☐☐☐.☐☐<br>REFUSED .... 999.94<br>NOT PRESENT .... 999.95<br>OTHER 999.96	KG. .... ☐☐☐.☐☐<br>REFUSED .... 999.94<br>NOT PRESENT .... 999.95<br>OTHER 999.96
525	HEIGHT IN CENTIMETERS.	CM. .... ☐☐☐.☐<br>REFUSED .... 999.4<br>NOT PRESENT .... 999.5<br>OTHER 999.6	CM. .... ☐☐☐.☐<br>REFUSED .... 999.4<br>NOT PRESENT .... 999.5<br>OTHER 999.6	CM. .... ☐☐☐.☐<br>REFUSED .... 999.4<br>NOT PRESENT .... 999.5<br>OTHER 999.6

	LINE NUMBER FROM COLUMN 10.<br><br>NAME FROM COLUMN 2.	LINE NUMBER . . . .<br><br>NAME	LINE NUMBER . . . .<br><br>NAME	LINE NUMBER . . . .<br><br>NAME
527	MID-UPPER ARM CIRCUMFERENCE (MUAC) IN CENTIMETERS.	CM . . . .<br><br>REFUSED . . . . 99.95<br>OTHER 99.96	CM . . . .<br><br>REFUSED . . . . 9995<br>OTHER 9996	CM .<br><br>REFUSED . . . . 9995<br>OTHER 9996
528	LAB TECH: ENTER YOUR ID NUMBER.	ID NUMBER	ID NUMBER	ID NUMBER
529	TIME BLOOD CENTRIFUGED	HOURS . . . .<br>MINUTES	HOURS . . . .<br>MINUTES	HOURS . . . .<br>MINUTES . . . .
530	CHECK 504:<br>AGREED FOR URINE TEST	CODE '3', '5', '6' OR '7' CIRCLED<br>CODE '3', '5', 6' OR '7' NOT CIRCLED (SKIP TO 535)	CODE '3', '5', '6' OR '7' CIRCLED<br>CODE '3', '5', 6' OR '7' NOT CIRCLED (SKIP TO 535)	CODE '3', '5', '6' OR '7' CIRCLED<br>CODE '3', '5', 6' OR '7' NOT CIRCLED (SKIP TO 535)
531	In order to determine if you have blood in your urine, which might suggest that you have schistosomiasis, we would like to collect a urine sample from you. If you can provide this now, we appreciate it. If not now, we can come back to pick up the sample at a later time.<br><br>INSTRUCTIONS IF UNABLE TO PRODUCE AT WILL:<br><br>FOR URINE: We will return tomorrow to pick up your urine. We would like the freshest urine you can give us. Please use this cup to collect your urine.			
532	**URINE SPECIMEN**<br>RECORD THE RESULT OF URINE SPECIMEN COLLECTION	URINE SPECIMEN COLLECTED .... 1<br>INSUFFICIENT SAMPLE . . . . 2<br>REFUSED . . . . 3<br>OTHER 6	URINE SPECIMEN COLLECTED .... 1<br>INSUFFICIENT SAMPLE . . . . 2<br>REFUSED . . . . 3<br>OTHER 6	URINE SPECIMEN COLLECTED .... 1<br>INSUFFICIENT SAMPLE . . . . 2<br>REFUSED . . . . 3<br>OTHER 6
533	DATE URINE SAMPLE COLLECTED (DAY/MONTH/YEAR)	DAY . . . .<br>MONTH . . . .<br>YEAR	DAY . . . .<br>MONTH . . . .<br>YEAR	DAY . . . .<br>MONTH . . . .<br>YEAR . . . .
534	RECORD RESULTS OF DIPSTICK FOR HEMATURIA	POSITIVE . . . . 1<br>NEGATIVE . . . . 2<br>INVALID . . . . 3<br>REFUSED . . . . 4<br>NOT PRESENT . . . . 5<br>OTHER 6	POSITIVE . . . . 1<br>NEGATIVE . . . . 2<br>INVALID . . . . 3<br>REFUSED . . . . 4<br>NOT PRESENT . . . . 5<br>OTHER 6	POSITIVE . . . . 1<br>NEGATIVE . . . . 2<br>INVALID . . . . 3<br>REFUSED . . . . 4<br>NOT PRESENT . . . . 5<br>OTHER 6
535	**REFERRAL CLINICAL MALARIA**<br><br>CHECK 520:<br>REFER IF RDT POSITIVE (520=1)	REFERRED . . . . 1<br>NOT REFERRED 2	REFERRED . . . . 1<br>NOT REFERRED 2	REFERRED . . . . 1<br>NOT REFERRED 2
536	**REFERRAL SEVERE ANEMIA**<br><br>CHECK 521:<br>REFER IF Hb <7 G/DL	REFERRED . . . . 1<br>NOT REFERRED 2	REFERRED . . . . 1<br>NOT REFERRED 2	REFERRED . . . . 1<br>NOT REFERRED 2
537	**REFERRAL MALNUTRITION**<br><br>CHECK 527:<br>REFER IF MUAC <19.0	REFERRED . . . . 1<br>NOT REFERRED 2	REFERRED . . . . 1<br>NOT REFERRED 2	REFERRED . . . . 1<br>NOT REFERRED 2
538	**REFERRAL PRESUMED SHISTO**<br><br>CHECK 534<br>REFER IF HEMATURIA POSITIVE (534=1)	REFERRED . . . . 1<br>NOT REFERRED 2	REFERRED . . . . 1<br>NOT REFERRED 2	REFERRED . . . . 1<br>NOT REFERRED 2
539	GO BACK TO 502 IN NEXT COLUMN OF THIS QUESTIONNAIRE OR IN THE FIRST COLUMN OF AN ADDITIONAL QUESTIONNAIRE;<br>IF NO MORE MEN 20-54 YEARS, END INTERVIEW.			

FIELDWORKER'S OBSERVATIONS

TO BE FILLED IN AFTER COMPLETING INTERVIEW AND TESTING

SUPERVISOR'S OBSERVATIONS

EDITOR'S OBSERVATIONS
