# Supplementary material for: Implication of asymptomatic and clinical Plasmodium falciparum infections on biomarkers of iron status among school-aged children in Malawi
Source: Malar J. 2022 Oct 1;21:278. doi: 10.1186/s12936-022-04297-1 (PMC9526385; doi:10.1186/s12936-022-04297-1)
Supplement: Supplementary file 2 — Additional file 2. Questionnaire for the main Malawi Demographic and Health Survey. [file 12936_2022_4297_MOESM2_ESM.pdf]

FORMATTING DATE: 10 Oct 2015
ENGLISH LANGUAGE: 10 Oct 2015

2015-2016 MALAWI DEMOGRAPHIC AND HEALTH SURVEY
MALAWI GOVERNMENT - NATIONAL STATISTICAL OFFICE
HOUSEHOLD QUESTIONNAIRE

IDENTIFICATION

PLACE NAME ____________________

NAME OF HOUSEHOLD HEAD ____________________

CLUSTER NUMBER . . . . [ ][ ][ ][ ]

HOUSEHOLD NUMBER . . . . [ ][ ][ ][ ]

HOUSEHOLD SELECTED FOR MAN'S SURVEY? (1=YES, 2=NO) . . . . [ ]

HOUSEHOLD SELECTED FOR MICRONUTRIENT'S STUDY? (1=YES, 2=NO) . . . . [ ]

INTERVIEWER VISITS

	1	2	3	FINAL VISIT
DATE	________	________	________	DAY [ ][ ]<br>MONTH [ ][ ]<br>YEAR [ ][ ][ ][ ]
INTERVIEWER'S NAME	________	________	________	INT. NO. [ ][ ][ ][ ]
RESULT*	________	________	________	RESULT* [ ]
NEXT VISIT: DATE	________	________		TOTAL NUMBER OF VISITS [ ]
TIME	________	________		

*RESULT CODES:

1 COMPLETED
2 NO HOUSEHOLD MEMBER AT HOME OR NO COMPETENT RESPONDENT AT HOME AT TIME OF VISIT
3 ENTIRE HOUSEHOLD ABSENT FOR EXTENDED PERIOD OF TIME
4 POSTPONED
5 REFUSED
6 DWELLING VACANT OR ADDRESS NOT A DWELLING
7 DWELLING DESTROYED
8 DWELLING NOT FOUND
9 OTHER ____________________ (SPECIFY)

TOTAL PERSONS IN HOUSEHOLD [ ][ ]

TOTAL ELIGIBLE WOMEN [ ][ ]

TOTAL ELIGIBLE MEN [ ][ ]

LINE NO. OF RESPONDENT TO HOUSEHOLD QUESTIONNAIRE [ ][ ]

LANGUAGE OF QUESTIONNAIRE** [0][1] LANGUAGE OF INTERVIEW** [ ][ ] NATIVE LANGUAGE OF RESPONDENT** [ ][ ] TRANSLATOR USED (YES = 1, NO = 2) [ ]

LANGUAGE OF QUESTIONNAIRE** ENGLISH

**LANGUAGE CODES:
01 ENGLISH 03 TUMBUKA
02 CHICHEWA 09 OTHER ____________ (SPECIFY)

SUPERVISOR		OFFICE EDITOR	KEYED BY
____________ NAME	[ ][ ][ ][ ] NUMBER	[ ][ ] NUMBER	[ ][ ] NUMBER

THIS PAGE IS INTENTIONALLY BLANK

INTRODUCTION AND CONSENT

Hello. My name is ______________________________________. I am working with The National Statistical Office. We are conducting a survey about health and other topics all over Malawi. The information we collect will help the government to plan health services. Your household was selected for the survey. I would like to ask you some questions about your household. The questions usually take about 15 to 20 minutes. All of the answers you give will be confidential and will not be shared with anyone other than members of our survey team. You don't have to be in the survey, but we hope you will agree to answer the questions since your views are important. If I ask you any question you don't want to answer, just let me know and I will go on to the next question or you can stop the interview at any time. In case you need more information about the survey, you may contact the person listed on this card.

GIVE CARD WITH CONTACT INFORMATION

Do you have any questions?
May I begin the interview now?

SIGNATURE OF INTERVIEWER ______________________________ DATE ____________________

RESPONDENT AGREES TO BE INTERVIEWED . . 1

RESPONDENT DOES NOT AGREE TO BE INTERVIEWED . . 2 → END

100	RECORD THE TIME.	HOURS . . . . ☐☐<br>MINUTES . . . . ☐☐

HOUSEHOLD SCHEDULE

							IF AGE 15 OR OLDER			
LINE NO.	USUAL RESIDENTS AND VISITORS	RELATIONSHIP TO HEAD OF HOUSEHOLD	SEX	RESIDENCE		AGE	MARITAL STATUS	ELIGIBILITY		
**1**	**2**	**3**	**4**	**5**	**6**	**7**	**8**	**9**	**10**	**11**
	Please give me the names of the persons who usually live in your household and guests of the household who stayed here last night, starting with the head of the household.	What is the relationship of (NAME) to the head of the household?	Is (NAME) male or female?	Does (NAME) usually live here?	Did (NAME) stay here last night?	How old is (NAME)?	What is (NAME)'s current marital status?	CIRCLE LINE NUMBER OF ALL WOMEN AGE 15-49	**IF HOUSE-HOLD SELEC-TED FOR MAN'S SURVEY**	**IF HOUSE-HOLD SELEC-TED FOR MAN'S SURVEY**
	AFTER LISTING THE NAMES AND RECORDING THE RELATIONSHIP AND SEX FOR EACH PERSON, ASK QUESTIONS 2A-2C TO BE SURE THAT THE LISTING IS COMPLETE.<br><br>THEN ASK APPROPRIATE QUESTIONS IN COLUMNS 5-20 FOR EACH PERSON.	SEE CODES BELOW.				IF 95 OR MORE, RECORD '95'.	1 = MARRIED OR LIVING TOGETHER<br>2 = DIVORCED/ SEPARATED<br>3 = WIDOWED<br>4 = NEVER-MARRIED AND NEVER LIVED TOGETHER		CIRCLE LINE NUMBER OF ALL MEN AGE 15-54	CIRCLE LINE NUMBER OF ALL CHILDREN AGE 0-5
			M F	Y N	Y N	IN YEARS				
01			1 2	1 2	1 2			01	01	01
02			1 2	1 2	1 2			02	02	02
03			1 2	1 2	1 2			03	03	03
04			1 2	1 2	1 2			04	04	04
05			1 2	1 2	1 2			05	05	05
06			1 2	1 2	1 2			06	06	06
07			1 2	1 2	1 2			07	07	07
08			1 2	1 2	1 2			08	08	08
09			1 2	1 2	1 2			09	09	09
10			1 2	1 2	1 2			10	10	10

2A)	Just to make sure that I have a complete listing: are there any other people such as small children or infants that we have not listed?	YES → ADD TO TABLE	NO
2B)	Are there any other people who may not be members of your family, such as domestic servants, lodgers, or friends who usually live here?	YES → ADD TO TABLE	NO
2C)	Are there any guests or temporary visitors staying here, or anyone else who stayed here last night, who have not been listed?	YES → ADD TO TABLE	NO

CODES FOR Q. 3: RELATIONSHIP TO HEAD OF HOUSEHOLD

01 = HEAD
02 = WIFE OR HUSBAND
03 = SON OR DAUGHTER
04 = SON-IN-LAW OR DAUGHTER-IN-LAW
05 = GRANDCHILD
06 = PARENT
07 = PARENT-IN-LAW
08 = BROTHER OR SISTER
09 = OTHER RELATIVE
10 = ADOPTED/FOSTER/ STEPCHILD
11 = NOT RELATED
98 = DON'T KNOW

	IF AGE 0-17 YEARS				IF AGE 5 YEARS OR OLDER		IF AGE 5-24 YEARS		IF AGE 0-4 YEARS	
LINE NO.	SURVIVORSHIP AND RESIDENCE OF BIOLOGICAL PARENTS				EVER ATTENDED SCHOOL		CURRENT/RECENT SCHOOL ATTENDANCE		BIRTH REGISTRATION	
	12	13	14	15	16	17	18	19	20	21
	Is (NAME)'s natural mother alive?	Does (NAME)'s natural mother usually live in this household or was she a guest last night?<br><br>IF YES: What is her name?<br><br>RECORD MOTHER'S LINE NUMBER.<br><br>IF NO, RECORD '00'.	Is (NAME)'s natural father alive?	Does (NAME)'s natural father usually live in this household or was he a guest last night?<br><br>IF YES: What is his name?<br><br>RECORD FATHER'S LINE NUMBER.<br><br>IF NO, RECORD '00'.	Has (NAME) ever attended school?	What is the highest level of school (NAME) has attended?<br><br>What is the highest grade (NAME) completed at that level?<br><br>SEE CODES BELOW.	Did (NAME) attend school at any time during the [2015-2016] school year?	During [this/that] school year, what level and grade [is/was] (NAME) attending?<br><br>SEE CODES BELOW.	Does (NAME) have a birth certificate?<br><br>IF NO, PROBE: Has (NAME)'s birth ever been registered with the civil authority?<br><br>1 = HAS CERTIFICATE<br>2 = REGISTERED<br>3 = NEITHER<br>8 = DON'T KNOW	IF Q.20=1 OR Q.20=2<br><br>Was (NAME)'s birth registered with the district commissioner, hospital, registrar general's office or the traditional village chief?<br><br>1= DISTRICT COMMISSIONER<br>2= HOSPITAL<br>3= REGISTRAR GENERAL<br>4= TRADITIONAL VILLAGE CHIEF<br>6=OTHER
01	Y N DK<br>1 2 8<br>GO TO 14		Y N DK<br>1 2 8<br>GO TO 16		Y N<br>1 2<br>NEXT LINE	LEVEL GRADE	Y N<br>1 2<br>NEXT LINE	LEVEL GRADE		
02	1 2 8<br>GO TO 14		1 2 8<br>GO TO 16		1 2<br>NEXT LINE		1 2<br>NEXT LINE			
03	1 2 8<br>GO TO 14		1 2 8<br>GO TO 16		1 2<br>NEXT LINE		1 2<br>NEXT LINE			
04	1 2 8<br>GO TO 14		1 2 8<br>GO TO 16		1 2<br>NEXT LINE		1 2<br>NEXT LINE			
05	1 2 8<br>GO TO 14		1 2 8<br>GO TO 16		1 2<br>NEXT LINE		1 2<br>NEXT LINE			
06	1 2 8<br>GO TO 14		1 2 8<br>GO TO 16		1 2<br>NEXT LINE		1 2<br>NEXT LINE			
07	1 2 8<br>GO TO 14		1 2 8<br>GO TO 16		1 2<br>NEXT LINE		1 2<br>NEXT LINE			
08	1 2 8<br>GO TO 14		1 2 8<br>GO TO 16		1 2<br>NEXT LINE		1 2<br>NEXT LINE			
09	1 2 8<br>GO TO 14		1 2 8<br>GO TO 16		1 2<br>NEXT LINE		1 2<br>NEXT LINE			
10	1 2 8<br>GO TO 14		1 2 8<br>GO TO 16		1 2<br>NEXT LINE		1 2<br>NEXT LINE			

CODES FOR Qs. 17 AND 19: EDUCATION

LEVEL	GRADE
0 = PRESCHOOL	00 = LESS THAN 1 YEAR COMPLETED (USE '00' FOR Q. 17 ONLY. THIS CODE IS NOT ALLOWED FOR Q. 19.)
1 = PRIMARY	98 = DON'T KNOW
2 = SECONDARY	
3 = HIGHER	
8 = DON'T KNOW	

HOUSEHOLD SCHEDULE

							IF AGE 15 OR OLDER			
LINE NO.	USUAL RESIDENTS AND VISITORS	RELATIONSHIP TO HEAD OF HOUSEHOLD	SEX	RESIDENCE		AGE	MARITAL STATUS	ELIGIBILITY		
1	2	3	4	5	6	7	8	9	10	11
	Please give me the names of the persons who usually live in your household and guests of the household who stayed here last night, starting with the head of the household.<br><br>AFTER LISTING THE NAMES AND RECORDING THE RELATIONSHIP AND SEX FOR EACH PERSON, ASK QUESTIONS 2A-2C TO BE SURE THAT THE LISTING IS COMPLETE.<br><br>THEN ASK APPROPRIATE QUESTIONS IN COLUMNS 5-20 FOR EACH PERSON.	What is the relationship of (NAME) to the head of the household?<br><br>SEE CODES BELOW.	Is (NAME) male or female?	Does (NAME) usually live here?	Did (NAME) stay here last night?	How old is (NAME)?<br><br>IF 95 OR MORE, RECORD '95'.	What is (NAME)'s current marital status?<br><br>1 = MARRIED OR LIVING TOGETHER<br>2 = DIVORCED/ SEPARATED<br>3 = WIDOWED<br>4 = NEVER-MARRIED AND NEVER LIVED TOGETHER	CIRCLE LINE NUMBER OF ALL WOMEN AGE 15-49	**IF HOUSE-HOLD SELEC-TED FOR MAN'S SURVEY**<br><br>CIRCLE LINE NUMBER OF ALL MEN AGE 15-54	**IF HOUSE-HOLD SELEC-TED FOR MAN'S SURVEY**<br><br>CIRCLE LINE NUMBER OF ALL CHILDREN AGE 0-5
			M F	Y N	Y N	IN YEARS				
11		☐☐	1 2	1 2	1 2	☐☐	☐	11	11	11
12		☐☐	1 2	1 2	1 2	☐☐	☐	12	12	12
13		☐☐	1 2	1 2	1 2	☐☐	☐	13	13	13
14		☐☐	1 2	1 2	1 2	☐☐	☐	14	14	14
15		☐☐	1 2	1 2	1 2	☐☐	☐	15	15	15
16		☐☐	1 2	1 2	1 2	☐☐	☐	16	16	16
17		☐☐	1 2	1 2	1 2	☐☐	☐	17	17	17
18		☐☐	1 2	1 2	1 2	☐☐	☐	18	18	18
19		☐☐	1 2	1 2	1 2	☐☐	☐	19	19	19
20		☐☐	1 2	1 2	1 2	☐☐	☐	20	20	20

TICK HERE IF CONTINUATION SHEET USED ☐

CODES FOR Q. 3: RELATIONSHIP TO HEAD OF HOUSEHOLD

01 = HEAD
02 = WIFE OR HUSBAND
03 = SON OR DAUGHTER
04 = SON-IN-LAW OR DAUGHTER-IN-LAW
05 = GRANDCHILD
06 = PARENT
07 = PARENT-IN-LAW
08 = BROTHER OR SISTER
09 = OTHER RELATIVE
10 = ADOPTED/FOSTER/ STEPCHILD
11 = NOT RELATED
98 = DON'T KNOW

HOUSEHOLD SCHEDULE

LINE NO.	IF AGE 0-17 YEARS: SURVIVORSHIP AND RESIDENCE OF BIOLOGICAL PARENTS				IF AGE 5 YEARS OR OLDER: EVER ATTENDED SCHOOL		IF AGE 5-24 YEARS: CURRENT/RECENT SCHOOL ATTENDANCE		IF AGE 0-4 YEARS: BIRTH REGISTRATION	
	12	13	14	15	16	17	18	19	20	21
	Is (NAME)'s natural mother alive?	Does (NAME)'s natural mother usually live in this household or was she a guest last night?<br>IF YES: What is her name?<br>RECORD MOTHER'S LINE NUMBER.<br>IF NO, RECORD '00'.	Is (NAME)'s natural father alive?	Does (NAME)'s natural father usually live in this household or was he a guest last night?<br>IF YES: What is his name?<br>RECORD FATHER'S LINE NUMBER.<br>IF NO, RECORD '00'.	Has (NAME) ever attended school?	What is the highest level of school (NAME) has attended?<br>What is the highest grade (NAME) completed at that level?<br>SEE CODES BELOW.	Did (NAME) attend school at any time during the [2015-2016] school year?	During [this/that] school year, what level and grade [is/was] (NAME) attending?<br>SEE CODES BELOW.	Does (NAME) have a birth certificate?<br>IF NO, PROBE: Has (NAME)'s birth ever been registered with the civil authority?<br>1 = HAS CERTIFICATE<br>2 = REGISTERED<br>3 = NEITHER<br>8 = DON'T KNOW	IF Q.20=1 OR Q.20=2<br>Was (NAME)'s birth registered with the district commissioner, hospital, registrar general's office or the traditional village chief?<br>1= DISTRICT COMMISSIONER<br>2= HOSPITAL<br>3= REGISTRAR GENERAL<br>4= TRADITIONAL VILLAGE CHIEF<br>6=OTHER
11	Y N DK<br>1 2 8<br>GO TO 14	☐☐	Y N DK<br>1 2 8<br>GO TO 16	☐☐	Y N<br>1 2<br>NEXT LINE	LEVEL ☐ GRADE ☐☐	Y N<br>1 2<br>NEXT LINE	LEVEL ☐ GRADE ☐☐	☐	☐
12	1 2 8<br>GO TO 14	☐☐	1 2 8<br>GO TO 16	☐☐	1 2<br>NEXT LINE	☐ ☐☐	1 2<br>NEXT LINE	☐ ☐☐	☐	☐
13	1 2 8<br>GO TO 14	☐☐	1 2 8<br>GO TO 16	☐☐	1 2<br>NEXT LINE	☐ ☐☐	1 2<br>NEXT LINE	☐ ☐☐	☐	☐
14	1 2 8<br>GO TO 14	☐☐	1 2 8<br>GO TO 16	☐☐	1 2<br>NEXT LINE	☐ ☐☐	1 2<br>NEXT LINE	☐ ☐☐	☐	☐
15	1 2 8<br>GO TO 14	☐☐	1 2 8<br>GO TO 16	☐☐	1 2<br>NEXT LINE	☐ ☐☐	1 2<br>NEXT LINE	☐ ☐☐	☐	☐
16	1 2 8<br>GO TO 14	☐☐	1 2 8<br>GO TO 16	☐☐	1 2<br>NEXT LINE	☐ ☐☐	1 2<br>NEXT LINE	☐ ☐☐	☐	☐
17	1 2 8<br>GO TO 14	☐☐	1 2 8<br>GO TO 16	☐☐	1 2<br>NEXT LINE	☐ ☐☐	1 2<br>NEXT LINE	☐ ☐☐	☐	☐
18	1 2 8<br>GO TO 14	☐☐	1 2 8<br>GO TO 16	☐☐	1 2<br>NEXT LINE	☐ ☐☐	1 2<br>NEXT LINE	☐ ☐☐	☐	☐
19	1 2 8<br>GO TO 14	☐☐	1 2 8<br>GO TO 16	☐☐	1 2<br>NEXT LINE	☐ ☐☐	1 2<br>NEXT LINE	☐ ☐☐	☐	☐
20	1 2 8<br>GO TO 14	☐☐	1 2 8<br>GO TO 16	☐☐	1 2<br>NEXT LINE	☐ ☐☐	1 2<br>NEXT LINE	☐ ☐☐	☐	☐

CODES FOR Qs. 17 AND 19: EDUCATION

LEVEL	GRADE
0 = PRESCHOOL	00 = LESS THAN 1 YEAR COMPLETED (USE '00' FOR Q. 17 ONLY. THIS CODE IS NOT ALLOWED FOR Q. 19.)
1 = PRIMARY	98 = DON'T KNOW
2 = SECONDARY	
3 = HIGHER	
8 = DON'T KNOW	

SELECTION OF WOMAN FOR THE DOMESTIC VIOLENCE QUESTIONS

CHECK FRONT COVER

HOUSEHOLD SELECTED FOR MAN'S SURVEY ☐ ↓

HOUSEHOLD NOT SELECTED FOR MAN'S SURVEY ☐ → 101

LOOK AT THE LAST DIGIT OF THE HOUSEHOLD QUESTIONNAIRE SERIAL NUMBER ON THE COVER PAGE. THIS IS THE ROW NUMBER YOU SHOULD GO TO. CHECK THE TOTAL NUMBER OF ELIGIBLE WOMEN (COLUMN 9) IN THE HOUSEHOLD SCHEDULE. THIS IS THE COLUMN NUMBER YOU SHOULD GO TO. FOLLOW THE SELECTED ROW AND COLUMN TO THE CELL WHERE THEY MEET AND CIRCLE THE NUMBER IN THE CELL. THIS IS THE NUMBER OF THE WOMAN SELECTED FOR THE DOMESTIC VIOLENCE QUESTIONS FROM THE LIST OF ELIGIBLE WOMEN IN COLUMN 9 OF THE HOUSEHOLD SCHEDULE. WRITE THE NAME AND LINE NUMBER OF THE SELECTED WOMAN IN THE SPACE BELOW THE TABLE.

EXAMPLE: THE HOUSEHOLD QUESTIONNAIRE SERIAL NUMBER IS '716' AND THE HOUSEHOLD SCHEDULE COLUMN 9 SHOWS THAT THERE ARE THREE ELIGIBLE WOMEN AGE 15-49 IN THE HOUSEHOLD (LINE NUMBERS 02, 04, AND 05). SINCE THE LAST DIGIT OF THE HOUSEHOLD SERIAL NUMBER IS '6' GO TO ROW '6' AND SINCE THERE ARE THREE ELIGIBLE WOMEN IN THE HOUSEHOLD, GO TO COLUMN '3'. FOLLOW THE ROW AND COLUMN AND FIND THE NUMBER IN THE CELL WHERE THEY MEET ('2') AND CIRCLE THE NUMBER. NOW GO TO THE HOUSEHOLD SCHEDULE AND FIND THE SECOND WOMAN WHO IS ELIGIBLE FOR THE WOMAN'S INTERVIEW (LINE NUMBER '04' IN THIS EXAMPLE). WRITE HER NAME AND LINE NUMBER IN

LAST DIGIT OF THE HOUSE-HOLD QUESTION-NAIRE SERIAL NUMBER	TOTAL NUMBER OF ELIGIBLE WOMEN AGE 15-49 IN HOUSEHOLD SCHEDULE COLUMN 9							
	1	2	3	4	5	6	7	8
0	1	2	2	4	3	6	5	4
1	1	1	3	1	4	1	6	5
2	1	2	1	2	5	2	7	6
3	1	1	2	3	1	3	1	7
4	1	2	3	4	2	4	2	8
5	1	1	1	1	3	5	3	1
6	1	2	2	2	4	6	4	2
7	1	1	3	3	5	1	5	3
8	1	2	1	4	1	2	6	4
9	1	1	2	1	2	3	7	5

30 NAME OF SELECTED WOMAN ________________

HH LINE NUMBER OF SELECTED WOMAN ☐☐

NO.	QUESTIONS AND FILTERS	CODING CATEGORIES	SKIP
101	What is the main source of drinking water for members of your household?	**PIPED WATER**<br>PIPED INTO DWELLING . . . . 11<br>PIPED TO YARD/PLOT . . . . 12<br>PIPED TO NEIGHBOR . . . . 13<br>PUBLIC TAP/STANDPIPE . . . . 14<br><br>TUBE WELL OR BOREHOLE . . . . 21<br>**DUG WELL**<br>PROTECTED WELL . . . . 31<br>UNPROTECTED WELL . . . . 32<br>**WATER FROM SPRING**<br>PROTECTED SPRING . . . . 41<br>UNPROTECTED SPRING . . . . 42<br><br>RAINWATER . . . . 51<br>TANKER TRUCK . . . . 61<br>CART WITH SMALL TANK . . . . 71<br>SURFACE WATER (RIVER/DAM/<br>LAKE/POND/STREAM/CANAL/<br>IRRIGATION CHANNEL) . . . . 81<br>BOTTLED WATER . . . . 91<br><br>OTHER ______________________ 96<br>(SPECIFY)	11–13 → 106<br>14–81 → 103<br>96 → 103
102	What is the main source of water used by your household for other purposes such as cooking and handwashing?	**PIPED WATER**<br>PIPED INTO DWELLING . . . . 11<br>PIPED TO YARD/PLOT . . . . 12<br>PIPED TO NEIGHBOR . . . . 13<br>PUBLIC TAP/STANDPIPE . . . . 14<br><br>TUBE WELL OR BOREHOLE . . . . 21<br>**DUG WELL**<br>PROTECTED WELL . . . . 31<br>UNPROTECTED WELL . . . . 32<br>**WATER FROM SPRING**<br>PROTECTED SPRING . . . . 41<br>UNPROTECTED SPRING . . . . 42<br><br>RAINWATER . . . . 51<br>TANKER TRUCK . . . . 61<br>CART WITH SMALL TANK . . . . 71<br>SURFACE WATER (RIVER/DAM/<br>LAKE/POND/STREAM/CANAL/<br>IRRIGATION CHANNEL) . . . . 81<br><br>OTHER ______________________ 96<br>(SPECIFY)	11–13 → 106
103	Where is that water source located?	IN OWN DWELLING . . . . 1<br>IN OWN YARD/PLOT . . . . 2<br>ELSEWHERE 3	1–2 → 105
104	How long does it take to go there, get water, and come back?	MINUTES . . . . [ ][ ][ ]<br><br>DON'T KNOW 998	
105	CHECK 101 AND 102: CODE '14' OR '21' CIRCLED?<br>YES [ ] ↓ NO [ ]		NO → 107

HOUSEHOLD CHARACTERISTICS

NO.	QUESTIONS AND FILTERS	CODING CATEGORIES	SKIP
106	In the past two weeks, was the water from this source not available for at least one full day?	YES . . . 1<br>NO . . . 2<br>DON'T KNOW . . . 8	
107	Do you do anything to the water to make it safer to drink?	YES . . . 1<br>NO . . . 2<br>DON'T KNOW . . . 8	(NO, DON'T KNOW) → 109
108	What do you usually do to make the water safer to drink?<br><br>Anything else?<br><br>RECORD ALL MENTIONED.	BOIL . . . A<br>ADD BLEACH/CHLORINE . . . B<br>STRAIN THROUGH A CLOTH . . . C<br>USE WATER FILTER (CERAMIC/ SAND/COMPOSITE/ETC) . . . D<br>SOLAR DISINFECTION . . . E<br>LET IT STAND AND SETTLE . . . F<br>OTHER ______ (SPECIFY) X<br>DON'T KNOW . . . Z	
109	What kind of toilet facility do members of your household usually use?<br><br>IF NOT POSSIBLE TO DETERMINE, ASK PERMISSION TO OBSERVE THE FACILITY.	**FLUSH OR POUR FLUSH TOILET**<br>FLUSH TO PIPED SEWER SYSTEM . . . 11<br>FLUSH TO SEPTIC TANK . . . 12<br>FLUSH TO PIT LATRINE . . . 13<br>FLUSH TO SOMEWHERE ELSE . . . 14<br>FLUSH, DON'T KNOW WHERE . . . 15<br>**PIT LATRINE**<br>VENTILATED IMPROVED PIT LATRINE . . . 21<br>PIT LATRINE WITH SLAB . . . 22<br>PIT LATRINE WITHOUT SLAB/OPEN PIT . . . 23<br><br>COMPOSTING TOILET . . . 31<br>BUCKET TOILET . . . 41<br>HANGING TOILET/HANGING LATRINE . . . 51<br>NO FACILITY/BUSH/FIELD . . . 61<br><br>OTHER ______ (SPECIFY) 96	(NO FACILITY) → 113
110	Do you share this toilet facility with other households?	YES . . . 1<br>NO . . . 2	(NO) → 112
111	Including your own household, how many households use this toilet facility?	NO. OF HOUSEHOLDS IF LESS THAN 10 . . . [0][ ]<br><br>10 OR MORE HOUSEHOLDS . . . 95<br>DON'T KNOW . . . 98	
112	Where is this toilet facility located?	IN OWN DWELLING . . . 1<br>IN OWN YARD/PLOT . . . 2<br>ELSEWHERE . . . 3	

HOUSEHOLD CHARACTERISTICS

NO.	QUESTIONS AND FILTERS	CODING CATEGORIES	SKIP
113	What type of fuel does your household mainly use for cooking?	ELECTRICITY . . . 01<br>LPG . . . 02<br>NATURAL GAS . . . 03<br>BIOGAS . . . 04<br>KEROSENE . . . 05<br>COAL, LIGNITE . . . 06<br>CHARCOAL . . . 07<br>WOOD . . . 08<br>STRAW/SHRUBS/GRASS . . . 09<br>AGRICULTURAL CROP . . . 10<br>ANIMAL DUNG . . . 11<br><br>NO FOOD COOKED IN HOUSEHOLD . . . 95<br><br>OTHER ______ 96<br>(SPECIFY)	 <br><br><br><br><br><br><br><br><br><br><br>→ 116
114	Is the cooking usually done in the house, in a separate building, or outdoors?	IN THE HOUSE . . . 1<br>IN A SEPARATE BUILDING . . . 2<br>OUTDOORS . . . 3<br><br>OTHER ______ 6<br>(SPECIFY)	2, 3, 6 → 116
115	Do you have a separate room which is used as a kitchen?	YES . . . 1<br>NO . . . 2	
116	How many rooms in this household are used for sleeping?	ROOMS . . . [ ][ ]	
117	Does this household own any livestock, herds, other farm animals, or poultry?	YES . . . 1<br>NO . . . 2	 → 119
118	How many of the following animals does this household own?<br>IF NONE, RECORD '00'.<br>IF 95 OR MORE, RECORD '95'.<br>IF UNKNOWN, RECORD '98'.<br><br>a) Milk cows or bulls?<br>b) Other cattle?<br>c) Donkeys, or mules?<br>d) Goats?<br>e) Sheep?<br>f) Pigs?<br>g) Chickens?<br>h) Other poultry?	 <br><br><br><br><br>a) COWS/BULLS . . . [ ][ ]<br>b) OTHER CATTLE . . . [ ][ ]<br>c) DONKEYS/MULES . . . [ ][ ]<br>d) GOATS . . . [ ][ ]<br>e) SHEEP . . . [ ][ ]<br>f) PIGS . . . [ ][ ]<br>g) CHICKENS . . . [ ][ ]<br>h) POULTRY . . . [ ][ ]	
119	Does any member of this household own any agricultural land?	YES . . . 1<br>NO . . . 2	 → 121
120	How many hectares of agricultural land do members of this household own?<br><br>IF 95 OR MORE, CIRCLE '950'.	HECTARES . . . [ ][ ].[ ]<br><br>95 OR MORE HECTARES . . . 950<br>DON'T KNOW . . . 998	

HOUSEHOLD CHARACTERISTICS

NO.	QUESTIONS AND FILTERS	CODING CATEGORIES	SKIP
121	Does your household have:<br>a) Electricity?<br>b) A radio?<br>c) A television?<br>d) A non-mobile telephone?<br>e) A computer?<br>f) A refrigerator?<br>g) Koloboyi?<br>h) A paraffin lamp?<br>i) A torch?<br>j) A bed with a matress?<br>k) A sofa set?	YES NO<br>a) ELECTRICITY .... 1 2<br>b) RADIO .... 1 2<br>c) TELEVISION .... 1 2<br>d) NON-MOBILE TELEPHONE .. 1 2<br>e) COMPUTER .... 1 2<br>f) REFRIGERATOR .... 1 2<br>g) KOLOBOYI .. 1 2<br>h) PARAFFIN LAMP .... 1 2<br>i) TORCH .... 1 2<br>j) BED WITH MAT .... 1 2<br>k) SOFA SET 1 2	
122	Does any member of this household own:<br>a) A wrist watch?<br>b) A mobile phone?<br>c) A bicycle?<br>d) A motorcycle or motor scooter?<br>e) An animal-drawn cart?<br>f) A car or truck?<br>g) A boat with a motor?	YES NO<br>a) WATCH .... 1 2<br>b) MOBILE PHONE .... 1 2<br>c) BICYCLE .... 1 2<br>d) MOTORCYCLE/SCOOTER ..... 1 2<br>e) ANIMAL-DRAWN CART ..... 1 2<br>f) CAR/TRUCK .... 1 2<br>g) BOAT WITH MOTOR 1 2	
123	Does any member of this household have a bank account?	YES .... 1<br>NO 2	
124	How often does anyone smoke inside your house? Would you say daily, weekly, monthly, less often than once a month, or never?	DAILY .... 1<br>WEEKLY .... 2<br>MONTHLY .... 3<br>LESS OFTEN THAN ONCE A MONTH .... 4<br>NEVER 5	
125	At any time in the past 12 months, has anyone come into your dwelling to spray the interior walls against mosquitoes?	YES .... 1<br>NO .... 2<br>DON'T KNOW 8	 2, 8 → 127
126	Who sprayed the dwelling?	GOVERNMENT WORKER/PROGRAM .... A<br>PRIVATE COMPANY .... B<br>NONGOVERNMENTAL ORGANIZATION (NGO) .. C<br>OTHER ______________________ X<br>(SPECIFY)<br>DON'T KNOW .... Z	
127	Does your household have any mosquito nets?	YES .... 1<br>NO 2	 → 139
128	How many mosquito nets does your household have?<br>IF 7 OR MORE NETS, RECORD '7'.	NUMBER OF NETS .... [ ]	

		NET #1	NET #2	NET #3
129	ASK THE RESPONDENT TO SHOW YOU ALL THE NETS IN THE HOUSEHOLD.<br><br>IF MORE THAN 3 NETS, USE ADDITIONAL QUESTIONNAIRE(S).	OBSERVED . . . . 1<br>NOT OBSERVED 2	OBSERVED . . . . 1<br>NOT OBSERVED 2	OBSERVED . . . . 1<br>NOT OBSERVED 2
129A	Is the net hanging for sleeping?	YES . . . . 1<br>NO 2	YES . . . . 1<br>NO 2	YES . . . . 1<br>NO 2
130	How many months ago did your household get the mosquito net?<br><br>IF LESS THAN ONE MONTH AGO, RECORD '00'.	MONTHS AGO . . . . . [ ][ ]<br>MORE THAN 36 MONTHS AGO . . . . . 95<br>NOT SURE 98	MONTHS AGO . . . . . [ ][ ]<br>MORE THAN 36 MONTHS AGO . . . . . 95<br>NOT SURE 98	MONTHS AGO . . . . . [ ][ ]<br>MORE THAN 36 MONTHS AGO . . . . . 95<br>NOT SURE 98
131	OBSERVE OR ASK BRAND/TYPE OF MOSQUITO NET.<br><br>IF BRAND IS UNKNOWN AND YOU CANNOT OBSERVE THE NET, SHOW PICTURES OF TYPICAL NET TYPES/BRANDS TO RESPONDENT.	**LONG-LASTING INSECTICIDE-TREATED NET (LLIN)**<br>DAWAPLUS . . . . 11<br>DURANET . . . . 12<br>INTERCEPTOR . . . . . 13<br>LIFENET . . . . 14<br>MAGNET . . . . 15<br>OLYSET . . . . 16<br>OLYSET PLUS . . . . . 17<br>PERMANET 2.0 . . . . . 18<br>PERMANET 3.0 . . . . . 19<br>ROYAL SENTRY . . . . . 20<br>YORKOOL . . . . 21<br>OTHER/DON'T KNOW BRAND . . . . 26<br>(SKIP TO 134)<br>OTHER TYPE . . . . 96<br>DON'T KNOW TYPE . . 98	**LONG-LASTING INSECTICIDE-TREATED NET (LLIN)**<br>DAWAPLUS . . . . 11<br>DURANET . . . . 12<br>INTERCEPTOR . . . . . 13<br>LIFENET . . . . 14<br>MAGNET . . . . 15<br>OLYSET . . . . 16<br>OLYSET PLUS . . . . . 17<br>PERMANET 2.0 . . . . . 18<br>PERMANET 3.0 . . . . . 19<br>ROYAL SENTRY . . . . . 20<br>YORKOOL . . . . 21<br>OTHER/DON'T KNOW BRAND . . . . 26<br>(SKIP TO 134)<br>OTHER TYPE . . . . 96<br>DON'T KNOW TYPE . . 98	**LONG-LASTING INSECTICIDE-TREATED NET (LLIN)**<br>DAWAPLUS . . . . 11<br>DURANET . . . . 12<br>INTERCEPTOR . . . . . 13<br>LIFENET . . . . 14<br>MAGNET . . . . 15<br>OLYSET . . . . 16<br>OLYSET PLUS . . . . . 17<br>PERMANET 2.0 . . . . . 18<br>PERMANET 3.0 . . . . . 19<br>ROYAL SENTRY . . . . . 20<br>YORKOOL . . . . 21<br>OTHER/DON'T KNOW BRAND . . . . 26<br>(SKIP TO 134)<br>OTHER TYPE . . . . 96<br>DON'T KNOW TYPE . . 98
132	Since you got the net, was it ever soaked or dipped in a liquid to kill or repel mosquitoes?	YES . . . . 1<br>NO . . . . 2<br>(SKIP TO 134)<br>NOT SURE 8	YES . . . . 1<br>NO . . . . 2<br>(SKIP TO 134)<br>NOT SURE 8	YES . . . . 1<br>NO . . . . 2<br>(SKIP TO 134)<br>NOT SURE 8
133	How many months ago was the net last soaked or dipped?<br><br>IF LESS THAN ONE MONTH AGO, RECORD '00'.	MONTHS AGO . . . . . [ ][ ]<br>MORE THAN 24 MONTHS AGO . . . . . 95<br>NOT SURE 98	MONTHS AGO . . . . . [ ][ ]<br>MORE THAN 24 MONTHS AGO . . . . . 95<br>NOT SURE 98	MONTHS AGO . . . . . [ ][ ]<br>MORE THAN 24 MONTHS AGO . . . . . 95<br>NOT SURE 98
134	Did you get the net through the 2014-2015 mass campaign, during an antenatal care visit, at birth, or first immunization visit?	YES, 2014-2015 MASS CAMPAIGN . . . . 1<br>YES, ANC . . . . 2<br>YES, AT BIRTH . . . . 3<br>YES, IMMUNIZATION VISIT . . . . 4<br>(SKIP TO 136)<br>NO 5	YES, 2014-2015 MASS CAMPAIGN . . . . 1<br>YES, ANC . . . . 2<br>YES, AT BIRTH . . . . 3<br>YES, IMMUNIZATION VISIT . . . . 4<br>(SKIP TO 136)<br>NO 5	YES, 2014-2015 MASS CAMPAIGN . . . . 1<br>YES, ANC . . . . 2<br>YES, AT BIRTH . . . . 3<br>YES, IMMUNIZATION VISIT . . . . 4<br>(SKIP TO 136)<br>NO 5
135	Where did you get the net?	GOVERNMENT HOSPITAL 01<br>GOVERNMENT HEALTH CENTER . . . . 02<br>GOVERNMENT HEALTH POST/OUTREAC . . . . . 03<br>CHAM/MISSION . . . . 04<br>PRIVATE HEALTH FACILITY . . . . 05<br>PHARMACY . . . . 06<br>SHOP/MARKET . . . . 07<br>WORKPLACE . . . . 08<br>OTHER ______ (SPECIFY) 96<br>DON'T KNOW 98	GOVERNMENT HOSPITAL 01<br>GOVERNMENT HEALTH CENTER . . . . 02<br>GOVERNMENT HEALTH POST/OUTREAC . . . . . 03<br>CHAM/MISSION . . . . 04<br>PRIVATE HEALTH FACILITY . . . . 05<br>PHARMACY . . . . 06<br>SHOP/MARKET . . . . 07<br>WORKPLACE . . . . 08<br>OTHER ______ (SPECIFY) 96<br>DON'T KNOW 98	GOVERNMENT HOSPITAL 01<br>GOVERNMENT HEALTH CENTER . . . . 02<br>GOVERNMENT HEALTH POST/OUTREAC . . . . . 03<br>CHAM/MISSION . . . . 04<br>PRIVATE HEALTH FACILITY . . . . 05<br>PHARMACY . . . . 06<br>SHOP/MARKET . . . . 07<br>WORKPLACE . . . . 08<br>OTHER ______ (SPECIFY) 96<br>DON'T KNOW 98

		NET #1	NET #2	NET #3
136	Did anyone sleep under this mosquito net last night?	YES . . . . 1<br>NO . . . . 2<br>(SKIP TO 138)<br>NOT SURE 8	YES . . . . 1<br>NO . . . . 2<br>(SKIP TO 138)<br>NOT SURE 8	YES . . . . 1<br>NO . . . . 2<br>(SKIP TO 138)<br>NOT SURE 8
137	Who slept under this mosquito net last night?<br><br>RECORD THE PERSON'S NAME AND LINE NUMBER FROM HOUSEHOLD SCHEDULE.	NAME ____<br>LINE NO. . . . . .<br>NAME ____<br>LINE NO. . . . . .<br>NAME ____<br>LINE NO. . . . . .<br>NAME ____<br>LINE NO.	NAME ____<br>LINE NO. . . . . .<br>NAME ____<br>LINE NO. . . . . .<br>NAME ____<br>LINE NO. . . . . .<br>NAME ____<br>LINE NO.	NAME ____<br>LINE NO. . . . . .<br>NAME ____<br>LINE NO. . . . . .<br>NAME ____<br>LINE NO. . . . . .<br>NAME ____<br>LINE NO. . . . . .
138		GO BACK TO 129 FOR NEXT NET; OR, IF NO MORE NETS, GO TO 139.	GO BACK TO 129 FOR NEXT NET; OR, IF NO MORE NETS, GO TO 139.	GO TO 129 IN FIRST COLUMN OF A NEW QUESTIONNAIRE; OR, IF NO MORE NETS, GO TO 139.

ADDITIONAL HOUSEHOLD CHARACTERISTICS

NO.	QUESTIONS AND FILTERS	CODING CATEGORIES	SKIP
139	We would like to learn about the places that households use to wash their hands. Can you please show me where members of your household most often wash their hands?	OBSERVED, FIXED PLACE . . . . 1<br>OBSERVED, MOBILE . . . . 2<br>NOT OBSERVED,<br>NOT IN DWELLING/YARD/PLOT . . . . 3<br>NOT OBSERVED, NO PERMISSION TO SEE . . . . . 4<br>NOT OBSERVED, OTHER REASON 5	3, 4, 5 → 142
140	OBSERVE PRESENCE OF WATER AT THE PLACE FOR HANDWASHING.<br><br>RECORD OBSERVATION.	WATER IS AVAILABLE . . . . 1<br>WATER IS NOT AVAILABLE 2	
141	OBSERVE PRESENCE OF SOAP, DETERGENT, OR OTHER CLEANSING AGENT AT THE PLACE FOR HANDWASHING.<br><br>RECORD OBSERVATION.	SOAP OR DETERGENT<br>(BAR, LIQUID, POWDER, PASTE) . . . . A<br>ASH, MUD, SAND . . . . B<br><br>NONE C	
142	OBSERVE MAIN MATERIAL OF THE FLOOR OF THE DWELLING.<br><br>RECORD OBSERVATION.	**NATURAL FLOOR**<br>EARTH/SAND . . . . 11<br>DUNG . . . . 12<br>**RUDIMENTARY FLOOR**<br>WOOD PLANKS . . . . 21<br>PALM/BAMBOO . . . . 22<br>**FINISHED FLOOR**<br>PARQUET OR POLISHED WOOD . . . . 31<br>VINYL OR ASPHALT STRIPS . . . . 32<br>CERAMIC TILES . . . . 33<br>CEMENT . . . . 34<br>CARPET . . . . 35<br><br>OTHER ______________________ 96<br>(SPECIFY)	
143	OBSERVE MAIN MATERIAL OF THE ROOF OF THE DWELLING.<br><br>RECORD OBSERVATION.	**NATURAL ROOFING**<br>NO ROOF . . . . 11<br>THATCH/PALM LEAF . . . . 12<br>SOD . . . . 13<br>**RUDIMENTARY ROOFING**<br>RUSTIC MAT . . . . 21<br>PALM/BAMBOO . . . . 22<br>WOOD PLANKS . . . . 23<br>CARDBOARD . . . . 24<br>**FINISHED ROOFING**<br>METAL . . . . 31<br>WOOD . . . . 32<br>CALAMINE/CEMENT FIBER . . . . 33<br>CERAMIC TILES . . . . 34<br>CEMENT . . . . 35<br>ROOFING SHINGLES . . . . 36<br><br>OTHER ______________________ 96<br>(SPECIFY)	

ADDITIONAL HOUSEHOLD CHARACTERISTICS

NO.	QUESTIONS AND FILTERS	CODING CATEGORIES	SKIP
144	OBSERVE MAIN MATERIAL OF THE EXTERIOR WALLS OF THE DWELLING.<br><br>RECORD OBSERVATION.	**NATURAL WALLS**<br>NO WALLS . . . . 11<br>CANE/PALM/TRUNKS . . . . 12<br>DIRT . . . . 13<br>**RUDIMENTARY WALLS**<br>POLE WITH MUD . . . . 21<br>STONE WITH MUD . . . . 22<br>UNCOVERED ADOBE . . . . 23<br>PLYWOOD . . . . 24<br>CARDBOARD . . . . 25<br>REUSED WOOD . . . . 26<br>**FINISHED WALLS**<br>CEMENT . . . . 31<br>STONE WITH LIME/CEMENT . . . . . 32<br>BRICKS . . . . 33<br>CEMENT BLOCKS . . . . 34<br>COVERED ADOBE . . . . 35<br>WOOD PLANKS/SHINGLES . . . . . 36<br><br>OTHER ______________ 96<br>(SPECIFY)	
145	I would like to check whether the salt used in your household is iodized. May I have a sample of the salt used to cook meals in your household?<br><br>TEST SALT FOR IODINE.	IODINE PRESENT . . . . 1<br>NO IODINE . . . . 2<br>NO SALT IN HOUSEHOLD . . . . 3<br><br>SALT NOT TESTED ______________ 6<br>(SPECIFY REASON)	

200	CHECK COL. (5) AND (7) IN THE LIST OF HOUSEHOLD MEMBERS AND WRITE THE TOTAL NUMBER OF CHILDREN **AGE 2-9 YEARS** WHO USUALLY LIVE IN THE HOUSEHOLD (**COL. 5="1"**)	TOTAL NUMBER . . [ ][ ]	
201	CHECK THE NUMBER OF CHILDREN IN 200: ONE OR MORE [ ] ZERO [ ] → 300		
202	CHECK COLUMNS 1, 2, 4, AND 7 IN THE LIST OF HOUSEHOLD MEMBERS. LIST BELOW EACH OF CHILDREN **AGE 2-9 YEARS** WHO U**SUALLY LIVE IN THE HOUSEHOLD**. RECORD THE LINE NUMBER, NAME, SEX AND AGE FOR EACH OF THE CHILDREN. IF MORE THAN FOUR CHILDREN, USE ADDITIONAL QUESTIONNAIRE(S).<br>Now I would like to talk to you about the health condition of children age 2-9 who usually live here. We will talk about each separately. This will take only a few minutes. All the information you give me will remain strictly confidential and your answers will never be shared with those outside of our team.		

		CHILD 1	CHILD 2
203	LINE NUMBER FROM COLUMN 1.<br>NAME FROM COLUMN 2.	LINE NUMBER .... [ ][ ]<br>NAME ________	LINE NUMBER .... [ ][ ]<br>NAME ________
204	CHILD SEX FROM COLUMN 4.	MALE . . . . . 1<br>FEMALE 2	MALE . . . . . 1<br>FEMALE 2
205	CHILD AGE FROM COLUMN 7.	AGE [ ][ ]	AGE [ ][ ]
206	Compared with other children, does or did (NAME) have any serious delay in sitting standing, or walking?	YES . . . . . 1<br>NO 2	YES . . . . . 1<br>NO 2
207	Compared with other children, does (NAME) have difficulty seeing, either in the daytime or at night?	YES . . . . . 1<br>NO 2	YES . . . . . 1<br>NO 2
208	Does (NAME) appear to have any difficulty hearing (uses hearing aid, hears with difficulty or completely deaf)?	YES . . . . . 1<br>NO 2	YES . . . . . 1<br>NO 2
209	When you tell (NAME) to do something, does he/she seem to understand what you are saying?	YES . . . . . 1<br>NO 2	YES . . . . . 1<br>NO 2
210	Does (NAME) have difficulty in walking or moving his/her arms or does he/she have weakness and/or stiffness in the arms or legs?	YES . . . . . 1<br>NO 2	YES . . . . . 1<br>NO 2
211	Does (NAME) sometimes have fits, become rigid, or lose consciousness?	YES . . . . . 1<br>NO 2	YES . . . . . 1<br>NO 2
212	Does (NAME) learn to do things like other children his/her age?	YES . . . . . 1<br>NO 2	YES . . . . . 1<br>NO 2
213	Does (NAME) speak at all (can he/she make him or herself understood in words; can he/she say any recognizable words)?	YES . . . . . 1<br>NO 2	YES . . . . . 1<br>NO 2
214	CHECK 205: CHILD AGE	3-9 YEARS [ ] 2 YEARS [ ] (GO TO 216)	3-9 YEARS [ ] 2 YEARS [ ] (GO TO 216)
215	Is (NAME)'s speech in any way different from normal (not clear enough to be understood by people other than the immediate family)?	YES . . . . . 1<br>(SKIP TO 217)<br>NO 2	YES . . . . . 1<br>(SKIP TO 217)<br>NO 2
216	Can (NAME) name at least one object (for example, an animal, a toy, a cup, a spoon)?	YES . . . . . 1<br>NO 2	YES . . . . . 1<br>NO 2
217	Compared with other children of the same age, does (NAME) appear in any way mentally backward, dull or slow?	YES . . . . . 1<br>NO 2	YES . . . . . 1<br>NO 2
218		GO BACK TO 206 IN NEXT COLUMN OF THIS QUESTIONNAIRE; IF NO MORE CHILDREN, GO TO 300.	GO BACK TO 206 IN THE FIRST COLUMN OF THE NEXT PAGE; IF NO MORE CHILDREN, GO TO 300.

		CHILD 3	CHILD 4
203	LINE NUMBER FROM COLUMN 1.<br><br>NAME FROM COLUMN 2.	LINE NUMBER .... [ ][ ]<br><br>NAME ________	LINE NUMBER .... [ ][ ]<br><br>NAME ________
204	CHILD SEX FROM COLUMN 4.	MALE .... 1<br>FEMALE 2	MALE .... 1<br>FEMALE 2
205	CHILD AGE FROM COLUMN 7.	AGE [ ][ ]	AGE [ ][ ]
206	Compared with other children, does or did (NAME) have any serious delay in sitting standing, or walking?	YES .... 1<br>NO 2	YES .... 1<br>NO 2
207	Compared with other children, does (NAME) have difficulty seeing, either in the daytime or at night?	YES .... 1<br>NO 2	YES .... 1<br>NO 2
208	Does (NAME) appear to have any difficulty hearing (uses hearing aid, hears with difficulty or completely deaf)?	YES .... 1<br>NO 2	YES .... 1<br>NO 2
209	When you tell (NAME) to do something, does he/she seem to understand what you are saying?	YES .... 1<br>NO 2	YES .... 1<br>NO 2
210	Does (NAME) have difficulty in walking or moving his/her arms or does he/she have weakness and/or stiffness in the arms or legs?	YES .... 1<br>NO 2	YES .... 1<br>NO 2
211	Does (NAME) sometimes have fits, become rigid, or lose consciousness?	YES .... 1<br>NO 2	YES .... 1<br>NO 2
212	Does (NAME) learn to do things like other children his/her age?	YES .... 1<br>NO 2	YES .... 1<br>NO 2
213	Does (NAME) speak at all (can he/she make him or herself understood in words; can he/she say any recognizable words)?	YES .... 1<br>NO 2	YES .... 1<br>NO 2
214	CHECK 205: CHILD AGE	3-9 YEARS [ ] ↓<br>2 YEARS [ ] (GO TO 216)	3-9 YEARS [ ] ↓<br>2 YEARS [ ] (GO TO 216)
215	Is (NAME)'s speech in any way different from normal (not clear enough to be understood by people other than the immediate family)?	YES .... 1<br>(SKIP TO 217)<br>NO 2	YES .... 1<br>(SKIP TO 217)<br>NO 2
216	Can (NAME) name at least one object (for example, an animal, a toy, a cup, a spoon)?	YES .... 1<br>NO 2	YES .... 1<br>NO 2
217	Compared with other children of the same age, does (NAME) appear in any way mentally backward, dull or slow?	YES .... 1<br>NO 2	YES .... 1<br>NO 2
218		GO BACK TO 206 IN NEXT COLUMN OF THIS QUESTIONNAIRE; IF NO MORE CHILDREN, GO TO 300.	GO BACK TO 206 IN THE FIRST COLUMN OF A NEW QUESTIONNAIRE; IF NO MORE CHILDREN, GO TO 300.

CHILD FUNCTIONING AND DISABILITY (AGE 10-17)

300	CHECK COL. (5) AND (7) IN THE LIST OF HOUSEHOLD MEMBERS AND WRITE THE TOTAL NUMBER OF CHILDREN **AGE 10-17 YEARS** WHO USUALLY LIVE IN THE HOUSEHOLD (**COL. 5="1"**)		TOTAL NUMBER . . [ ][ ]
301	CHECK THE NUMBER OF CHILDREN IN 300:<br>ONE OR MORE [ ] ↓ ZERO [ ] → 401		
302	CHECK COLUMNS 1, 2, 4, AND 7 IN THE LIST OF HOUSEHOLD MEMBERS. LIST BELOW EACH OF CHILDREN **AGE 10-17 YEARS** WHO U**SUALLY LIVE IN THE HOUSEHOLD**. RECORD THE LINE NUMBER, NAME, SEX AND AGE FOR EACH OF THE CHILDREN. IF MORE THAN FOUR CHILDREN, USE ADDITIONAL QUESTIONNAIRE(S).<br>Now I would like to talk to you about the health condition of children age 10-17 who usually live here. We will talk about each separately. This will take only a few minutes. All the information you give me will remain strictly confidential and your answers will never be shared with those outside of our team.		
		CHILD 1	CHILD 2
303	LINE NUMBER FROM COLUMN 1.<br>NAME FROM COLUMN 2.	LINE NUMBER .... [ ][ ]<br>NAME ________	LINE NUMBER .... [ ][ ]<br>NAME ________
304	CHILD SEX FROM COLUMN 4.	MALE . . . . 1<br>FEMALE 2	MALE . . . . 1<br>FEMALE 2
305	CHILD AGE FROM COLUMN 7.	AGE [ ][ ]	AGE [ ][ ]
306	Does (NAME) wear glasses or contact lenses?	YES . . . . 1<br>NO . . . . 2<br>(SKIP TO 309)	YES . . . . 1<br>NO . . . . 2<br>(SKIP TO 309)
307	Does (NAME) have difficulty seeing even if he/she is wearing glasses or contact lenses?	YES . . . . 1<br>NO . . . . 2<br>(SKIP TO 311)<br>DON'T KNOW 8	YES . . . . 1<br>NO . . . . 2<br>(SKIP TO 311)<br>DON'T KNOW 8
308	Would you say that (NAME) has some difficulty seeing, a lot of difficultly, or can he/she not see at all?	SOME DIFFICULTY . . . . 1<br>A LOT OF DIFFICULTY . . . . 2<br>CAN'T SEE AT ALL . . . . 3<br>DON'T KNOW . . . . 8<br>(SKIP TO 311)	SOME DIFFICULTY . . . . 1<br>A LOT OF DIFFICULTY . . . . 2<br>CAN'T SEE AT ALL . . . . 3<br>DON'T KNOW . . . . 8<br>(SKIP TO 311)
309	Does (NAME) have difficulty seeing?	YES . . . . 1<br>NO . . . . 2<br>(SKIP TO 311)<br>DON'T KNOW 8	YES . . . . 1<br>NO . . . . 2<br>(SKIP TO 311)<br>DON'T KNOW 8
310	Would you say that (NAME) has some difficulty seeing, a lot of difficultly, or can he/she not see at all?	SOME DIFFICULTY . . . . 1<br>A LOT OF DIFFICULTY . . . . 2<br>CAN'T SEE AT ALL . . . . 3<br>DON'T KNOW 8	SOME DIFFICULTY . . . . 1<br>A LOT OF DIFFICULTY . . . . 2<br>CAN'T SEE AT ALL . . . . 3<br>DON'T KNOW 8
311	Does (NAME) use a hearing aid?	YES . . . . 1<br>NO . . . . 2<br>(SKIP TO 314)<br>DON'T KNOW 8	YES . . . . 1<br>NO . . . . 2<br>(SKIP TO 314)<br>DON'T KNOW 8
312	Does (NAME) have difficulty hearing even if he/she is using a hearing aid?	YES . . . . 1<br>NO . . . . 2<br>(SKIP TO 316)<br>DON'T KNOW 8	YES . . . . 1<br>NO . . . . 2<br>(SKIP TO 316)<br>DON'T KNOW 8
313	Would you say that (NAME) has some difficulty hearing, a lot of difficultly, or can he/she not hear at all?	SOME DIFFICULTY . . . . 1<br>A LOT OF DIFFICULTY . . . . 2<br>CAN'T HEAR AT ALL . . . . 3<br>DON'T KNOW . . . . 8<br>(SKIP TO 316)	SOME DIFFICULTY . . . . 1<br>A LOT OF DIFFICULTY . . . . 2<br>CAN'T HEAR AT ALL . . . . 3<br>DON'T KNOW . . . . 8<br>(SKIP TO 316)
314	Does (NAME) have difficulty hearing ?	YES . . . . 1<br>NO . . . . 2<br>(SKIP TO 316)<br>DON'T KNOW 8	YES . . . . 1<br>NO . . . . 2<br>(SKIP TO 316)<br>DON'T KNOW 8
315	Would you say that (NAME) has some difficulty hearing, a lot of difficultly, or can he/she not hear at all?	SOME DIFFICULTY . . . . 1<br>A LOT OF DIFFICULTY . . . . 2<br>CAN'T HEAR AT ALL . . . . 3<br>DON'T KNOW 8	SOME DIFFICULTY . . . . 1<br>A LOT OF DIFFICULTY . . . . 2<br>CAN'T HEAR AT ALL . . . . 3<br>DON'T KNOW 8

		CHILD 1	CHILD 2
303	LINE NUMBER FROM COLUMN 1.<br><br>NAME FROM COLUMN 2.	LINE NUMBER .... [ ][ ]<br><br>NAME ________________	LINE NUMBER .... [ ][ ]<br><br>NAME ________________
316	Does (NAME) have difficulty communicating using his/her usual language, for example understanding or being understood?	YES .... 1<br>NO .... 2<br>(SKIP TO 318)<br>DON'T KNOW 8	YES .... 1<br>NO .... 2<br>(SKIP TO 318)<br>DON'T KNOW 8
317	Would you say that (NAME) has some difficulty communicating, a lot of difficultly, or can he/she not communicate at all?	SOME DIFFICULTY .... 1<br>A LOT OF DIFFICULTY .... 2<br>CAN'T COMMUNICATE AT ALL 3<br>DON'T KNOW 8	SOME DIFFICULTY .... 1<br>A LOT OF DIFFICULTY .... 2<br>CAN'T COMMUNICATE AT ALL 3<br>DON'T KNOW 8
318	Does (NAME) have difficulty remembering or concentrating?	YES .... 1<br>NO .... 2<br>(SKIP TO 320)<br>DON'T KNOW 8	YES .... 1<br>NO .... 2<br>(SKIP TO 320)<br>DON'T KNOW 8
319	Would you say that (NAME) has some difficulty remembering or concentrating, a lot of difficulty, or can he/she not remember or concentrate at all?	SOME DIFFICULTY .... 1<br>A LOT OF DIFFICULTY .... 2<br>CAN'T REM./CONCENT. AT ALL 3<br>DON'T KNOW 8	SOME DIFFICULTY .... 1<br>A LOT OF DIFFICULTY .... 2<br>CAN'T REM./CONCENT. AT ALL 3<br>DON'T KNOW 8
320	Does (NAME) have difficulty walking or climbing steps?	YES .... 1<br>NO .... 2<br>(SKIP TO 322)<br>DON'T KNOW 8	YES .... 1<br>NO .... 2<br>(SKIP TO 322)<br>DON'T KNOW 8
321	Would you say that (NAME) has some difficulty walking or climbing steps, a lot of difficulty, or can he/she not walk or climb steps at all?	SOME DIFFICULTY .... 1<br>A LOT OF DIFFICULTY .... 2<br>CAN'T WALK/CLIMB AT ALL 3<br>DON'T KNOW 8	SOME DIFFICULTY .... 1<br>A LOT OF DIFFICULTY .... 2<br>CAN'T WALK/CLIMB AT ALL 3<br>DON'T KNOW 8
322	Does (NAME) have difficulty washing all over or dressing?	YES .... 1<br>NO .... 2<br>(SKIP TO 324)<br>DON'T KNOW 8	YES .... 1<br>NO .... 2<br>(SKIP TO 324)<br>DON'T KNOW 8
323	Would you say that (NAME) has some difficulty washing all over or dressing, a lot of difficultly, or can he/she not wash all over or dress at all?	SOME DIFFICULTY .... 1<br>A LOT OF DIFFICULTY .... 2<br>CAN'T WASH/DRESS AT ALL . . 3<br>DON'T KNOW 8	SOME DIFFICULTY .... 1<br>A LOT OF DIFFICULTY .... 2<br>CAN'T WASH/DRESS AT ALL . . 3<br>DON'T KNOW 8
324		GO BACK TO 306 IN NEXT COLUMN OF THIS QUESTIONNAIRE; IF NO MORE CHILDREN, GO TO 401.	GO BACK TO 306 IN THE FIRST COLUMN OF THE NEXT PAGE; IF NO MORE CHILDREN, GO TO 401.

		CHILD 3	CHILD 4
303	LINE NUMBER FROM COLUMN 1.<br><br>NAME FROM COLUMN 2.	LINE NUMBER .... [ ][ ]<br><br>NAME ______________	LINE NUMBER .... [ ][ ]<br><br>NAME ______________
304	CHILD SEX FROM COLUMN 4.	MALE .... 1<br>FEMALE 2	MALE .... 1<br>FEMALE 2
305	CHILD AGE FROM COLUMN 7.	AGE [ ][ ]	AGE [ ][ ]
306	Does (NAME) wear glasses or contact lenses?	YES .... 1<br>NO .... 2<br>(SKIP TO 309)	YES .... 1<br>NO .... 2<br>(SKIP TO 309)
307	Does (NAME) have difficulty seeing even if he/she is wearing glasses or contact lenses?	YES .... 1<br>NO .... 2<br>(SKIP TO 311)<br>DON'T KNOW 8	YES .... 1<br>NO .... 2<br>(SKIP TO 311)<br>DON'T KNOW 8
308	Would you say that (NAME) has some difficulty seeing, a lot of difficultly, or can he/she not see at all?	SOME DIFFICULTY .... 1<br>A LOT OF DIFFICULTY .... 2<br>CAN'T SEE AT ALL .... 3<br>DON'T KNOW .... 8<br>(SKIP TO 311)	SOME DIFFICULTY .... 1<br>A LOT OF DIFFICULTY .... 2<br>CAN'T SEE AT ALL .... 3<br>DON'T KNOW .... 8<br>(SKIP TO 311)
309	Does (NAME) have difficulty seeing?	YES .... 1<br>NO .... 2<br>(SKIP TO 311)<br>DON'T KNOW 8	YES .... 1<br>NO .... 2<br>(SKIP TO 311)<br>DON'T KNOW 8
310	Would you say that (NAME) has some difficulty seeing, a lot of difficultly, or can he/she not see at all?	SOME DIFFICULTY .... 1<br>A LOT OF DIFFICULTY .... 2<br>CAN'T SEE AT ALL .... 3<br>DON'T KNOW 8	SOME DIFFICULTY .... 1<br>A LOT OF DIFFICULTY .... 2<br>CAN'T SEE AT ALL .... 3<br>DON'T KNOW 8
311	Does (NAME) use a hearing aid?	YES .... 1<br>NO .... 2<br>(SKIP TO 314)<br>DON'T KNOW 8	YES .... 1<br>NO .... 2<br>(SKIP TO 314)<br>DON'T KNOW 8
312	Does (NAME) have difficulty hearing even if he/she is using a hearing aid?	YES .... 1<br>NO .... 2<br>(SKIP TO 316)<br>DON'T KNOW 8	YES .... 1<br>NO .... 2<br>(SKIP TO 316)<br>DON'T KNOW 8
313	Would you say that (NAME) has some difficulty hearing, a lot of difficultly, or can he/she not hear at all?	SOME DIFFICULTY .... 1<br>A LOT OF DIFFICULTY .... 2<br>CAN'T HEAR AT ALL .... 3<br>DON'T KNOW .... 8<br>(SKIP TO 316)	SOME DIFFICULTY .... 1<br>A LOT OF DIFFICULTY .... 2<br>CAN'T HEAR AT ALL .... 3<br>DON'T KNOW .... 8<br>(SKIP TO 316)
314	Does (NAME) have difficulty hearing ?	YES .... 1<br>NO .... 2<br>(SKIP TO 316)<br>DON'T KNOW 8	YES .... 1<br>NO .... 2<br>(SKIP TO 316)<br>DON'T KNOW 8
315	Would you say that (NAME) has some difficulty hearing, a lot of difficultly, or can he/she not hear at all?	SOME DIFFICULTY .... 1<br>A LOT OF DIFFICULTY .... 2<br>CAN'T HEAR AT ALL .... 3<br>DON'T KNOW 8	SOME DIFFICULTY .... 1<br>A LOT OF DIFFICULTY .... 2<br>CAN'T HEAR AT ALL .... 3<br>DON'T KNOW 8

		CHILD 3	CHILD 4
303	LINE NUMBER FROM COLUMN 1.<br><br>NAME FROM COLUMN 2.	LINE NUMBER .... <br>NAME ___	LINE NUMBER .... <br>NAME ___
316	Does (NAME) have difficulty communicating using his/her usual language, for example understanding or being understood?	YES .... 1<br>NO .... 2<br>(SKIP TO 318)<br>DON'T KNOW 8	YES .... 1<br>NO .... 2<br>(SKIP TO 318)<br>DON'T KNOW 8
317	Would you say that (NAME) has some difficulty communicating, a lot of difficultly, or can he/she not communicate at all?	SOME DIFFICULTY .... 1<br>A LOT OF DIFFICULTY .... 2<br>CAN'T COMMUNICATE AT ALL 3<br>DON'T KNOW 8	SOME DIFFICULTY .... 1<br>A LOT OF DIFFICULTY .... 2<br>CAN'T COMMUNICATE AT ALL 3<br>DON'T KNOW 8
318	Does (NAME) have difficulty remembering or concentrating?	YES .... 1<br>NO .... 2<br>(SKIP TO 320)<br>DON'T KNOW 8	YES .... 1<br>NO .... 2<br>(SKIP TO 320)<br>DON'T KNOW 8
319	Would you say that (NAME) has some difficulty remembering or concentrating, a lot of difficulty, or can he/she not remember or concentrate at all?	SOME DIFFICULTY .... 1<br>A LOT OF DIFFICULTY .... 2<br>CAN'T REM./CONCENT. AT ALL 3<br>DON'T KNOW 8	SOME DIFFICULTY .... 1<br>A LOT OF DIFFICULTY .... 2<br>CAN'T REM./CONCENT. AT ALL 3<br>DON'T KNOW 8
320	Does (NAME) have difficulty walking or climbing steps?	YES .... 1<br>NO .... 2<br>(SKIP TO 322)<br>DON'T KNOW 8	YES .... 1<br>NO .... 2<br>(SKIP TO 322)<br>DON'T KNOW 8
321	Would you say that (NAME) has some difficulty walking or climbing steps, a lot of difficulty, or can he/she not walk or climb steps at all?	SOME DIFFICULTY .... 1<br>A LOT OF DIFFICULTY .... 2<br>CAN'T WALK/CLIMB AT ALL 3<br>DON'T KNOW 8	SOME DIFFICULTY .... 1<br>A LOT OF DIFFICULTY .... 2<br>CAN'T WALK/CLIMB AT ALL 3<br>DON'T KNOW 8
322	Does (NAME) have difficulty washing all over or dressing?	YES .... 1<br>NO .... 2<br>(SKIP TO 324)<br>DON'T KNOW 8	YES .... 1<br>NO .... 2<br>(SKIP TO 324)<br>DON'T KNOW 8
323	Would you say that (NAME) has some difficulty washing all over or dressing, a lot of difficultly, or can he/she not wash all over or dress at all?	SOME DIFFICULTY .... 1<br>A LOT OF DIFFICULTY .... 2<br>CAN'T WASH/DRESS AT ALL .. 3<br>DON'T KNOW 8	SOME DIFFICULTY .... 1<br>A LOT OF DIFFICULTY .... 2<br>CAN'T WASH/DRESS AT ALL .. 3<br>DON'T KNOW 8
324		GO BACK TO 306 IN NEXT COLUMN OF THIS QUESTIONNAIRE; IF NO MORE CHILDREN, GO TO 401.	GO BACK TO 306 IN THE FIRST COLUMN OF A NEW QUESTIONNAIRE; IF NO MORE CHILDREN, GO TO 401.

ELIGIBILITY AND CONSENT FOR THE MICRONUTRIENT SURVEY

NO.			
401	CHECK FRONT COVER<br>HOUSEHOLD SELECTED FOR THE MICRONUTRIENT SURVEY ☐ ↓<br>HOUSEHOLD NOT SELECTED MICRONUTRIENT SURVEY ☐ → 404		
402	a)	AFFIX THE FIRST HOUSEHOLD BAR CODE TO THE MICRONUTRIENT QUESTIONNAIIRE TRANSMITTAL SHEET AND RECORD THE CODE	a) BAR CODE ☐☐☐☐☐
	b)	CHECK COL. 7 IN THE LIST OF HOUSEHOLD MEMBERS AND WRITE THE TOTAL NUMBER OF CHILDREN AGE 0-5 YEARS.	b) TOTAL ELIGIBLE PRESCHOOL (0-5 YRS) ☐☐
	c)	IF HOUSEHOLD **IS NOT** SELECTED FOR SCHOOL-AGE CHILDREN'S SURVEY: RECORD "95"<br>IF HOUSEHOLD **IS SELECTED** FOR SCHOOL-AGE CHILDREN'S SURVEY:<br>CHECK COL. 7 IN THE LIST OF HOUSEHOLD MEMBERS AND WRITE THE TOTAL NUMBER OF CHILDREN AGE 6-14 YEARS.	c) TOTAL ELIGIBLE SCHOOL-AGE (6-14 YRS) ☐☐
	d)	IF HOUSEHOLD **IS NOT** SELECTED FOR WOMEN'S SURVEY: RECORD "95"<br>HOUSEHOLD **IS SELECTED** FOR WOMEN'S SURVEY:<br>CHECK COL. 7 IN THE LIST OF HOUSEHOLD MEMBERS AND WRITE THE TOTAL NUMBER OF WOMEN AGE 15-49 YEARS.	d) TOTAL ELIGIBLE WOMEN (15-49 YRS) ☐☐
	e)	IF HOUSEHOLD **IS NOT** SELECTED FOR MEN'S SURVEY, RECORD "95"<br>IF HOUSEHOLD **IS SELECTED** FOR MEN'S<br>CHECK COL. 7 IN THE LIST OF HOUSEHOLD MEMBERS AND WRITE THE TOTAL NUMBER OF MEN AGE 20-54 YEARS.	e) TOTAL ELIGIBLE MEN (20-54 YRS) ☐☐

ELIGIBILITY AND CONSENT FOR THE MICRONUTRIENT SURVEY

NO.		
403	**PERMISSION TO REVISIT THE HOUSEHOLD BY THE MICRONUTRIENT TEAM**<br><br>In the next few days, my colleagues who are working with the ministry of health would like to revisit your household to conduct a micronutrient study. The micronutrient team will collect samples of sugar, oil, and salt used in the household; conduct a brief interview to assess individual and household-level exposures to nutrition interventions; and collect venous blood and urine samples to evaluate micronutrient status of children aged 6-59 months, school-age children (6-14 years), women age 15-49 years, and men age 20-54 years. You don't have to permit the visit, but we hope you will agree since your household participation is very important. In case you need more information about the revisit, you may contact the person listed on this card.<br><br>GIVE CARD WITH CONTACT INFORMATION<br><br>Do you have any questions?<br>Do you agree for your household to be revisited?<br><br>SIGNATURE OF INTERVIEWER ____________________ DATE __________	
	RESPONDENT AGREES TO BE REVISITED . . 1<br>↓<br>1) COMPLETE IDENTIFICATION SECTION OF THE MICRONUTRIENT QUESTIONNAIRE USING HOUSEHOLD INFORMATION<br><br>2) AFFIX THE SECOND HOUSEHOLD BAR CODE TO THE MICRONUTRIENT QUESTIONNAIRE<br><br>3) RECORD "1": PERMISSION FOR REVISIT WAS GRANTED<br><br>4) RECORD TOTAL NUMBER OF ELIGIBLE RESPONDENTS USING IFNORMATION FROM QUESTION 402<br><br>5) RECORD INFORMATION ABOUT ELIGIBLE PRESCHOOL CHILDREN (201;202); SCHOOL-AGE CHILDREN (301,302); WOMEN (401,402,403); MEN (501) IN THE MICRONUTRIENT QUESTIONNAIRE<br><br>6) HAND OVER THE MICRONUTRIENT QUESTIONNAIRE TO THE MICRONUTRIENT TEAM	RESPONDENT DOES NOT AGREES TO BE REVISITED . . 2<br>↓<br>1) COMPLETE IDENTIFICATION SECTION OF THE MICRONUTRIENT QUESTIONNAIRE USING HOUSEHOLD INFORMATION<br><br>2) AFFIX THE SECOND HOUSEHOLD BAR CODE TO THE MICRONUTRIENT QUESTIONNAIRE<br><br>3) RECORD "2": PERMISSION FOR REVISIT WAS NOT GRANTED<br><br>4) RECORD TOTAL NUMBER OF ELIGIBLE RESPONDENTS USING IFNORMATION FROM QUESTION 402<br><br>5) HAND OVER THE MICRONUTRIENT QUESTIONNAIRE TO THE MICRONUTRIENT TEAM
404	RECORD THE TIME.	HOURS . . . . [ ][ ]<br>MINUTES . . . . [ ][ ]

INTERVIEWER'S OBSERVATIONS

TO BE FILLED IN AFTER COMPLETING INTERVIEW

COMMENTS ABOUT INTERVIEW:

COMMENTS ON SPECIFIC QUESTIONS:

ANY OTHER COMMENTS:

SUPERVISOR'S OBSERVATIONS

EDITOR'S OBSERVATIONS

2015-2016 MALAWI DEMOGRAPHIC AND HEALTH SURVEY
MALAWI GOVERNMENT - NATIONAL STATISTICAL OFFICE
BIOMARKER QUESTIONNAIRE

IDENTIFICATION

PLACE NAME ____________________

NAME OF HOUSEHOLD HEAD ____________________

CLUSTER NUMBER . . . . [ ][ ][ ][ ]

HOUSEHOLD NUMBER . . . . [ ][ ][ ][ ]

HOUSEHOLD SELECTED FOR MAN'S SURVEY? (1=YES, 2=NO) . . . . [ ]

FIELDWORKER VISITS

	1	2	3	FINAL VISIT
DATE<br>FIELDWORKER'S NAME	______<br>______	______<br>______	______<br>______	DAY [ ][ ]<br>MONTH [ ][ ]<br>YEAR [ ][ ][ ][ ]
NEXT VISIT: DATE<br>TIME	______<br>______	______<br>______		TOTAL NUMBER OF VISITS [ ]

NOTES:

TOTAL ELIGIBLE WOMEN [ ][ ]

TOTAL ELIGIBLE MEN [ ][ ]

TOTAL ELIGIBLE CHILDREN [ ][ ]

LANGUAGE OF QUESTIONNAIRE** [0][1]
LANGUAGE OF INTERVIEW** [ ][ ]
NATIVE LANGUAGE OF RESPONDENT** [ ][ ]
TRANSLATOR (YES = 1, NO = 2) [ ]

LANGUAGE OF QUESTIONNAIRE** ENGLISH

**LANGUAGE CODES:
01 ENGLISH
02 CHICHEWA
03 TUMBUKA
09 OTHER ____________ (SPECIFY)

SUPERVISOR		OFFICE EDITOR	KEYED BY
NAME	NUMBER [ ][ ][ ][ ]	NUMBER [ ][ ]	NUMBER [ ][ ]

WEIGHT, HEIGHT AND HEMOGLOBIN MEASUREMENT FOR CHILDREN AGE 0-5

101	CHECK COLUMN 11 IN HOUSEHOLD QUESTIONNAIRE. RECORD THE LINE NUMBER AND NAME FOR ALL ELIGIBLE CHILDREN 0-5 YEARS IN QUESTION 102; IF MORE THAN SIX CHILDREN, USE ADDITIONAL QUESTIONNAIRE(S).			
		CHILD 1	CHILD 2	CHILD 3
102	CHECK HOUSEHOLD QUESTIONNAIRE:<br>LINE NUMBER FROM COLUMN 11.	LINE NUMBER . . . . . . ☐☐<br>NAME ________	LINE NUMBER . . . . . . ☐☐<br>NAME ________	LINE NUMBER . . . . . . ☐☐<br>NAME ________
103	IF MOTHER INTERVIEWED:<br>COPY CHILD'S DATE OF BIRTH (DAY, MONTH, AND YEAR) FROM BIRTH HISTORY. IF MOTHER NOT INTERVIEWED ASK:<br>What is (NAME)'s date of birth?	DAY . . . . ☐☐<br>MONTH . . . . ☐☐<br>YEAR . . . ☐☐☐☐	DAY . . . . ☐☐<br>MONTH . . . . ☐☐<br>YEAR . . . ☐☐☐☐	DAY . . . . ☐☐<br>MONTH . . . . ☐☐<br>YEAR . . . ☐☐☐☐
104	CHECK 103: CHILD BORN IN 2010-2015?	YES . . . . 1<br>NO . . . . 2<br>(SKIP TO 114)	YES . . . . 1<br>NO . . . . 2<br>(SKIP TO 114)	YES . . . . 1<br>NO . . . . 2<br>(SKIP TO 114)
105	WEIGHT IN KILOGRAMS.	KG. . . . ☐☐.☐☐<br>NOT PRESENT . . . . 9994<br>REFUSED . . . . 9995<br>OTHER 9996	KG. . . . ☐☐.☐☐<br>NOT PRESENT . . . . 9994<br>REFUSED . . . . 9995<br>OTHER 9996	KG. . . . ☐☐.☐☐<br>NOT PRESENT . . . . 9994<br>REFUSED . . . . 9995<br>OTHER 9996
106	HEIGHT IN CENTIMETERS.	CM. . . . ☐☐☐.☐<br>NOT PRESENT . . . . 9994<br>REFUSED . . . . 9995<br>OTHER . . . . 9996<br>(SKIP TO 108)	CM. . . . ☐☐☐.☐<br>NOT PRESENT . . . . 9994<br>REFUSED . . . . 9995<br>OTHER . . . . 9996<br>(SKIP TO 108)	CM. . . . ☐☐☐.☐<br>NOT PRESENT . . . . 9994<br>REFUSED . . . . 9995<br>OTHER . . . . 9996<br>(SKIP TO 108)
107	MEASURED LYING DOWN OR STANDING UP?	LYING DOWN . . . . . . 1<br>STANDING UP 2	LYING DOWN . . . . . . 1<br>STANDING UP 2	LYING DOWN . . . . . . 1<br>STANDING UP 2
108	MEASURER: ENTER YOUR FIELDWORKER NUMBER.	☐☐☐☐<br>FIELDWORKER NUMBER	☐☐☐☐<br>FIELDWORKER NUMBER	☐☐☐☐<br>FIELDWORKER NUMBER

WEIGHT, HEIGHT AND HEMOGLOBIN MEASUREMENT FOR CHILDREN AGE 0-5

101	CHECK COLUMN 11 IN HOUSEHOLD QUESTIONNAIRE. RECORD THE LINE NUMBER AND NAME FOR ALL ELIGIBLE CHILDREN 0-5 YEARS IN QUESTION 102; IF MORE THAN SIX CHILDREN, USE ADDITIONAL QUESTIONNAIRE(S).			
		CHILD 1	CHILD 2	CHILD 3
102	CHECK HOUSEHOLD QUESTIONNAIRE:<br>LINE NUMBER FROM COLUMN 11.	LINE NUMBER . . . . . . [ ][ ]<br>NAME ________	LINE NUMBER . . . . . . [ ][ ]<br>NAME ________	LINE NUMBER . . . . . . [ ][ ]<br>NAME ________
109	CHECK 103: CHILD AGE 0-5 MONTHS, I.E., WAS CHILD BORN IN MONTH OF INTERVIEW OR 5 PREVIOUS MONTHS?	0-5 MONTHS . . . . 1<br>(SKIP TO 114)<br>OLDER 2	0-5 MONTHS . . . . 1<br>(SKIP TO 114)<br>OLDER 2	0-5 MONTHS . . . . 1<br>(SKIP TO 114)<br>OLDER 2
110	LINE NUMBER OF PARENT/OTHER ADULT RESPONSIBLE FOR THE CHILD FROM COLUMN 1 OF HOUSEHOLD SCHEDULE.	LINE NUMBER . . . . . . [ ][ ]<br>(RECORD '00' IF NOT LISTED)	LINE NUMBER . . . . . . [ ][ ]<br>(RECORD '00' IF NOT LISTED)	LINE NUMBER . . . . . . [ ][ ]<br>(RECORD '00' IF NOT LISTED)
111	ASK CONSENT FOR ANEMIA TEST FROM PARENT/OTHER ADULT.	As part of this survey, we are asking people all over the country to take an anemia test. Anemia is a serious health problem that usually results from poor nutrition, infection, or chronic disease. This survey will assist the government to develop programs to prevent and treat anemia. We ask that all children born in 2010 or later take part in anemia testing in this survey and give a few drops of blood from a finger or heel. The equipment used to take the blood is clean and completely safe. It has never been used before and will be thrown away after each test.<br><br>The blood will be tested for anemia immediately, and the result will be told to you right away. The result will be kept strictly confidential and will not be shared with anyone other than members of our survey team.<br><br>Do you have any questions?<br>You can say yes or no. It is up to you to decide.<br>Will you allow (NAME OF CHILD) to participate in the anemia test?		
112	CIRCLE THE CODE AND SIGN YOUR NAME.	GRANTED . . . . 1<br>________ (SIGN)<br>REFUSED . . . . 2<br>NOT PRESENT/OTHER . 3<br>(SKIP TO 114)	GRANTED . . . . 1<br>________ (SIGN)<br>REFUSED . . . . 2<br>NOT PRESENT/OTHER . 3<br>(SKIP TO 114)	GRANTED . . . . 1<br>________ (SIGN)<br>REFUSED . . . . 2<br>NOT PRESENT/OTHER . 3<br>(SKIP TO 114)
113	RECORD HEMOGLOBIN LEVEL HERE AND IN THE ANEMIA PAMPHLET.	G/DL . . . . [ ][ ].[ ]<br>REFUSED . . . . .995<br>OTHER 996	G/DL . . . . [ ][ ].[ ]<br>REFUSED . . . . .995<br>OTHER 996	G/DL . . . . [ ][ ].[ ]<br>REFUSED . . . . .995<br>OTHER 996
114	GO BACK TO 103 IN NEXT COLUMN OF THIS QUESTIONNAIRE OR IN THE FIRST COLUMN OF THE NEXT PAGE;<br>IF NO MORE CHILDREN, GO TO 201.			

		CHILD 4	CHILD 5	CHILD 6
102	CHECK HOUSEHOLD QUESTIONNAIRE:<br>LINE NUMBER FROM COLUMN 11.	LINE NUMBER . . . . . .<br>NAME	LINE NUMBER . . . . . .<br>NAME	LINE NUMBER . . . . . .<br>NAME
103	IF MOTHER INTERVIEWED:<br>COPY CHILD'S DATE OF BIRTH (DAY, MONTH, AND YEAR) FROM BIRTH HISTORY. IF MOTHER NOT INTERVIEWED ASK:<br>What is (NAME)'s date of birth?	DAY . . . .<br>MONTH . . . .<br>YEAR . . .	DAY . . . .<br>MONTH . . . .<br>YEAR . . .	DAY . . . .<br>MONTH . . . .<br>YEAR . . .
104	CHECK 103: CHILD BORN IN 2010-2015?	YES . . . . 1<br>NO . . . . 2<br>(SKIP TO 114)	YES . . . . 1<br>NO . . . . 2<br>(SKIP TO 114)	YES . . . . 1<br>NO . . . . 2<br>(SKIP TO 114)
105	WEIGHT IN KILOGRAMS.	KG. . . . __ __ . __ __<br>NOT PRESENT . . . . 9994<br>REFUSED . . . . 9995<br>OTHER 9996	KG. . . . __ __ . __ __<br>NOT PRESENT . . . . 9994<br>REFUSED . . . . 9995<br>OTHER 9996	KG. . . . __ __ . __ __<br>NOT PRESENT . . . . 9994<br>REFUSED . . . . 9995<br>OTHER 9996
106	HEIGHT IN CENTIMETERS.	CM. . . . __ __ __ . __<br>NOT PRESENT . . . . 9994<br>REFUSED . . . . 9995<br>OTHER . . . . 9996<br>(SKIP TO 108)	CM. . . . __ __ __ . __<br>NOT PRESENT . . . . 9994<br>REFUSED . . . . 9995<br>OTHER . . . . 9996<br>(SKIP TO 108)	CM. . . . __ __ __ . __<br>NOT PRESENT . . . . 9994<br>REFUSED . . . . 9995<br>OTHER . . . . 9996<br>(SKIP TO 108)
107	MEASURED LYING DOWN OR STANDING UP?	LYING DOWN . . . . . . 1<br>STANDING UP 2	LYING DOWN . . . . . . 1<br>STANDING UP 2	LYING DOWN . . . . . . 1<br>STANDING UP 2
108	MEASURER: ENTER YOUR FIELDWORKER NUMBER.	FIELDWORKER NUMBER	FIELDWORKER NUMBER	FIELDWORKER NUMBER

		CHILD 4	CHILD 5	CHILD 6
102	CHECK HOUSEHOLD QUESTIONNAIRE:<br>LINE NUMBER FROM COLUMN 11.	LINE NUMBER . . . . . . ☐☐<br>NAME ______	LINE NUMBER . . . . . . ☐☐<br>NAME ______	LINE NUMBER . . . . . . ☐☐<br>NAME ______
109	CHECK 103: CHILD AGE 0-5 MONTHS, I.E., WAS CHILD BORN IN MONTH OF INTERVIEW OR 5 PREVIOUS MONTHS?	0-5 MONTHS . . . . 1<br>(SKIP TO 114)<br>OLDER 2	0-5 MONTHS . . . . 1<br>(SKIP TO 114)<br>OLDER 2	0-5 MONTHS . . . . 1<br>(SKIP TO 114)<br>OLDER 2
110	LINE NUMBER OF PARENT/OTHER ADULT RESPONSIBLE FOR THE CHILD FROM COLUMN 1 OF HOUSEHOLD SCHEDULE.	LINE NUMBER . . . . . . ☐☐<br>(RECORD '00' IF NOT LISTED)	LINE NUMBER . . . . . . ☐☐<br>(RECORD '00' IF NOT LISTED)	LINE NUMBER . . . . . . ☐☐<br>(RECORD '00' IF NOT LISTED)
111	ASK CONSENT FOR ANEMIA TEST FROM PARENT/OTHER ADULT.	As part of this survey, we are asking people all over the country to take an anemia test. Anemia is a serious health problem that usually results from poor nutrition, infection, or chronic disease. This survey will assist the government to develop programs to prevent and treat anemia. We ask that all children born in 2010 or later take part in anemia testing in this survey and give a few drops of blood from a finger or heel. The equipment used to take the blood is clean and completely safe. It has never been used before and will be thrown away after each test.<br><br>The blood will be tested for anemia immediately, and the result will be told to you right away. The result will be kept strictly confidential and will not be shared with anyone other than members of our survey team.<br><br>Do you have any questions?<br>You can say yes or no. It is up to you to decide.<br>Will you allow (NAME OF CHILD) to participate in the anemia test?		
112	CIRCLE THE CODE AND SIGN YOUR NAME.	GRANTED . . . . 1<br>______ (SIGN)<br>REFUSED . . . . 2<br>NOT PRESENT/OTHER . 3<br>(SKIP TO 114)	GRANTED . . . . 1<br>______ (SIGN)<br>REFUSED . . . . 2<br>NOT PRESENT/OTHER . 3<br>(SKIP TO 114)	GRANTED . . . . 1<br>______ (SIGN)<br>REFUSED . . . . 2<br>NOT PRESENT/OTHER . 3<br>(SKIP TO 114)
113	RECORD HEMOGLOBIN LEVEL HERE AND IN THE ANEMIA PAMPHLET.	G/DL . . . . ☐☐.☐<br>REFUSED . . . . 995<br>OTHER 996	G/DL . . . . ☐☐.☐<br>REFUSED . . . . 995<br>OTHER 996	G/DL . . . . ☐☐.☐<br>REFUSED . . . . 995<br>OTHER 996
114	GO BACK TO 103 IN NEXT COLUMN OF THIS QUESTIONNAIRE OR IN THE FIRST COLUMN OF AN ADDITIONAL QUESTIONNAIRE; IF NO MORE CHILDREN, GO TO 201.			

201	CHECK COLUMN 9 IN HOUSEHOLD QUESTIONNAIRE. RECORD THE LINE NUMBER, NAME, AGE, AND MARITAL STATUS FOR ALL ELIGIBLE WOMEN IN 202, 203, AND 204.<br>IF THERE ARE MORE THAN THREE WOMEN, USE ADDITIONAL QUESTIONNAIRE(S).			
		WOMAN 1	WOMAN 2	WOMAN 3
202	CHECK HOUSEHOLD QUESTIONNAIRE:<br>LINE NUMBER FROM COLUMN 9.<br>NAME FROM COLUMN 2.	LINE NUMBER . . . . ☐☐<br>NAME ________	LINE NUMBER . . . . ☐☐<br>NAME ________	LINE NUMBER . . . . ☐☐<br>NAME ________
203	CHECK HOUSEHOLD QUESTIONNAIRE COLUMN 7 (AGE):	15-17 YEARS . . . . 1<br>18-49 YEARS 2	15-17 YEARS . . . . 1<br>18-49 YEARS 2	15-17 YEARS . . . . 1<br>18-49 YEARS 2
204	CHECK HOUSEHOLD QUESTIONNAIRE COLUMN 8 (MARITAL STATUS):	CODE 4 (NEVER IN UNION) . 1<br>OTHER 2	CODE 4 (NEVER IN UNION) . 1<br>OTHER 2	CODE 4 (NEVER IN UNION) . 1<br>OTHER 2

205	WEIGHT IN KILOGRAMS.	KG. . . . ☐☐☐.☐☐<br>NOT PRESENT . . . . . . 99994<br>REFUSED . . . . 99995<br>OTHER 99996	KG. . . . ☐☐☐.☐☐<br>NOT PRESENT . . . . . . 99994<br>REFUSED . . . . 99995<br>OTHER 99996	KG. . . . ☐☐☐.☐☐<br>NOT PRESENT . . . . . . 99994<br>REFUSED . . . . 99995<br>OTHER 99996
206	HEIGHT IN CENTIMETERS.	CM. . . . . . . ☐☐☐.☐<br>NOT PRESENT . . . . 9994<br>REFUSED . . . . 9995<br>OTHER 9996	CM. . . . . . . ☐☐☐.☐<br>NOT PRESENT . . . . 9994<br>REFUSED . . . . 9995<br>OTHER 9996	CM. . . . . . . ☐☐☐.☐<br>NOT PRESENT . . . . 9994<br>REFUSED . . . . 9995<br>OTHER 9996
207	MEASURER: ENTER YOUR FIELDWORKER NUMBER.	☐☐☐☐<br>FIELDWORKER NUMBER	☐☐☐☐<br>FIELDWORKER NUMBER	☐☐☐☐<br>FIELDWORKER NUMBER
208	CHECK 203: AGE	15-17 YEARS . . . . 1<br>18-49 YEARS . . . . 2<br>(SKIP TO 210)	15-17 YEARS . . . . 1<br>18-49 YEARS . . . . 2<br>(SKIP TO 210)	15-17 YEARS . . . . 1<br>18-49 YEARS . . . . 2<br>(SKIP TO 210)
209	CHECK 204: MARITAL STATUS	CODE 4 (NEVER IN UNION) . 1<br>(SKIP TO 216)<br>OTHER 2	CODE 4 (NEVER IN UNION) . 1<br>(SKIP TO 216)<br>OTHER 2	CODE 4 (NEVER IN UNION) . 1<br>(SKIP TO 216)<br>OTHER 2

		WOMAN 1	WOMAN 2	WOMAN 3
	NAME FROM COLUMN 2.	NAME ____________	NAME ____________	NAME ____________

ADULT RESPONDENT CONSENT FOR ANEMIA TEST

		WOMAN 1	WOMAN 2	WOMAN 3
210	ASK CONSENT FOR ANEMIA TEST.	As part of this survey, we are asking people all over the country to take an anemia test. Anemia is a serious health problem that usually results from poor nutrition, infection, or chronic disease. This survey will assist the government to develop programs to prevent and treat anemia.<br><br>For the anemia testing, we will need a few drops of blood from a finger. The equipment used to take the blood is clean and completely safe. It has never been used before and will be thrown away after we take your blood. The blood will be tested for anemia immediately, and the result will be told to you right away. The result will be kept strictly confidential and will not be shared with anyone other than members of our survey team.<br><br>Do you have any questions?<br>You can say yes or no. It is up to you to decide.<br>Will you take the anemia test?		
211	CIRCLE THE CODE AND SIGN YOUR NAME.	GRANTED . . . . 1<br>RESPONDENT REFUSED . . . 2<br>____________<br>(SIGN)<br>(IF REFUSED, SKIP TO 212)<br>NOT PRESENT/OTHER . . . . 3<br>(SKIP TO 212)	GRANTED . . . . 1<br>RESPONDENT REFUSED . . . 2<br>____________<br>(SIGN)<br>(IF REFUSED, SKIP TO 212)<br>NOT PRESENT/OTHER . . . . 3<br>(SKIP TO 212)	GRANTED . . . . 1<br>RESPONDENT REFUSED . . . 2<br>____________<br>(SIGN)<br>(IF REFUSED, SKIP TO 212)<br>NOT PRESENT/OTHER . . . . 3<br>(SKIP TO 212)
211A	CHECK 226 IN WOMAN'S QUESTIONNAIRE OR ASK:<br>Are you pregnant?	YES . . . . 1<br>NO . . . . 2<br>DON'T KNOW 8	YES . . . . 1<br>NO . . . . 2<br>DON'T KNOW 8	YES . . . . 1<br>NO . . . . 2<br>DON'T KNOW 8

ADULT RESPONDENT CONSENT FOR DBS COLLECTION

		WOMAN 1	WOMAN 2	WOMAN 3
212	ASK CONSENT FOR DBS COLLECTION.	As part of the survey we also are asking people all over the country to give blood for HIV testing. HIV is the virus that can lead to AIDS. The HIV testing is being done to see how many people have HIV.<br><br>For the HIV testing, we need a few (more) drops of blood from a finger. The equipment used to take the blood is clean and completely safe. It has never been used before and will be thrown away after we take your blood. No names will be attached so we will not be able to tell you the test results. No one else will be able to know your test results either. If you want to know whether you have HIV, I can provide you with a list of [nearby] facilities offering counseling and testing for HIV. I will also give you a voucher for free services for you (and for your partner if you want) that you can use at any of these facilities.<br><br>Do you have any questions?<br>You can say yes or no. It is up to you to decide.<br>Will you give blood for the HIV testing?		
213	CIRCLE THE CODE, SIGN YOUR NAME, AND ENTER YOUR FIELDWORKER NUMBER.	GRANTED . . . . 1<br>RESPONDENT REFUSED . . . 2<br>____________<br>(SIGN AND ENTER YOUR FIELDWORKER NUMBER)<br>[ ][ ][ ][ ]<br>(IF REFUSED, SKIP TO 229)<br>NOT PRESENT/OTHER . . . . 3<br>(SKIP TO 229)	GRANTED . . . . 1<br>RESPONDENT REFUSED . . . 2<br>____________<br>(SIGN AND ENTER YOUR FIELDWORKER ID NUMBER)<br>[ ][ ][ ][ ]<br>(IF REFUSED, SKIP TO 229)<br>NOT PRESENT/OTHER . . . . 3<br>(SKIP TO 229)	GRANTED . . . . 1<br>RESPONDENT REFUSED . . . 2<br>____________<br>(SIGN AND ENTER YOUR FIELDWORKER ID NUMBER)<br>[ ][ ][ ][ ]<br>(IF REFUSED, SKIP TO 229)<br>NOT PRESENT/OTHER . . . . 3<br>(SKIP TO 229)

		WOMAN 1	WOMAN 2	WOMAN 3
	NAME FROM COLUMN 2.	NAME ____________	NAME ____________	NAME ____________
**ADULT RESPONDENT CONSENT FOR ADDITIONAL TESTING**				
214	ASK CONSENT FOR ADDITIONAL TESTING.	We ask you to allow the National Statistical Office to store part of the blood sample at the laboratory for additional tests or research. We are not certain about what additional tests might be done.<br><br>The blood sample will not have any name or other data attached that could identify you. You do not have to agree. If you do not want the blood sample stored for additional testing, you can still participate in the HIV testing in this survey.<br><br>Will you allow us to keep the blood sample stored for additional testing?		
215	CIRCLE THE CODE AND SIGN YOUR NAME.	GRANTED . . . . 1<br>RESPONDENT REFUSED . . . 2<br>____________<br>(SIGN AND SKIP TO 229)	GRANTED . . . . 1<br>RESPONDENT REFUSED . . . 2<br>____________<br>(SIGN AND SKIP TO 229)	GRANTED . . . . 1<br>RESPONDENT REFUSED . . . 2<br>____________<br>(SIGN AND SKIP TO 229)

ADULT RESPONDENT CONSENT

		WOMAN 1	WOMAN 2	WOMAN 3
	NAME FROM COLUMN 2.	NAME ________	NAME ________	NAME ________
216	RECORD LINE NUMBER OF PARENT/OTHER ADULT RESPONSIBLE FOR ADOLESCENT.	LINE NUMBER OF PARENT OR OTHER RESPONSIBLE ADULT [ ][ ] (RECORD '00' IF NOT LISTED)	LINE NUMBER OF PARENT OR OTHER RESPONSIBLE ADULT [ ][ ] (RECORD '00' IF NOT LISTED)	LINE NUMBER OF PARENT OR OTHER RESPONSIBLE ADULT [ ][ ] (RECORD '00' IF NOT LISTED)

PARENT — RESP ADULT CONSENT

PARENTAL/RESPONSIBLE ADULT CONSENT FOR ANEMIA TEST

217	ASK CONSENT FOR ANEMIA TEST FROM PARENT/ADULT.	As part of this survey, we are asking people all over the country to take an anemia test. Anemia is a serious health problem that usually results from poor nutrition, infection, or chronic disease. This survey will assist the government to develop programs to prevent and treat anemia.<br><br>For the anemia testing, we will need a few drops of blood from a finger. The equipment used to take the blood is clean and completely safe. It has never been used before and will be thrown away after each test. The blood will be tested for anemia immediately, and the result will be told to you and (NAME OF MINOR) right away. The result will be kept strictly confidential and will not be shared with anyone other than members of our survey team.<br><br>Do you have any questions?<br>You can say yes or no. It is up to you to decide.<br>Will you allow (NAME OF MINOR) to take the anemia test?

		WOMAN 1	WOMAN 2	WOMAN 3
218	CIRCLE THE CODE AND SIGN YOUR NAME.	GRANTED . . . . 1<br>PARENT/OTHER RESPONSIBLE ADULT REFUSED . . . . . . 2<br>________<br>(SIGN)<br>(IF REFUSED, SKIP TO 221)<br>NOT PRESENT/OTHER . . . . 3<br>(SKIP TO 221)	GRANTED . . . . 1<br>PARENT/OTHER RESPONSIBLE ADULT REFUSED . . . . . . 2<br>________<br>(SIGN)<br>(IF REFUSED, SKIP TO 221)<br>NOT PRESENT/OTHER . . . . 3<br>(SKIP TO 221)	GRANTED . . . . 1<br>PARENT/OTHER RESPONSIBLE ADULT REFUSED . . . . . . 2<br>________<br>(SIGN)<br>(IF REFUSED, SKIP TO 221)<br>NOT PRESENT/OTHER . . . . 3<br>(SKIP TO 221)

MINOR RESPONDENT CONSENT

MINOR RESPONDENT CONSENT FOR ANEMIA TEST

219	ASK CONSENT FOR ANEMIA TEST FROM RESPONDENT.	As part of this survey, we are asking people all over the country to take an anemia test. Anemia is a serious health problem that usually results from poor nutrition, infection, or chronic disease. This survey will assist the government to develop programs to prevent and treat anemia.<br><br>For the anemia testing, we will need a few drops of blood from a finger. The equipment used to take the blood is clean and completely safe. It has never been used before and will be thrown away after we take your blood. The blood will be tested for anemia immediately, and the result will be told to you and (NAME OF PARENT/RESPONSIBLE ADULT) right away. The result will be kept strictly confidential and will not be shared with anyone other than members of our survey team.<br><br>Do you have any questions?<br>You can say yes or no. It is up to you to decide.<br>Will you take the anemia test?

		WOMAN 1	WOMAN 2	WOMAN 3
220	CIRCLE THE CODE AND SIGN YOUR NAME.	GRANTED . . . . 1<br>MINOR RESPONDENT REFUSED . . . . 2<br>________<br>(SIGN)<br>(IF REFUSED, SKIP TO 221)<br>NOT PRESENT/OTHER . . . . 3<br>(SKIP TO 221)	GRANTED . . . . 1<br>MINOR RESPONDENT REFUSED . . . . 2<br>________<br>(SIGN)<br>(IF REFUSED, SKIP TO 221)<br>NOT PRESENT/OTHER . . . . 3<br>(SKIP TO 221)	GRANTED . . . . 1<br>MINOR RESPONDENT REFUSED . . . . 2<br>________<br>(SIGN)<br>(IF REFUSED, SKIP TO 221)<br>NOT PRESENT/OTHER . . . . 3<br>(SKIP TO 221)
220A	CHECK 226 IN WOMAN'S QUESTIONNAIRE OR ASK:<br>Are you pregnant?	YES . . . . 1<br>NO . . . . 2<br>DON'T KNOW 8	YES . . . . 1<br>NO . . . . 2<br>DON'T KNOW 8	YES . . . . 1<br>NO . . . . 2<br>DON'T KNOW 8

		WOMAN 1	WOMAN 2	WOMAN 3
	NAME FROM COLUMN 2.	NAME ______	NAME ______	NAME ______

PARENTAL/RESPONSIBLE ADULT CONSENT FOR DBS COLLECTION

PARENT/RESP ADULT CONSENT

221	ASK CONSENT FOR DBS COLLECTION FROM PARENT/ADULT.	As part of the survey we also are asking people all over the country to take an HIV test. HIV is the virus that can lead to AIDS. The HIV test is being done to see how many people have HIV.<br><br>For the HIV test, we need a few (more) drops of blood from a finger. The equipment used to take the blood is clean and completely safe. It has never been used before and will be thrown away after each test. No names will be attached so we will not be able to tell you the test results. No one else will be able to know (NAME OF MINOR)'s test results either. If (NAME OF MINOR) wants to know her HIV status, I can provide a list of [nearby] facilities offering counseling and testing for HIV. I will also give her a voucher for free services that can be used at any of these facilities.<br><br>Do you have any questions?<br>You can say yes or no. It is up to you to decide.<br>Will you allow (NAME OF MINOR) to give blood for the HIV testing?		
222	CIRCLE THE CODE, SIGN YOUR NAME, AND ENTER YOUR FIELDWORKER NUMBER.	GRANTED . . . . 1<br>PARENT/OTHER RESPONSIBLE ADULT REFUSED . . . . . . 2<br><br>______<br>(SIGN AND ENTER YOUR FIELDWORKER NUMBER)<br>[ ][ ][ ][ ]<br>(IF REFUSED, SKIP TO 229)<br><br>NOT PRESENT/OTHER . . . . 3<br>(SKIP TO 229)	GRANTED . . . . 1<br>PARENT/OTHER RESPONSIBLE ADULT REFUSED . . . . . . 2<br><br>______<br>(SIGN AND ENTER YOUR FIELDWORKER NUMBER)<br>[ ][ ][ ][ ]<br>(IF REFUSED, SKIP TO 229)<br><br>NOT PRESENT/OTHER . . . . 3<br>(SKIP TO 229)	GRANTED . . . . 1<br>PARENT/OTHER RESPONSIBLE ADULT REFUSED . . . . . . 2<br><br>______<br>(SIGN AND ENTER YOUR FIELDWORKER NUMBER)<br>[ ][ ][ ][ ]<br>(IF REFUSED, SKIP TO 229)<br><br>NOT PRESENT/OTHER . . . . 3<br>(SKIP TO 229)

MINOR RESPONDENT CONSENT FOR DBS COLLECTION

MINOR RESPONDENT CONSENT

223	ASK CONSENT FOR DBS COLLECTION FROM MINOR RESPONDENT.	As part of the survey we also are asking people all over the country to give blood for HIV testing. HIV is the virus that can lead to AIDS. The HIV testing is being done to see how many people have HIV.<br><br>For the HIV testing, we need a few (more) drops of blood from a finger. The equipment used to take the blood is clean and completely safe. It has never been used before and will be thrown away after we take your blood. No names will be attached so we will not be able to tell you the test results. No one else will be able to know your test results either. If you want to know whether you have HIV, I can provide you with a list of [nearby] facilities offering counseling and testing for HIV. I will also give you a voucher for free services for you (and for your partner if you want) that you can use at any of these facilities.<br><br>Do you have any questions?<br>You can say yes or no. It is up to you to decide.<br>Will you give blood for the HIV testing?		
224	CIRCLE THE CODE AND SIGN YOUR NAME.	GRANTED . . . . 1<br>MINOR RESPONDENT REFUSED . . . . 2<br><br>______<br>(SIGN)<br>(IF REFUSED, SKIP TO 229)<br><br>NOT PRESENT/OTHER . . . . 3<br>(SKIP TO 229)	GRANTED . . . . 1<br>MINOR RESPONDENT REFUSED . . . . 2<br><br>______<br>(SIGN)<br>(IF REFUSED, SKIP TO 229)<br><br>NOT PRESENT/OTHER . . . . 3<br>(SKIP TO 229)	GRANTED . . . . 1<br>MINOR RESPONDENT REFUSED . . . . 2<br><br>______<br>(SIGN)<br>(IF REFUSED, SKIP TO 229)<br><br>NOT PRESENT/OTHER . . . . 3<br>(SKIP TO 229)

		WOMAN 1	WOMAN 2	WOMAN 3
	NAME FROM COLUMN 2.	NAME ____________	NAME ____________	NAME ____________
**PARENTAL/RESPONSIBLE ADULT CONSENT FOR ADDITIONAL TESTING**				
225	ASK CONSENT FOR ADDITIONAL TESTING FROM PARENT/ADULT.	We ask you to allow the National Statistical Office to store part of the blood sample at the laboratory for additional tests or research. We are not certain about what additional tests might be done.<br><br>The blood sample will not have any name or other data attached that could identify (NAME OF MINOR). You do not have to agree. If you do not want the blood sample stored for additional testing, (NAME OF MINOR) can still participate in the HIV testing in this survey.<br><br>Will you allow us to keep the blood sample stored for additional testing?		
226	CIRCLE THE CODE AND SIGN YOUR NAME.	GRANTED . . . . 1<br>PARENT/OTHER RESPONSIBLE ADULT REFUSED . . . . . . 2<br><br>____________<br>(SIGN)<br>(IF REFUSED, SKIP TO 229)	GRANTED . . . . 1<br>PARENT/OTHER RESPONSIBLE ADULT REFUSED . . . . . . 2<br><br>____________<br>(SIGN)<br>(IF REFUSED, SKIP TO 229)	GRANTED . . . . 1<br>PARENT/OTHER RESPONSIBLE ADULT REFUSED . . . . . . 2<br><br>____________<br>(SIGN)<br>(IF REFUSED, SKIP TO 229)

MINOR RESPONDENT CONSENT

**MINOR RESPONDENT CONSENT FOR ADDITIONAL TESTING**				
227	ASK CONSENT FOR ADDITIONAL TESTING FROM MINOR RESPONDENT.	We ask you to allow the National Statistical Office to store part of the blood sample at the laboratory for additional tests or research. We are not certain about what additional tests might be done.<br><br>The blood sample will not have any name or other data attached that could identify you. You do not have to agree. If you do not want the blood sample stored for additional testing, you can still participate in the HIV testing in this survey.<br><br>Will you allow us to keep the blood sample stored for additional testing?		
228	CIRCLE THE CODE AND SIGN YOUR NAME.	GRANTED . . . . 1<br>MINOR RESPONDENT REFUSED . . . . 2<br><br>____________<br>(SIGN)	GRANTED . . . . 1<br>MINOR RESPONDENT REFUSED . . . . 2<br><br>____________<br>(SIGN)	GRANTED . . . . 1<br>MINOR RESPONDENT REFUSED . . . . 2<br><br>____________<br>(SIGN)

		WOMAN 1	WOMAN 2	WOMAN 3
	NAME FROM COLUMN 2.	NAME ____________	NAME ____________	NAME ____________
229	PREPARE EQUIPMENT AND SUPPLIES ONLY FOR THE TEST(S) FOR WHICH CONSENT HAS BEEN OBTAINED AND PROCEED WITH THE TEST(S).			
230	ADDITIONAL TESTS.	IF ADULT RESPONDENT, CHECK 215; IF MINOR RESPONDENT, CHECK 226 AND 228.<br><br>IF CONSENT HAS NOT BEEN GRANTED, WRITE "NO ADDITIONAL TESTS" ON THE FILTER PAPER.	IF ADULT RESPONDENT, CHECK 215; IF MINOR RESPONDENT, CHECK 226 AND 228.<br><br>IF CONSENT HAS NOT BEEN GRANTED, WRITE "NO ADDITIONAL TESTS" ON THE FILTER PAPER.	IF ADULT RESPONDENT, CHECK 215; IF MINOR RESPONDENT, CHECK 226 AND 228.<br><br>IF CONSENT HAS NOT BEEN GRANTED, WRITE "NO ADDITIONAL TESTS" ON THE FILTER PAPER.
231	RECORD HEMOGLOBIN LEVEL HERE AND IN ANEMIA PAMPHLET.	G/DL . . . . [ ][ ].[ ]<br>NOT PRESENT . . . . 994<br>REFUSED . . . . 995<br>OTHER 996	G/DL . . . . [ ][ ].[ ]<br>NOT PRESENT . . . . 994<br>REFUSED . . . . 995<br>OTHER 996	G/DL . . . . [ ][ ].[ ]<br>NOT PRESENT . . . . 994<br>REFUSED . . . . 995<br>OTHER 996
232	PLACE BAR CODE LABEL.	PUT THE 1ST BAR CODE LABEL HERE.<br><br>NOT PRESENT . . . . . . 99994<br>REFUSED . . . . 99995<br>OTHER . . . . 99996<br><br>PUT THE 2ND BAR CODE LABEL ON THE RESPONDENT'S FILTER PAPER AND THE 3RD ON THE TRANSMITTAL FORM.	PUT THE 1ST BAR CODE LABEL HERE.<br><br>NOT PRESENT . . . . . . 99994<br>REFUSED . . . . 99995<br>OTHER . . . . 99996<br><br>PUT THE 2ND BAR CODE LABEL ON THE RESPONDENT'S FILTER PAPER AND THE 3RD ON THE TRANSMITTAL FORM.	PUT THE 1ST BAR CODE LABEL HERE.<br><br>NOT PRESENT . . . . . . 99994<br>REFUSED . . . . 99995<br>OTHER . . . . 99996<br><br>PUT THE 2ND BAR CODE LABEL ON THE RESPONDENT'S FILTER PAPER AND THE 3RD ON THE TRANSMITTAL FORM.
233	GO BACK TO 202 IN NEXT COLUMN OF THIS QUESTIONNAIRE OR IN THE FIRST COLUMN OF AN ADDITIONAL QUESTIONNAIRE; IF NO MORE WOMEN, GO TO 301.			

301	CHECK COLUMN 10 IN HOUSEHOLD QUESTIONNAIRE. RECORD THE LINE NUMBER, NAME, AGE, AND MARITAL STATUS FOR ALL ELIGIBLE MEN IN 302, 303, AND 304.<br>IF THERE ARE MORE THAN THREE MEN, USE ADDITIONAL QUESTIONNAIRE(S).			
		MAN 1	MAN 2	MAN 3
302	CHECK HOUSEHOLD QUESTIONNAIRE:<br><br>LINE NUMBER FROM COLUMN 10.<br><br>NAME FROM COLUMN 2.	LINE NUMBER . . . . [ ][ ]<br><br>NAME ____________	LINE NUMBER . . . . [ ][ ]<br><br>NAME ____________	LINE NUMBER . . . . [ ][ ]<br><br>NAME ____________
303	CHECK HOUSEHOLD QUESTIONNAIRE COLUMN 7 (AGE):	15-17 YEARS . . . . 1<br>18-54 YEARS 2	15-17 YEARS . . . . 1<br>18-54 YEARS 2	15-17 YEARS . . . . 1<br>18-54 YEARS 2
304	CHECK HOUSEHOLD QUESTIONNAIRE COLUMN 8 (MARITAL STATUS):	CODE 4 (NEVER IN UNION) . 1<br>OTHER 2	CODE 4 (NEVER IN UNION) . 1<br>OTHER 2	CODE 4 (NEVER IN UNION) . 1<br>OTHER 2

308	CHECK 303: AGE	15-17 YEARS . . . . 1<br>18-54 YEARS . . . . 2<br>(SKIP TO 312)	15-17 YEARS . . . . 1<br>18-54 YEARS . . . . 2<br>(SKIP TO 312)	15-17 YEARS . . . . 1<br>18-54 YEARS . . . . 2<br>(SKIP TO 312)
309	CHECK 304: MARITAL STATUS	CODE 4 (NEVER IN UNION) . 1<br>(SKIP TO 316)<br>OTHER 2	CODE 4 (NEVER IN UNION) . 1<br>(SKIP TO 316)<br>OTHER 2	CODE 4 (NEVER IN UNION) . 1<br>(SKIP TO 312)<br>OTHER 2

		MAN 1	MAN 2	MAN 3
	NAME FROM COLUMN 2.	NAME ______	NAME ______	NAME ______

ADULT RESPONDENT CONSENT FOR DBS COLLECTION

312	ASK CONSENT FOR DBS COLLECTION.	As part of the survey we also are asking people all over the country to give blood for HIV testing. HIV is the virus that can lead to AIDS. The HIV testing is being done to see how many people have HIV.<br><br>For the HIV testing, we need a few drops of blood from a finger. The equipment used to take the blood is clean and completely safe. It has never been used before and will be thrown away after we take your blood. No names will be attached so we will not be able to tell you the test results. No one else will be able to know your test results either. If you want to know whether you have HIV, I can provide you with a list of [nearby] facilities offering counseling and testing for HIV. I will also give you a voucher for free services for you (and for your partner if you want) that you can use at any of these facilities.<br><br>Do you have any questions?<br>You can say yes or no. It is up to you to decide.<br>Will you give blood for the HIV testing?		
313	CIRCLE THE CODE, SIGN YOUR NAME, AND ENTER YOUR FIELDWORKER NUMBER.	GRANTED . . . . 1<br>RESPONDENT REFUSED . . . 2<br>______<br>(SIGN AND ENTER YOUR FIELDWORKER NUMBER)<br>[ ][ ][ ][ ]<br>(IF REFUSED, SKIP TO 329)<br>NOT PRESENT/OTHER . . . . 3<br>(SKIP TO 329)	GRANTED . . . . 1<br>RESPONDENT REFUSED . . . 2<br>______<br>(SIGN AND ENTER YOUR FIELDWORKER NUMBER)<br>[ ][ ][ ][ ]<br>(IF REFUSED, SKIP TO 329)<br>NOT PRESENT/OTHER . . . . 3<br>(SKIP TO 329)	GRANTED . . . . 1<br>RESPONDENT REFUSED . . . 2<br>______<br>(SIGN AND ENTER YOUR FIELDWORKER NUMBER)<br>[ ][ ][ ][ ]<br>(IF REFUSED, SKIP TO 329)<br>NOT PRESENT/OTHER . . . . 3<br>(SKIP TO 329)

ADULT RESPONDENT CONSENT FOR ADDITIONAL TESTING

314	ASK CONSENT FOR ADDITIONAL TESTING.	We ask you to allow the National Statistical Office to store part of the blood sample at the laboratory for additional tests or research. We are not certain about what additional tests might be done.<br><br>The blood sample will not have any name or other data attached that could identify you. You do not have to agree. If you do not want the blood sample stored for additional testing, you can still participate in the HIV testing in this survey.<br><br>Will you allow us to keep the blood sample stored for additional testing?		
315	CIRCLE THE CODE AND SIGN YOUR NAME.	GRANTED . . . . 1<br>RESPONDENT REFUSED . . . 2<br>______<br>(SIGN AND SKIP TO 329)	GRANTED . . . . 1<br>RESPONDENT REFUSED . . . 2<br>______<br>(SIGN AND SKIP TO 329)	GRANTED . . . . 1<br>RESPONDENT REFUSED . . . 2<br>______<br>(SIGN AND SKIP TO 329)

		MAN 1	MAN 2	MAN 3
	NAME FROM COLUMN 2.	NAME ______	NAME ______	NAME ______
316	RECORD LINE NUMBER OF PARENT/OTHER ADULT RESPONSIBLE FOR ADOLESCENT.	LINE NUMBER OF PARENT OR OTHER RESPONSIBLE ADULT [ ][ ] (RECORD '00' IF NOT LISTED)	LINE NUMBER OF PARENT OR OTHER RESPONSIBLE ADULT [ ][ ] (RECORD '00' IF NOT LISTED)	LINE NUMBER OF PARENT OR OTHER RESPONSIBLE ADULT [ ][ ] (RECORD '00' IF NOT LISTED)

PARENTAL/RESPONSIBLE ADULT CONSENT FOR DBS COLLECTION

PARENT — RESP ADULT CONSENT

321	ASK CONSENT FOR DBS COLLECTION FROM PARENT/ADULT.	As part of the survey we also are asking people all over the country to take an HIV test. HIV is the virus that can lead to AIDS. The HIV test is being done to see how many people have HIV.<br><br>For the HIV test, we need a few drops of blood from a finger. The equipment used to take the blood is clean and completely safe. It has never been used before and will be thrown away after we take your blood. No names will be attached so we will not be able to tell you the test results. No one else will be able to know (NAME OF MINOR)'s test results either. If (NAME OF MINOR) wants to know his HIV status, I can provide a list of [nearby] facilities offering counseling and testing for HIV. I will also give him a voucher for free services that can be used at any of these facilities.<br><br>Do you have any questions?<br>You can say yes or no. It is up to you to decide.<br>Will you allow (NAME OF MINOR) to give blood for the HIV testing?		
322	CIRCLE THE CODE, SIGN YOUR NAME, AND ENTER YOUR FIELDWORKER NUMBER.	GRANTED . . . . 1<br>PARENT/OTHER RESPONSIBLE ADULT REFUSED . . . . . . 2<br>______<br>(SIGN AND ENTER YOUR FIELDWORKER NUMBER)<br>[ ][ ][ ][ ]<br>(IF REFUSED, SKIP TO 329)<br>NOT PRESENT/OTHER . . . . 3<br>(SKIP TO 329)	GRANTED . . . . 1<br>PARENT/OTHER RESPONSIBLE ADULT REFUSED . . . . . . 2<br>______<br>(SIGN AND ENTER YOUR FIELDWORKER NUMBER)<br>[ ][ ][ ][ ]<br>(IF REFUSED, SKIP TO 329)<br>NOT PRESENT/OTHER . . . . 3<br>(SKIP TO 329)	GRANTED . . . . 1<br>PARENT/OTHER RESPONSIBLE ADULT REFUSED . . . . . . 2<br>______<br>(SIGN AND ENTER YOUR FIELDWORKER NUMBER)<br>[ ][ ][ ][ ]<br>(IF REFUSED, SKIP TO 329)<br>NOT PRESENT/OTHER . . . . 3<br>(SKIP TO 329)

MINOR RESPONDENT CONSENT FOR DBS COLLECTION

MINOR RESPONDENT CONSENT

323	ASK CONSENT FOR DBS COLLECTION FROM MINOR RESPONDENT.	As part of the survey we also are asking people all over the country to give blood for HIV testing. HIV is the virus that can lead to AIDS. The HIV testing is being done to see how many people have HIV.<br><br>For the HIV testing, we need a few drops of blood from a finger. The equipment used to take the blood is clean and completely safe. It has never been used before and will be thrown away after each test. No names will be attached so we will not be able to tell you the test results. No one else will be able to know your test results either. If you want to know whether you have HIV, I can provide you with a list of [nearby] facilities offering counseling and testing for HIV. I will also give you a voucher for free services for you (and for your partner if you want) that you can use at any of these facilities.<br><br>Do you have any questions?<br>You can say yes or no. It is up to you to decide.<br>Will you give blood for the HIV testing?		
324	CIRCLE THE CODE AND SIGN YOUR NAME.	GRANTED . . . . 1<br>MINOR RESPONDENT REFUSED . . . . 2<br>______<br>(SIGN)<br>(IF REFUSED, SKIP TO 329)<br>NOT PRESENT/OTHER . . . . 3<br>(SKIP TO 329)	GRANTED . . . . 1<br>MINOR RESPONDENT REFUSED . . . . 2<br>______<br>(SIGN)<br>(IF REFUSED, SKIP TO 329)<br>NOT PRESENT/OTHER . . . . 3<br>(SKIP TO 329)	GRANTED . . . . 1<br>MINOR RESPONDENT REFUSED . . . . 2<br>______<br>(SIGN)<br>(IF REFUSED, SKIP TO 329)<br>NOT PRESENT/OTHER . . . . 3<br>(SKIP TO 329)

		MAN 1	MAN 2	MAN 3
	NAME FROM COLUMN 2.	NAME ________	NAME ________	NAME ________

PARENTAL/RESPONSIBLE ADULT CONSENT FOR ADDITIONAL TESTING

(PARENT — RESP ADULT CONSENT)

325	ASK CONSENT FOR ADDITIONAL TESTING FROM PARENT/ADULT.	We ask you to allow National Statistical Office to store part of the blood sample at the laboratory for additional tests or research. We are not certain about what additional tests might be done.<br><br>The blood sample will not have any name or other data attached that could identify (NAME OF MINOR). You do not have to agree. If you do not want the blood sample stored for additional testing, (NAME OF MINOR) can still participate in the HIV testing in this survey.<br><br>Will you allow us to keep the blood sample stored for additional testing?		
326	CIRCLE THE CODE AND SIGN YOUR NAME.	GRANTED . . . . 1<br>PARENT/OTHER RESPONSIBLE ADULT REFUSED . . . . . . 2<br><br>________<br>(SIGN)<br>(IF REFUSED, SKIP TO 329)	GRANTED . . . . 1<br>PARENT/OTHER RESPONSIBLE ADULT REFUSED . . . . . . 2<br><br>________<br>(SIGN)<br>(IF REFUSED, SKIP TO 329)	GRANTED . . . . 1<br>PARENT/OTHER RESPONSIBLE ADULT REFUSED . . . . . . 2<br><br>________<br>(SIGN)<br>(IF REFUSED, SKIP TO 329)

MINOR RESPONDENT CONSENT FOR ADDITIONAL TESTING

(MINOR RESPONDENT CONSENT)

327	ASK CONSENT FOR ADDITIONAL TESTING FROM MINOR RESPONDENT.	We ask you to allow National Statistical Office to store part of the blood sample at the laboratory for additional tests or research. We are not certain about what additional tests might be done.<br><br>The blood sample will not have any name or other data attached that could identify you. You do not have to agree. If you do not want the blood sample stored for additional testing, you can still participate in the HIV testing in this survey.<br><br>Will you allow us to keep the blood sample stored for additional testing?		
328	CIRCLE THE CODE AND SIGN YOUR NAME.	GRANTED . . . . 1<br>MINOR RESPONDENT REFUSED . . . . 2<br><br>________<br>(SIGN)	GRANTED . . . . 1<br>MINOR RESPONDENT REFUSED . . . . 2<br><br>________<br>(SIGN)	GRANTED . . . . 1<br>MINOR RESPONDENT REFUSED . . . . 2<br><br>________<br>(SIGN)

		MAN 1	MAN 2	MAN 3
	NAME FROM COLUMN 2.	NAME ______	NAME ______	NAME ______
329	PREPARE EQUIPMENT AND SUPPLIES ONLY FOR THE TEST(S) FOR WHICH CONSENT HAS BEEN OBTAINED AND PROCEED WITH THE TEST(S).			
330	ADDITIONAL TESTS.	IF ADULT RESPONDENT, CHECK 315; IF MINOR RESPONDENT, CHECK 326 AND 328.<br><br>IF CONSENT HAS NOT BEEN GRANTED, WRITE "NO ADDITIONAL TESTS" ON THE FILTER PAPER.	IF ADULT RESPONDENT, CHECK 315; IF MINOR RESPONDENT, CHECK 326 AND 328.<br><br>IF CONSENT HAS NOT BEEN GRANTED, WRITE "NO ADDITIONAL TESTS" ON THE FILTER PAPER.	IF ADULT RESPONDENT, CHECK 315; IF MINOR RESPONDENT, CHECK 326 AND 328.<br><br>IF CONSENT HAS NOT BEEN GRANTED, WRITE "NO ADDITIONAL TESTS" ON THE FILTER PAPER.
332	PLACE BAR CODE LABEL.	PUT THE 1ST BAR CODE LABEL HERE.<br><br>NOT PRESENT . . . . . . 99994<br>REFUSED . . . . 99995<br>OTHER . . . . 99996<br><br>PUT THE 2ND BAR CODE LABEL ON THE RESPONDENT'S FILTER PAPER AND THE 3RD ON THE TRANSMITTAL FORM.	PUT THE 1ST BAR CODE LABEL HERE.<br><br>NOT PRESENT . . . . . . 99994<br>REFUSED . . . . 99995<br>OTHER . . . . 99996<br><br>PUT THE 2ND BAR CODE LABEL ON THE RESPONDENT'S FILTER PAPER AND THE 3RD ON THE TRANSMITTAL FORM.	PUT THE 1ST BAR CODE LABEL HERE.<br><br>NOT PRESENT . . . . . . 99994<br>REFUSED . . . . 99995<br>OTHER . . . . 99996<br><br>PUT THE 2ND BAR CODE LABEL ON THE RESPONDENT'S FILTER PAPER AND THE 3RD ON THE TRANSMITTAL FORM.
333	GO BACK TO 302 IN NEXT COLUMN OF THIS QUESTIONNAIRE OR IN THE FIRST COLUMN OF AN ADDITIONAL QUESTIONNAIRE; IF NO MORE MEN, END INTERVIEW.			

FIELDWORKER'S OBSERVATIONS

TO BE FILLED IN AFTER COMPLETING BIOMARKERS

SUPERVISOR'S OBSERVATIONS

EDITOR'S OBSERVATIONS

2015-2016 MALAWI DEMOGRAPHIC AND HEALTH SURVEY
MALAWI GOVERNMENT - NATIONAL STATISTICAL OFFICE
WOMAN'S QUESTIONNAIRE

IDENTIFICATION

PLACE NAME ______

NAME OF HOUSEHOLD HEAD ______

CLUSTER NUMBER . . . . [ ][ ][ ][ ]

HOUSEHOLD NUMBER . . . . [ ][ ][ ][ ]

NAME AND LINE NUMBER OF WOMAN ______ [ ][ ]

WOMAN SELECTED FOR DOMESTIC VIOLENCE MODULE? (1=YES, 2=NO) . . . . [ ]

INTERVIEWER VISITS

	1	2	3	FINAL VISIT
DATE	______	______	______	DAY [ ][ ]<br>MONTH [ ][ ]<br>YEAR [ ][ ][ ][ ]
INTERVIEWER'S NAME	______	______	______	INT. NO. [ ][ ][ ][ ]
RESULT*	______	______	______	RESULT* [ ]
NEXT VISIT: DATE	______	______		TOTAL NUMBER OF VISITS [ ]
TIME	______	______		

*RESULT CODES:
1 COMPLETED
2 NOT AT HOME
3 POSTPONED
4 REFUSED
5 PARTLY COMPLETED
6 INCAPACITATED
7 OTHER ______ (SPECIFY)

LANGUAGE OF QUESTIONNAIRE** [0][1]
LANGUAGE OF INTERVIEW** [ ][ ]
NATIVE LANGUAGE OF RESPONDENT** [ ][ ]
TRANSLATOR USED (YES = 1, NO = 2) [ ]

LANGUAGE OF QUESTIONNAIRE** **ENGLISH**

**LANGUAGE CODES:
01 ENGLISH
02 CHICHEWA
03 TUMBUKA
09 OTHER ______ (SPECIFY)

SUPERVISOR		OFFICE EDITOR	KEYED BY
______<br>NAME	[ ][ ][ ][ ]<br>NUMBER	[ ][ ]<br>NUMBER	[ ][ ]<br>NUMBER

INTRODUCTION AND CONSENT

Hello. My name is ______________________________________. I am working with The National Statistical Office. We are conducting a survey about health and other topics all over Malawi. The information we collect will help the government to plan health services. Your household was selected for the survey. The questions usually take about 30 to 60 minutes. All of the answers you give will be confidential and will not be shared with anyone other than members of our survey team. You don't have to be in the survey, but we hope you will agree to answer the questions since your views are important. If I ask you any question you don't want to answer, just let me know and I will go on to the next question or you can stop the interview at any time.

In case you need more information about the survey, you may contact the person listed on the card that has already been given to your household.

Do you have any questions?
May I begin the interview now?

SIGNATURE OF INTERVIEWER ______________________ DATE ______________

RESPONDENT AGREES TO BE INTERVIEWED . . 1 → SECTION 1

RESPONDENT DOES NOT AGREE TO BE INTERVIEWED . . 2 → END

SECTION 1. RESPONDENT'S BACKGROUND

NO.	QUESTIONS AND FILTERS	CODING CATEGORIES	SKIP
101	RECORD THE TIME.	HOURS . . . . [ ][ ]<br>MINUTES . . . . [ ][ ]	
102	How long have you been living continuously in (NAME OF CURRENT CITY, TOWN OR VILLAGE OF RESIDENCE)?<br><br>IF LESS THAN ONE YEAR, RECORD ‘00’ YEARS.	YEARS . . . . [ ][ ]<br>ALWAYS . . . . 95<br>VISITOR 96	95, 96 → 105
103	Just before you moved here, did you live in a city, in a town, or in a rural area?	CITY . . . . 1<br>TOWN . . . . 2<br>RURAL AREA 3	
104	Before you moved here, which [REGION] did you live in?	NOTHERN . . . . 01<br>CENTRAL . . . . 02<br>SOUTHERN . . . . 03<br>OUTSIDE OF MALAWI 96	
105	In what month and year were you born?	MONTH . . . . [ ][ ]<br>DON'T KNOW MONTH . . . . 98<br>YEAR . . . . [ ][ ][ ][ ]<br>DON'T KNOW YEAR 9998	
106	How old were you at your last birthday?<br><br>COMPARE AND CORRECT 105 AND/OR 106 IF INCONSISTENT.	AGE IN COMPLETED YEARS . . . . [ ][ ]	
107	Have you ever attended school?	YES . . . . 1<br>NO 2	2 → 111
108	What is the highest level of school you attended: primary, secondary, or higher?	PRIMARY . . . . 1<br>SECONDARY . . . . 2<br>HIGHER 3	

SECTION 1. RESPONDENT'S BACKGROUND

NO.	QUESTIONS AND FILTERS	CODING CATEGORIES	SKIP
109	What is the highest [FORM/YEAR] you completed at that level?<br><br>IF COMPLETED LESS THAN ONE YEAR AT THAT LEVEL, RECORD '00'.	[GRADE/FORM/YEAR] . . . . ☐☐	
110	CHECK 108:<br>PRIMARY OR SECONDARY ☐ ↓ HIGHER ☐		→ 113
111	Now I would like you to read this sentence to me.<br><br>SHOW CARD TO RESPONDENT.<br><br>IF RESPONDENT CANNOT READ WHOLE SENTENCE,<br>PROBE: Can you read any part of the sentence to me?	CANNOT READ AT ALL . . . . 1<br>ABLE TO READ ONLY PART OF THE SENTENCE . . . . 2<br>ABLE TO READ WHOLE SENTENCE . . . . 3<br>NO CARD WITH REQUIRED LANGUAGE ________ 4<br>(SPECIFY LANGUAGE)<br>BLIND/VISUALLY IMPAIRED 5	
112	CHECK 111:<br>CODE '2', '3' OR '4' CIRCLED ☐ ↓ CODE '1' OR '5' CIRCLED ☐		→ 114
113	Do you read a newspaper or magazine at least once a week, less than once a week or not at all?	AT LEAST ONCE A WEEK . . . . 1<br>LESS THAN ONCE A WEEK . . . . 2<br>NOT AT ALL 3	
114	Do you listen to the radio at least once a week, less than once a week or not at all?	AT LEAST ONCE A WEEK . . . . 1<br>LESS THAN ONCE A WEEK . . . . 2<br>NOT AT ALL 3	
115	Do you watch television at least once a week, less than once a week or not at all?	AT LEAST ONCE A WEEK . . . . 1<br>LESS THAN ONCE A WEEK . . . . 2<br>NOT AT ALL 3	
116	Do you own a mobile telephone?	YES . . . . 1<br>NO 2	 → 118
117	Do you use your mobile phone for any financial transactions?	YES . . . . 1<br>NO 2	
118	Do you have an account in a bank or other financial institution that you yourself use?	YES . . . . 1<br>NO 2	
119	Have you ever used the internet?<br><br>IF NECESSARY, PROBE FOR USE FROM ANY LOCATION, WITH ANY DEVICE.	YES . . . . 1<br>NO 2	 → 122
120	In the last 12 months, have you used the internet?<br><br>IF NECESSARY, PROBE FOR USE FROM ANY LOCATION, WITH ANY DEVICE.	YES . . . . 1<br>NO 2	 → 122
121	During the last one month, how often did you use the internet: almost every day, at least once a week, less than once a week, or not at all?	ALMOST EVERY DAY . . . . 1<br>AT LEAST ONCE A WEEK . . . . 2<br>LESS THAN ONCE A WEEK . . . . 3<br>NOT AT ALL 4	

SECTION 1. RESPONDENT'S BACKGROUND

NO.	QUESTIONS AND FILTERS	CODING CATEGORIES	SKIP
122	What is your religion?	CATHOLIC . . . 01<br>CCAP . . . 02<br>ANGLICAN . . . 03<br>SEVENTH DAY ADVENT./BAPTIST . . . 04<br>OTHER CHRISTIAN . . . 05<br>MUSLIM . . . 06<br>NO RELIGION . . . 07<br><br>OTHER ______ 96<br>(SPECIFY)	
123	What is your tribe or ethnic group?	CHEWA . . . 01<br>TUMBUKA . . . 02<br>LOMWE . . . 03<br>TONGA . . . 04<br>YAO . . . 05<br>SENA . . . 06<br>NKHONDE . . . 07<br>NGONI . . . 08<br><br>OTHER ______ 96<br>(SPECIFY)	
124	In the last 12 months, how many times have you been away from home for one or more nights?	NUMBER OF TIMES . . . [ ][ ]<br><br>NONE . . . 00	→201
125	In the last 12 months, have you been away from home for more than one month at a time?	YES . . . 1<br>NO . . . 2	

NO.	QUESTIONS AND FILTERS	CODING CATEGORIES	SKIP
201	Now I would like to ask about all the births you have had during your life. Have you ever given birth?	YES . . . . . 1<br>NO 2	 → 206
202	Do you have any sons or daughters to whom you have given birth who are now living with you?	YES . . . . . 1<br>NO 2	 → 204
203	a) How many sons live with you?<br>b) And how many daughters live with you?<br>IF NONE, RECORD '00'.	a) SONS AT HOME . . . . . [ ][ ]<br>b) DAUGHTERS AT HOME . . . . . [ ][ ]	
204	Do you have any sons or daughters to whom you have given birth who are alive but do not live with you?	YES . . . . . 1<br>NO 2	 → 206
205	a) How many sons are alive but do not live with you?<br>b) And how many daughters are alive but do not live with you?<br>IF NONE, RECORD '00'.	a) SONS ELSEWHERE . . . . . [ ][ ]<br>b) DAUGHTERS ELSEWHERE . . . . . [ ][ ]	
206	Have you ever given birth to a boy or girl who was born alive but later died?<br>IF NO, PROBE: Any baby who cried, who made any movement, sound, or effort to breathe, or who showed any other signs of life even if for a very short time?	YES . . . . . 1<br>NO 2	 → 208
207	a) How many boys have died?<br>b) And how many girls have died?<br>IF NONE, RECORD '00'.	a) BOYS DEAD . . . . . [ ][ ]<br>b) GIRLS DEAD . . . . . [ ][ ]	
208	SUM ANSWERS TO 203, 205, AND 207, AND ENTER TOTAL. IF NONE, RECORD '00'.	TOTAL BIRTHS . . . . . [ ][ ]	
209	CHECK 208:<br>Just to make sure that I have this right: you have had in TOTAL _____ births during your life. Is that correct?<br>YES [ ] ↓ NO [ ] → PROBE AND CORRECT 201-208 AS NECESSARY.		
210	CHECK 208:<br>ONE OR MORE BIRTHS [ ] ↓ NO BIRTHS [ ]		→ 226

211 Now I would like to record the names of all your births, whether still alive or not, starting with the first one you had.
RECORD NAMES OF ALL THE BIRTHS IN 212. RECORD TWINS AND TRIPLETS ON SEPARATE ROWS. IF THERE ARE MORE THAN 10 BIRTHS, USE AN ADDITIONAL QUESTIONNAIRE, STARTING WITH THE SECOND ROW.

212<br>What name was given to your (first/ next) baby?<br>RECORD NAME.<br>BIRTH HISTORY NUMBER.	213<br>Is (NAME) a boy or a girl?	214<br>Were any of these births twins?	215<br>On what day, month, and year was (NAME) born?	216<br>Is (NAME) still alive?	217<br>IF ALIVE:<br>How old was (NAME) at (NAME)'s last birthday?<br>RECORD AGE IN COMP-LETED YEARS.	218<br>IF ALIVE:<br>Is (NAME) living with you?	219<br>IF ALIVE:<br>RECORD HOUSEHOLD LINE NUMBER OF CHILD. RECORD '00' IF CHILD NOT LISTED IN HOUSEHOLD.	220<br>IF DEAD:<br>How old was (NAME) when (he/she) died?<br>IF '12 MONTHS' OR '1 YR', ASK: Did (NAME) have (his/her) first birthday?<br>THEN ASK: Exactly how many months old was (NAME) when (he/she) died?<br>RECORD DAYS IF LESS THAN 1 MONTH; MONTHS IF LESS THAN TWO YEARS; OR YEARS.	221<br>Were there any other live births between (NAME OF PREVIOUS BIRTH) and (NAME), including any children who died after birth?
01	BOY 1<br>GIRL 2	SING 1<br>MULT 2	DAY ☐☐<br>MONTH ☐☐<br>☐☐☐☐<br>YEAR	YES 1<br>NO 2<br>(SKIP TO 220)	AGE IN YEARS<br>☐☐	YES 1<br>NO 2	HOUSEHOLD LINE NUMBER<br>☐☐<br>(NEXT BIRTH)	DAYS 1 ☐☐<br>MONTHS 2 ☐☐<br>YEARS 3 ☐☐	
02	BOY 1<br>GIRL 2	SING 1<br>MULT 2	DAY ☐☐<br>MONTH ☐☐<br>☐☐☐☐<br>YEAR	YES 1<br>NO 2<br>(SKIP TO 220)	AGE IN YEARS<br>☐☐	YES 1<br>NO 2	HOUSEHOLD LINE NUMBER<br>☐☐<br>(SKIP TO 221)	DAYS 1 ☐☐<br>MONTHS 2 ☐☐<br>YEARS 3 ☐☐	YES 1 (ADD BIRTH)<br>NO 2 (NEXT BIRTH)
03	BOY 1<br>GIRL 2	SING 1<br>MULT 2	DAY ☐☐<br>MONTH ☐☐<br>☐☐☐☐<br>YEAR	YES 1<br>NO 2<br>(SKIP TO 220)	AGE IN YEARS<br>☐☐	YES 1<br>NO 2	HOUSEHOLD LINE NUMBER<br>☐☐<br>(SKIP TO 221)	DAYS 1 ☐☐<br>MONTHS 2 ☐☐<br>YEARS 3 ☐☐	YES 1 (ADD BIRTH)<br>NO 2 (NEXT BIRTH)
04	BOY 1<br>GIRL 2	SING 1<br>MULT 2	DAY ☐☐<br>MONTH ☐☐<br>☐☐☐☐<br>YEAR	YES 1<br>NO 2<br>(SKIP TO 220)	AGE IN YEARS<br>☐☐	YES 1<br>NO 2	HOUSEHOLD LINE NUMBER<br>☐☐<br>(SKIP TO 221)	DAYS 1 ☐☐<br>MONTHS 2 ☐☐<br>YEARS 3 ☐☐	YES 1 (ADD BIRTH)<br>NO 2 (NEXT BIRTH)
05	BOY 1<br>GIRL 2	SING 1<br>MULT 2	DAY ☐☐<br>MONTH ☐☐<br>☐☐☐☐<br>YEAR	YES 1<br>NO 2<br>(SKIP TO 220)	AGE IN YEARS<br>☐☐	YES 1<br>NO 2	HOUSEHOLD LINE NUMBER<br>☐☐<br>(SKIP TO 221)	DAYS 1 ☐☐<br>MONTHS 2 ☐☐<br>YEARS 3 ☐☐	YES 1 (ADD BIRTH)<br>NO 2 (NEXT BIRTH)

212 What name was given to your (first/ next) baby? RECORD NAME. BIRTH HISTORY NUMBER.	213 Is (NAME) a boy or a girl?	214 Were any of these births twins?	215 On what day, month, and year was (NAME) born?	216 Is (NAME) still alive?	217 IF ALIVE: How old was (NAME) at (NAME)'s last birthday? RECORD AGE IN COMP-LETED YEARS.	218 IF ALIVE: Is (NAME) living with you?	219 IF ALIVE: RECORD HOUSEHOLD LINE NUMBER OF CHILD. RECORD '00' IF CHILD NOT LISTED IN HOUSEHOLD.	220 IF DEAD: How old was (NAME) when (he/she) died? IF '12 MONTHS' OR '1 YR', ASK: Did (NAME) have (his/her) first birthday? THEN ASK: Exactly how many months old was (NAME) when (he/she) died? RECORD DAYS IF LESS THAN 1 MONTH; MONTHS IF LESS THAN TWO YEARS; OR YEARS.	221 Were there any other live births between (NAME OF PREVIOUS BIRTH) and (NAME), including any children who died after birth?
06	BOY 1<br>GIRL 2	SING 1<br>MULT 2	DAY<br>MONTH<br>YEAR	YES 1<br>NO 2<br>(SKIP TO 220)	AGE IN YEARS	YES 1<br>NO 2	HOUSEHOLD LINE NUMBER<br>(SKIP TO 221)	DAYS 1<br>MONTHS 2<br>YEARS 3	YES 1 (ADD BIRTH)<br>NO 2 (NEXT BIRTH)
07	BOY 1<br>GIRL 2	SING 1<br>MULT 2	DAY<br>MONTH<br>YEAR	YES 1<br>NO 2<br>(SKIP TO 220)	AGE IN YEARS	YES 1<br>NO 2	HOUSEHOLD LINE NUMBER<br>(SKIP TO 221)	DAYS 1<br>MONTHS 2<br>YEARS 3	YES 1 (ADD BIRTH)<br>NO 2 (NEXT BIRTH)
08	BOY 1<br>GIRL 2	SING 1<br>MULT 2	DAY<br>MONTH<br>YEAR	YES 1<br>NO 2<br>(SKIP TO 220)	AGE IN YEARS	YES 1<br>NO 2	HOUSEHOLD LINE NUMBER<br>(SKIP TO 221)	DAYS 1<br>MONTHS 2<br>YEARS 3	YES 1 (ADD BIRTH)<br>NO 2 (NEXT BIRTH)
09	BOY 1<br>GIRL 2	SING 1<br>MULT 2	DAY<br>MONTH<br>YEAR	YES 1<br>NO 2<br>(SKIP TO 220)	AGE IN YEARS	YES 1<br>NO 2	HOUSEHOLD LINE NUMBER<br>(SKIP TO 221)	DAYS 1<br>MONTHS 2<br>YEARS 3	YES 1 (ADD BIRTH)<br>NO 2 (NEXT BIRTH)
10	BOY 1<br>GIRL 2	SING 1<br>MULT 2	DAY<br>MONTH<br>YEAR	YES 1<br>NO 2<br>(SKIP TO 220)	AGE IN YEARS	YES 1<br>NO 2	HOUSEHOLD LINE NUMBER<br>(SKIP TO 221)	DAYS 1<br>MONTHS 2<br>YEARS 3	YES 1 (ADD BIRTH)<br>NO 2 (NEXT BIRTH)

SECTION 2. REPRODUCTION

NO.	QUESTIONS AND FILTERS	CODING CATEGORIES	SKIP
222	Have you had any live births since the birth of (NAME OF LAST BIRTH)?	YES . . . . . 1 (RECORD BIRTH(S) IN TABLE)<br>NO 2	
223	COMPARE 208 WITH NUMBER OF BIRTHS IN BIRTH HISTORY<br>NUMBERS ARE SAME ☐<br>NUMBERS ARE DIFFERENT ☐ (PROBE AND RECONCILE)		
224	CHECK 215: ENTER THE NUMBER OF BIRTHS IN 2010-2015	NUMBER OF BIRTHS . . . . . ☐<br>NONE 0	0 → 226
225	C FOR EACH BIRTH IN 2010-2015, ENTER 'B' IN THE MONTH OF BIRTH IN THE CALENDAR. WRITE THE NAME OF THE CHILD TO THE LEFT OF THE 'B' CODE. FOR EACH BIRTH, ASK THE NUMBER OF COMPLETED MONTHS THE PREGNANCY LASTED AND RECORD 'P' IN EACH OF THE PRECEDING MONTHS ACCORDING TO THE DURATION OF PREGNANCY. (NOTE: THE NUMBER OF 'P's MUST BE ONE LESS THAN THE NUMBER OF MONTHS THAT THE PREGNANCY LASTED.)		
226	Are you pregnant now?	YES . . . . . 1<br>NO . . . . . 2<br>UNSURE 8	2, 8 → 230
227	How many months pregnant are you?<br>RECORD NUMBER OF COMPLETED MONTHS.<br>C ENTER 'P's IN THE CALENDAR, BEGINNING WITH THE MONTH OF INTERVIEW AND FOR THE TOTAL NUMBER OF COMPLETED MONTHS.	MONTHS . . . . . ☐☐	
228	When you got pregnant, did you want to get pregnant at that time?	YES . . . . . 1<br>NO 2	1 → 230
229	CHECK 208: TOTAL NUMBER OF BIRTHS<br>ONE OR MORE ☐ a) Did you want to have a baby later on or did you not want any more children?<br>NONE ☐ b) Did you want to have a baby later on or did you not want any children?	LATER . . . . . 1<br>NO MORE/NONE 2	
230	Have you ever had a pregnancy that miscarried, was aborted, or ended in a stillbirth?	YES . . . . . 1<br>NO 2	2 → 239
230A	I will now ask you about each of them separately.<br>IF NONE, RECORDE "00'<br>01 In total, how many miscarriages have you had?<br>02 In total, how many abortions have you had? Please, also include abortions induced by cytotec or other medicines/herbs with abortive effect conducted at home or elswhere by yourself or with a help of a health professional.<br>03 In total, how many stillbirths have you had?	01. TOTAL MISCARRIAGES . . . . . ☐☐<br>02. TOTAL INDUCED ABORTIONS . . ☐☐<br>03. TOTAL STILLBIRTHS . . . . . ☐☐	
231	When did the last such pregnancy end?	MONTH . . . . . ☐☐<br>YEAR . . . . . ☐☐☐☐	

NO.	QUESTIONS AND FILTERS	CODING CATEGORIES		SKIP
232	CHECK 231: LAST PREGNANCY ENDED IN 2010-2015 ☐			→ 234
		LAST PREGNANCY ENDED IN 2009 OR EARLIER ☐		→ 239

LINE NO.	233<br>In what month and year did the preceding such pregnancy end?	234<br>How many months pregnant were you when that pregnancy ended?	235<br>Since January 2010, have you had any other pregnancies that did not result in a live birth?	
01		NUMBER OF MONTHS	YES . . . . 1<br>NO 2	→ NEXT LINE<br>→ 236
02	MONTH YEAR	NUMBER OF MONTHS	YES . . . . 1<br>NO 2	→ NEXT LINE<br>→ 236
03	MONTH YEAR	NUMBER OF MONTHS	YES . . . . 1<br>NO 2	→ NEXT LINE<br>→ 236
04	MONTH YEAR	NUMBER OF MONTHS	YES . . . . 1<br>NO 2	→ 236

NO.	QUESTIONS AND FILTERS	CODING CATEGORIES	SKIP
236	**C** FOR EACH PREGNANCY THAT DID NOT END IN A LIVE BIRTH IN 2010-2015 OR LATER, ENTER 'T' IN THE CALENDAR IN THE MONTH THAT THE PREGNANCY TERMINATED AND 'P' FOR THE REMAINING NUMBER OF COMPLETED MONTHS OF PREGNANCY.IF THERE ARE MORE THAN FOUR PREGNANCIES THAT DID NOT END IN A LIVE BIRTH, USE AN ADDITIONAL QUESTIONNAIRE STARTING ON THE SECOND LINE.		
237	Did you have any miscarriages, abortions or stillbirths that ended before 2010?	YES . . . . 1<br>NO 2	 → 239
238	When did the last such pregnancy that terminated before 2010 end?	MONTH . . . .<br>YEAR	

SECTION 2. REPRODUCTION

NO.	QUESTIONS AND FILTERS	CODING CATEGORIES	SKIP
239	When did your last menstrual period start?<br><br>______________________<br>(DATE, IF GIVEN)	DAYS AGO . . . . 1 [ ][ ]<br>WEEKS AGO . . . . 2 [ ][ ]<br>MONTHS AGO . . . . 3 [ ][ ]<br>YEARS AGO . . . . 4 [ ][ ]<br><br>IN MENOPAUSE/<br>HAS HAD HYSTERECTOMY . . . . 994<br><br>BEFORE LAST BIRTH . . . . 995<br><br>NEVER MENSTRUATED 996	
240	From one menstrual period to the next, are there certain days when a woman is more likely to become pregnant?	YES . . . . 1<br>NO . . . . 2<br>DON'T KNOW 8	2, 8 → 242
241	Is this time just before her period begins, during her period, right after her period has ended, or halfway between two periods?	JUST BEFORE HER PERIOD BEGINS . . . . 1<br>DURING HER PERIOD . . . . 2<br>RIGHT AFTER HER PERIOD HAS ENDED . . . . . 3<br>HALFWAY BETWEEN TWO PERIODS . . . . 4<br><br>OTHER ______________________ 6<br>(SPECIFY)<br>DON'T KNOW 8	
242	After the birth of a child, can a woman become pregnant before her menstrual period has returned?	YES . . . . 1<br>NO . . . . 2<br>DON'T KNOW 8	

SECTION 3. CONTRACEPTION

301	Now I would like to talk about family planning - the various ways or methods that a couple can use to delay or avoid a pregnancy. Have you ever heard of (METHOD)?	
01	Female Sterilization.<br>PROBE: Women can have an operation to avoid having any more children.	YES . . . . 1<br>NO 2
02	Male Sterilization.<br>PROBE: Men can have an operation to avoid having any more children.	YES . . . . 1<br>NO 2
03	IUD.<br>PROBE: Women can have a loop or coil placed inside them by a doctor or a nurse which can prevent pregnancy for one or more years.	YES . . . . 1<br>NO 2
04	Injectables.<br>PROBE: Women can have an injection by a health provider that stops them from becoming pregnant for one or more months.	YES . . . . 1<br>NO 2
05	Implants.<br>PROBE: Women can have one or more small rods placed in their upper arm by a doctor or nurse which can prevent pregnancy for one or more years.	YES . . . . 1<br>NO 2
06	Pill.<br>PROBE: Women can take a pill every day to avoid becoming pregnant.	YES . . . . 1<br>NO 2
07	Condom.<br>PROBE: Men can put a rubber sheath on their penis before sexual intercourse.	YES . . . . 1<br>NO 2
08	Female Condom.<br>PROBE: Women can place a sheath in their vagina before sexual intercourse.	YES . . . . 1<br>NO 2
09	Emergency Contraception.<br>PROBE: As an emergency measure, within three days after they have unprotected sexual intercourse, women can take special pills to prevent pregnancy.	YES . . . . 1<br>NO 2
10	Standard Days Method.<br>PROBE: A woman uses a string of colored beads to know the days she can get pregnant. On the days she can get pregnant, she uses a condom or does not have sexual intercourse.	YES . . . . 1<br>NO 2
11	Lactational Amenorrhea Method (LAM).<br>PROBE: Up to six months after childbirth, before the menstrual period has returned, women use a method requiring frequent breastfeeding day and night.	YES . . . . 1<br>NO 2
12	Rhythm Method.<br>PROBE: To avoid pregnancy, women do not have sexual intercourse on the days of the month they think they can get pregnant.	YES . . . . 1<br>NO 2
13	Withdrawal.<br>PROBE: Men can be careful and pull out before climax.	YES . . . . 1<br>NO 2
14	Have you heard of any other ways or methods that women or men can use to avoid pregnancy?	YES, MODERN METHOD<br>________________ 1<br>(SPECIFY)<br>YES, TRADITIONAL METHOD<br>________________ 2<br>(SPECIFY)<br>NO 3

SECTION 3. CONTRACEPTION

NO.	QUESTIONS AND FILTERS	CODING CATEGORIES	SKIP
302	CHECK 226:<br>NOT PREGNANT OR UNSURE ☐ ↓	PREGNANT ☐	→ 312
303	Are you or your partner currently doing something or using any method to delay or avoid getting pregnant?	YES . . . . . 1<br>NO 2	 → 312
304	Which method are you using?<br><br>RECORD ALL MENTIONED.<br><br>IF MORE THAN ONE METHOD MENTIONED, FOLLOW SKIP INSTRUCTION FOR HIGHEST METHOD IN LIST.	FEMALE STERILIZATION . . . . . A<br>MALE STERILIZATION . . . . . B<br>IUD . . . . . C<br>INJECTABLES . . . . . D<br>IMPLANTS . . . . . E<br>PILL . . . . . F<br>CONDOM . . . . . G<br>FEMALE CONDOM . . . . . H<br>EMERGENCY CONTRACEPTION . . . . . I<br>STANDARD DAYS METHOD . . . . . J<br>LACTATIONAL AMENORRHEA METHOD . . . . . K<br>RHYTHM METHOD . . . . . L<br>WITHDRAWAL . . . . . M<br>OTHER MODERN METHOD . . . . . X<br>OTHER TRADITIONAL METHOD . . . . . Y	A–B → 307<br>C–E → 309<br><br>G → 306<br>H–Y → 309
305	What is the brand name of the pills you are using?<br><br>IF DON'T KNOW THE BRAND, ASK TO SEE THE PACKAGE.	LOFEMINOL . . . . . 01<br>MICROGYNON . . . . . 02<br>OVRETTE . . . . . 03<br><br>OTHER ______________ 96<br>(SPECIFY)<br>DON'T KNOW 98	→ 309
306	What is the brand name of the condoms you are using?<br><br>IF DON'T KNOW THE BRAND, ASK TO SEE THE PACKAGE.	CHISHANGO . . . . . 01<br>MANYUCHI . . . . . 02<br>SILVERTOUCH . . . . . 03<br>CARE(FEMALE CONDOMS) . . . . . 04<br>PUBLIC SECTOR CONDOMS . . . . . 05<br><br>OTHER ______________ 96<br>(SPECIFY)<br>DON'T KNOW 98	→ 309

NO.	QUESTIONS AND FILTERS	CODING CATEGORIES	SKIP
307	In what facility did the sterilization take place?<br><br>PROBE TO IDENTIFY THE TYPE OF SOURCE.<br><br>IF UNABLE TO DETERMINE IF PUBLIC OR PRIVATE SECTOR, WRITE THE NAME OF THE PLACE.<br><br>__________<br>(NAME OF PLACE)	**PUBLIC SECTOR**<br>GOVERNMENT HOSPITAL . . . . . 11<br>GOVERNMENT HEALTH CENTER . . . . . 12<br>OTHER PUBLIC SECTOR<br>__________ 16<br>(SPECIFY)<br><br>**CHAM/MISSION**<br>HOSPITAL . . . . . 21<br>HEALTH CENTER . . . . . 22<br><br>**PRIVATE MEDICAL SECTOR**<br>PRIVATE HOSPITAL/CLINIC . . . . . 31<br>PRIVATE DOCTOR'S OFFICE . . . . . 32<br>OTHER PRIVATE MEDICAL SECTOR<br>__________ 36<br>(SPECIFY)<br><br>**BLM** . . . . . 41<br><br>OTHER __________ 96<br>(SPECIFY)<br>DON'T KNOW 98	
308	In what month and year was the sterilization performed?	MONTH . . . . . [ ][ ]<br>YEAR . . . . . [ ][ ][ ][ ]	→ 310
309	Since what month and year have you been using (CURRENT METHOD) without stopping?<br><br>PROBE: For how long have you been using (CURRENT METHOD) now without stopping?	MONTH . . . . . [ ][ ]<br>YEAR . . . . . [ ][ ][ ][ ]	
310	CHECK 308 AND 309, 215 AND 231: ANY BIRTH OR PREGNANCY TERMINATION AFTER MONTH AND YEAR OF START OF USE OF CONTRACEPTION IN 308 OR 309<br><br>NO [ ] ↓<br><br>YES [ ] → GO BACK TO 308 OR 309, PROBE AND RECORD MONTH AND YEAR AT START OF CONTINUOUS USE OF CURRENT METHOD (MUST BE AFTER LAST BIRTH OR PREGNANCY TERMINATION).		

311	CHECK 308 AND 309:<br>YEAR IS 2010-2015 ☐<br>C ENTER CODE FOR METHOD USED IN MONTH OF INTERVIEW IN THE CALENDAR AND IN EACH MONTH BACK TO THE DATE STARTED USING.<br>THEN CONTINUE<br><br>YEAR IS 2009 OR EARLIER ☐<br>C ENTER CODE FOR METHOD USED IN MONTH OF INTERVIEW IN THE CALENDAR AND EACH MONTH BACK TO JANUARY 2010 .<br>THEN (SKIP TO 324)			
312	I would like to ask you some questions about the times you or your partner may have used a method to avoid getting pregnant during the last few years.<br>C USE CALENDAR TO PROBE FOR EARLIER PERIODS OF USE AND NONUSE, STARTING WITH MOST RECENT USE, BACK TO JANUARY 2010. USE NAMES OF CHILDREN, DATES OF BIRTH, AND PERIODS OF PREGNANCY AS REFERENCE POINTS.			
		COLUMN 1	COLUMN 2	COLUMN 3
312A	MONTH AND YEAR OF START OF INTERVAL OF USE OR NON-USE.	MONTH ☐☐<br>☐☐☐☐<br>YEAR	MONTH ☐☐<br>☐☐☐☐<br>YEAR	MONTH ☐☐<br>☐☐☐☐<br>YEAR
312B	Between (EVENT) in (MONTH/YEAR) and (EVENT) in (MONTH/YEAR), did you or your partner use any method of contraception?	YES . . . . 1<br>NO . . . . 2<br>(SKIP TO 312I)	YES . . . . 1<br>NO . . . . 2<br>(SKIP TO 312I)	YES . . . . 1<br>NO . . . . 2<br>(SKIP TO 312I)
312C	Which method was that?	METHOD CODE . . ☐	METHOD CODE . . ☐	METHOD CODE . . ☐
312D	How many months after (EVENT) in (MONTH/YEAR) did you start to use (METHOD)?<br><br>CIRCLE '95' IF RESPONDENT GIVES THE DATE OF STARTING TO USE THE METHOD.	IMMEDIATELY . . . . . 00<br>MONTHS . . ☐☐<br>(SKIP TO 312F)<br>DATE GIVEN 95	IMMEDIATELY . . . . . 00<br>MONTHS . . ☐☐<br>(SKIP TO 312F)<br>DATE GIVEN 95	IMMEDIATELY . . . . . 00<br>MONTHS . . ☐☐<br>(SKIP TO 312F)<br>DATE GIVEN 95
312E	RECORD MONTH AND YEAR RESPONDENT STARTED USING METHOD.	MONTH ☐☐<br>☐☐☐☐<br>YEAR	MONTH ☐☐<br>☐☐☐☐<br>YEAR	MONTH ☐☐<br>☐☐☐☐<br>YEAR
312F	For how many months did you use (METHOD)?<br>CIRCLE '95' IF RESPONDENT GIVES THE DATE OF TERMINATION OF USE.	MONTHS . . ☐☐<br>(SKIP TO 312H)<br>DATE GIVEN 95	MONTHS . . ☐☐<br>(SKIP TO 312H)<br>DATE GIVEN 95	MONTHS . . ☐☐<br>(SKIP TO 312H)<br>DATE GIVEN 95
312G	RECORD MONTH AND YEAR RESPONDENT STOPPED USING METHOD.	MONTH ☐☐<br>☐☐☐☐<br>YEAR	MONTH ☐☐<br>☐☐☐☐<br>YEAR	MONTH ☐☐<br>☐☐☐☐<br>YEAR
312H	Why did you stop using (METHOD)?	REASON STOPPED . . . . . ☐	REASON STOPPED . . . . . ☐	REASON STOPPED . . . . . ☐
312I		GO BACK TO 312A IN NEXT COLUMN; OR, IF NO MORE GAPS, GO TO 313.	GO BACK TO 312A IN NEXT COLUMN; OR, IF NO MORE GAPS, GO TO 313.	GO BACK TO 312A IN NEW QUESTIONNAIRE; OR, IF NO MORE GAPS, GO TO 313.

SECTION 3. CONTRACEPTION

NO.	QUESTIONS AND FILTERS	CODING CATEGORIES	SKIP
313	CHECK THE CALENDAR FOR USE OF ANY CONTRACEPTIVE METHOD IN ANY MONTH<br>NO METHOD USED ☐ ↓ ANY METHOD USED ☐		→ 315
314	Have you ever used anything or tried in any way to delay or avoid getting pregnant?	YES . . . 1<br>NO . . . 2	→ 326
315	CHECK 304:<br><br>CIRCLE METHOD CODE:<br><br>IF MORE THAN ONE METHOD CODE CIRCLED IN 304, CIRCLE CODE FOR HIGHEST METHOD IN LIST.	NO CODE CIRCLED . . . 00<br>FEMALE STERILIZATION . . . 01<br>MALE STERILIZATION . . . 02<br>IUD . . . 03<br>INJECTABLES . . . 04<br>IMPLANTS . . . 05<br>PILL . . . 06<br>CONDOM . . . 07<br>FEMALE CONDOM . . . 08<br>EMERGENCY CONTRACEPTION . . . 09<br>STANDARD DAYS METHOD . . . 10<br>LACTATIONAL AMENORRHEA METHOD . . . 11<br>RHYTHM METHOD . . . 12<br>WITHDRAWAL . . . 13<br>OTHER MODERN METHOD . . . 95<br>OTHER TRADITIONAL METHOD . . . 96	→ 326<br>→ 319<br>→ 327<br><br><br><br><br><br><br><br><br>11–13 → 323
316	You first started using (CURRENT METHOD) in (DATE FROM 308 OR 309). Where did you get it at that time?<br><br>PROBE TO IDENTIFY THE TYPE OF SOURCE.<br><br>IF UNABLE TO DETERMINE IF PUBLIC OR PRIVATE SECTOR, WRITE THE NAME OF THE PLACE.<br><br>__________<br>(NAME OF PLACE)	**PUBLIC SECTOR**<br>GOVERNMENT HOSPITAL . . . 11<br>GOVERNMENT HEALTH CENTER . . . 12<br>GOVERNMENT HEALTH POST/ OUTREACH . . . 13<br>MOBILE CLINIC . . . 14<br>HSA . . . 15<br>CBDA/DOOR TO DOOR . . . 16<br>OTHER PUBLIC SECTOR<br>__________ 17<br>(SPECIFY)<br><br>**CHAM/MISSION**<br>HOSPITAL . . . 21<br>HEALTH CENTER . . . 22<br>MOBILE CLINIC . . . 23<br>CBDA/DOOR TO DOOR . . . 24<br><br>**PRIVATE MEDICAL SECTOR**<br>PRIVATE HOSPITAL/CLINIC . . . 31<br>PHARMACY . . . 32<br>PRIVATE DOCTOR . . . 33<br>MOBILE CLINIC . . . 34<br>CBDA/DOOR TO DOOR . . . 35<br>OTHER PRIVATE MEDICAL SECTOR<br>__________ 36<br>(SPECIFY)<br><br>**BLM** . . . 41<br><br>**MACRO** . . . 51<br><br>**YOUTH DROP IN CENTRE** . . . 61<br><br>**OTHER SOURCE**<br>SHOP . . . 71<br>CHURCH . . . 72<br>FRIEND/RELATIVE . . . 73<br><br>OTHER __________ 96<br>(SPECIFY)	
317	CHECK 304:<br><br>CIRCLE METHOD CODE:<br><br>IF MORE THAN ONE METHOD CODE CIRCLED IN 304, CIRCLE CODE FOR HIGHEST METHOD IN LIST.	IUD . . . 03<br>INJECTABLES . . . 04<br>IMPLANTS . . . 05<br>PILL . . . 06<br>CONDOM . . . 07<br>FEMALE CONDOM . . . 08<br>EMERGENCY CONTRACEPTION . . . 09<br>STANDARD DAYS METHOD . . . 10<br>OTHER MODERN METHOD . . . 95<br>OTHER TRADITIONAL METHOD . . . 96	 <br><br><br>→ 323<br>08–95 → 322<br><br><br><br>→ 323

NO.	QUESTIONS AND FILTERS	CODING CATEGORIES	SKIP
318	At that time, were you told about side effects or problems you might have with the method?	YES . . . . 1<br>NO 2	→ 321<br>→ 320
319	When you got sterilized, were you told about side effects or problems you might have with the method?	YES . . . . 1<br>NO 2	→ 321
320	Were you ever told by a health or family planning worker about side effects or problems you might have with the method?	YES . . . . 1<br>NO 2	 → 322
321	Were you told what to do if you experienced side effects or problems?	YES . . . . 1<br>NO 2	
322	CHECK 318 AND 319:<br>ANY 'YES' ☐ ↓ / OTHER ☐ ↓<br>a) At that time, were you told about other methods of family planning that you could use?<br>b) When you obtained (CURRENT METHOD FROM 315) from (SOURCE OF METHOD FROM 307 OR 316), were you told about other methods of family planning that you could use?	YES . . . . 1<br>NO 2	→ 324
323	Were you ever told by a health or family planning worker about other methods of family planning that you could use?	YES . . . . 1<br>NO 2	
324	CHECK 304:<br><br>CIRCLE METHOD CODE:<br><br>IF MORE THAN ONE METHOD CODE CIRCLED IN 304, CIRCLE CODE FOR HIGHEST METHOD IN LIST.	FEMALE STERILIZATION . . . . 01<br>MALE STERILIZATION . . . . 02<br>IUD . . . . 03<br>INJECTABLES . . . . 04<br>IMPLANTS . . . . 05<br>PILL . . . . 06<br>CONDOM . . . . 07<br>FEMALE CONDOM . . . . 08<br>EMERGENCY CONTRACEPTION . . . . 09<br>STANDARD DAYS METHOD . . . . 10<br>LACTATIONAL AMENORRHEA METHOD . . . . 11<br>RHYTHM METHOD . . . . 12<br>WITHDRAWAL . . . . 13<br>OTHER MODERN METHOD . . . . 95<br>OTHER TRADITIONAL METHOD 96	⌉ → 327 (01, 02)<br><br><br><br><br><br><br><br><br><br>⌉ → 327 (11, 12, 13)<br><br><br><br>→ 327 (96)

SECTION 3. CONTRACEPTION

NO.	QUESTIONS AND FILTERS	CODING CATEGORIES	SKIP
325	Where did you obtain (CURRENT METHOD) the last time?<br><br>PROBE TO IDENTIFY THE TYPE OF SOURCE.<br><br>IF UNABLE TO DETERMINE IF PUBLIC OR PRIVATE SECTOR, WRITE THE NAME OF THE PLACE.<br><br>_____________________<br>(NAME OF PLACE)	**PUBLIC SECTOR**<br>GOVERNMENT HOSPITAL . . . . 11<br>GOVERNMENT HEALTH CENTER . . . . 12<br>GOVERNMENT HEALTH POST/ OUTREACH . . . . 13<br>MOBILE CLINIC . . . . 14<br>HSA . . . . 15<br>CBDA/DOOR TO DOOR . . . . 16<br>OTHER PUBLIC SECTOR<br>_____________________ 17<br>(SPECIFY)<br><br>**CHAM/MISSION**<br>HOSPITAL . . . . 21<br>HEALTH CENTER . . . . 22<br>MOBILE CLINIC . . . . 23<br>CBDA/DOOR TO DOOR . . . . 24<br><br>**PRIVATE MEDICAL SECTOR**<br>PRIVATE HOSPITAL/CLINIC . . . . 31<br>PHARMACY . . . . 32<br>PRIVATE DOCTOR . . . . 33<br>MOBILE CLINIC . . . . 34<br>CBDA/DOOR TO DOOR . . . . 35<br>OTHER PRIVATE MEDICAL SECTOR<br>_____________________ 36<br>(SPECIFY)<br><br>**BLM** . . . . 41<br><br>**MACRO** . . . . 51<br><br>**YOUTH DROP IN CENTRE** . . . . 61<br><br>**OTHER SOURCE**<br>SHOP . . . . 71<br>CHURCH . . . . 72<br>FRIEND/RELATIVE . . . . 73<br><br>OTHER _____________________ 96<br>(SPECIFY)	→ 327
326	Do you know of a place where you can obtain a method of family planning?	YES . . . . 1<br>NO 2	
327	In the last 12 months, were you visited by a fieldworker?	YES . . . . 1<br>NO 2	 → 329
328	Did the fieldworker talk to you about family planning?	YES . . . . 1<br>NO 2	
329	CHECK 202: LIVING CHILDREN<br>YES ↓ / NO ↓<br>a) In the last 12 months, have you visited a health facility for care for yourself or your children?<br>b) In the last 12 months, have you visited a health facility for care for yourself?	YES . . . . 1<br>NO 2	 → 401
330	Did any staff member at the health facility speak to you about family planning methods?	YES . . . . 1<br>NO 2	

SECTION 4. PREGNANCY AND POSTNATAL CARE

401	CHECK 224:<br>ONE OR MORE BIRTHS IN 2010-2015 ☐<br>NO BIRTHS IN 2010-2015 ☐ → 648		
402	CHECK 215. RECORD THE BIRTH HISTORY NUMBER IN 403 AND THE NAME AND SURVIVAL STATUS IN 404 FOR EACH BIRTH IN 2010-2015. ASK THE QUESTIONS ABOUT ALL OF THESE BIRTHS. BEGIN WITH THE LAST BIRTH.IF THERE ARE MORE THAN 2 BIRTHS, USE LAST COLUMN OF ADDITIONAL QUESTIONNAIRE(S).<br>Now I would like to ask some questions about your children born in the last five years. (We will talk about each separately.)		
403	BIRTH HISTORY NUMBER FROM 212 IN BIRTH HISTORY.	LAST BIRTH<br>BIRTH HISTORY NUMBER	NEXT-TO-LAST BIRTH<br>BIRTH HISTORY NUMBER . . . .
404	FROM 212 AND 216:	NAME ____<br>LIVING ☐ DEAD ☐	NAME ____<br>LIVING ☐ DEAD ☐
405	When you got pregnant with (NAME), did you want to get pregnant at that time?	YES . . . . 1<br>(SKIP TO 408)<br>NO 2	YES . . . . 1<br>(SKIP TO 426)<br>NO 2
406	CHECK 208:<br>ONLY ONE BIRTH ☐ / MORE THAN ONE BIRTH ☐<br>a) Did you want to have a baby later on, or did you not want any children?<br>b) Did you want to have a baby later on, or did you not want any more children?	LATER . . . . 1<br>NO MORE/NONE . . . . 2<br>(SKIP TO 408)	LATER . . . . 1<br>NO MORE/NONE . . . . 2<br>(SKIP TO 426)
407	How much longer did you want to wait?	MONTHS . . . . 1<br>YEARS . . . . 2<br>DON'T KNOW 998	MONTHS . . . . 1<br>YEARS . . . . 2<br>DON'T KNOW 998
408	Did you see anyone for antenatal care for this pregnancy?	YES . . . . 1<br>NO . . . . 2<br>(SKIP TO 414)	
409	Whom did you see?<br>Anyone else?<br>PROBE TO IDENTIFY EACH TYPE OF PERSON AND RECORD ALL MENTIONED.	**HEALTH PERSONNEL**<br>DOCTOR/CLINICAL OFFICER/MEDICAL ASSISTANT . . . . A<br>NURSE/MIDWIFE . . . . B<br>PATIENT ATTENDANT . . . . . C<br>HSA . . . . D<br>**OTHER PERSON**<br>TRADITIONAL BIRTH ATTENDANT . . . . E<br>OTHER ____ X<br>(SPECIFY)	

NO.	QUESTIONS AND FILTERS	LAST BIRTH<br>NAME ______	NEXT-TO-LAST BIRTH<br>NAME ______
410	Where did you receive antenatal care for this pregnancy?<br><br>Anywhere else?<br><br>PROBE TO IDENTIFY THE TYPE OF SOURCE.<br><br>IF UNABLE TO DETERMINE IF PUBLIC OR PRIVATE SECTOR, WRITE THE NAME OF THE PLACE.<br><br>______<br>(NAME OF PLACE)	**HOME**<br>HER HOME . . . . A<br>OTHER HOME . . . . B<br><br>**PUBLIC SECTOR**<br>GOVERNMENT HOSPITAL . . C<br>GOVERNMENT HEALTH CENTER . . . . D<br>GOVERNMENT HEALTH POST . . . . E<br>MOBILE CLINIC . . . . F<br>OTHER PUBLIC SECTOR<br>______ G<br>(SPECIFY)<br><br>**CHAM/MISSION**<br>HOSPITAL . . . . H<br>HEALTH CENTER . . . . I<br><br>**PRIVATE MEDICAL SECTOR**<br>PRIVATE HOSPITAL/ CLINIC . . . . J<br>MOBILE CLINIC . . . . K<br>OTHER PRIVATE MEDICAL SECTOR<br>______ L<br>(SPECIFY)<br><br>**BLM** . . . . M<br><br>OTHER ______ X<br>(SPECIFY)	
411	How many months pregnant were you when you first received antenatal care for this pregnancy?	MONTHS . . . . [ ][ ]<br><br>DON'T KNOW 98	
412	How many times did you receive antenatal care during this pregnancy?	NUMBER OF TIMES . . . . . [ ][ ]<br><br>DON'T KNOW 98	
413	As part of your antenatal care during this pregnancy, were any of the following done at least once:<br><br>a) Was your blood pressure measured?<br>b) Did you give a urine sample?<br>c) Did you give a blood sample?<br>d) Was your height measured?<br>e) Were you weighed?<br>f) Was the fetal heartbeat checked?<br>g) Did you receive information on what foods to eat?	YES NO<br>a) BP . . . . 1 2<br>b) URINE . . . . 1 2<br>c) BLOOD . . . . 1 2<br>d) HEIGHT . . . . 1 2<br>e) WEIGHT . . . . 1 2<br>f) HEART . . . . 1 2<br>g) FOODS 1 2	
414	During this pregnancy, were you given an injection in the arm to prevent the baby from getting tetanus, that is, convulsions after birth?	YES . . . . 1<br>NO . . . . 2<br>(SKIP TO 417)<br>DON'T KNOW 8	
415	During this pregnancy, how many times did you get a tetanus injection?	TIMES . . . . [ ]<br><br>DON'T KNOW 8	
416	CHECK 415:	2 OR MORE TIMES [ ] (SKIP TO 420)<br>OTHER [ ]	

SECTION 4. PREGNANCY AND POSTNATAL CARE

NO.	QUESTIONS AND FILTERS	LAST BIRTH<br>NAME ______	NEXT-TO-LAST BIRTH<br>NAME ______
417	At any time before this pregnancy, did you receive any tetanus injections?	YES . . . . 1<br>NO . . . . 2 (SKIP TO 420)<br>DON'T KNOW . . . . 8 (SKIP TO 420)	
418	Before this pregnancy, how many times did you receive a tetanus injection?<br><br>IF 7 OR MORE TIMES, RECORD '7'.	TIMES . . . . [ ]<br><br>DON'T KNOW 8	
419	CHECK 418:<br>ONLY ONE [ ] ↓ a) How many years ago did you receive that tetanus injection?<br>MORE THAN ONE [ ] ↓ b) How many years ago did you receive the last tetanus injection prior to this pregnancy?	YEARS AGO . . . . [ ][ ]	
420	During this pregnancy, were you given or did you buy any iron tablets?<br><br>SHOW TABLETS.	YES . . . . 1<br>NO . . . . 2 (SKIP TO 422)<br>DON'T KNOW . . . . 8 (SKIP TO 422)	
421	During the whole pregnancy, for how many days did you take the tablets?<br><br>IF ANSWER IS NOT NUMERIC, PROBE FOR APPROXIMATE NUMBER OF DAYS.	DAYS . . . . [ ][ ][ ]<br><br>DON'T KNOW 998	
422	During this pregnancy, did you take any drug for intestinal worms?	YES . . . . 1<br>NO . . . . 2<br>DON'T KNOW 8	
423	During this pregnancy, did you take SP/Fansidar to keep you from getting malaria?	YES . . . . 1<br>NO . . . . 2 (SKIP TO 426)<br>DON'T KNOW . . . . 8 (SKIP TO 426)	
424	How many times did you take SP/Fansidar during this pregnancy?	TIMES . . . . [ ][ ]	
425	Did you get the SP/Fansidar during any antenatal care visit, during another visit to a health facility or from another source?<br><br>IF MORE THAN ONE SOURCE, RECORD THE HIGHEST SOURCE ON THE LIST.	ANTENATAL VISIT . . . . 1<br>ANOTHER FACILITY VISIT . . . . 2<br>OTHER SOURCE 6	
426	When (NAME) was born, was (NAME) very large, larger than average, average, smaller than average, or very small?	VERY LARGE . . . . 1<br>LARGER THAN AVERAGE . . . . 2<br>AVERAGE . . . . 3<br>SMALLER THAN AVERAGE . . . . 4<br>VERY SMALL . . . . 5<br>DON'T KNOW 8	VERY LARGE . . . . 1<br>LARGER THAN AVERAGE . . . . 2<br>AVERAGE . . . . 3<br>SMALLER THAN AVERAGE . . . . 4<br>VERY SMALL . . . . 5<br>DON'T KNOW 8
427	Was (NAME) weighed at birth?	YES . . . . 1<br>NO . . . . 2 (SKIP TO 429)<br>DON'T KNOW . . . . 8 (SKIP TO 429)	YES . . . . 1<br>NO . . . . 2 (SKIP TO 429)<br>DON'T KNOW . . . . 8 (SKIP TO 429)

NO.	QUESTIONS AND FILTERS	LAST BIRTH<br>NAME ______	NEXT-TO-LAST BIRTH<br>NAME ______
428	How much did (NAME) weigh?<br><br>RECORD WEIGHT IN KILOGRAMS FROM HEALTH CARD, IF AVAILABLE.	KG FROM CARD<br>1 [ ] . [ ][ ][ ]<br>KG FROM RECALL<br>2 [ ] . [ ][ ][ ]<br>DON'T KNOW 99998	KG FROM CARD<br>1 [ ] . [ ][ ][ ]<br>KG FROM RECALL<br>2 [ ] . [ ][ ][ ]<br>DON'T KNOW 99998
429	Who assisted with the delivery of (NAME)?<br><br>Anyone else?<br><br>PROBE FOR THE TYPE(S) OF PERSON(S) AND RECORD ALL MENTIONED.<br><br>IF RESPONDENT SAYS NO ONE ASSISTED, PROBE TO DETERMINE WHETHER ANY ADULTS WERE PRESENT AT THE DELIVERY.	**HEALTH PERSONNEL**<br>DOCTOR/CLINICAL OFFICER/MEDICAL ASSISTANT . . . . A<br>NURSE/MIDWIFE . . . . B<br>PATIENT ATTENDANT . . . . . C<br><br>**OTHER PERSON**<br>TRADITIONAL BIRTH ATTENDANT . . . . D<br>RELATIVE/FRIEND . . . . E<br>OTHER ______ X<br>(SPECIFY)<br>NO ONE ASSISTED . . . . Y	**HEALTH PERSONNEL**<br>DOCTOR/CLINICAL OFFICER/MEDICAL ASSISTANT . . . . A<br>NURSE/MIDWIFE . . . . B<br>PATIENT ATTENDANT . . . . . C<br><br>**OTHER PERSON**<br>TRADITIONAL BIRTH ATTENDANT . . . . D<br>RELATIVE/FRIEND . . . . E<br>OTHER ______ X<br>(SPECIFY)<br>NO ONE ASSISTED . . . . Y
430	Where did you give birth to (NAME)?<br><br>PROBE TO IDENTIFY THE TYPE OF SOURCE.<br><br>IF UNABLE TO DETERMINE IF PUBLIC OR PRIVATE SECTOR, WRITE THE NAME OF THE PLACE.<br><br>______<br>(NAME OF PLACE)	**HOME**<br>HER HOME . . . . 11<br>(SKIP TO 434)<br>OTHER HOME . . . . 12<br><br>**PUBLIC SECTOR**<br>GOVERNMENT HOSPITAL . . 21<br>GOVERNMENT HEALTH CENTER . . . . 22<br>GOVERNMENT HEALTH POST/OUTREACH . . . . . 23<br>OTHER PUBLIC SECTOR<br>______ 26<br>(SPECIFY)<br><br>**CHAM/MISSION**<br>HOSPITAL . . . . 31<br>HEALTH CENTER . . . . 32<br><br>**PRIVATE MEDICAL SECTOR**<br>PRIVATE HOSPITAL/ CLINIC . . . . 41<br>OTHER PRIVATE MEDICAL SECTOR<br>______ 46<br>(SPECIFY)<br><br>**BLM** . . . . 51<br><br>OTHER ______ 96<br>(SPECIFY)<br>(SKIP TO 434)	**HOME**<br>HER HOME . . . . 11<br>(SKIP TO 434)<br>OTHER HOME . . . . 12<br><br>**PUBLIC SECTOR**<br>GOVERNMENT HOSPITAL . . 21<br>GOVERNMENT HEALTH CENTER . . . . 22<br>GOVERNMENT HEALTH POST/OUTREACH . . . . . 23<br>OTHER PUBLIC SECTOR<br>______ 26<br>(SPECIFY)<br><br>**CHAM/MISSION**<br>HOSPITAL . . . . 31<br>HEALTH CENTER . . . . 32<br><br>**PRIVATE MEDICAL SECTOR**<br>PRIVATE HOSPITAL/ CLINIC . . . . 41<br>OTHER PRIVATE MEDICAL SECTOR<br>______ 46<br>(SPECIFY)<br><br>**BLM** . . . . 51<br><br>OTHER ______ 96<br>(SPECIFY)<br>(SKIP TO 434)
431	How long after (NAME) was delivered did you stay there?<br><br>IF LESS THAN ONE DAY, RECORD HOURS;<br>IF LESS THAN ONE WEEK, RECORD DAYS.	HOURS . . . . 1 [ ][ ]<br>DAYS . . . . 2 [ ][ ]<br>WEEKS . . . . 3 [ ][ ]<br>DON'T KNOW 998	

SECTION 4. PREGNANCY AND POSTNATAL CARE

NO.	QUESTIONS AND FILTERS	LAST BIRTH<br>NAME ______	NEXT-TO-LAST BIRTH<br>NAME ______
432	Was (NAME) delivered by caesarean, that is, did they cut your belly open to take the baby out?	YES . . . 1<br>NO . . . 2 (SKIP TO 434)	YES . . . 1<br>NO . . . 2 (SKIP TO 434)
433	When was the decision made to have the caesarean section? Was it before or after your labor pains started?	BEFORE . . . 1<br>AFTER . . . 2	BEFORE . . . 1<br>AFTER . . . 2
434	Immediately after the birth, was (NAME) put directly on the bare skin of your chest?	YES . . . 1<br>NO . . . 2<br>DON'T KNOW . . . 8	YES . . . 1<br>NO . . . 2<br>DON'T KNOW . . . 8
434A	CHECK 430: PLACE OF DELIVERY	CODE 11, 12, OR 96 CIRCLED ☐ (SKIP TO 449)<br>OTHER ☐	CODE 11, 12, OR 96 CIRCLED ☐ (SKIP TO 459)<br>OTHER ☐
435	I would like to talk to you about checks on your health after delivery, for example, someone asking you questions about your health or examining you. Did anyone check on your health while you were still in the facility?	YES . . . 1<br>NO . . . 2 (SKIP TO 438)	
436	How long after delivery did the first check take place?<br><br>IF LESS THAN ONE DAY, RECORD HOURS;<br>IF LESS THAN ONE WEEK, RECORD DAYS.	HOURS . . . 1 ☐☐<br>DAYS . . . 2 ☐☐<br>WEEKS . . . 3 ☐☐<br>DON'T KNOW . . . 998	
437	Who checked on your health at that time?<br><br>PROBE FOR MOST QUALIFIED PERSON.	**HEALTH PERSONNEL**<br>DOCTOR/CLINICAL OFFICER/MEDICAL ASSISTANT . . . 11<br>NURSE/MIDWIFE . . . 12<br>PATIENT ATTENDANT . . . 13<br>HSA . . . 14<br><br>**OTHER PERSON**<br>TRADITIONAL BIRTH ATTENDANT . . . 21<br><br>OTHER ______ 96<br>(SPECIFY)	
438	Now I would like to talk to you about checks on (NAME)'s health after delivery – for example, someone examining (NAME), checking the cord, or seeing if (NAME) is OK. Did anyone check on (NAME)'s health while you were still in the facility?	YES . . . 1<br>NO . . . 2<br>DON'T KNOW . . . 8<br>(NO, DON'T KNOW: SKIP TO 441)	

NO.	QUESTIONS AND FILTERS	LAST BIRTH<br>NAME ______	NEXT-TO-LAST BIRTH<br>NAME ______
439	How long after delivery was (NAME)'s health first checked?<br><br>IF LESS THAN ONE DAY, RECORD HOURS;<br>IF LESS THAN ONE WEEK, RECORD DAYS.	HOURS . . . . 1<br>DAYS . . . . 2<br>WEEKS . . . . 3<br>DON'T KNOW 998	
440	Who checked on (NAME)'s health at that time?<br><br>PROBE FOR MOST QUALIFIED PERSON.	**HEALTH PERSONNEL**<br>DOCTOR/CLINICAL OFFICER/MEDICAL ASSISTANT . . . . 11<br>NURSE/MIDWIFE . . . . 12<br>PATIENT ATTENDANT . . . . . 13<br>HSA . . . . 14<br><br>**OTHER PERSON**<br>TRADITIONAL BIRTH ATTENDANT . . . . 21<br><br>OTHER ______ 96<br>(SPECIFY)	
441	Now I want to talk to you about what happened after you left the facility. Did anyone check on your health after you left the facility?	YES . . . . 1<br>NO . . . . 2 (SKIP TO 445)	
442	How long after delivery did that check take place?<br><br>IF LESS THAN ONE DAY, RECORD HOURS;<br>IF LESS THAN ONE WEEK, RECORD DAYS.	HOURS . . . . 1<br>DAYS . . . . 2<br>WEEKS . . . . 3<br>DON'T KNOW 998	
443	Who checked on your health at that time?<br><br>PROBE FOR MOST QUALIFIED PERSON.	**HEALTH PERSONNEL**<br>DOCTOR/CLINICAL OFFICER/MEDICAL ASSISTANT . . . . 11<br>NURSE/MIDWIFE . . . . 12<br>PATIENT ATTENDANT . . . . . 13<br>HSA . . . . 14<br><br>**OTHER PERSON**<br>TRADITIONAL BIRTH ATTENDANT . . . . 21<br><br>OTHER ______ 96<br>(SPECIFY)	

NO.	QUESTIONS AND FILTERS	LAST BIRTH<br>NAME ______	NEXT-TO-LAST BIRTH<br>NAME ______
444	Where did the check take place?<br><br>PROBE TO IDENTIFY THE TYPE OF SOURCE.<br><br>IF UNABLE TO DETERMINE IF PUBLIC OR PRIVATE SECTOR, WRITE THE NAME OF THE PLACE.<br><br>______<br>(NAME OF PLACE)	**HOME**<br>HER HOME . . . . 11<br>OTHER HOME . . . . 12<br><br>**PUBLIC SECTOR**<br>GOVERNMENT HOSPITAL . . 21<br>GOVERNMENT HEALTH CENTER . . . . 22<br>GOVERNMENT HEALTH POST/OUTREACH . . . . . 23<br>OTHER PUBLIC SECTOR<br>______ 26<br>(SPECIFY)<br><br>**CHAM/MISSION**<br>HOSPITAL . . . . 31<br>HEALTH CENTER . . . . 32<br><br>**PRIVATE MEDICAL SECTOR**<br>PRIVATE HOSPITAL/ CLINIC . . . . 41<br>OTHER PRIVATE MEDICAL SECTOR<br>______ 46<br>(SPECIFY)<br><br>**BLM** . . . . 51<br><br>OTHER ______ 96<br>(SPECIFY)	
445	I would like to talk to you about checks on (NAME)'s health after you left (FACILITY IN 430). Did any health care provider or a traditional birth attendant check on (NAME)'s health in the two months after you left (FACILITY IN 430)?	YES . . . . 1<br>NO . . . . 2<br>(SKIP TO 457) ←<br>DON'T KNOW 8	
446	How many hours, days or weeks after the birth of (NAME) did that check take place?<br><br>IF LESS THAN ONE DAY, RECORD HOURS;<br>IF LESS THAN ONE WEEK, RECORD DAYS.	HOURS . . . . 1 [ ][ ]<br>DAYS . . . . 2 [ ][ ]<br>WEEKS . . . . 3 [ ][ ]<br><br>DON'T KNOW 998	
447	Who checked on (NAME)'s health at that time?<br><br>PROBE FOR MOST QUALIFIED PERSON.	**HEALTH PERSONNEL**<br>DOCTOR/CLINICAL OFFICER/MEDICAL ASSISTANT . . . . 11<br>NURSE/MIDWIFE . . . . 12<br>PATIENT ATTENDANT . . . . . 13<br>HSA . . . . 14<br><br>**OTHER PERSON**<br>TRADITIONAL BIRTH ATTENDANT . . . . 21<br><br>OTHER ______ 96<br>(SPECIFY)	

NO.	QUESTIONS AND FILTERS	LAST BIRTH<br>NAME ______	NEXT-TO-LAST BIRTH<br>NAME ______
448	Where did this check of (NAME) take place?<br><br>PROBE TO IDENTIFY THE TYPE OF SOURCE.<br><br>IF UNABLE TO DETERMINE IF PUBLIC OR PRIVATE SECTOR, WRITE THE NAME OF THE PLACE.<br><br>______<br>(NAME OF PLACE)	**HOME**<br>HER HOME . . . . 11<br>OTHER HOME . . . . 12<br><br>**PUBLIC SECTOR**<br>GOVERNMENT HOSPITAL . . 21<br>GOVERNMENT HEALTH CENTER . . . . 22<br>GOVERNMENT HEALTH POST/OUTREACH . . 23<br>OTHER PUBLIC SECTOR<br>______ 26<br>(SPECIFY)<br><br>**CHAM/MISSION**<br>HOSPITAL . . . . 31<br>HEALTH CENTER . . . . 32<br><br>**PRIVATE MEDICAL SECTOR**<br>PRIVATE HOSPITAL/ CLINIC . . . . 41<br>OTHER PRIVATE MEDICAL SECTOR<br>______ 46<br>(SPECIFY)<br><br>**BLM** . . . . 51<br><br>OTHER ______ 96<br>(SPECIFY)<br><br>(SKIP TO 457)	
449	I would like to talk to you about checks on your health after delivery, for example, someone asking you questions about your health or examining you. Did anyone check on your health after you gave birth to (NAME)?	YES . . . . 1<br>NO . . . . 2<br>(SKIP TO 453)	
450	How long after delivery did the first check take place?<br><br>IF LESS THAN ONE DAY, RECORD HOURS;<br>IF LESS THAN ONE WEEK, RECORD DAYS.	HOURS . . . . 1 [ ][ ]<br>DAYS . . . . 2 [ ][ ]<br>WEEKS . . . . 3 [ ][ ]<br><br>DON'T KNOW 998	
451	Who checked on your health at that time?<br><br>PROBE FOR MOST QUALIFIED PERSON.	**HEALTH PERSONNEL**<br>DOCTOR/CLINICAL OFFICER/MEDICAL ASSISTANT . . . . 11<br>NURSE/MIDWIFE . . . . 12<br>PATIENT ATTENDANT . . . . . 13<br>HSA . . . . 14<br><br>**OTHER PERSON**<br>TRADITIONAL BIRTH ATTENDANT . . . . 21<br><br>OTHER ______ 96<br>(SPECIFY)	

NO.	QUESTIONS AND FILTERS	LAST BIRTH<br>NAME ______	NEXT-TO-LAST BIRTH<br>NAME ______
452	Where did this first check take place?<br><br>PROBE TO IDENTIFY THE TYPE OF SOURCE.<br><br>IF UNABLE TO DETERMINE IF PUBLIC OR PRIVATE SECTOR, WRITE THE NAME OF THE PLACE.<br><br>______<br>(NAME OF PLACE)	**HOME**<br>HER HOME . . . . 11<br>OTHER HOME . . . . 12<br><br>**PUBLIC SECTOR**<br>GOVERNMENT HOSPITAL . . 21<br>GOVERNMENT HEALTH CENTER . . . . 22<br>GOVERNMENT HEALTH POST/OUTREACH . . . . . 23<br>OTHER PUBLIC SECTOR<br>______ 26<br>(SPECIFY)<br><br>**CHAM/MISSION**<br>HOSPITAL . . . . 31<br>HEALTH CENTER . . . . 32<br><br>**PRIVATE MEDICAL SECTOR**<br>PRIVATE HOSPITAL/ CLINIC . . . . 41<br>OTHER PRIVATE MEDICAL SECTOR<br>______ 46<br>(SPECIFY)<br><br>**BLM** . . . . 51<br><br>OTHER ______ 96<br>(SPECIFY)	
453	I would like to talk to you about checks on (NAME)'s health after delivery – for example, someone examining (NAME), checking the cord, or seeing if (NAME) is OK. In the two months after (NAME) was born, did any health care provider or a traditional birth attendant check on (NAME)'s health?	YES . . . . 1<br>NO . . . . 2<br>DON'T KNOW . . . . 8<br>(NO/DON'T KNOW: SKIP TO 457)	
454	How many hours, days or weeks after the birth of (NAME) did the first check take place?<br><br>IF LESS THAN ONE DAY, RECORD HOURS;<br>IF LESS THAN ONE WEEK, RECORD DAYS.	HOURS AFTER BIRTH . . . . . 1 [ ][ ]<br>DAYS AFTER BIRTH . . . . . 2 [ ][ ]<br>WEEKS AFTER BIRTH . . . . . 3 [ ][ ]<br><br>DON'T KNOW 998	
455	Who checked on (NAME)'s health at that time?<br><br>PROBE FOR MOST QUALIFIED PERSON.	**HEALTH PERSONNEL**<br>DOCTOR/CLINICAL OFFICER/MEDICAL ASSISTANT . . . . 11<br>NURSE/MIDWIFE . . . . 12<br>PATIENT ATTENDANT . . . . . 13<br>HSA . . . . 14<br><br>**OTHER PERSON**<br>TRADITIONAL BIRTH ATTENDANT . . . . 21<br><br>OTHER ______ 96<br>(SPECIFY)	

NO.	QUESTIONS AND FILTERS	LAST BIRTH<br>NAME ______	NEXT-TO-LAST BIRTH<br>NAME ______
456	Where did this first check of (NAME) take place?<br><br>PROBE TO IDENTIFY THE TYPE OF SOURCE.<br><br>IF UNABLE TO DETERMINE IF PUBLIC OR PRIVATE SECTOR, WRITE THE NAME OF THE PLACE.<br><br>______<br>(NAME OF PLACE)	**HOME**<br>HER HOME . . . . 11<br>OTHER HOME . . . . 12<br><br>**PUBLIC SECTOR**<br>GOVERNMENT HOSPITAL . . 21<br>GOVERNMENT HEALTH CENTER . . . . 22<br>GOVERNMENT HEALTH POST/OUTREACH . . . . 23<br>OTHER PUBLIC SECTOR<br>______ 26<br>(SPECIFY)<br><br>**CHAM/MISSION**<br>HOSPITAL . . . . 31<br>HEALTH CENTER . . . . 32<br><br>**PRIVATE MEDICAL SECTOR**<br>PRIVATE HOSPITAL/ CLINIC . . . . 41<br>OTHER PRIVATE MEDICAL SECTOR<br>______ 46<br>(SPECIFY)<br><br>**BLM** . . . . 51<br><br>OTHER ______ 96<br>(SPECIFY)	
457	During the first two days after (NAME)'s birth, did any health care provider do the following:<br>a) Examine the cord?<br>b) Measure (NAME)'s temperature?<br>c) Counsel you on danger signs for newborns?<br>d) Counsel you on breastfeeding?<br>e) Observe (NAME) breastfeeding?	YES NO DK<br>a) CORD . . . . 1 2 8<br>b) TEMP. . . . . 1 2 8<br>c) SIGNS . . . . 1 2 8<br>d) COUNSEL BREAST-FEED 1 2 8<br>e) OBSERVE BREAST-FEED 1 2 8	
458	Has your menstrual period returned since the birth of (NAME)?	YES . . . . 1 (SKIP TO 460)<br>NO . . . . 2 (SKIP TO 461)	
459	Did your period return between the birth of (NAME) and your next pregnancy?		YES . . . . 1<br>NO . . . . 2 (SKIP TO 463)
460	For how many months after the birth of (NAME) did you not have a period?	MONTHS . . . . [ ][ ]<br><br>DON'T KNOW 98	MONTHS . . . . [ ][ ]<br><br>DON'T KNOW 98
461	CHECK 226: IS RESPONDENT PREGNANT?	NOT PREGNANT [ ]<br>PREGNANT OR UNSURE [ ] (SKIP TO 463)	

NO.	QUESTIONS AND FILTERS	LAST BIRTH<br>NAME ______	NEXT-TO-LAST BIRTH<br>NAME ______
462	Have you had sexual intercourse since the birth of (NAME)?	YES . . . . 1<br>NO . . . . 2<br>(SKIP TO 464)	
463	For how many months after the birth of (NAME) did you not have sexual intercourse?	MONTHS . . . . [ ][ ]<br>DON'T KNOW 98	MONTHS . . . . [ ][ ]<br>DON'T KNOW 98
464	Did you ever breastfeed (NAME)?	YES . . . . 1<br>(SKIP TO 466)<br>NO 2	YES . . . . 1<br>NO 2
465	CHECK 404: IS CHILD LIVING?	LIVING [ ] (SKIP TO 470)<br>DEAD [ ] (GO TO 471)	
466	How long after birth did you first put (NAME) to the breast?<br><br>IF LESS THAN 1 HOUR, RECORD '00' HOURS;<br>IF LESS THAN 24 HOURS, RECORD HOURS;<br>OTHERWISE, RECORD DAYS.	IMMEDIATELY . . . . .000<br>HOURS . . . . 1 [ ][ ]<br>DAYS . . . . 2 [ ][ ]	
467	In the first three days after delivery, was (NAME) given anything to drink other than breast milk?	YES . . . . 1<br>NO 2	
468	CHECK 404: IS CHILD LIVING?	LIVING [ ]<br>DEAD [ ] (GO TO 471)	LIVING [ ]<br>DEAD [ ] (GO TO 471)
469	Are you still breastfeeding (NAME)?	YES . . . . 1<br>NO 2	
470	Did (NAME) drink anything from a bottle with a nipple yesterday or last night?	YES . . . . 1<br>NO . . . . 2<br>DON'T KNOW 8	YES . . . . 1<br>NO . . . . 2<br>DON'T KNOW 8
471		GO BACK TO 405 IN NEXT COLUMN; OR, IF NO MORE BIRTHS, GO TO 501A.	GO BACK TO 405 IN NEXT-TO-LAST COLUMN OF NEW QUESTIONNAIRE; OR, IF NO MORE BIRTHS, GO TO 501A.

SECTION 5A. CHILD IMMUNIZATION (LAST BIRTH)

NO.	QUESTIONS AND FILTERS	CODING CATEGORIES	SKIP
501A	CHECK 215 IN THE BIRTH HISTORY: ANY BIRTHS IN 2012-2015?<br>ONE OR MORE BIRTHS IN 2012-2015 ☐ ↓	NO BIRTHS IN 2012-2015 ☐	→ 601
502A	RECORD THE NAME AND BIRTH HISTORY NUMBER FROM 212 OF THE LAST CHILD BORN IN 2012-2015.<br>NAME OF LAST BIRTH ______________	BIRTH HISTORY NUMBER . . . . ☐☐	
503A	CHECK 216 FOR CHILD:<br>LIVING ☐ ↓	DEAD ☐	→ 501B
504A	Do you have a Health Passport or other document where (NAME)'s vaccinations are written down?	YES, HAS ONLY A HEALTH PASSPORT . . . . 1<br>YES, HAS ONLY AN OTHER DOCUMENT . . . . . 2<br>YES, HAS HEALTH PPT AND OTHER DOCUMENT 3<br>NO, NO HEALTH PPT AND NO OTHER DOCUMEN 4	1 → 507A<br><br>3 → 507A
505A	Did you ever have a Health Passport for (NAME)?	YES . . . . 1<br>NO 2	
506A	CHECK 504A:<br>CODE '2' CIRCLED ☐ ↓	CODE '4' CIRCLED ☐	→ 511A
507A	May I see the Health Passport or other document where (NAME)'s vaccinations are written down?	YES, ONLY HEALTH PPT SEEN . . . . 1<br>YES, ONLY OTHER DOCUMENT SEEN . . . . 2<br>YES, HEALTH PPT AND OTHER DOCUMENT SEE 3<br>NO HEALTH PPT AND NO OTHER DOCUM. SEEN 4	 <br><br>4 → 511A

SECTION 5A. CHILD IMMUNIZATION (LAST BIRTH)

NO.	QUESTIONS AND FILTERS	CODING CATEGORIES	SKIP
	NAME OF LAST BIRTH ____________	BIRTH HISTORY NUMBER . . . . [ ][ ]	
508A	COPY DATES FROM THE HEALTH PASSPORT OR FROM OTHER DOCUMENT.<br>WRITE '44' IN 'DAY' COLUMN IF HEALTH PPT OR OTHER DOCUMENT SHOWS THAT A DOSE WAS GIVEN, BUT NO DATE IS RECORDED.		

	DAY		MONTH		YEAR			
BCG								
ORAL POLIO VACCINE (OPV) 0 (BIRTH DOSE)								
ORAL POLIO VACCINE (OPV) 1								
ORAL POLIO VACCINE (OPV) 2								
ORAL POLIO VACCINE (OPV) 3								
DPT-HEP.B-HIB (PENTAVALENT) 1								
DPT-HEP.B-HIB (PENTAVALENT) 2								
DPT-HEP.B-HIB (PENTAVALENT) 3								
PCV/PNEUMOCOCCAL 1								
PCV/PNEUMOCOCCAL 2								
PCV/PNEUMOCOCCAL 3								
ROTAVIRUS 1								
ROTAVIRUS 2								
MEASLES VACCINE 1								
MEASLES VACCINE 2								
VITAMIN A (MOST RECENT)								

NO.	QUESTIONS AND FILTERS	CODING CATEGORIES	SKIP
509A	CHECK 508A: 'BCG' TO 'MEASLES VACCINE 2' ALL RECORDED?<br>NO [ ] ↓	YES [ ]	→ 525A
510A	In addition to what is recorded on (this document/these documents), did (NAME) receive any other vaccinations, including vaccinations received in campaigns or immunization days or child health days?<br><br>RECORD 'YES' ONLY IF THE RESPONDENT MENTIONS AT LEAST ONE OF THE VACCINATIONS IN 508A THAT ARE NOT RECORDED AS HAVING BEEN GIVEN.	YES . . . . 1<br>(PROBE FOR VACCINATIONS AND WRITE '66' IN THE CORRESPONDING DAY COLUMN IN 508A)<br>(THEN SKIP TO 525A)<br><br>NO . . . . 2<br>DON'T KNOW 8	 <br><br><br><br>→ 525A

SECTION 5A. CHILD IMMUNIZATION (LAST BIRTH)

NO.	QUESTIONS AND FILTERS	CODING CATEGORIES	SKIP
	NAME OF LAST BIRTH ______	BIRTH HISTORY NUMBER . . . . [ ][ ]	
511A	Did (NAME) ever receive any vaccinations to prevent (NAME) from getting diseases, including vaccinations received in campaigns or immunization days or child health days?	YES . . . 1<br>NO . . . 2<br>DON'T KNOW . . . 8	 2, 8 → 525A
512A	Has (NAME) ever received a BCG vaccination against tuberculosis, that is, an injection in the arm or shoulder that usually causes a scar?	YES . . . 1<br>NO . . . 2<br>DON'T KNOW . . . 8	
514A	Has (NAME) ever received oral polio vaccine, that is, about two drops in the mouth to prevent polio?	YES . . . 1<br>NO . . . 2<br>DON'T KNOW . . . 8	 2, 8 → 517A
515A	Did (NAME) receive the first oral polio vaccine in the first two weeks after birth or later?	FIRST TWO WEEKS . . . 1<br>LATER . . . 2	
516A	How many times did (NAME) receive the oral polio vaccine?	NUMBER OF TIMES . . . [ ]	
517A	Has (NAME) ever received a pentavalent vaccination, that is, an injection given in the thigh sometimes at the same time as polio drops?	YES . . . 1<br>NO . . . 2<br>DON'T KNOW . . . 8	 2, 8 → 519A
518A	How many times did (NAME) receive the pentavalent vaccine?	NUMBER OF TIMES . . . [ ]	

SECTION 5A. CHILD IMMUNIZATION (LAST BIRTH)

NO.	QUESTIONS AND FILTERS	CODING CATEGORIES	SKIP
	NAME OF LAST BIRTH ____________	BIRTH HISTORY NUMBER . . . . [ ][ ]	
519A	Has (NAME) ever received a pneumococcal vaccination (PCV), that is, an injection in the thigh to prevent pneumonia?	YES . . . . 1<br>NO . . . . 2<br>DON'T KNOW 8	NO, DON'T KNOW → 521A
520A	How many times did (NAME) receive the pneumococcal vaccine (PCV)?	NUMBER OF TIMES . . . . [ ]	
521A	Has (NAME) ever received a rotavirus vaccination, that is, liquid in the mouth to prevent diarrhea?	YES . . . . 1<br>NO . . . . 2<br>DON'T KNOW 8	NO, DON'T KNOW → 523A
522A	How many times did (NAME) receive the rotavirus vaccine?	NUMBER OF TIMES . . . . [ ]	
523A	Has (NAME) ever received a measles vaccination, that is, an injection in the arm to prevent measles?	YES . . . . 1<br>NO . . . . 2<br>DON'T KNOW 8	NO, DON'T KNOW → 525A
524A	How many times did (NAME) receive the measles vaccine?	NUMBER OF TIMES . . . . [ ]	
525A	In the last 7 days was (NAME) given:<br>a) MULTIPLE MICRONUTRIENT POWDER ?<br>b) READY TO USE THERAPEUTIC FOOD SUCH AS CHIPONDE ?<br>c) SUPPLEMENTARY FOOD SUCH AS LIKUNI PHALA?	YES NO DK<br>a) POWDER . . . . 1 2 8<br>b) CHIPONDE . . . . 1 2 8<br>c) LIKUNI PHALA 1 2 8	
526A	CONTINUE WITH 501B.		

SECTION 5B. CHILD IMMUNIZATION (NEXT-TO-LAST BIRTH)

NO.	QUESTIONS AND FILTERS	CODING CATEGORIES	SKIP
501B	CHECK 215 IN THE BIRTH HISTORY: ANY MORE BIRTHS IN 2012-2015?<br>MORE BIRTHS IN 2012-2015 ☐ ↓ NO MORE BIRTHS IN 2012-2015 ☐ →		601
502B	RECORD THE NAME AND BIRTH HISTORY NUMBER FROM 212 OF THE NEXT-TO-LAST CHILD BORN IN 2012-2015.<br>NAME OF NEXT-TO-LAST BIRTH ____________	BIRTH HISTORY NUMBER . . . . ☐☐	
503B	CHECK 216 FOR CHILD:<br>LIVING ☐ ↓ DEAD ☐ →		526B
504B	Do you have a Health Passport or other document where (NAME)'s vaccinations are written down?	YES, HAS ONLY A HEALTH PASSPORT . . . . 1<br>YES, HAS ONLY AN OTHER DOCUMENT . . . . . 2<br>YES, HAS HEALTH PPT AND OTHER DOCUMENT 3<br>NO, NO HEALTH PPT AND NO OTHER DOCUMEN 4	1 → 507B<br><br>3 → 507B
505B	Did you ever have a Health Passport for (NAME)?	YES . . . . 1<br>NO 2	
506B	CHECK 504B:<br>CODE '2' CIRCLED ☐ ↓ CODE '4' CIRCLED ☐ →		511B
507B	May I see the Health Passport or other document where (NAME)'s vaccinations are written down?	YES, ONLY HEALTH PPT SEEN . . . . 1<br>YES, ONLY OTHER DOCUMENT SEEN . . . . 2<br>YES, HEALTH PPT AND OTHER DOCUMENT SEE 3<br>NO HEALTH PPT AND NO OTHER DOCUM. SEEN 4	 <br><br>4 → 511B

SECTION 5B. CHILD IMMUNIZATION (NEXT-TO-LAST BIRTH)

NO.	QUESTIONS AND FILTERS	CODING CATEGORIES	SKIP
	NAME OF NEXT-TO-LAST BIRTH ____________	BIRTH HISTORY NUMBER . . . . [ ][ ]	
508B	COPY DATES FROM THE HEALTH PASSPORT OR FROM OTHER DOCUMENT. WRITE '44' IN 'DAY' COLUMN IF HEALTH PASSPORT OR OTHER DOCUMENT SHOWS THAT A DOSE WAS GIVEN, BUT NO DATE IS RECORDED.		

	DAY	MONTH	YEAR
BCG			
ORAL POLIO VACCINE (OPV) 0 (BIRTH DOSE)			
ORAL POLIO VACCINE (OPV) 1			
ORAL POLIO VACCINE (OPV) 2			
ORAL POLIO VACCINE (OPV) 3			
DPT-HEP.B-HIB (PENTAVALENT) 1			
DPT-HEP.B-HIB (PENTAVALENT) 2			
DPT-HEP.B-HIB (PENTAVALENT) 3			
PCV/PNEUMOCOCCAL 1			
PCV/PNEUMOCOCCAL 2			
PCV/PNEUMOCOCCAL 3			
ROTAVIRUS 1			
ROTAVIRUS 2			
MEASLES VACCINE 1			
MEASLES VACCINE 2			
VITAMIN A (MOST RECENT)			

NO.	QUESTIONS AND FILTERS	CODING CATEGORIES	SKIP
509B	CHECK 508B: 'BCG' TO 'MEASLES VACCINE 2' ALL RECORDED?<br>NO [ ] ↓	YES [ ] →	525B
510B	In addition to what is recorded on (this document/these documents), did (NAME) receive any other vaccinations, including vaccinations received in campaigns or immunization days or child health days?<br><br>RECORD 'YES' ONLY IF THE RESPONDENT MENTIONS AT LEAST ONE OF THE VACCINATIONS IN 508B THAT ARE NOT RECORDED AS HAVING BEEN GIVEN.	YES . . . . 1<br>(PROBE FOR VACCINATIONS AND WRITE '66' IN THE CORRESPONDING DAY COLUMN IN 508B)<br>(THEN SKIP TO 525B)<br><br>NO . . . . 2<br>DON'T KNOW 8	 <br><br><br>525B

SECTION 5B. CHILD IMMUNIZATION (NEXT-TO-LAST BIRTH)

NO.	QUESTIONS AND FILTERS	CODING CATEGORIES	SKIP
	NAME OF NEXT-TO-LAST BIRTH ______________	BIRTH HISTORY NUMBER . . . . [ ][ ]	
511B	Did (NAME) ever receive any vaccinations to prevent (NAME) from getting diseases, including vaccinations received in campaigns or immunization days or child health days?	YES . . . . . 1<br>NO . . . . . 2<br>DON'T KNOW 8	 2, 8 → 525B
512B	Has (NAME) ever received a BCG vaccination against tuberculosis, that is, an injection in the arm or shoulder that usually causes a scar?	YES . . . . . 1<br>NO . . . . . 2<br>DON'T KNOW 8	
514B	Has (NAME) ever received oral polio vaccine, that is, about two drops in the mouth to prevent polio?	YES . . . . . 1<br>NO . . . . . 2<br>DON'T KNOW 8	 2, 8 → 517B
515B	Did (NAME) receive the first oral polio vaccine in the first two weeks after birth or later?	FIRST TWO WEEKS . . . . . 1<br>LATER 2	
516B	How many times did (NAME) receive the oral polio vaccine?	NUMBER OF TIMES . . . . . [ ]	
517B	Has (NAME) ever received a pentavalent vaccination, that is, an injection given in the thigh sometimes at the same time as polio drops?	YES . . . . . 1<br>NO . . . . . 2<br>DON'T KNOW 8	 2, 8 → 519B
518B	How many times did (NAME) receive the pentavalent vaccine?	NUMBER OF TIMES . . . . . [ ]	

SECTION 5B. CHILD IMMUNIZATION (NEXT-TO-LAST BIRTH)

NO.	QUESTIONS AND FILTERS	CODING CATEGORIES	SKIP
	NAME OF NEXT-TO-LAST BIRTH ______	BIRTH HISTORY NUMBER . . . . [ ][ ]	
519B	Has (NAME) ever received a pneumococcal vaccination (PCV), that is, an injection in the thigh to prevent pneumonia?	YES . . . . . 1<br>NO . . . . . 2<br>DON'T KNOW 8	NO, DON'T KNOW → 521B
520B	How many times did (NAME) receive the pneumococcal vaccine (PCV)?	NUMBER OF TIMES . . . . . [ ]	
521B	Has (NAME) ever received a rotavirus vaccination, that is, liquid in the mouth to prevent diarrhea?	YES . . . . . 1<br>NO . . . . . 2<br>DON'T KNOW 8	NO, DON'T KNOW → 523B
522B	How many times did (NAME) receive the rotavirus vaccine?	NUMBER OF TIMES . . . . . [ ]	
523B	Has (NAME) ever received a measles vaccination, that is, an injection in the arm to prevent measles?	YES . . . . . 1<br>NO . . . . . 2<br>DON'T KNOW 8	NO, DON'T KNOW → 525B
524B	How many times did (NAME) receive the measles vaccine?	NUMBER OF TIMES . . . . . [ ]	
525B	In the last 7 days was (NAME) given:<br>a) MULTIPLE MICRONUTRIENT POWDER ?<br>b) READY TO USE THERAPEUTIC FOOD SUCH AS CHIPONDE ?<br>c) SUPPLEMENTARY FOOD SUCH AS LIKUNI PHALA?	YES NO DK<br>a) POWDER . . . . . 1 2 8<br>b) CHIPONDE . . . . . 1 2 8<br>c) LIKUNI PHALA 1 2 8	
526B	CHECK 215 IN BIRTH HISTORY: ANY MORE BIRTHS IN 2012-2015?<br>MORE BIRTHS IN 2012-2015 [ ] → (GO TO 502B IN AN ADDITIONAL QUESTIONNAIRE)<br>NO MORE BIRTHS IN 2012-2015 [ ] → 601		601

SECTION 6. CHILD HEALTH AND NUTRITION

601	CHECK 224:<br>ONE OR MORE BIRTHS IN 2010-2015 ☐ ↓ NO BIRTHS IN 2010-2015 ☐ → 648		
602	CHECK 215: RECORD THE BIRTH HISTORY NUMBER IN 603 AND THE NAME AND SURVIVAL STATUS IN 604 FOR EACH BIRTH IN 2010-2015. ASK THE QUESTIONS ABOUT ALL OF THESE BIRTHS. BEGIN WITH THE LAST BIRTH.IF THERE ARE MORE THAN 2 BIRTHS, USE LAST COLUMN OF ADDITIONAL QUESTIONNAIRE(S).<br><br>Now I would like to ask some questions about your children born in the last five years. (We will talk about each separately.)		
603	BIRTH HISTORY NUMBER FROM 212 IN BIRTH HISTORY.	LAST BIRTH<br>BIRTH HISTORY NUMBER . . . . ☐☐	NEXT-TO-LAST BIRTH<br>BIRTH HISTORY NUMBER . . . . ☐☐
604	FROM 212 AND 216:	NAME ________<br>LIVING ☐ ↓ DEAD ☐ (SKIP TO 646)	NAME ________<br>LIVING ☐ ↓ DEAD ☐ (SKIP TO 646)
605	In the last six months, was (NAME) given a vitamin A dose like [this/any of these]?<br><br>SHOW COMMON TYPES OF AMPULES/CAPSULES/SYRUPS.	YES . . . . 1<br>NO . . . . 2<br>DON'T KNOW 8	YES . . . . 1<br>NO . . . . 2<br>DON'T KNOW 8
606	In the last seven days, was (NAME) given iron pills, sprinkles with iron, or iron syrup like [this/any of these]?<br><br>SHOW COMMON TYPES OF PILLS/SPRINKLES/SYRUPS.	YES . . . . 1<br>NO . . . . 2<br>DON'T KNOW 8	YES . . . . 1<br>NO . . . . 2<br>DON'T KNOW 8
607	Was (NAME) given any drug for intestinal worms in the last six months?	YES . . . . 1<br>NO . . . . 2<br>DON'T KNOW 8	YES . . . . 1<br>NO . . . . 2<br>DON'T KNOW 8
608	Has (NAME) had diarrhea in the last 2 weeks?	YES . . . . 1<br>NO . . . . 2<br>(SKIP TO 618)<br>DON'T KNOW 8	YES . . . . 1<br>NO . . . . 2<br>(SKIP TO 618)<br>DON'T KNOW 8

SECTION 6. CHILD HEALTH AND NUTRITION

NO.	QUESTIONS AND FILTERS	LAST BIRTH<br>NAME ________	NEXT-TO-LAST BIRTH<br>NAME ________
609	CHECK 464: EVER BREASTFED?<br>YES / NO<br>a) (YES) Now I would like to know how much (NAME) was given to drink during the diarrhea including breastmilk. Was (NAME) given less than usual to drink, about the same amount, or more than usual to drink?<br>IF LESS, PROBE: Was (NAME) given much less than usual to drink or somewhat less?<br>b) (NO) Now I would like to know how much (NAME) was given to drink during the diarrhea. Was (NAME) given less than usual to drink, about the same amount, or more than usual to drink?<br>IF LESS, PROBE: Was (NAME) given much less than usual to drink or somewhat less?	MUCH LESS . . . . . 1<br>SOMEWHAT LESS . . . . . 2<br>ABOUT THE SAME . . . . . 3<br>MORE . . . . . 4<br>NOTHING TO DRINK . . . . . 5<br>DON'T KNOW 8	MUCH LESS . . . . . 1<br>SOMEWHAT LESS . . . . . 2<br>ABOUT THE SAME . . . . . 3<br>MORE . . . . . 4<br>NOTHING TO DRINK . . . . . 5<br>DON'T KNOW 8
610	When (NAME) had diarrhea, was (NAME) given less than usual to eat, about the same amount, more than usual, or nothing to eat?<br>IF LESS, PROBE: Was (NAME) given much less than usual to eat or somewhat less?	MUCH LESS . . . . . 1<br>SOMEWHAT LESS . . . . . 2<br>ABOUT THE SAME . . . . . 3<br>MORE . . . . . 4<br>STOPPED FOOD . . . . . 5<br>NEVER GAVE FOOD . . . . . 6<br>DON'T KNOW 8	MUCH LESS . . . . . 1<br>SOMEWHAT LESS . . . . . 2<br>ABOUT THE SAME . . . . . 3<br>MORE . . . . . 4<br>STOPPED FOOD . . . . . 5<br>NEVER GAVE FOOD . . . . . 6<br>DON'T KNOW 8
611	Did you seek advice or treatment for the diarrhea from any source?	YES . . . . . 1<br>NO . . . . . 2<br>(SKIP TO 615)	YES . . . . . 1<br>NO . . . . . 2<br>(SKIP TO 615)

NO.	QUESTIONS AND FILTERS	LAST BIRTH<br>NAME ______	NEXT-TO-LAST BIRTH<br>NAME ______
611A	How many days after the illness began did you first seek advice or treatment for (NAME)?<br><br>IF THE SAME DAY RECORD '00'.	DAYS . . . . [ ][ ]	DAYS . . . . [ ][ ]
612	Where did you seek advice or treatment?<br><br>Anywhere else?<br><br>PROBE TO IDENTIFY THE TYPE OF SOURCE.<br><br>IF UNABLE TO DETERMINE IF PUBLIC OR PRIVATE SECTOR, WRITE THE NAME OF THE PLACE(S).<br><br>______<br>(NAME OF PLACE(S))	**PUBLIC SECTOR**<br>GOVERNMENT HOSPITAL . . A<br>GOVERNMENT HEALTH CENTER . . . . B<br>GOVERNMENT HEALTH POST/OUTREACH . . . . . C<br>MOBILE CLINIC . . . . D<br>HSA . . . . E<br>OTHER PUBLIC SECTOR ______ F<br>(SPECIFY)<br><br>**CHAM/MISSION**<br>HOSPITAL . . . . G<br>HEALTH CENTER . . . . H<br><br>**PRIVATE MEDICAL SECTOR**<br>PRIVATE HOSPITAL/ CLINIC . . . . I<br>PHARMACY . . . . J<br>PRIVATE DOCTOR . . . . K<br>MOBILE CLINIC . . . . L<br>HSA . . . . M<br>OTHER PRIVATE MEDICAL SECTOR ______ N<br>(SPECIFY)<br><br>**BLM** . . . . O<br><br>**MACRO** . . . . P<br><br>**YOUTH DROP CENTER** . . . . . Q<br><br>**OTHER SOURCE**<br>SHOP . . . . R<br>TRADITIONAL PRACTITIONER . . . . S<br>MARKET . . . . T<br><br>OTHER ______ X<br>(SPECIFY)	**PUBLIC SECTOR**<br>GOVERNMENT HOSPITAL . . A<br>GOVERNMENT HEALTH CENTER . . . . B<br>GOVERNMENT HEALTH POST/OUTREACH . . . . . C<br>MOBILE CLINIC . . . . D<br>HSA . . . . E<br>OTHER PUBLIC SECTOR ______ F<br>(SPECIFY)<br><br>**CHAM/MISSION**<br>HOSPITAL . . . . G<br>HEALTH CENTER . . . . H<br><br>**PRIVATE MEDICAL SECTOR**<br>PRIVATE HOSPITAL/ CLINIC . . . . I<br>PHARMACY . . . . J<br>PRIVATE DOCTOR . . . . K<br>MOBILE CLINIC . . . . L<br>HSA . . . . M<br>OTHER PRIVATE MEDICAL SECTOR ______ N<br>(SPECIFY)<br><br>**BLM** . . . . O<br><br>**MACRO** . . . . P<br><br>**YOUTH DROP CENTER** . . . . . Q<br><br>**OTHER SOURCE**<br>SHOP . . . . R<br>TRADITIONAL PRACTITIONER . . . . S<br>MARKET . . . . T<br><br>OTHER ______ X<br>(SPECIFY)
613	CHECK 612:	TWO OR MORE CODES CIRCLED [ ] ↓<br>ONLY ONE CODE CIRCLED [ ] (SKIP TO 615)	TWO OR MORE CODES CIRCLED [ ] ↓<br>ONLY ONE CODE CIRCLED [ ] (SKIP TO 615)
614	Where did you first seek advice or treatment?<br><br>USE LETTER CODE FROM 612.	FIRST PLACE . . . . [ ]	FIRST PLACE . . . . [ ]

NO.	QUESTIONS AND FILTERS	LAST BIRTH<br>NAME ______	NEXT-TO-LAST BIRTH<br>NAME ______
615	Was (NAME) given any of the following at any time since (NAME) started having the diarrhea:<br>a) A fluid made from a special packet called THANZI-ORS?<br>b) A homemade fluid such as THOBWA?<br>c) Zinc tablets or syrup?	YES NO DK<br>a) FLUID FROM ORS PACKET . . 1 2 8<br>b) HOMEMADE FLUID . . . . . 1 2 8<br>c) ZINC 1 2 8	YES NO DK<br>a) FLUID FROM ORS PACKET . . 1 2 8<br>c) HOMEMADE FLUID . . . . . 1 2 8<br>d) ZINC 1 2 8
616	CHECK 615:<br>ANY 'YES' ☐ / ALL 'NO' OR 'DK' ☐<br>a) Was anything else given to treat the diarrhea?<br>b) Was anything given to treat the diarrhea?	YES . . . . 1<br>NO . . . . 2<br>(SKIP TO 618)<br>DON'T KNOW 8	YES . . . . 1<br>NO . . . . 2<br>(SKIP TO 618)<br>DON'T KNOW 8
617	CHECK 615:<br>ANY 'YES' ☐ / ALL 'NO' OR 'DK' ☐<br>a) What else was given to treat the diarrhea? Anything else?<br>b) What was given to treat the diarrhea? Anything else?<br>RECORD ALL TREATMENTS GIVEN.	**PILL OR SYRUP**<br>ANTIBIOTIC . . . . . . A<br>ANTIMOTILITY . . . . . B<br>OTHER (NOT ANTIBIOTIC OR ANTIMOTILITY) . . . . . C<br>UNKNOWN PILL OR SYRUP . . . . . D<br>**INJECTION**<br>ANTIBIOTIC . . . . . . E<br>NON-ANTIBIOTIC . . . . F<br>UNKNOWN INJECTION . . . . . G<br>(IV) INTRAVENOUS . . . . . H<br>HOME REMEDY/ HERBAL MEDICINE . . . . I<br>OTHER ______ X<br>(SPECIFY)	**PILL OR SYRUP**<br>ANTIBIOTIC . . . . . . A<br>ANTIMOTILITY . . . . . B<br>OTHER (NOT ANTIBIOTIC OR ANTIMOTILITY) . . . . . C<br>UNKNOWN PILL OR SYRUP . . . . . D<br>**INJECTION**<br>ANTIBIOTIC . . . . . . E<br>NON-ANTIBIOTIC . . . . F<br>UNKNOWN INJECTION . . . . . G<br>(IV) INTRAVENOUS . . . . . H<br>HOME REMEDY/ HERBAL MEDICINE . . . . I<br>OTHER ______ X<br>(SPECIFY)
618	Has (NAME) been ill with a fever at any time in the last 2 weeks?	YES . . . . 1<br>NO . . . . 2<br>(SKIP TO 620)<br>DON'T KNOW 8	YES . . . . 1<br>NO . . . . 2<br>(SKIP TO 620)<br>DON'T KNOW 8
619	At any time during the illness, did (NAME) have blood taken from (NAME)'s finger or heel for testing?	YES . . . . 1<br>NO . . . . 2<br>DON'T KNOW 8	YES . . . . 1<br>NO . . . . 2<br>DON'T KNOW 8
620	Has (NAME) had an illness with a cough at any time in the last 2 weeks?	YES . . . . 1<br>NO . . . . 2<br>DON'T KNOW 8	YES . . . . 1<br>NO . . . . 2<br>DON'T KNOW 8
621	Has (NAME) had fast, short, rapid breaths or difficulty breathing at any time in the last 2 weeks?	YES . . . . 1<br>NO . . . . 2<br>(SKIP TO 623)<br>DON'T KNOW 8	YES . . . . 1<br>NO . . . . 2<br>(SKIP TO 623)<br>DON'T KNOW 8

NO.	QUESTIONS AND FILTERS	LAST BIRTH<br>NAME ______	NEXT-TO-LAST BIRTH<br>NAME ______
622	Was the fast or difficult breathing due to a problem in the chest or to a blocked or runny nose?	CHEST ONLY . . . . 1<br>NOSE ONLY . . . . 2<br>BOTH . . . . 3<br>OTHER ______ 6<br>(SPECIFY)<br>DON'T KNOW . . . . 8<br>(SKIP TO 624)	CHEST ONLY . . . . 1<br>NOSE ONLY . . . . 2<br>BOTH . . . . 3<br>OTHER ______ 6<br>(SPECIFY)<br>DON'T KNOW . . . . 8<br>(SKIP TO 624)
623	CHECK 618: HAD FEVER?	YES ☐ NO OR DK ☐ (SKIP TO 646)	YES ☐ NO OR DK ☐ (SKIP TO 646)
624	Did you seek advice or treatment for the illness from any source?	YES . . . . 1<br>NO . . . . 2<br>(SKIP TO 629)	YES . . . . 1<br>NO . . . . 2<br>(SKIP TO 629)
625	Where did you seek advice or treatment?<br><br>Anywhere else?<br><br>PROBE TO IDENTIFY THE TYPE OF SOURCE.<br><br>IF UNABLE TO DETERMINE IF PUBLIC OR PRIVATE SECTOR, WRITE THE NAME OF THE PLACE(S).<br><br>______<br>(NAME OF PLACE(S))	**PUBLIC SECTOR**<br>GOVERNMENT HOSPITAL . . A<br>GOVERNMENT HEALTH CENTER . . . . B<br>GOVERNMENT HEALTH POST/OUTREACH . . . . . C<br>MOBILE CLINIC . . . . D<br>HSA . . . . E<br>OTHER PUBLIC SECTOR<br>______ F<br>(SPECIFY)<br><br>**CHAM/MISSION**<br>HOSPITAL . . . . G<br>HEALTH CENTER . . . . H<br><br>**PRIVATE MEDICAL SECTOR**<br>PRIVATE HOSPITAL/ CLINIC . . . . I<br>PHARMACY . . . . J<br>PRIVATE DOCTOR . . . . K<br>MOBILE CLINIC . . . . L<br>HSA . . . . M<br>OTHER PRIVATE MEDICAL SECTOR<br>______ N<br>(SPECIFY)<br><br>**BLM** . . . . O<br><br>**MACRO** . . . . P<br><br>**YOUTH DROP IN CENTRE** . . Q<br><br>**OTHER SOURCE**<br>SHOP . . . . R<br>TRADITIONAL PRACTITIONER . . . . S<br>MARKET . . . . T<br>ITINERANT DRUG SELLER . . . . U<br><br>OTHER ______ X<br>(SPECIFY)	**PUBLIC SECTOR**<br>GOVERNMENT HOSPITAL . . A<br>GOVERNMENT HEALTH CENTER . . . . B<br>GOVERNMENT HEALTH POST/OUTREACH . . . . . C<br>MOBILE CLINIC . . . . D<br>HSA . . . . E<br>OTHER PUBLIC SECTOR<br>______ F<br>(SPECIFY)<br><br>**CHAM/MISSION**<br>HOSPITAL . . . . G<br>HEALTH CENTER . . . . H<br><br>**PRIVATE MEDICAL SECTOR**<br>PRIVATE HOSPITAL/ CLINIC . . . . I<br>PHARMACY . . . . J<br>PRIVATE DOCTOR . . . . K<br>MOBILE CLINIC . . . . L<br>HSA . . . . M<br>OTHER PRIVATE MEDICAL SECTOR<br>______ N<br>(SPECIFY)<br><br>**BLM** . . . . O<br><br>**MACRO** . . . . P<br><br>**YOUTH DROP IN CENTRE** . . Q<br><br>**OTHER SOURCE**<br>SHOP . . . . R<br>TRADITIONAL PRACTITIONER . . . . S<br>MARKET . . . . T<br>ITINERANT DRUG SELLER . . . . U<br><br>OTHER ______ X<br>(SPECIFY)
626	CHECK 625:	TWO OR MORE CODES CIRCLED ☐ ONLY ONE CODE CIRCLED ☐ (SKIP TO 628)	TWO OR MORE CODES CIRCLED ☐ ONLY ONE CODE CIRCLED ☐ (SKIP TO 628)

NO.	QUESTIONS AND FILTERS	LAST BIRTH<br>NAME ______	NEXT-TO-LAST BIRTH<br>NAME ______
627	Where did you first seek advice or treatment?<br><br>USE LETTER CODE FROM 625.	FIRST PLACE . . . . [ ]	FIRST PLACE . . . . [ ]
628	How many days after the illness began did you first seek advice or treatment for (NAME)?<br><br>IF THE SAME DAY RECORD '00'.	DAYS . . . . [ ][ ]	DAYS . . . . [ ][ ]
629	At any time during the illness, did (NAME) take any drugs for the illness?	YES . . . . 1<br>NO . . . . 2<br>(SKIP TO 646)<br>DON'T KNOW 8	YES . . . . 1<br>NO . . . . 2<br>(SKIP TO 646)<br>DON'T KNOW 8
630	What drugs did (NAME) take?<br><br>Any other drugs?<br><br>RECORD ALL MENTIONED.	**ANTIMALARIAL DRUGS**<br>LA . . . . A<br>ASAQ (COMBINED AMODIAQUINE AND ARTESUNATE) . . . . B<br>SP/FANSIDAR/ NOVIDAR SP . . . . C<br>QUININE<br>TABLETS . . . . D<br>INJECTION/IV . . . . E<br>ARTESUNATE<br>RECTAL . . . . F<br>INJECTION/IV . . . . G<br><br>OTHER ANTIMALARIAL<br>______ H<br>(SPECIFY)<br><br>**ANTIBIOTIC DRUGS**<br>PILL/SYRUP . . . . I<br>INJECTION/IV . . . . J<br><br>**OTHER DRUGS**<br>ASPIRIN/CAFENOL . . . . . K<br>ACETAMINOPHEN/ PANADOL/ PARACETAMOL . . . . . L<br>IBUPROFEN . . . . M<br><br>OTHER ______ X<br>(SPECIFY)<br>DON'T KNOW . . . . Z	**ANTIMALARIAL DRUGS**<br>LA . . . . A<br>AA/ASAQ (COMBINED AMODIAQUINE AND ARTESUNATE) . . . . B<br>SP/FANSIDAR/ NOVIDAR SP . . . . C<br>QUININE<br>TABLETS . . . . D<br>INJECTION/IV . . . . E<br>ARTESUNATE<br>RECTAL . . . . F<br>INJECTION/IV . . . . G<br><br>OTHER ANTIMALARIAL<br>______ H<br>(SPECIFY)<br><br>**ANTIBIOTIC DRUGS**<br>PILL/SYRUP . . . . I<br>INJECTION/IV . . . . J<br><br>**OTHER DRUGS**<br>ASPIRIN/CAFENOL . . . . . K<br>ACETAMINOPHEN/ PANADOL/ PARACETAMOL . . . . . L<br>IBUPROFEN . . . . M<br><br>OTHER ______ X<br>(SPECIFY)<br>DON'T KNOW . . . . Z
631	CHECK 630:<br>ANY CODE A-H CIRCLED?	YES [ ] NO [ ]<br>(SKIP TO 646)	YES [ ] NO [ ]<br>(SKIP TO 646)

NO.	QUESTIONS AND FILTERS	LAST BIRTH<br>NAME ____________	NEXT-TO-LAST BIRTH<br>NAME ____________
632	CHECK 630:<br>LA ('A') GIVEN	CODE 'A' CIRCLED ☐ ↓<br>CODE 'A' NOT CIRCLED ☐ (SKIP TO 634)	CODE 'A' CIRCLED ☐ ↓<br>CODE 'A' NOT CIRCLED ☐ (SKIP TO 634)
633	How long after the fever started did (NAME) first take LA?	SAME DAY . . . . 0<br>NEXT DAY . . . . 1<br>TWO DAYS AFTER<br>FEVER . . . . 2<br>THREE OR MORE DAYS<br>AFTER FEVER . . . . 3<br>DON'T KNOW 8	SAME DAY . . . . 0<br>NEXT DAY . . . . 1<br>TWO DAYS AFTER<br>FEVER . . . . 2<br>THREE OR MORE DAYS<br>AFTER FEVER . . . . 3<br>DON'T KNOW 8
634	CHECK 630:<br>ASAQ (COMBINED AMODIAQUINE AND ARTESUNATE) ('B') GIVEN	CODE 'B' CIRCLED ☐ ↓<br>CODE 'B' NOT CIRCLED ☐ (SKIP TO 636)	CODE 'B' CIRCLED ☐ ↓<br>CODE 'B' NOT CIRCLED ☐ (SKIP TO 636)
635	How long after the fever started did (NAME) first take ASAQ?	SAME DAY . . . . 0<br>NEXT DAY . . . . 1<br>TWO DAYS AFTER<br>FEVER . . . . 2<br>THREE OR MORE DAYS<br>AFTER FEVER . . . . 3<br>DON'T KNOW 8	SAME DAY . . . . 0<br>NEXT DAY . . . . 1<br>TWO DAYS AFTER<br>FEVER . . . . 2<br>THREE OR MORE DAYS<br>AFTER FEVER . . . . 3<br>DON'T KNOW 8
636	CHECK 630:<br>SP/FANSIDAR/NOVIDAR SP ('C') GIVEN	CODE 'C' CIRCLED ☐ ↓<br>CODE 'C' NOT CIRCLED ☐ (SKIP TO 640)	CODE 'C' CIRCLED ☐ ↓<br>CODE 'C' NOT CIRCLED ☐ (SKIP TO 640)
637	How long after the fever started did (NAME) first take SP/Fansidar/Novidar SP?	SAME DAY . . . . 0<br>NEXT DAY . . . . 1<br>TWO DAYS AFTER<br>FEVER . . . . 2<br>THREE OR MORE DAYS<br>AFTER FEVER . . . . 3<br>DON'T KNOW 8	SAME DAY . . . . 0<br>NEXT DAY . . . . 1<br>TWO DAYS AFTER<br>FEVER . . . . 2<br>THREE OR MORE DAYS<br>AFTER FEVER . . . . 3<br>DON'T KNOW 8

SECTION 6. CHILD HEALTH AND NUTRITION

NO.	QUESTIONS AND FILTERS	LAST BIRTH<br>NAME ______	NEXT-TO-LAST BIRTH<br>NAME ______
640	CHECK 630:<br>QUININE ('D' OR 'E') GIVEN	CODE 'D' OR 'E' CIRCLED ☐<br>CODE 'D' OR 'E' NOT CIRCLED ☐ (SKIP TO 642)	CODE 'D' OR 'E' CIRCLED ☐<br>CODE 'D' OR 'E' NOT CIRCLED ☐ (SKIP TO 642)
641	How long after the fever started did (NAME) first take quinine?	SAME DAY . . . . 0<br>NEXT DAY . . . . 1<br>TWO DAYS AFTER FEVER . . . . 2<br>THREE OR MORE DAYS AFTER FEVER . . . . 3<br>DON'T KNOW 8	SAME DAY . . . . 0<br>NEXT DAY . . . . 1<br>TWO DAYS AFTER FEVER . . . . 2<br>THREE OR MORE DAYS AFTER FEVER . . . . 3<br>DON'T KNOW 8
642	CHECK 630:<br>ARTESUNATE ('F' OR 'G') GIVEN	CODE 'F' OR 'G' CIRCLED ☐<br>CODE 'F' OR 'G' NOT CIRCLED ☐ (SKIP TO 644)	CODE 'F' OR 'G' CIRCLED ☐<br>CODE 'F' OR 'G' NOT CIRCLED ☐ (SKIP TO 644)
643	How long after the fever started did (NAME) first take artesunate?	SAME DAY . . . . 0<br>NEXT DAY . . . . 1<br>TWO DAYS AFTER FEVER . . . . 2<br>THREE OR MORE DAYS AFTER FEVER . . . . 3<br>DON'T KNOW 8	SAME DAY . . . . 0<br>NEXT DAY . . . . 1<br>TWO DAYS AFTER FEVER . . . . 2<br>THREE OR MORE DAYS AFTER FEVER . . . . 3<br>DON'T KNOW 8
644	CHECK 630:<br>OTHER ANTIMALARIAL ('H') GIVEN	CODE 'H' CIRCLED ☐<br>CODE 'H' NOT CIRCLED ☐ (SKIP TO 646)	CODE 'H' CIRCLED ☐<br>CODE 'H' NOT CIRCLED ☐ (SKIP TO 646)
645	How long after the fever started did (NAME) first take (OTHER ANTIMALARIAL)?	SAME DAY . . . . 0<br>NEXT DAY . . . . 1<br>TWO DAYS AFTER FEVER . . . . 2<br>THREE OR MORE DAYS AFTER FEVER . . . . 3<br>DON'T KNOW 8	SAME DAY . . . . 0<br>NEXT DAY . . . . 1<br>TWO DAYS AFTER FEVER . . . . 2<br>THREE OR MORE DAYS AFTER FEVER . . . . 3<br>DON'T KNOW 8
646		GO BACK TO 604 IN NEXT COLUMN; OR, IF NO MORE BIRTHS, GO TO 647.	GO TO 604 IN NEXT-TO-LAST COLUMN OF NEW QUESTIONNAIRE; OR, IF NO MORE BIRTHS, GO TO 647.

NO.	QUESTIONS AND FILTERS	CODING CATEGORIES	SKIP
647	CHECK 615(a), ALL COLUMNS:<br>NO CHILD RECEIVED FLUID FROM ORS PACKET ☐ ↓	ANY CHILD RECEIVED FLUID FROM ORS PACKET ☐	→ 649
648	Have you ever heard of a special product called THANZI-ORS PACKET you can get for the treatment of diarrhea?	YES . . . . . . 1<br>NO 2	
649	CHECK 215 AND 218, ALL ROWS: NUMBER OF CHILDREN BORN IN 2013-2015 LIVING WITH THE RESPONDENT<br>ONE OR MORE ☐ ↓<br>__________<br>(NAME OF YOUNGEST CHILD LIVING WITH HER) ↓	NONE ☐	→ 701

NO.	QUESTIONS AND FILTERS	CODING CATEGORIES				SKIP
650	Now I would like to ask you about liquids or foods that (NAME FROM 649) had yesterday during the day or at night. I am interested in whether your child had the item I mention even if it was combined with other foods.<br>Did (NAME FROM 649) drink or eat:		YES	NO	DK	
	a) Plain water?	a)	1	2	8	
	b) Juice or juice drinks?	b)	1	2	8	
	c) Soft drinks?	c)	1	2	8	
	d) Clear broth?	d)	1	2	8	
	e) Milk such as tinned, powdered, or fresh animal milk?<br>IF YES: How many times did (NAME) drink milk?<br>IF 7 OR MORE TIMES, RECORD '7'.	e) . . . .<br>NUMBER OF TIMES DRANK [ ]	1	2	8	
	f) Infant formula (S26, Naan, Lactogene, Infantcare)?<br>IF YES: How many times did (NAME) drink infant formula?<br>IF 7 OR MORE TIMES, RECORD '7'.	f) . . . .<br>NUMBER OF TIMES DRANK [ ]	1	2	8	
	g) Any other liquids?	g)	1	2	8	
	h) Yogurt?<br>IF YES: How many times did (NAME) eat yogurt?<br>IF 7 OR MORE TIMES, RECORD '7'.	h) . . . .<br>NUMBER OF TIMES ATE [ ]	1	2	8	
	i) Any fortified cereals (Cerelac, Likuni Phala, Nestum, Purity, Sibusiso, Gluco Phala)?	i)	1	2	8	
	j) Bread, rice, noodles, porridge, maize meal (ngaiwa), maize flour (ufawoyera), millet, sorghum, or other foods made from grains?	j)	1	2	8	
	k) Pumpkin, carrots, squash, or sweet potatoes that are yellow or orange inside?	k)	1	2	8	
	l) Cocoyams, irish potatoes, white sweet potatoes, white yams, cassava, or any other foods made from roots or tubers?	l)	1	2	8	
	m) Any dark green, leafy vegetables such as amaranth, pumpkin leaves, chinese cabbage, greens, kale, cassava leaves, beans, cow peas or sweet potato leaves that are fresh?	m)	1	2	8	
	n) Ripe mangoes, papayas, or guava?	n)	1	2	8	
	o) Any other fruits or vegetables (e.g. bananas, apples, green beans, avocados, tomatoes, okra)?	o)	1	2	8	
	p) Liver, kidney, heart, or other organ meats?	o)	1	2	8	
	q) Any meat, such as beef, pork, lamb, goat, chicken, duck, rabbit or rodents (such as mice, moles, etc.)?	q)	1	2	8	
	r) Grubs, snails or insects?	r)	1	2	8	
	s) Eggs?	s)	1	2	8	
	t) Fresh or dried fish or shellfish, crabs or seafood?	t)	1	2	8	
	u) Any foods made from beans, pigeon peas, cow peas, lentils, nuts, soybeans or ground nut powder (nsinjiro)?	u)	1	2	8	
	v) Cheese or other food made from milk?	v)	1	2	8	
	w) Any oil, fats, or butter, or foods made with any of these?	w)	1	2	8	
	x) Any sugary foods such as chocolates, sweets, candies, sugar cane, honey, patries, cakes, or biscuits?	x)	1	2	8	
	y) Any other solid, semi-solid, or soft food?	y)	1	2	8	

NO.	QUESTIONS AND FILTERS	CODING CATEGORIES	SKIP
651	CHECK 650 (CATEGORIES 'h' THROUGH 'y'):<br>NOT A SINGLE 'YES' ☐ ↓ AT LEAST ONE 'YES' ☐		→ 653
652	Did (NAME FROM 649) eat any solid, semi-solid, or soft foods yesterday during the day or at night?<br><br>IF 'YES' PROBE: What kind of solid, semi-solid or soft foods did (NAME) eat?	YES . . . . 1<br>(GO BACK TO 650 TO RECORD FOOD EATEN YESTERDAY)<br>(THEN CONTINUE TO 653)<br><br>NO 2	 <br><br><br>→ 654
653	How many times did (NAME FROM 649) eat solid, semi-solid, or soft foods yesterday during the day or at night?<br><br>IF 7 OR MORE TIMES, RECORD '7'.	NUMBER OF TIMES . . . . . . ☐<br><br>DON'T KNOW 8	
654	The last time (NAME FROM 649) passed stools, what was done to dispose of the stools?	CHILD USED TOILET OR LATRINE . . . . 01<br>PUT/RINSED<br>INTO TOILET OR LATRINE . . . . 02<br>PUT/RINSED<br>INTO DRAIN OR DITCH . . . . 03<br>THROWN INTO GARBAGE . . . . 04<br>BURIED . . . . 05<br>LEFT IN THE OPEN . . . . 06<br><br>OTHER ______ 96<br>(SPECIFY)	

SECTION 7. MARRIAGE AND SEXUAL ACTIVITY

NO.	QUESTIONS AND FILTERS	CODING CATEGORIES	SKIP
701	Are you currently married or living together with a man as if married?	YES, CURRENTLY MARRIED . . . . 1<br>YES, LIVING WITH A MAN . . . . 2<br>NO, NOT IN UNION 3	1, 2 → 704
702	Have you ever been married or lived together with a man as if married?	YES, FORMERLY MARRIED . . . . 1<br>YES, LIVED WITH A MAN . . . . 2<br>NO 3	3 → 712
703	What is your marital status now: are you widowed, divorced, or separated?	WIDOWED . . . . 1<br>DIVORCED . . . . 2<br>SEPARATED 3	→ 709
704	Is your (husband/partner) living with you now or is he staying elsewhere?	LIVING WITH HER . . . . 1<br>STAYING ELSEWHERE 2	
705	RECORD THE HUSBAND'S/PARTNER'S NAME AND LINE NUMBER FROM THE HOUSEHOLD QUESTIONNAIRE. IF HE IS NOT LISTED IN THE HOUSEHOLD, RECORD '00'.	NAME ____________<br><br>LINE NO. . . . . [ ][ ]	
706	Does your (husband/partner) have other wives or does he live with other women as if married?	YES . . . . 1<br>NO . . . . 2<br>DON'T KNOW 8	2, 8 → 709
707	Including yourself, in total, how many wives or live-in partners does he have?	TOTAL NUMBER OF WIVES AND LIVE-IN PARTNERS . . . . [ ][ ]<br><br>DON'T KNOW 98	
708	Are you the first, second, … wife?	RANK . . . . [ ][ ]	
709	Have you been married or lived with a man only once or more than once?	ONLY ONCE . . . . 1<br>MORE THAN ONCE 2	
710	CHECK 709:<br>MARRIED/LIVED WITH A MAN ONLY ONCE [ ] ↓ / MARRIED/LIVED WITH A MAN MORE THAN ONCE [ ] ↓<br>a) In what month and year did you start living with your (husband/partner)?<br>b) Now I would like to ask about your first (husband/partner). In what month and year did you start living with him?	MONTH . . . . [ ][ ]<br>DON'T KNOW MONTH . . . . 98<br>YEAR . . . . [ ][ ][ ][ ]<br>DON'T KNOW YEAR 9998	→ 712
711	How old were you when you first started living with him?	AGE . . . . [ ][ ]	

SECTION 7. MARRIAGE AND SEXUAL ACTIVITY

NO.	QUESTIONS AND FILTERS	CODING CATEGORIES	SKIP
712	**CHECK FOR PRESENCE OF OTHERS. BEFORE CONTINUING, MAKE EVERY EFFORT TO ENSURE PRIVACY.**		
713	Now I would like to ask some questions about sexual activity in order to gain a better understanding of some important life issues. Let me assure you again that your answers are completely confidential and will not be told to anyone. If we should come to any question that you don't want to answer, just let me know and we will go to the next question. How old were you when you had sexual intercourse for the very first time?	NEVER HAD SEXUAL INTERCOURSE . . . . 00<br>AGE IN YEARS . . . . [ ][ ]	→731
714	I would like to ask you about your recent sexual activity. When was the last time you had sexual intercourse?<br><br>IF LESS THAN 12 MONTHS, ANSWER MUST BE RECORDED IN DAYS, WEEKS OR MONTHS. IF 12 MONTHS (ONE YEAR) OR MORE, ANSWER MUST BE RECORDED IN YEARS.	DAYS AGO . . . . 1 [ ][ ]<br>WEEKS AGO . . . . 2 [ ][ ]<br>MONTHS AGO . . . . 3 [ ][ ]<br>YEARS AGO . . . . 4 [ ][ ]	(1–3) →716<br>(4) →727

		LAST SEXUAL PARTNER	SECOND-TO-LAST SEXUAL PARTNER	THIRD-TO-LAST SEXUAL PARTNER
715	When was the last time you had sexual intercourse with this person?		DAYS AGO . . 1<br>WEEKS AGO . . 2<br>MONTHS AGO . . 3	DAYS AGO . . 1<br>WEEKS AGO . . 2<br>MONTHS AGO . . 3
716	The last time you had sexual intercourse with this person, was a male or female used?	YES . . . . 1<br>NO . . . . 2<br>(SKIP TO 718)	YES . . . . 1<br>NO . . . . 2<br>(SKIP TO 718)	YES . . . . 1<br>NO . . . . 2<br>(SKIP TO 718)
717	Was a condom used every time you had sexual intercourse with this person in the last 12 months?	YES . . . . 1<br>NO 2	YES . . . . 1<br>NO 2	YES . . . . 1<br>NO 2
718	What was your relationship to this person with whom you had sexual intercourse?<br><br>IF BOYFRIEND: Were you living together as if married?<br><br>IF YES, RECORD '2'.<br>IF NO, RECORD '3'.	HUSBAND . . . . 1<br>LIVE-IN PARTNER . . . . . 2<br>BOYFRIEND NOT LIVING WITH RESPONDENT . . . . . 3<br>CASUAL ACQUAINTANCE . . 4<br>CLIENT/SEX WORKER . . 5<br>OTHER ______ 6<br>(SPECIFY)	HUSBAND . . . . 1<br>LIVE-IN PARTNER . . . . . 2<br>BOYFRIEND NOT LIVING WITH RESPONDENT . . . . . 3<br>CASUAL ACQUAINTANCE . . 4<br>CLIENT/SEX WORKER . . 5<br>OTHER ______ 6<br>(SPECIFY)	HUSBAND . . . . 1<br>LIVE-IN PARTNER . . . . . 2<br>BOYFRIEND NOT LIVING WITH RESPONDENT . . . . . 3<br>CASUAL ACQUAINTANCE . . 4<br>CLIENT/SEX WORKER . . 5<br>OTHER ______ 6<br>(SPECIFY)
719	How long ago did you first have sexual intercourse with this person?	DAYS AGO . . 1<br>WEEKS AGO . . 2<br>MONTHS AGO . . 3<br>YEARS AGO . . 4	DAYS AGO . . 1<br>WEEKS AGO . . 2<br>MONTHS AGO . . 3<br>YEARS AGO . . 4	DAYS AGO . . 1<br>WEEKS AGO . . 2<br>MONTHS AGO . . 3<br>YEARS AGO . . 4
720	How many times during the last 12 months did you have sexual intercourse with this person?<br>IF NON-NUMERIC ANSWER, PROBE TO GET AN ESTIMATE. IF NUMBER OF TIMES IS 95 OR MORE, RECORD '95'.	NUMBER OF TIMES	NUMBER OF TIMES	NUMBER OF TIMES . . . . .
721	How old is this person?	AGE OF PARTNER<br><br>DON'T KNOW 98	AGE OF PARTNER<br><br>DON'T KNOW 98	AGE OF PARTNER<br><br>DON'T KNOW 98
722	Apart from this person, have you had sexual intercourse with any other person in the last 12 months?	YES . . . . 1<br>(GO BACK TO 715 IN NEXT COLUMN)<br>NO . . . . 2<br>(SKIP TO 724)	YES . . . . 1<br>(GO BACK TO 715 IN NEXT COLUMN)<br>NO . . . . 2<br>(SKIP TO 724)	
723	In total, with how many different people have you had sexual intercourse in the last 12 months?<br><br>IF NON-NUMERIC ANSWER, PROBE TO GET AN ESTIMATE. IF NUMBER OF PARTNERS IS 95 OR MORE, RECORD '95'.			NUMBER OF PARTNERS LAST 12 MONTHS . .<br><br>DON'T KNOW 98

NO.	QUESTIONS AND FILTERS	CODING CATEGORIES	SKIP
724	CHECK 106:<br>AGE 15-24 ☐ ↓	AGE 25-49 ☐	→ 727
725	CHECK 701:<br>NOT IN A UNION ☐ ↓	CURRENTLY MARRIED/ LIVING WITH A MAN ☐	→ 727
726	In the past 12 months have you had sex or been sexually involved with anyone because he gave you or told you he would give you gifts, cash, or anything else?	YES . . . . . 1<br>NO 2	
727	In total, with how many different people have you had sexual intercourse in your lifetime?<br><br>IF NON-NUMERIC ANSWER, PROBE TO GET AN ESTIMATE. IF NUMBER OF PARTNERS IS 95 OR MORE, RECORD '95'.	NUMBER OF PARTNERS IN LIFETIME . . . . . ☐☐<br><br>DON'T KNOW 98	
728	CHECK 716, MOST RECENT PARTNER (FIRST COLUMN):<br>YES, CONDOM USED ☐ ↓	NO, CONDOM NOT USED ☐<br>NOT ASKED ☐	→ 731<br>→ 731
729	You told me that a condom was used the last time you had sex. What is the brand name of the condom used at that time?<br><br>IF BRAND NOT KNOWN, ASK TO SEE THE PACKAGE.	CHISHANGO . . . . . 01<br>MANYUCHI . . . . . 02<br>SILVERTOUCH . . . . . 03<br>CARE(FEMALE CONDOMS) . . . . . 04<br>PUBLIC SECTOR CONDOMS . . . . . 05<br><br>OTHER ______ 96<br>(SPECIFY)<br>DON'T KNOW 98	

NO.	QUESTIONS AND FILTERS	CODING CATEGORIES	SKIP
730	From where did you obtain the condom the last time?<br><br>PROBE TO IDENTIFY TYPE OF SOURCE.<br><br>IF UNABLE TO DETERMINE IF PUBLIC OR PRIVATE SECTOR, WRITE THE NAME OF THE PLACE.<br><br>______________________<br>(NAME OF PLACE)	**PUBLIC SECTOR**<br>GOVERNMENT HOSPITAL . . . . . 11<br>GOVERNMENT HEALTH CENTER . . . . . 12<br>GOVERNMENT HEALTH POST/ OUTREACH . . . . . 13<br>MOBILE CLINIC . . . . . 14<br>HSA . . . . . 15<br>CBDA/DOOR TO DOOR . . . . . 16<br>OTHER PUBLIC SECTOR<br>______________________ 17<br>(SPECIFY)<br><br>**CHAM/MISSION**<br>HOSPITAL . . . . . 21<br>HEALTH CENTER . . . . . 22<br>MOBILE CLINIC . . . . . 23<br>DOOR TO DOOR . . . . . 24<br><br>**PRIVATE MEDICAL SECTOR**<br>PRIVATE HOSPITAL/CLINIC . . . . . 31<br>PHARMACY . . . . . 32<br>PRIVATE DOCTOR . . . . . 33<br>MOBILE CLINIC . . . . . 34<br>CBDA/DOOR TC . . . . . 35<br>OTHER PRIVATE MEDICAL SECTOR<br>______________________ 36<br>(SPECIFY)<br><br>**BLM** . . . . . 41<br><br>**MACRO** . . . . . 51<br><br>**YOUTH DROP IN CENTRE** . . . . . 61<br><br>**OTHER SOURCE**<br>SHOP . . . . . 71<br>CHURCH . . . . . 72<br>FRIEND/RELATIVE . . . . . 73<br>CONDOMISED CAMPAIGNS . . . . . 74<br><br>OTHER ______________________ 96<br>(SPECIFY)<br>DON'T KNOW 98	
731	PRESENCE OF OTHERS DURING THIS SECTION.	YES NO<br>CHILDREN <10 . . . . . 1 2<br>MALE ADULTS . . . . . 1 2<br>FEMALE ADULTS 1 2	

SECTION 8. FERTILITY PREFERENCES

NO.	QUESTIONS AND FILTERS	CODING CATEGORIES	SKIP
801	CHECK 304: NEITHER STERILIZED ☐ ↓	HE OR SHE STERILIZED ☐	→ 813
802	CHECK 226: PREGNANT ☐ ↓	NOT PREGNANT OR UNSURE ☐	→ 804
803	Now I have some questions about the future. After the child you are expecting now, would you like to have another child, or would you prefer not to have any more children?	HAVE ANOTHER CHILD . . . . 1<br>NO MORE . . . . 2<br>UNDECIDED/DON'T KNOW 8	1 → 805<br>2, 8 → 812
804	Now I have some questions about the future. Would you like to have (a/another) child, or would you prefer not to have any (more) children?	HAVE (A/ANOTHER) CHILD . . . . 1<br>NO MORE/NONE . . . . 2<br>SAYS SHE CAN'T GET PREGNANT . . . . 3<br>UNDECIDED/DON'T KNOW 8	 → 807<br>→ 813<br>→ 811
805	CHECK 226:<br>NOT PREGNANT OR UNSURE ☐ ↓ a) How long would you like to wait from now before the birth of (a/another) child?<br>PREGNANT ☐ ↓ b) After the birth of the child you are expecting now, how long would you like to wait before the birth of another child?	MONTHS . . . . 1 [ ][ ]<br>YEARS . . . . 2 [ ][ ]<br>SOON/NOW . . . . 993<br>SAYS SHE CAN'T GET PREGNANT . . . . 994<br>AFTER MARRIAGE . . . . 995<br>OTHER ______________ 996<br>(SPECIFY)<br>DON'T KNOW 998	 <br>993 → 811<br>994 → 813<br>995, 996, 998 → 811
806	CHECK 226: NOT PREGNANT OR UNSURE ☐ ↓	PREGNANT ☐	→ 812
807	CHECK 303: USING A CONTRACEPTIVE METHOD?<br>NOT CURRENTLY USING ☐ ↓	CURRENTLY USING ☐	→ 813
808	CHECK 805:<br>'24' OR MORE MONTHS OR '02' OR MORE YEARS ☐ ↓ NOT ASKED ☐ ↓	'00-23' MONTHS OR '00-01' YEAR ☐	→ 812
809	CHECK 714:<br>DAYS, WEEKS OR MONTHS AGO ☐ ↓	YEARS AGO ☐<br>NOT ASKED ☐	→ 811<br>→ 811

NO.	QUESTIONS AND FILTERS	CODING CATEGORIES	SKIP
810	CHECK 804:<br>WANTS TO HAVE A/ANOTHER CHILD ☐ ↓ | WANTS NO MORE/ NONE ☐ ↓<br>a) You have said that you do not want (a/another) child soon. Can you tell me why you are not using a method to prevent pregnancy?<br>Any other reason?<br>b) You have said that you do not want any (more) children. Can you tell me why you are not using a method to prevent pregnancy?<br>Any other reason?<br>RECORD ALL REASONS MENTIONED.	NOT MARRIED . . . . A<br>**FERTILITY-RELATED REASONS**<br>NOT HAVING SEX . . . . B<br>INFREQUENT SEX . . . . C<br>MENOPAUSAL/HYSTERECTOMY . . . . . D<br>CAN'T GET PREGNANT . . . . E<br>NOT MENSTRUATED SINCE LAST BIRTH . . . . F<br>BREASTFEEDING . . . . G<br>UP TO GOD/FATALISTIC . . . . H<br>**OPPOSITION TO USE**<br>RESPONDENT OPPOSED . . . . I<br>HUSBAND/PARTNER OPPOSED . . . . . J<br>OTHERS OPPOSED . . . . K<br>RELIGIOUS PROHIBITION . . . . L<br>**LACK OF KNOWLEDGE**<br>KNOWS NO METHOD . . . . M<br>KNOWS NO SOURCE . . . . N<br>**METHOD-RELATED REASONS**<br>SIDE EFFECTS/HEALTH CONCERNS . . . . O<br>LACK OF ACCESS/TOO FAR . . . . . . P<br>COSTS TOO MUCH . . . . Q<br>PREFERRED METHOD NOT AVAILABLE . . . . R<br>NO METHOD AVAILABLE . . . . S<br>INCONVENIENT TO USE . . . . T<br>INTERFERES WITH BODY'S NORMAL PROCESSES . . . . U<br>OTHER ______________ X<br>(SPECIFY)<br>DON'T KNOW . . . . Z	
811	CHECK 303: USING A CONTRACEPTIVE METHOD?<br>NOT ASKED ☐ ↓ NO, NOT CURRENTLY USING ☐ ↓ YES, CURRENTLY USING ☐ →		813
812	Do you think you will use a contraceptive method to delay or avoid pregnancy at any time in the future?	YES . . . . 1<br>NO . . . . 2<br>DON'T KNOW 8	
813	CHECK 216:<br>HAS LIVING CHILDREN ☐ ↓ | NO LIVING CHILDREN ☐ ↓<br>a) If you could go back to the time you did not have any children and could choose exactly the number of children to have in your whole life, how many would that be?<br>b) If you could choose exactly the number of children to have in your whole life, how many would that be?<br>PROBE FOR A NUMERIC RESPONSE.	NONE . . . . 00<br>NUMBER . . . . ☐☐<br>OTHER ______________ 96<br>(SPECIFY)	00 → 815<br>96 → 815
814	How many of these children would you like to be boys, how many would you like to be girls and for how many would it not matter if it's a boy or a girl?	BOYS / GIRLS / EITHER<br>NUMBER . . ☐☐ ☐☐ ☐☐<br>OTHER ______________ 96<br>(SPECIFY)	

SECTION 8. FERTILITY PREFERENCES

NO.	QUESTIONS AND FILTERS	CODING CATEGORIES	SKIP
815	In the last few months have you:<br>a) Heard about family planning on the radio?<br>b) Seen anything about family planning on the television?<br>c) Read about family planning in a newspaper or magazine?<br>d) Received a voice or text message about family planning on a mobile phone?<br>e) Read about family planning on the internet/website?<br>f) Read about family planning on a poster?<br>g) Read about family planning on clothing (i.e. cap. chitenji, t-shirt)?<br>h) Heard about family planning in a drama?	YES NO<br>a) RADIO . . . . 1 2<br>b) TELEVISION . . . . 1 2<br>c) NEWSPAPER OR MAGAZINE . . . . 1 2<br>d) MOBILE PHONE . . . . 1 2<br>e) INTERNET/WEBSITE . . . . 1 2<br>f) POSTER/FLYERS/REFLETS . . . . 1 2<br>g) CLOTHING . . . . 1 2<br>h) DRAMA 1 2	
817	CHECK 701:<br>YES, CURRENTLY MARRIED ☐ ↓ YES, LIVING WITH A MAN ☐ ↓ NO, NOT IN A UNION ☐ → 901		901
818	CHECK 303: USING A CONTRACEPTIVE METHOD?<br>CURRENTLY USING ☐ ↓ NOT CURRENTLY USING ☐ → 820 NOT ASKED ☐ → 822		820<br>822
819	Would you say that using contraception is mainly your decision, mainly your (husband's/partner's) decision, or did you both decide together?	MAINLY RESPONDENT . . . . 1<br>MAINLY HUSBAND/PARTNER . . . . 2<br>JOINT DECISION . . . . 3<br><br>OTHER ______________________ 6<br>(SPECIFY)	821
820	Would you say that not using contraception is mainly your decision, mainly your (husband's/partner's) decision, or did you both decide together?	MAINLY RESPONDENT . . . . 1<br>MAINLY HUSBAND/PARTNER . . . . 2<br>JOINT DECISION . . . . 3<br><br>OTHER ______________________ 6<br>(SPECIFY)	
821	CHECK 304:<br>NEITHER ARE STERILIZED ☐ ↓ HE OR SHE ARE STERILIZED ☐ → 901		901
822	Does your (husband/partner) want the same number of children that you want, or does he want more or fewer than you want?	SAME NUMBER . . . . 1<br>MORE CHILDREN . . . . 2<br>FEWER CHILDREN . . . . 3<br>DON'T KNOW 8	

SECTION 9. HUSBAND'S BACKGROUND AND WOMAN'S WORK

NO.	QUESTIONS AND FILTERS	CODING CATEGORIES	SKIP
901	CHECK 701:<br>CURRENTLY MARRIED/ LIVING WITH A MAN ☐ ↓	NOT IN UNION ☐	→ 909
902	How old was your (husband/partner) on his last birthday?	AGE IN COMPLETED YEARS . . . . ☐☐	
903	Did your (husband/partner) ever attend school?	YES . . . . 1<br>NO 2	 → 906
904	What was the highest level of school he attended: primary, secondary, or higher?	PRIMARY . . . . 1<br>SECONDARY . . . . 2<br>HIGHER . . . . 3<br>DON'T KNOW 8	 <br><br>→ 906
905	What was the highest [FORM/YEAR] he completed at that level?<br>IF COMPLETED LESS THAN ONE YEAR AT THAT LEVEL, RECORD '00'.	[GRADE/FORM/YEAR] . . . . ☐☐<br>DON'T KNOW 98	
906	Has your (husband/partner) done any work in the last 7 days?	YES . . . . 1<br>NO . . . . 2<br>DON'T KNOW 8	→ 908
907	Has your (husband/partner) done any work in the last 12 months?	YES . . . . 1<br>NO . . . . 2<br>DON'T KNOW 8	 NO, DON'T KNOW → 909
908	What is your (husband's/partner's) occupation? That is, what kind of work does he mainly do?	____________________<br>____________________ ☐☐<br>____________________	
909	Aside from your own housework, have you done any work in the last seven days?	YES . . . . 1<br>NO 2	→ 913
910	As you know, some women take up jobs for which they are paid in cash or kind. Others sell things, have a small business or work on the family farm or in the family business. In the last seven days, have you done any of these things or any other work?	YES . . . . 1<br>NO 2	→ 913
911	Although you did not work in the last seven days, do you have any job or business from which you were absent for leave, illness, vacation, maternity leave, or any other such reason?	YES . . . . 1<br>NO 2	→ 913
912	Have you done any work in the last 12 months?	YES . . . . 1<br>NO 2	 → 917
913	What is your occupation? That is, what kind of work do you mainly do?	____________________<br>____________________ ☐☐<br>____________________	

NO.	QUESTIONS AND FILTERS	CODING CATEGORIES	SKIP
914	Do you do this work for a member of your family, for someone else, or are you self-employed?	FOR FAMILY MEMBER . . . . . 1<br>FOR SOMEONE ELSE . . . . . 2<br>SELF-EMPLOYED 3	
915	Do you usually work throughout the year, or do you work seasonally, or only once in a while?	THROUGHOUT THE YEAR . . . . . 1<br>SEASONALLY/PART OF THE YEAR . . . . . 2<br>ONCE IN A WHILE 3	
916	Are you paid in cash or kind for this work or are you not paid at all?	CASH ONLY . . . . . 1<br>CASH AND KIND . . . . . 2<br>IN KIND ONLY . . . . . 3<br>NOT PAID 4	
917	CHECK 701:<br>CURRENTLY MARRIED/LIVING WITH A MAN ☐ ↓	NOT IN UNION ☐	→ 925
918	CHECK 916:<br>CODE '1' OR '2' CIRCLED ☐ ↓	OTHER ☐	→ 921
919	Who usually decides how the money you earn will be used: you, your (husband/partner), or you and your (husband/partner) jointly?	RESPONDENT . . . . . 1<br>HUSBAND/PARTNER . . . . . 2<br>RESPONDENT AND<br>HUSBAND/PARTNER JOINTLY . . . . . 3<br><br>OTHER ______ 6<br>(SPECIFY)	
920	Would you say that the money that you earn is more than what your (husband/partner) earns, less than what he earns, or about the same?	MORE THAN HIM . . . . . 1<br>LESS THAN HIM . . . . . 2<br>ABOUT THE SAME . . . . . 3<br>HUSBAND/PARTNER HAS<br>NO EARNINGS . . . . . 4<br>DON'T KNOW 8	 <br><br><br>→ 922
921	Who usually decides how your (husband's/partner's) earnings will be used: you, your (husband/partner), or you and your (husband/partner) jointly?	RESPONDENT . . . . . 1<br>HUSBAND/PARTNER . . . . . 2<br>RESPONDENT AND<br>HUSBAND/PARTNER JOINTLY . . . . . 3<br>HUSBAND/PARTNER HAS<br>NO EARNINGS . . . . . 4<br><br>OTHER ______ 6<br>(SPECIFY)	
922	Who usually makes decisions about health care for yourself: you, your (husband/partner), you and your (husband/partner) jointly, or someone else?	RESPONDENT . . . . . 1<br>HUSBAND/PARTNER . . . . . 2<br>RESPONDENT AND<br>HUSBAND/PARTNER JOINTLY . . . . . 3<br>SOMEONE ELSE . . . . . 4<br>OTHER 6	
923	Who usually makes decisions about making major household purchases:you, your (husband/partner), you and your (husband/partner) jointly, or someone else?	RESPONDENT . . . . . 1<br>HUSBAND/PARTNER . . . . . 2<br>RESPONDENT AND<br>HUSBAND/PARTNER JOINTLY . . . . . 3<br>SOMEONE ELSE . . . . . 4<br>OTHER 6	

SECTION 9. HUSBAND'S BACKGROUND AND WOMAN'S WORK

NO.	QUESTIONS AND FILTERS	CODING CATEGORIES	SKIP
924	Who usually makes decisions about visits to your family or relatives:you, your (husband/partner), you and your (husband/partner) jointly, or someone else?	RESPONDENT . . . . . . 1<br>HUSBAND/PARTNER . . . . . . 2<br>RESPONDENT AND<br>HUSBAND/PARTNER JOINTLY . . . . . . 3<br>SOMEONE ELSE . . . . . . 4<br>OTHER 6	
925	Do you own this or any other house either alone or jointly with someone else?	ALONE ONLY . . . . . . 1<br>JOINTLY ONLY . . . . . . 2<br>BOTH ALONE AND JOINTLY . . . . . . 3<br>DOES NOT OWN 4	4 → 928
926	Do you have a title deed for any house you own?	YES . . . . . . 1<br>NO . . . . . . 2<br>DON'T KNOW 8	2, 8 → 928
927	Is your name on the title deed?	YES . . . . . . 1<br>NO . . . . . . 2<br>DON'T KNOW 8	
928	Do you own any agricultural or non-agricultural land either alone or jointly with someone else?	ALONE ONLY . . . . . . 1<br>JOINTLY ONLY . . . . . . 2<br>BOTH ALONE AND JOINTLY . . . . . . 3<br>DOES NOT OWN 4	4 → 931
929	Do you have a title deed for any land you own?	YES . . . . . . 1<br>NO . . . . . . 2<br>DON'T KNOW 8	2, 8 → 931
930	Is your name on the title deed?	YES . . . . . . 1<br>NO . . . . . . 2<br>DON'T KNOW 8	
931	PRESENCE OF OTHERS AT THIS POINT (PRESENT AND LISTENING, PRESENT BUT NOT LISTENING, OR NOT PRESENT)	PRES./LISTEN. — PRES./NOT LISTEN. — NOT PRES.<br>CHILDREN < 10 . . . . . 1 2 3<br>HUSBAND . . . . . 1 2 3<br>OTHER MALES . . . . . 1 2 3<br>OTHER FEMALES 1 2 3	
932	In your opinion, is a husband justified in hitting or beating his wife in the following situations:<br><br>a) If she goes out without telling him?<br>b) If she neglects the children?<br>c) If she argues with him?<br>d) If she refuses to have sex with him?<br>e) If she does not properly cook the food?	YES — NO — DK<br>a) GOES OUT . . . . . 1 2 8<br>b) NEGLECTS CHILDREN . . 1 2 8<br>c) ARGUES . . . . . 1 2 8<br>d) REFUSES SEX . . . . . 1 2 8<br>e) FOOD 1 2 8	

SECTION 10. HIV/AIDS

NO.	QUESTIONS AND FILTERS	CODING CATEGORIES	SKIP
1001	Now I would like to talk about something else. Have you ever heard of HIV or AIDS?	YES . . . 1<br>NO . . . 2	 2 → 1042
1002	HIV is the virus that can lead to AIDS. Can people reduce their chance of getting HIV by having just one uninfected sex partner who has no other sex partners?	YES . . . 1<br>NO . . . 2<br>DON'T KNOW . . . 8	
1003	Can people get HIV from mosquito bites?	YES . . . 1<br>NO . . . 2<br>DON'T KNOW . . . 8	
1004	Can people reduce their chance of getting HIV by using a condom every time they have sex?	YES . . . 1<br>NO . . . 2<br>DON'T KNOW . . . 8	
1004A	Can people reduce their chance of getting the AIDS virus by not having sexual intercourse at all?	YES . . . 1<br>NO . . . 2<br>DON'T KNOW . . . 8	
1005	Can people get HIV by sharing food with a person who has HIV?	YES . . . 1<br>NO . . . 2<br>DON'T KNOW . . . 8	
1006	Can people get HIV because of witchcraft or other supernatural means?	YES . . . 1<br>NO . . . 2<br>DON'T KNOW . . . 8	
1007	Is it possible for a healthy-looking person to have HIV?	YES . . . 1<br>NO . . . 2<br>DON'T KNOW . . . 8	
1008	Can HIV be transmitted from a mother to her baby:<br>a) During pregnancy?<br>b) During delivery?<br>c) By breastfeeding?	YES NO DK<br>a) DURING PREGNANCY . . 1 2 8<br>b) DURING DELIVERY . . . . . 1 2 8<br>c) BREASTFEEDING 1 2 8	
1009	CHECK 1008:<br>AT LEAST ONE 'YES' ☐ ↓ OTHER ☐ → 1011		1011
1010	Are there any special drugs that a doctor or a nurse can give to a woman infected with HIV to reduce the risk of transmission to the baby?	YES . . . 1<br>NO . . . 2<br>DON'T KNOW . . . 8	
1011	CHECK 208 AND 215:<br>LAST BIRTH IN 2013-2015 ☐ ↓ NO BIRTHS ☐ → 1027 LAST BIRTH IN 2012 OR EARLIER ☐ → 1027		1027<br>1027
1012	CHECK 408 FOR LAST BIRTH:<br>HAD ANTENATAL CARE ☐ ↓ NO ANTENATAL CARE ☐ → 1020		1020
1013	**CHECK FOR PRESENCE OF OTHERS. BEFORE CONTINUING, MAKE EVERY EFFORT TO ENSURE PRIVACY.**		
1014	During any of the antenatal visits for your last birth were you given any information about:<br>a) Babies getting HIV from their mother?<br>b) Things that you can do to prevent getting HIV?<br>c) Getting tested for HIV?	YES NO DK<br>a) HIV FROM MOTHER . . 1 2 8<br>b) THINGS TO DO . . . . 1 2 8<br>c) TESTED FOR HIV 1 2 8	

NO.	QUESTIONS AND FILTERS	CODING CATEGORIES	SKIP
1015	Were you offered a test for HIV as part of your antenatal care?	YES . . . 1<br>NO . . . 2	
1016	I don't want to know the results, but were you tested for HIV as part of your antenatal care?	YES . . . 1<br>NO . . . 2	2 → 1019A
1017	Where was the test done?<br><br>PROBE TO IDENTIFY THE TYPE OF SOURCE.<br><br>IF UNABLE TO DETERMINE IF PUBLIC OR PRIVATE SECTOR, WRITE THE NAME OF THE PLACE.<br><br>__________<br>(NAME OF PLACE)	**PUBLIC SECTOR**<br>GOVERNMENT HOSPITAL . . . 11<br>GOVERNMENT HEALTH CENTER . . . 12<br>GOVERNMENT HEALTH POST/<br>OUTREACH . . . 13<br>HSA . . . 14<br>DOOR TO DOOR . . . 15<br>OTHER PUBLIC SECTOR<br>__________ 16<br>(SPECIFY)<br><br>**CHAM/MISSION**<br>HOSPITAL . . . 21<br>HEALTH CENTER . . . 22<br>MOBILE CLINIC . . . 23<br>DOOR TO DOOR . . . 24<br><br>**PRIVATE MEDICAL SECTOR**<br>PRIVATE HOSPITAL/CLINIC/<br>PRIVATE DOCTOR . . . 31<br>LIGHT HOUSE . . . 32<br>DREAM CENTRE . . . 33<br>PHARMACY . . . 34<br>OTHER PRIVATE MEDICAL SECTOR<br>__________ 36<br>(SPECIFY)<br><br>**BLM** . . . 41<br><br>**MACRO** . . . 51<br><br>**OTHER SOURCE**<br>HOME . . . 61<br>WORKPLACE . . . 62<br>CORRECTIONAL FACILITY . . . 63<br><br>OTHER __________ 96<br>(SPECIFY)	
1018	I don't want to know the results, but did you get the results of the test?	YES . . . 1<br>NO . . . 2	2 → 1019A
1019	All women are supposed to receive counseling after being tested. After you were tested, did you receive counseling?	YES . . . 1<br>NO . . . 2<br>DON'T KNOW . . . 8	
1019A	During any of the antenatal visits for your last birth, was the baby's father offered a test for HIV by your health provider?	YES . . . 1<br>NO . . . 2	2 → 1020
1019B	I don't want to know the results, but was he tested for HIV at that time?	YES . . . 1<br>NO . . . 2<br>DON'T KNOW . . . 8	
1020	CHECK 430 FOR LAST BIRTH:<br>ANY CODE '21-51' CIRCLED ☐ ↓ OTHER ☐		OTHER → 1024
1021	Between the time you went for delivery but before the baby was born, were you offered an HIV test?	YES . . . 1<br>NO . . . 2	
1022	I don't want to know the results, but were you tested for HIV at that time?	YES . . . 1<br>NO . . . 2	2 → 1024
1023	I don't want to know the results, but did you get the results of the test?	YES . . . 1<br>NO . . . 2	1, 2 → 1025
1024	CHECK 1016:<br>YES ☐ ↓ NO OR NOT ASKED ☐		NO OR NOT ASKED → 1027
1025	Have you been tested for HIV since that time you were tested during your pregnancy?	YES . . . 1<br>NO . . . 2	1 → 1028
1026	How many months ago was your most recent HIV test?	MONTHS AGO . . . [ ][ ]<br><br>TWO OR MORE YEARS . . . 95	→ 1033

SECTION 10. HIV/AIDS

NO.	QUESTIONS AND FILTERS	CODING CATEGORIES	SKIP
1027	I don't want to know the results, but have you ever been tested for HIV?	YES . . . . 1<br>NO 2	 2 → 1031
1028	How many months ago was your most recent HIV test?	MONTHS AGO . . . . [ ][ ]<br>TWO OR MORE YEARS 95	
1029	I don't want to know the results, but did you get the results of the test?	YES . . . . 1<br>NO 2	
1029A	The last time you had the test, did you yourself ask for the test, was it offered to you and you accepted, or was it required by the health provider?	TEST REQUESTED BY THE RESPONDENT . . . . 1<br>TEST OFFERED BY THE HEALTH PROVIDER . . 2<br>TEST REQUIRED BY THE HEALTH PROVIDER . . 3	
1030	Where was the test done?<br><br>PROBE TO IDENTIFY THE TYPE OF SOURCE.<br><br>IF UNABLE TO DETERMINE IF PUBLIC OR PRIVATE SECTOR, WRITE THE NAME OF THE PLACE.<br><br>______________________<br>(NAME OF PLACE)	**PUBLIC SECTOR**<br>GOVERNMENT HOSPITAL . . . . 11<br>GOVERNMENT HEALTH CENTER . . . . 12<br>GOVERNMENT HEALTH POST/ OUTREACH . . . . 13<br>HSA . . . . 14<br>DOOR TO DOOR . . . . 15<br>OTHER PUBLIC SECTOR<br>______________________ 16<br>(SPECIFY)<br><br>**CHAM/MISSION**<br>HOSPITAL . . . . 21<br>HEALTH CENTER . . . . 22<br>MOBILE CLINIC . . . . 23<br>DOOR TO DOOR . . . . 24<br><br>**PRIVATE MEDICAL SECTOR**<br>PRIVATE HOSPITAL/CLINIC/ PRIVATE DOCTOR . . . . 31<br>PHARMACY . . . . 32<br>OTHER PRIVATE MEDICAL SECTOR<br>______________________ 36<br>(SPECIFY)<br><br>**BLM** . . . . 41<br><br>**MACRO** . . . . 51<br><br>**OTHER SOURCE**<br>HOME . . . . 61<br>WORKPLACE . . . . 62<br>CORRECTIONAL FACILITY . . . . 63<br><br>OTHER ______________________ 96<br>(SPECIFY)	→ 1033
1031	Do you know of a place where people can go to get an HIV test?	YES . . . . 1<br>NO 2	 2 → 1033
1032	Where is that?<br><br>Any other place?<br><br>PROBE TO IDENTIFY THE TYPE OF SOURCE.<br><br>IF UNABLE TO DETERMINE IF PUBLIC OR PRIVATE SECTOR, WRITE THE NAME OF THE PLACE.<br><br>______________________<br>(NAME OF PLACE)	**PUBLIC SECTOR**<br>GOVERNMENT HOSPITAL . . . . A<br>GOVERNMENT HEALTH CENTER . . . . B<br>GOVERNMENT HEALTH POST/ OUTREACH . . . . C<br>HSA . . . . D<br>DOOR TO DOOR . . . . E<br>OTHER PUBLIC SECTOR<br>______________________ F<br>(SPECIFY)<br><br>**CHAM/MISSION**<br>HOSPITAL . . . . G<br>HEALTH CENTER . . . . H<br>MOBILE CLINIC . . . . I<br>DOOR TO DOOR . . . . J<br><br>**PRIVATE MEDICAL SECTOR**<br>PRIVATE HOSPITAL/CLINIC/ PRIVATE DOCTOR . . . . K<br>PHARMACY . . . . L<br>OTHER PRIVATE MEDICAL SECTOR<br>______________________ M<br>(SPECIFY)<br><br>**BLM** . . . . N<br><br>**MACRO** . . . . O<br><br>**OTHER SOURCE**<br>HOME . . . . P<br>WORKPLACE . . . . Q<br>CORRECTIONAL FACILITY . . . . R<br><br>OTHER ______________________ X<br>(SPECIFY)	

SECTION 10. HIV/AIDS

NO.	QUESTIONS AND FILTERS	CODING CATEGORIES	SKIP
1033	Have you heard of test kits people can use to test themselves for HIV?	YES . . . . . 1<br>NO 2	 → 1035
1034	Have you ever tested yourself for HIV using a self-test kit?	YES . . . . . 1<br>NO 2	
1035	Would you buy fresh vegetables from a shopkeeper or vendor if you knew that this person had HIV?	YES . . . . . 1<br>NO . . . . . 2<br>DON'T KNOW/NOT SURE/DEPENDS 8	
1036	Do you think children living with HIV should be allowed to attend school with children who do not have HIV?	YES . . . . . 1<br>NO . . . . . 2<br>DON'T KNOW/NOT SURE/DEPENDS 8	
1037	Do you think people hesitate to take an HIV test because they are afraid of how other people will react if the test result is positive for HIV?	YES . . . . . 1<br>NO . . . . . 2<br>DON'T KNOW/NOT SURE/DEPENDS 8	
1038	Do people talk badly about people living with HIV, or who are thought to be living with HIV?	YES . . . . . 1<br>NO . . . . . 2<br>DON'T KNOW/NOT SURE/DEPENDS 8	
1039	Do people living with HIV, or thought to be living with HIV, lose the respect of other people?	YES . . . . . 1<br>NO . . . . . 2<br>DON'T KNOW/NOT SURE/DEPENDS 8	
1040	Do you agree or disagree with the following statement: I would be ashamed if someone in my family had HIV.	AGREE . . . . . 1<br>DISAGREE . . . . . 2<br>DON'T KNOW/NOT SURE/DEPENDS 8	
1041	Do you fear that you could get HIV if you come into contact with the saliva of a person living with HIV?	YES . . . . . 1<br>NO . . . . . 2<br>SAYS SHE HAS HIV . . . . . 3<br>DON'T KNOW/NOT SURE/DEPENDS 8	
1042	CHECK 1001:<br>HEARD ABOUT HIV OR AIDS ☐ ↓ / NOT HEARD ABOUT HIV OR AIDS ☐ ↓<br>a) Apart from HIV, have you heard about other infections that can be transmitted through sexual contact?<br>b) Have you heard about infections that can be transmitted through sexual contact?	YES . . . . . 1<br>NO 2	
1043	CHECK 713:<br>HAS HAD SEXUAL INTERCOURSE ☐ ↓ / NEVER HAD SEXUAL INTERCOURSE ☐ →		 → 1051
1044	CHECK 1042: HEARD ABOUT OTHER SEXUALLY TRANSMITTED INFECTIONS?<br>YES ☐ ↓ / NO ☐ →		 → 1046

NO.	QUESTIONS AND FILTERS	CODING CATEGORIES	SKIP
1045	Now I would like to ask you some questions about your health in the last 12 months. During the last 12 months, have you had a disease which you got through sexual contact?	YES . . . 1<br>NO . . . 2<br>DON'T KNOW . . . 8	
1046	Sometimes women experience a bad-smelling abnormal genital discharge. During the last 12 months, have you had a bad-smelling abnormal genital discharge?	YES . . . 1<br>NO . . . 2<br>DON'T KNOW . . . 8	
1047	Sometimes women have a genital sore or ulcer. During the last 12 months, have you had a genital sore or ulcer?	YES . . . 1<br>NO . . . 2<br>DON'T KNOW . . . 8	
1048	CHECK 1045, 1046, AND 1047:<br>HAS HAD AN INFECTION (ANY 'YES') ☐<br>HAS NOT HAD AN INFECTION OR DOES NOT KNOW ☐ → 1051		→ 1051
1049	The last time you had (PROBLEM FROM 1045/1046/1047), did you seek any kind of advice or treatment?	YES . . . 1<br>NO . . . 2	NO → 1051
1050	Where did you go?<br><br>Any other place?<br><br>PROBE TO IDENTIFY THE TYPE OF SOURCE.<br><br>IF UNABLE TO DETERMINE IF PUBLIC OR PRIVATE SECTOR, WRITE THE NAME OF THE PLACE.<br><br>____________<br>(NAME OF PLACE)	**PUBLIC SECTOR**<br>GOVERNMENT HOSPITAL . . . A<br>GOVERNMENT HEALTH CENTER . . . B<br>GOVERNMENT HEALTH POST/ OUTREACH . . . C<br>HSA . . . D<br>DOOR TO DOOR . . . E<br>OTHER PUBLIC SECTOR<br>____________ F<br>(SPECIFY)<br><br>**CHAM/MISSION**<br>HOSPITAL . . . G<br>HEALTH CENTER . . . H<br>MOBILE CLINIC . . . I<br>DOOR TO DOOR . . . J<br><br>**PRIVATE MEDICAL SECTOR**<br>PRIVATE HOSPITAL/CLINIC/ PRIVATE DOCTOR . . . K<br>PHARMACY . . . L<br>OTHER PRIVATE MEDICAL SECTOR<br>____________ M<br>(SPECIFY)<br><br>**BLM** . . . N<br><br>**MACRO** . . . O<br><br>**OTHER SOURCE**<br>SHOP . . . P<br><br>OTHER ____________ X<br>(SPECIFY)	
1051	If a wife knows her husband has a disease that she can get during sexual intercourse, is she justified in asking that they use a condom when they have sex?	YES . . . 1<br>NO . . . 2<br>DON'T KNOW . . . 8	
1052	Is a wife justified in refusing to have sex with her husband when she knows he has sex with other women?	YES . . . 1<br>NO . . . 2<br>DON'T KNOW . . . 8	

NO.	QUESTIONS AND FILTERS	CODING CATEGORIES	SKIP
1053	CHECK 701:<br>CURRENTLY MARRIED/ LIVING WITH A MAN ☐ ↓	NOT IN UNION ☐	→ 1101
1054	Can you say no to your (husband/partner) if you do not want to have sexual intercourse?	YES . . . . . 1<br>NO . . . . . 2<br>DEPENDS/NOT SURE 8	
1055	Could you ask your (husband/partner) to use a condom if you wanted him to?	YES . . . . . 1<br>NO . . . . . 2<br>DEPENDS/NOT SURE 8	

SECTION 11. OTHER HEALTH ISSUES

NO.	QUESTIONS AND FILTERS	CODING CATEGORIES	SKIP
1101	Now I would like to ask you some other questions relating to health matters. Have you had an injection for any reason in the last 12 months?<br><br>IF YES: How many injections have you had?<br><br>IF NUMBER OF INJECTIONS IS 90 OR MORE, OR DAILY FOR 3 MONTHS OR MORE, RECORD '90'. IF NON-NUMERIC ANSWER, PROBE TO GET AN ESTIMATE.	NUMBER OF INJECTIONS . . . . [ ][ ]<br><br>NONE 00	 <br>(NONE) → 1104
1102	Among these injections, how many were administered by a doctor, a nurse, a pharmacist, a dentist, or any other health worker?<br><br>IF NUMBER OF INJECTIONS IS 90 OR MORE, OR DAILY FOR 3 MONTHS OR MORE, RECORD '90'. IF NON-NUMERIC ANSWER, PROBE TO GET AN ESTIMATE.	NUMBER OF INJECTIONS . . . . [ ][ ]<br><br>NONE 00	 <br>(NONE) → 1104
1103	The last time you got an injection from a health worker, did he/she take the syringe and needle from a new, unopened package?	YES . . . . 1<br>NO . . . . 2<br>DON'T KNOW 8	
1104	Do you currently smoke cigarettes every day, some days, or not at all?	EVERY DAY . . . . 1<br>SOME DAYS . . . . 2<br>NOT AT ALL 3	(SOME DAYS, NOT AT ALL) → 1106
1105	On average, how many cigarettes do you currently smoke each day?	NUMBER OF CIGARETTES . . . . [ ][ ]	
1106	Do you currently smoke or use any other type of tobacco every day, some days, or not at all?	EVERY DAY . . . . 1<br>SOME DAYS . . . . 2<br>NOT AT ALL 3	(NOT AT ALL) → 1108
1107	What other type of tobacco do you currently smoke or use?<br><br>RECORD ALL MENTIONED.	PIPES FULL OF TOBACCO . . . . A<br>CIGARS, CHEROOTS, OR CIGARILLOS . . . . B<br>WATER PIPE . . . . C<br>SNUFF BY MOUTH . . . . D<br>SNUFF BY NOSE . . . . E<br>CHEWING TOBACCO . . . . F<br><br>OTHER ______________ X<br>(SPECIFY)	

NO.	QUESTIONS AND FILTERS	CODING CATEGORIES	BIG PROBLEM	NOT A BIG PROBLEM	SKIP
1108	Many different factors can prevent women from getting medical advice or treatment for themselves. When you are sick and want to get medical advice or treatment, is each of the following a big problem or not a big problem:				
	a) Getting permission to go to the doctor?	a) PERMISSION TO GO	1	2	
	b) Getting money needed for advice or treatment?	b) GETTING MONEY	1	2	
	c) The distance to the health facility?	c) DISTANCE	1	2	
	d) Not wanting to go alone?	d) GO ALONE	1	2	
	e) Concern that there may not be a female health provider?	e) NO FEMALE PROVIDER . .	1	2	
	f) Concern that there may not be any health provider?	f) NO PROVIDER	1	2	
	g) Concern that there may be no drugs available?	g) NO DRUGS	1	2	

SECTION 11. OTHER HEALTH ISSUES

NO.	QUESTIONS AND FILTERS	CODING CATEGORIES	SKIP
1109	Are you covered by any health insurance?	YES . . . 1<br>NO . . . 2	 → 1111
1110	What type of health insurance are you covered by?<br><br>RECORD ALL MENTIONED.	HEALTH INSURANCE THROUGH<br>EMPLOYER . . . A<br>PRIVATELY PURCHASED<br>COMMERCIAL HEALTH INSURANCE . . . B<br><br>OTHER ______ X<br>(SPECIFY)	
1111	Sometimes a woman can have a problem of constant leakage of urine or stool from her vagina during the day and night. This problem usually occurs after a difficult childbirth, but may also occur after a sexual assault or after pelvic surgery.<br><br>Have you ever experienced a constant leakage of urine or stool from your vagina during the day and night?	YES . . . 1<br>NO . . . 2	→ 1114
1112	Have you ever heard of this problem?	YES . . . 1<br>NO . . . 2	
1113	Do you know any woman who currently has or who has ever experienced this problem?	YES . . . 1<br>NO . . . 2	→ 1123
1114	Did this problem start after you delivered a baby or had a stillbirth?	AFTER DELIVERED BABY . . . 1<br>AFTER HAD STILLBIRTH . . . 2<br>NEITHER . . . 3	 <br>→ 1116
1115	Did this problem start after a normal labor and delivery, or after a very difficult labor and delivery, or after a very difficult labor and pelvic surgery ?	NORMAL LABOR/DELIVERY . . . 1<br>VERY DIFFICULT LABOR/DELIVERY . . . 2<br>PELVIC SURGERY . . . 3	→ 1117
1116	What do you think caused this problem?	SEXUAL ASSAULT . . . 1<br>PELVIC SURGERY . . . 2<br><br>OTHER ______ 6<br>(SPECIFY)<br>DON'T KNOW . . . 8	 <br><br><br><br>→ 1118
1117	How many days after (CAUSE OF PROBLEM FROM 1114 OR 1116) did the leakage start?<br><br>ENTER '90' IF 90 DAYS OR MORE.	NUMBER OF DAYS AFTER<br>DELIVERY/OTHER EVENT . . . [ ][ ]	
1118	Have you sought treatment for this condition?	YES . . . 1<br>NO . . . 2	→ 1120
1119	Why have you not sought treatment?<br><br>PROBE AND RECORD ALL MENTIONED.	DO NOT KNOW CAN BE FIXED . . . A<br>DO NOT KNOW WHERE TO GO . . . B<br>TOO EXPENSIVE . . . C<br>TOO FAR . . . D<br>POOR QUALITY OF CARE . . . E<br>COULD NOT GET PERMISSION . . . F<br>EMBARRASSMENT/STIGMA . . . G<br>PROBLEM DISAPPEARED . . . H<br><br>OTHER ______ X<br>(SPECIFY)	→ 1123

NO.	QUESTIONS AND FILTERS	CODING CATEGORIES	SKIP
1120	From whom did you last seek treatment?	**HEALTH PROFESSIONAL**<br>DOCTOR/CLINICAL OFFICER . . . . 1<br>NURSE/MIDWIFE . . . . 2<br>PATIENT ATTENDANT . . . . 4<br>**OTHER PERSON**<br>TRADITIONAL PRACTITIONEI . . . . 5<br><br>OTHER ______________________ 6<br>(SPECIFY)	
1121	Did you have an operation to fix the problem?	YES . . . . 1<br>NO 2	
1122	Did the treatment stop the leakage completely?<br><br>IF NO: Did the treatment reduce the leakage?	YES, STOPPED COMPLETELY . . . . 1<br>NOT STOPPED BUT REDUCED . . . . 2<br>NOT STOPPED AT ALL 3	
1123	Have you ever heard of an illness called tuberculosis or TB?	YES . . . . 1<br>NO 2	 → 1201
1124	How does tuberculosis spread from one person to another?<br><br>PROBE: Any other ways?<br>RECORD ALL MENTIONED.	THROUGH THE AIR WHEN<br>COUGHING OR SNEEZING . . . . A<br>THROUGH SHARING UTENSILS . . . . B<br>THROUGH TOUCHING A PERSON WITH TI . . . . . C<br>THROUGH FOOD . . . . D<br>THROUGH SEXUAL CONTACT . . . . E<br>THROUGH MOSQUITO BITES . . . . F<br><br>OTHER ______________________ X<br>(SPECIFY)<br>DON'T KNOW . . . . Z	
1125	Can tuberculosis be cured?	YES . . . . 1<br>NO . . . . 2<br>DON'T KNOW 8	
1126	If a member of your family got tuberculosis, would you want it to remain a secret or not?	YES, REMAIN A SECRET . . . . 1<br>NO . . . . 2<br>DON'T KNOW/NOT SURE/DEPENDS 8	

SECTION 12. MATERNAL MORTALITY MODULE

NO.	QUESTIONS AND FILTERS	CODING CATEGORIES	SKIP
1201	Now I would like to ask you some questions about your brothers and sisters, that is, all of the children born to your natural mother, including those who are living with you, those living elsewhere and those who have died. How many children did your mother give birth to, including you?	NUMBER OF BIRTHS TO NATURAL MOTHER . . . . ☐☐	
1202	CHECK 1201:<br>TWO OR MORE BIRTHS ☐ ↓ ONLY ONE BIRTH (RESPONDENT ONLY) ☐ →		1300
1203	How many births did your mother have before you were born?	NUMBER OF PRECEDING BIRTHS . . . . ☐☐	

NO.	QUESTION	(1)	(2)	(3)	(4)	(5)	(6)
1204	What was the name given to your (oldest/ next oldest) brother or sister?	________	________	________	________	________	________
1205	Is (NAME) male or female?	MALE . . . . 1<br>FEMALE . 2	MALE . . . . 1<br>FEMALE . 2	MALE . . . . 1<br>FEMALE . 2	MALE . . . . 1<br>FEMALE . 2	MALE . . . . 1<br>FEMALE . 2	MALE . . . . 1<br>FEMALE . 2
1206	Is (NAME) still alive?	YES . . . . . . 1<br>NO . . . . . . 2 GO TO 1208<br>DK . . . . . . 8 GO TO (2)	YES . . . . . . 1<br>NO . . . . . . 2 GO TO 1208<br>DK . . . . . . 8 GO TO (3)	YES . . . . . . 1<br>NO . . . . . . 2 GO TO 1208<br>DK . . . . . . 8 GO TO (4)	YES . . . . . . 1<br>NO . . . . . . 2 GO TO 1208<br>DK . . . . . . 8 GO TO (5)	YES . . . . . . 1<br>NO . . . . . . 2 GO TO 1208<br>DK . . . . . . 8 GO TO (6)	YES . . . . . . 1<br>NO . . . . . . 2 GO TO 1208<br>DK . . . . . . 8 GO TO (7)
1207	How old is (NAME)?	☐☐<br>GO TO (2)	☐☐<br>GO TO (3)	☐☐<br>GO TO (4)	☐☐<br>GO TO (5)	☐☐<br>GO TO (6)	☐☐<br>GO TO (7)
1208	How many years ago did (NAME) die?	☐☐	☐☐	☐☐	☐☐	☐☐	☐☐
1209	How old was (NAME) when (he/she) died?<br>IF DON'T KNOW, PROBE AND ASK ADDITIONAL QUESTIONS TO GET AN ESTIMATE.	☐☐<br>IF MALE OR DIED BEFORE 12 YEARS OF AGE GO TO 1214	☐☐<br>IF MALE OR DIED BEFORE 12 YEARS OF AGE GO TO 1214	☐☐<br>IF MALE OR DIED BEFORE 12 YEARS OF AGE GO TO 1214	☐☐<br>IF MALE OR DIED BEFORE 12 YEARS OF AGE GO TO 1214	☐☐<br>IF MALE OR DIED BEFORE 12 YEARS OF AGE GO TO 1214	☐☐<br>IF MALE OR DIED BEFORE 12 YEARS OF AGE GO TO 1214
1210	Was (NAME) pregnant when she died?	YES . . . . . . 1 GO TO 1214<br>NO 2	YES . . . . . . 1 GO TO 1214<br>NO 2	YES . . . . . . 1 GO TO 1214<br>NO 2	YES . . . . . . 1 GO TO 1214<br>NO 2	YES . . . . . . 1 GO TO 1214<br>NO 2	YES . . . . . . 1 GO TO 1214<br>NO 2
1211	Did (NAME) die during childbirth?	YES . . . . . . 1 GO TO 1214<br>NO 2	YES . . . . . . 1 GO TO 1214<br>NO 2	YES . . . . . . 1 GO TO 1214<br>NO 2	YES . . . . . . 1 GO TO 1214<br>NO 2	YES . . . . . . 1 GO TO 1214<br>NO 2	YES . . . . . . 1 GO TO 1214<br>NO 2
1212	Did (NAME) die within two months after the end of a pregnancy or childbirth?	YES . . . . . . 1<br>NO . . . . . . 2 GO TO 1214	YES . . . . . . 1<br>NO . . . . . . 2 GO TO 1214	YES . . . . . . 1<br>NO . . . . . . 2 GO TO 1214	YES . . . . . . 1<br>NO . . . . . . 2 GO TO 1214	YES . . . . . . 1<br>NO . . . . . . 2 GO TO 1214	YES . . . . . . 1<br>NO . . . . . . 2 GO TO 1214
1213	How many days after the end of the pregnancy did (NAME) die?	☐☐	☐☐	☐☐	☐☐	☐☐	☐☐
1214	Was (NAME)'s death due to an act of violence?	YES . . . . . . 1 GO TO (2)<br>NO 2	YES . . . . . . 1 GO TO (3)<br>NO 2	YES . . . . . . 1 GO TO (4)<br>NO 2	YES . . . . . . 1 GO TO (5)<br>NO 2	YES . . . . . . 1 GO TO (6)<br>NO 2	YES . . . . . . 1 GO TO (7)<br>NO 2
1215	Was (NAME)'s death due to an accident?	YES . . . . . . 1<br>NO . . . . . . 2<br>GO TO (2)	YES . . . . . . 1<br>NO . . . . . . 2<br>GO TO (3)	YES . . . . . . 1<br>NO . . . . . . 2<br>GO TO (4)	YES . . . . . . 1<br>NO . . . . . . 2<br>GO TO (5)	YES . . . . . . 1<br>NO . . . . . . 2<br>GO TO (6)	YES . . . . . . 1<br>NO . . . . . . 2<br>GO TO (7)

IF NO MORE BROTHERS OR SISTERS, GO TO 1300.

1204	What was the name given to your (oldest/ next oldest) brother or sister?	(7)<br>__________	(8)<br>__________	(9)<br>__________	(10)<br>__________	(11)<br>__________	(12)<br>__________
1205	Is (NAME) male or female?	MALE . . . . 1<br>FEMALE . 2	MALE . . . . 1<br>FEMALE . 2	MALE . . . . 1<br>FEMALE . 2	MALE . . . . 1<br>FEMALE . 2	MALE . . . . 1<br>FEMALE . 2	MALE . . . . 1<br>FEMALE . 2
1206	Is (NAME) still alive?	YES . . . . . . 1<br>NO . . . . . . 2<br>GO TO 1208<br>DK . . . . . . 8<br>GO TO (8)	YES . . . . . . 1<br>NO . . . . . . 2<br>GO TO 1208<br>DK . . . . . . 8<br>GO TO (9)	YES . . . . . . 1<br>NO . . . . . . 2<br>GO TO 1208<br>DK . . . . . . 8<br>GO TO (10)	YES . . . . . . 1<br>NO . . . . . . 2<br>GO TO 1208<br>DK . . . . . . 8<br>GO TO (11)	YES . . . . . . 1<br>NO . . . . . . 2<br>GO TO 1208<br>DK . . . . . . 8<br>GO TO (12)	YES . . . . . . 1<br>NO . . . . . . 2<br>GO TO 1208<br>DK . . . . . . 8<br>GO TO (13)
1207	How old is (NAME)?	GO TO (8)	GO TO (9)	GO TO (10)	GO TO (11)	GO TO (12)	GO TO (13)
1208	How many years ago did (NAME) die?						
1209	How old was (NAME) when (he/she) died?<br><br>IF DON'T KNOW, PROBE AND ASK ADDITIONAL QUESTIONS TO GET AN ESTIMATE.	IF MALE OR DIED BEFORE 12 YEARS OF AGE GO TO 1214	IF MALE OR DIED BEFORE 12 YEARS OF AGE GO TO 1214	IF MALE OR DIED BEFORE 12 YEARS OF AGE GO TO 1214	IF MALE OR DIED BEFORE 12 YEARS OF AGE GO TO 1214	IF MALE OR DIED BEFORE 12 YEARS OF AGE GO TO 1214	IF MALE OR DIED BEFORE 12 YEARS OF AGE GO TO 1214
1210	Was (NAME) pregnant when she died?	YES . . . . . . 1<br>GO TO 1214<br>NO 2	YES . . . . . . 1<br>GO TO 1214<br>NO 2	YES . . . . . . 1<br>GO TO 1214<br>NO 2	YES . . . . . . 1<br>GO TO 1214<br>NO 2	YES . . . . . . 1<br>GO TO 1214<br>NO 2	YES . . . . . . 1<br>GO TO 1214<br>NO 2
1211	Did (NAME) die during childbirth?	YES . . . . . . 1<br>GO TO 1214<br>NO 2	YES . . . . . . 1<br>GO TO 1214<br>NO 2	YES . . . . . . 1<br>GO TO 1214<br>NO 2	YES . . . . . . 1<br>GO TO 1214<br>NO 2	YES . . . . . . 1<br>GO TO 1214<br>NO 2	YES . . . . . . 1<br>GO TO 1214<br>NO 2
1212	Did (NAME) die within two months after the end of a pregnancy or childbirth?	YES . . . . . . 1<br>NO . . . . . . 2<br>GO TO 1214	YES . . . . . . 1<br>NO . . . . . . 2<br>GO TO 1214	YES . . . . . . 1<br>NO . . . . . . 2<br>GO TO 1214	YES . . . . . . 1<br>NO . . . . . . 2<br>GO TO 1214	YES . . . . . . 1<br>NO . . . . . . 2<br>GO TO 1214	YES . . . . . . 1<br>NO . . . . . . 2<br>GO TO 1214
1213	How many days after the end of the pregnancy did (NAME) die?						
1214	Was (NAME)'s death due to an act of violence?	YES . . . . . . 1<br>GO TO (8)<br>NO 2	YES . . . . . . 1<br>GO TO (9)<br>NO 2	YES . . . . . . 1<br>GO TO (10)<br>NO 2	YES . . . . . . 1<br>GO TO (11)<br>NO 2	YES . . . . . . 1<br>GO TO (12)<br>NO 2	YES . . . . . . 1<br>GO TO (13)<br>NO 2
1215	Was (NAME)'s death due to an accident?	YES . . . . . . 1<br>NO . . . . . . 2<br>GO TO (8)	YES . . . . . . 1<br>NO . . . . . . 2<br>GO TO (9)	YES . . . . . . 1<br>NO . . . . . . 2<br>GO TO (10)	YES . . . . . . 1<br>NO . . . . . . 2<br>GO TO (11)	YES . . . . . . 1<br>NO . . . . . . 2<br>GO TO (12)	YES . . . . . . 1<br>NO . . . . . . 2<br>GO TO (13)
IF NO MORE BROTHERS OR SISTERS, GO TO 1300.							

SECTION 13. DOMESTIC VIOLENCE MODULE

NO.	QUESTIONS AND FILTERS	CODING CATEGORIES	SKIP
1300	CHECK FRONT COVER<br>WOMAN SELECTED FOR THIS SECTION ☐ ↓	WOMAN NOT SELECTED ☐	→ 1333
1301	CHECK FOR PRESENCE OF OTHERS:<br>DO NOT CONTINUE UNTIL PRIVACY IS ENSURED.<br>PRIVACY OBTAINED . . . . 1 ↓	PRIVACY NOT POSSIBLE 2	→ 1332
1301A	READ TO THE RESPONDENT:<br>Now I would like to ask you questions about some other important aspects of a woman's life. You may find some of these questions very personal. However, your answers are crucial for helping to understand the condition of women in Malawi. Let me assure you that your answers are completely confidential and will not be told to anyone and no one else in your household will know that you were asked these questions. If I ask you any question you don't want to answer, just let me know and I will go on to the next question.		
1302	CHECK 701 AND 702:<br>CURRENTLY MARRIED/ LIVING WITH A MAN ☐ ↓<br>FORMERLY MARRIED/ LIVED WITH A MAN (READ IN PAST TENSE AND USE 'LAST' WITH 'HUSBAND/PARTNER') ☐ ↓	NEVER MARRIED/ NEVER LIVED WITH A MAN ☐	→ 1316

NO.	QUESTIONS AND FILTERS	CODING CATEGORIES	YES	NO	DK	SKIP
1303	First, I am going to ask you about some situations which happen to some women. Please tell me if these apply to your relationship with your (last) (husband/partner)?					
	a) He (is/was) jealous or angry if you (talk/talked) to other men?	JEALOUS	1	2	8	
	b) He frequently (accuses/accused) you of being unfaithful?	ACCUSES	1	2	8	
	c) He (does/did) not permit you to meet your female friends?	NOT MEET FRIENDS . .	1	2	8	
	d) He (tries/tried) to limit your contact with your family?	NO FAMILY	1	2	8	
	e) He (insists/insisted) on knowing where you (are/were) at all times?	WHERE YOU ARE	1	2	8	

1304 Now I need to ask some more questions about your relationship with your (last) (husband/partner).

A. Did your (last) (husband/partner) ever:

B. How often did this happen during the last 12 months: often, only sometimes, or not at all?

	A. EVER		B. OFTEN	SOME-TIMES	NOT IN LAST 12 MONTHS
a) say or do something to humiliate you in front of others?	YES	1 →	1	2	3
	NO	2 ↓			
b) threaten to hurt or harm you or someone you care about?	YES	1 →	1	2	3
	NO	2 ↓			
c) insult you or make you feel bad about yourself?	YES	1 →	1	2	3
	NO	2 ↓			

SECTION 13. DOMESTIC VIOLENCE MODULE

NO.	QUESTIONS AND FILTERS		CODING CATEGORIES			SKIP
1305	A. Did your (last) (husband/partner) ever do any of the following things to you:		B. How often did this happen during the last 12 months: often, only sometimes, or not at all?			
		EVER	OFTEN	SOME-TIMES	NOT IN LAST 12 MONTHS	
	a) push you, shake you, or throw something at you?	YES 1 → / NO 2 ↓	1	2	3	
	b) slap you?	YES 1 → / NO 2 ↓	1	2	3	
	c) twist your arm or pull your hair?	YES 1 → / NO 2 ↓	1	2	3	
	d) punch you with his fist or with something that could hurt you?	YES 1 → / NO 2 ↓	1	2	3	
	e) kick you, drag you, or beat you up?	YES 1 → / NO 2 ↓	1	2	3	
	f) try to choke you or burn you on purpose?	YES 1 → / NO 2 ↓	1	2	3	
	g) threaten or attack you with a knife, gun, or other weapon?	YES 1 → / NO 2 ↓	1	2	3	
	h) physically force you to have sexual intercourse with him when you did not want to?	YES 1 → / NO 2 ↓	1	2	3	
	i) physically force you to perform any other sexual acts you did not want to?	YES 1 → / NO 2 ↓	1	2	3	
	j) force you with threats or in any other way to perform sexual acts you did not want to?	YES 1 → / NO 2 ↓	1	2	3	

NO.	QUESTIONS AND FILTERS	CODING CATEGORIES	SKIP
1306	CHECK 1305A (a-j): AT LEAST ONE 'YES' ☐ ↓	NOT A SINGLE 'YES' ☐	→ 1309
1307	How long after you first (got married/started living together) with your (last) (husband/partner) did (this/any of these things) first happen?<br><br>IF LESS THAN ONE YEAR, RECORD '00'.	NUMBER OF YEARS . . . . . ☐☐<br><br>BEFORE MARRIAGE/BEFORE LIVING TOGETHER 95	
1308	Did the following ever happen as a result of what your (last) (husband/partner) did to you:		
	a) You had cuts, bruises, or aches?	YES . . . . 1<br>NO 2	
	b) You had eye injuries, sprains, dislocations, or burns?	YES . . . . 1<br>NO 2	
	c) You had deep wounds, broken bones, broken teeth, or any other serious injury?	YES . . . . 1<br>NO 2	
1309	Have you ever hit, slapped, kicked, or done anything else to physically hurt your (last) (husband/partner) at times when he was not already beating or physically hurting you?	YES . . . . 1<br>NO 2	 → 1311
1310	In the last 12 months, how often have you done this to your (last) (husband/partner): often, only sometimes, or not at all?	OFTEN . . . . 1<br>SOMETIMES . . . . 2<br>NOT AT ALL 3	

SECTION 13. DOMESTIC VIOLENCE MODULE

NO.	QUESTIONS AND FILTERS	CODING CATEGORIES	SKIP
1311	Does (did) your (last) (husband/partner) drink alcohol?	YES . . . 1<br>NO . . . 2	 2 → 1313
1312	How often does (did) he get drunk: often, only sometimes, or never?	OFTEN . . . 1<br>SOMETIMES . . . 2<br>NEVER . . . 3	
1313	Are (Were) you afraid of your (last) (husband/partner): most of the time, sometimes, or never?	MOST OF THE TIME AFRAID . . . 1<br>SOMETIMES AFRAID . . . 2<br>NEVER AFRAID . . . 3	
1314	CHECK 709:<br>MARRIED MORE THAN ONCE ☐ ↓	MARRIED ONLY ONCE ☐	→ 1316
1315	A. So far we have been talking about the behavior of your (current/last) (husband/partner). Now I want to ask you about the behavior of any previous (husband/partner).	B. How long ago did this last happen?	

	EVER	0 - 11 MONTHS AGO	12+ MONTHS AGO	DON'T REMEMBER
a) Did any previous (husband/partner) ever hit, slap, kick, or do anything else to hurt you physically?	YES 1 →<br>NO 2 ↓	1	2	3
b) Did any previous (husband/partner) physically force you to have intercourse or perform any other sexual acts against your will?	YES 1 →<br>NO 2 ↓	1	2	3

NO.	QUESTIONS AND FILTERS	CODING CATEGORIES	SKIP
1316	CHECK 701 AND 702:<br>EVER MARRIED/EVER LIVED WITH A MAN ☐ ↓<br>a) From the time you were 15 years old has anyone other than (your/any) (husband/partner) hit you, slapped you, kicked you, or done anything else to hurt you physically?<br>NEVER MARRIED/NEVER LIVED WITH A MAN ☐ ↓<br>b) From the time you were 15 years old has anyone hit you, slapped you, kicked you, or done anything else to hurt you physically?	YES . . . 1<br>NO . . . 2<br>REFUSED TO ANSWER/ NO ANSWER . . . 3	 2, 3 → 1319
1317	Who has hurt you in this way?<br><br>Anyone else?<br><br>RECORD ALL MENTIONED.	MOTHER . . . A<br>STEP-MOTHER . . . B<br>FATHER . . . C<br>STEP-FATHER . . . D<br>SISTER/BROTHER . . . E<br>DAUGHTER/SON . . . F<br>OTHER RELATIVE . . . G<br>CURRENT BOYFRIEND . . . H<br>FORMER BOYFRIEND . . . I<br>MOTHER-IN-LAW . . . J<br>FATHER-IN-LAW . . . K<br>OTHER IN-LAW . . . L<br>TEACHER . . . M<br>EMPLOYER/SOMEONE AT WORK . . . N<br>POLICE/SOLDIER . . . O<br><br>OTHER ________ X<br>(SPECIFY)	
1318	In the last 12 months, how often has (this person/have these persons) physically hurt you: often, only sometimes, or not at all?	OFTEN . . . 1<br>SOMETIMES . . . 2<br>NOT AT ALL . . . 3	

NO.	QUESTIONS AND FILTERS	CODING CATEGORIES	SKIP
1319	CHECK 201, 226, AND 230:<br>EVER BEEN PREGNANT ☐ ('YES' ON 201 OR 226 OR 230) ↓ NEVER BEEN PREGNANT ☐		→ 1322
1320	Has any one ever hit, slapped, kicked, or done anything else to hurt you physically while you were pregnant?	YES . . . . . 1<br>NO 2	 2 → 1322
1321	Who has done any of these things to physically hurt you while you were pregnant?<br><br>Anyone else?<br><br>RECORD ALL MENTIONED.	CURRENT HUSBAND/PARTNER . . . . . A<br>MOTHER . . . . . B<br>STEP-MOTHER . . . . . C<br>FATHER . . . . . D<br>STEP-FATHER . . . . . E<br>SISTER/BROTHER . . . . . F<br>DAUGHTER/SON . . . . . G<br>OTHER RELATIVE . . . . . H<br>FORMER HUSBAND/PARTNER . . . . . I<br>CURRENT BOYFRIEND . . . . . J<br>FORMER BOYFRIEND . . . . . K<br>MOTHER-IN-LAW . . . . . L<br>FATHER-IN-LAW . . . . . M<br>OTHER IN-LAW . . . . . N<br>TEACHER . . . . . O<br>EMPLOYER/SOMEONE AT WORK . . . . . P<br>POLICE/SOLDIER . . . . . Q<br><br>OTHER ______________ X<br>(SPECIFY)	
1322	CHECK 701 AND 702:<br>EVER MARRIED/EVER LIVED WITH A MAN ☐ ↓ NEVER MARRIED/NEVER LIVED WITH A MAN ☐		→ 1322B
1322A	Now I want to ask you about things that may have been done to you by someone other than (your/any) (husband/partner). At any time in your life, as a child or as an adult, has anyone ever forced you in any way to have sexual intercourse or perform any other sexual acts when you did not want to?	YES . . . . . 1<br>NO . . . . . 2<br>REFUSED TO ANSWER/<br>NO ANSWER 3	1 → 1323<br>2, 3 → 1324A
1322B	At any time in your life, as a child or as an adult, has anyone ever forced you in any way to have sexual intercourse or perform any other sexual acts when you did not want to?	YES . . . . . 1<br>NO . . . . . 2<br>REFUSED TO ANSWER/<br>NO ANSWER 3	 2, 3 → 1326
1323	Who was the person who was forcing you the very first time this happened?	CURRENT HUSBAND/PARTNER . . . . . 01<br>FORMER HUSBAND/PARTNER . . . . . 02<br>CURRENT/FORMER BOYFRIEND . . . . . 03<br>FATHER . . . . . 04<br>STEP-FATHER . . . . . 05<br>BROTHER . . . . . 06<br>STEP-BROTHER . . . . . 07<br>OTHER RELATIVE . . . . . 08<br>IN-LAW . . . . . 09<br>OWN FRIEND/ACQUAINTANCE . . . . . 10<br>FAMILY FRIEND . . . . . 11<br>TEACHER . . . . . 12<br>EMPLOYER/SOMEONE AT WORK . . . . . 13<br>POLICE/SOLDIER . . . . . 14<br>PRIEST/RELIGIOUS LEADER . . . . . 15<br>STRANGER . . . . . 16<br><br>OTHER ______________ 96<br>(SPECIFY)	

NO.	QUESTIONS AND FILTERS	CODING CATEGORIES	SKIP
1324	CHECK 701 AND 702:<br>EVER MARRIED/EVER LIVED WITH A MAN ☐ | NEVER MARRIED/NEVER LIVED WITH A MAN ☐<br>a) In the last 12 months, has anyone other than (your/any) (husband/partner) physically forced you to have sexual intercourse when you did not want to?<br>b) In the last 12 months has anyone physically forced you to have sexual intercourse when you did not want to?	YES . . . 1<br>NO . . . 2	→ 1325
1324A	CHECK 1305A (h-j) and 1315A(b)<br>AT LEAST ONE 'YES' ☐ ↓ NOT A SINGLE 'YES' ☐		→ 1326
1325	CHECK 701 AND 702:<br>EVER MARRIED/EVER LIVED WITH A MAN ☐ | NEVER MARRIED/NEVER LIVED WITH A MAN ☐<br>a) How old were you the first time you were forced to have sexual intercourse or perform any other sexual acts by anyone, including (your/any) husband/partner?<br>b) How old were you the first first time you were forced to have sexual intercourse or perform any other sexual acts?	AGE IN COMPLETED YEARS . . . [ ][ ]<br>DON'T KNOW . . . 98	
1326	CHECK 1305A (a-j), 1315A (a,b), 1316, 1320, 1322A, AND 1322B:<br>AT LEAST ONE 'YES' ☐ ↓ NOT A SINGLE 'YES' ☐		→ 1330
1327	Thinking about what you yourself have experienced among the different things we have been talking about, have you ever tried to seek help?	YES . . . 1<br>NO . . . 2	 → 1329
1328	From whom have you sought help?<br><br>Anyone else?<br><br>RECORD ALL MENTIONED.	OWN FAMILY . . . A<br>HUSBAND'S/PARTNER'S FAMILY . . . B<br>CURRENT/FORMER HUSBAND/PARTNER . . . C<br>CURRENT/FORMER BOYFRIEND . . . D<br>FRIEND . . . E<br>NEIGHBOR . . . F<br>RELIGIOUS LEADEI . . . G<br>DOCTOR/MEDICAL PERSONNEL . . . H<br>POLICE . . . I<br>LAWYER . . . J<br>SOCIAL SERVICE ORGANIZATIOI . . . K<br>DISTRICT SOCIAL WELFARE OFFICER L<br>TRADITIONAL AUTHORITY/CHIEI . . . M<br>EMPLOYER/SOMEONE AT WORI . . . N<br><br>OTHER ______ X<br>(SPECIFY)	→ 1330
1329	Have you ever told any one about this?	YES . . . 1<br>NO . . . 2	
1330	As far as you know, did your father ever beat your mother?	YES . . . 1<br>NO . . . 2<br>DON'T KNOW . . . 8	

SECTION 13. DOMESTIC VIOLENCE MODULE

NO.	QUESTIONS AND FILTERS	CODING CATEGORIES	SKIP
	THANK THE RESPONDENT FOR HER COOPERATION AND REASSURE HER ABOUT THE CONFIDENTIALITY OF HER ANSWERS. FILL OUT THE QUESTIONS BELOW WITH REFERENCE TO THE DOMESTIC VIOLENCE		
1331	DID YOU HAVE TO INTERRUPT THE INTERVIEW BECAUSE SOME ADULT WAS TRYING TO LISTEN, OR CAME INTO THE ROOM, OR INTERFERED IN ANY OTHER WAY?	YES, ONCE / YES, MORE THAN ONCE / NO<br>HUSBAND . . . . 1 2 3<br>OTHER MALE ADULT . . . . . 1 2 3<br>FEMALE ADULT 1 2 3	
1332	INTERVIEWER'S COMMENTS/EXPLANATION FOR NOT COMPLETING THE DOMESTIC VIOLENCE MODULE.		
1333	RECORD THE TIME.	HOURS . . . . [ ][ ]<br>MINUTES . . . . [ ][ ]	

INTERVIEWER'S OBSERVATIONS

TO BE FILLED IN AFTER COMPLETING INTERVIEW

COMMENTS ABOUT INTERVIEW:

COMMENTS ON SPECIFIC QUESTIONS:

ANY OTHER COMMENTS:

SUPERVISOR'S OBSERVATIONS

EDITOR'S OBSERVATIONS

INSTRUCTIONS:

ONLY ONE CODE SHOULD APPEAR IN ANY BOX.
COLUMN 1 REQUIRES A CODE IN EVERY MONTH.

CODES FOR EACH COLUMN:

COLUMN 1: <u>BIRTHS, PREGNANCIES, CONTRACEPTIVE USE</u>

B BIRTHS
P PREGNANCIES
T TERMINATIONS

0 NO METHOD

1 FEMALE STERILIZATION
2 MALE STERILIZATION
3 IUD
4 INJECTABLES

5 IMPLANTS
6 PILL
7 CONDOM
8 FEMALE CONDOM
9 EMERGENCY CONTRACEPTION
J STANDARD DAYS METHOD
K LACTATIONAL AMENORRHEA METHOD
L RHYTHM METHOD

M WITHDRAWAL
X OTHER MODERN METHOD
Y OTHER TRADITIONAL METHOD

COLUMN 2: <u>DISCONTINUATION OF CONTRACEPTIVE USE</u>

0 INFREQUENT SEX/HUSBAND AWAY
1 BECAME PREGNANT WHILE USING
2 WANTED TO BECOME PREGNANT
3 HUSBAND/PARTNER DISAPPROVED
4 WANTED MORE EFFECTIVE METHOD
5 SIDE EFFECTS/HEALTH CONCERNS

6 LACK OF ACCESS/TOO FAR
7 COSTS TOO MUCH

8 INCONVENIENT TO USE
F UP TO GOD/FATALISTIC
A DIFFICULT TO GET PREGNANT/MENOPAUSAL
D MARITAL DISSOLUTION/SEPARATION
X OTHER

____
(SPECIFY)

Z DON'T KNOW

Year				COL. 1	COL. 2	Year
2016	04	APR	01			2016
	03	MAR	02			
	02	FEB	03			
	01	JAN	04			
2015	12	DEC	05			2015
	11	NOV	06			
	10	OCT	07			
	09	SEP	08			
	08	AUG	09			
	07	JUL	10			
	06	JUN	11			
	05	MAY	12			
	04	APR	13			
	03	MAR	14			
	02	FEB	15			
	01	JAN	16			
2014	12	DEC	17			2014
	11	NOV	18			
	10	OCT	19			
	09	SEP	20			
	08	AUG	21			
	07	JUL	22			
	06	JUN	23			
	05	MAY	24			
	04	APR	25			
	03	MAR	26			
	02	FEB	27			
	01	JAN	28			
2013	12	DEC	29			2013
	11	NOV	30			
	10	OCT	31			
	09	SEP	32			
	08	AUG	33			
	07	JUL	34			
	06	JUN	35			
	05	MAY	36			
	04	APR	37			
	03	MAR	38			
	02	FEB	39			
	01	JAN	40			
2012	12	DEC	41			2012
	11	NOV	42			
	10	OCT	43			
	09	SEP	44			
	08	AUG	45			
	07	JUL	46			
	06	JUN	47			
	05	MAY	48			
	04	APR	49			
	03	MAR	50			
	02	FEB	51			
	01	JAN	52			
2011	12	DEC	53			2011
	11	NOV	54			
	10	OCT	55			
	09	SEP	56			
	08	AUG	57			
	07	JUL	58			
	06	JUN	59			
	05	MAY	60			
	04	APR	61			
	03	MAR	62			
	02	FEB	63			
	01	JAN	64			
2010	12	DEC	65			2010
	11	NOV	66			
	10	OCT	67			
	09	SEP	68			
	08	AUG	69			
	07	JUL	70			
	06	JUN	71			
	05	MAY	72			
	04	APR	73			
	03	MAR	74			
	02	FEB	75			
	01	JAN	76			

2015-2016 MALAWI DEMOGRAPHIC AND HEALTH SURVEY
MALAWI GOVERNEMNT - NATIONAL STATISTICAL OFFICE
MAN'S QUESTIONNAIRE

IDENTIFICATION

PLACE NAME ______________________

NAME OF HOUSEHOLD HEAD ______________________

CLUSTER NUMBER . . . . [ ][ ][ ][ ]

HOUSEHOLD NUMBER . . . . [ ][ ][ ][ ]

NAME AND LINE NUMBER OF MAN ______________________ [ ][ ]

INTERVIEWER VISITS

	1	2	3	FINAL VISIT	
DATE	______	______	______	DAY	[ ][ ]
				MONTH	[ ][ ]
				YEAR	[ ][ ][ ][ ]
INTERVIEWER'S NAME	______	______	______	INT. NO.	[ ][ ][ ][ ]
RESULT*	______	______	______	RESULT*	[ ]
NEXT VISIT: DATE	______	______		TOTAL NUMBER OF VISITS	[ ]
TIME	______	______			

*RESULT CODES: 1 COMPLETED 2 NOT AT HOME 3 POSTPONED 4 REFUSED 5 PARTLY COMPLETED 6 INCAPACITATED 7 OTHER ______ SPECIFY

LANGUAGE OF QUESTIONNAIRE** [0][1] LANGUAGE OF INTERVIEW** [ ][ ] NATIVE LANGUAGE OF RESPONDENT** [ ][ ] TRANSLATOR USED (YES = 1, NO = 2) [ ]

LANGUAGE OF QUESTIONNAIRE** ENGLISH

**LANGUAGE CODES:
01 ENGLISH
02 CHICHEWA
03 TUMBUKA
09 OTHER ______ (SPECIFY)

SUPERVISOR		OFFICE EDITOR	KEYED BY
______ NAME	[ ][ ][ ][ ] NUMBER	[ ][ ] NUMBER	[ ][ ] NUMBER

INTRODUCTION AND CONSENT

Hello. My name is ______________________________________. I am working with The National Statistical Office. We are conducting a survey about health and other topics all over Malawi. The information we collect will help the government to plan health services. Your household was selected for the survey. The questions usually take about 20 minutes. All of the answers you give will be confidential and will not be shared with anyone other than members of our survey team. You don't have to be in the survey, but we hope you will agree to answer the questions since your views are important. If I ask you any question you don't want to answer, just let me know and I will go on to the next question or you can stop the interview at any time.

In case you need more information about the survey, you may contact the person listed on the card that has already been given to your household.

Do you have any questions?
May I begin the interview now?

SIGNATURE OF INTERVIEWER ______________________________ DATE ________________

RESPONDENT AGREES TO BE INTERVIEWED . . 1 ↓

RESPONDENT DOES NOT AGREE TO BE INTERVIEWED . . 2 → END

SECTION 1. RESPONDENT'S BACKGROUND

NO.	QUESTIONS AND FILTERS	CODING CATEGORIES	SKIP
101	RECORD THE TIME.	HOURS . . . . [ ][ ]<br>MINUTES . . . . [ ][ ]	
102	How long have you been living continuously in (NAME OF CURRENT CITY, TOWN OR VILLAGE OF RESIDENCE)?<br><br>IF LESS THAN ONE YEAR, RECORD '00' YEARS.	YEARS . . . . [ ][ ]<br>ALWAYS . . . . 95<br>VISITOR 96	95, 96 → 105
103	Just before you moved here, did you live in a city, in a town, or in a rural area?	CITY . . . . 1<br>TOWN . . . . 2<br>RURAL AREA 3	
104	Before you moved here, which REGION did you live in?	NOTHERN . . . . 01<br>CENTRAL . . . . 02<br>SOUTHERN . . . . 03<br>OUTSIDE OF MALAWI 96	
105	In what month and year were you born?	MONTH . . . . [ ][ ]<br>DON'T KNOW MONTH . . . . 98<br>YEAR . . . . [ ][ ][ ][ ]<br>DON'T KNOW YEAR 9998	
106	How old were you at your last birthday?<br><br>COMPARE AND CORRECT 105 AND/OR 106 IF INCONSISTENT.	AGE IN COMPLETED YEARS . . . . [ ][ ]	
107	Have you ever attended school?	YES . . . . 1<br>NO 2	2 → 111
108	What is the highest level of school you attended: primary, secondary, or higher?	PRIMARY . . . . 1<br>SECONDARY . . . . 2<br>HIGHER 3	

SECTION 1. RESPONDENT'S BACKGROUND

NO.	QUESTIONS AND FILTERS	CODING CATEGORIES	SKIP
109	What is the highest [FORM/YEAR] you completed at that level?<br><br>IF COMPLETED LESS THAN ONE YEAR AT THAT LEVEL, RECORD '00'.	[GRADE/FORM/YEAR] . . . . [ ][ ]	
110	CHECK 108:<br>PRIMARY OR SECONDARY [ ] ↓ HIGHER [ ]		→ 113
111	Now I would like you to read this sentence to me.<br><br>SHOW CARD TO RESPONDENT.<br><br>IF RESPONDENT CANNOT READ WHOLE SENTENCE,<br>PROBE: Can you read any part of the sentence to me?	CANNOT READ AT ALL . . . . 1<br>ABLE TO READ ONLY PART OF THE SENTENCE . . . . 2<br>ABLE TO READ WHOLE SENTENCE . . . . 3<br>NO CARD WITH REQUIRED LANGUAGE ______________ 4<br>(SPECIFY LANGUAGE)<br>BLIND/VISUALLY IMPAIRED 5	
112	CHECK 111:<br>CODE '2', '3' OR '4' CIRCLED [ ] ↓ CODE '1' OR '5' CIRCLED [ ]		→ 114
113	Do you read a newspaper or magazine at least once a week, less than once a week or not at all?	AT LEAST ONCE A WEEK . . . . 1<br>LESS THAN ONCE A WEEK . . . . 2<br>NOT AT ALL 3	
114	Do you listen to the radio at least once a week, less than once a week or not at all?	AT LEAST ONCE A WEEK . . . . 1<br>LESS THAN ONCE A WEEK . . . . 2<br>NOT AT ALL 3	
115	Do you watch television at least once a week, less than once a week or not at all?	AT LEAST ONCE A WEEK . . . . 1<br>LESS THAN ONCE A WEEK . . . . 2<br>NOT AT ALL 3	
116	Do you own a mobile telephone?	YES . . . . 1<br>NO 2	 → 118
117	Do you use your mobile phone for any financial transactions?	YES . . . . 1<br>NO 2	
118	Do you have an account in a bank or other financial institution that you yourself use?	YES . . . . 1<br>NO 2	
119	Have you ever used the internet?<br><br>IF NECESSARY, PROBE FOR USE FROM ANY LOCATION, WITH ANY DEVICE.	YES . . . . 1<br>NO 2	 → 122
120	In the last 12 months, have you used the internet?<br><br>IF NECESSARY, PROBE FOR USE FROM ANY LOCATION, WITH ANY DEVICE.	YES . . . . 1<br>NO 2	 → 122
121	During the last one month, how often did you use the internet: almost every day, at least once a week, less than once a week, or not at all?	ALMOST EVERY DAY . . . . 1<br>AT LEAST ONCE A WEEK . . . . 2<br>LESS THAN ONCE A WEEK . . . . 3<br>NOT AT ALL 4	

SECTION 1. RESPONDENT'S BACKGROUND

NO.	QUESTIONS AND FILTERS	CODING CATEGORIES	SKIP
122	What is your religion?	CATHOLIC . . . . . 01<br>CCAP . . . . . 02<br>ANGLICAN . . . . . 03<br>SEVENTH DAY ADVENT./BAPTIST . . . . . 04<br>OTHER CHRISTIAN . . . . . 05<br>MUSLIM . . . . . 06<br>NO RELIGION . . . . . 07<br>OTHER ______ (SPECIFY) 96	
123	What is your tribe or ethnic group?	CHEWA . . . . . 01<br>TUMBUKA . . . . . 02<br>LOMWE . . . . . 03<br>TONGA . . . . . 04<br>YAO . . . . . 05<br>SENA . . . . . 06<br>NKHONDE . . . . . 07<br>NGONI . . . . . 08<br>OTHER ______ (SPECIFY) 96	
124	In the last 12 months, how many times have you been away from home for one or more nights?	NUMBER OF TIMES . . . . . [ ][ ]<br>NONE 00	 → 201
125	In the last 12 months, have you been away from home for more than one month at a time?	YES . . . . . 1<br>NO 2	

SECTION 2. REPRODUCTION

NO.	QUESTIONS AND FILTERS	CODING CATEGORIES	SKIP
201	Now I would like to ask about any children you have had during your life. I am interested in all of the children that are biologically yours, even if they are not legally yours or do not have your last name. Have you ever fathered any children with any woman?	YES . . . 1<br>NO . . . 2<br>DON'T KNOW . . . 8	2, 8 → 206
202	Do you have any sons or daughters that you have fathered who are now living with you?	YES . . . 1<br>NO . . . 2	2 → 204
203	a) How many sons live with you?<br>b) And how many daughters live with you?<br>IF NONE, RECORD '00'.	a) SONS AT HOME . . . [ ][ ]<br>b) DAUGHTERS AT HOME . . . [ ][ ]	
204	Do you have any sons or daughters that you have fathered who are alive but do not live with you?	YES . . . 1<br>NO . . . 2	2 → 206
205	a) How many sons are alive but do not live with you?<br>b) And how many daughters are alive but do not live with you?<br>IF NONE, RECORD '00'.	a) SONS ELSEWHERE . . . [ ][ ]<br>b) DAUGHTERS ELSEWHERE . . . [ ][ ]	
206	Have you ever fathered a son or a daughter who was born alive but later died?<br>IF NO, PROBE: Any baby who cried, who made any movement, sound, or effort to breathe, or who showed any other signs of life even if for a very short time?	YES . . . 1<br>NO . . . 2<br>DON'T KNOW . . . 8	2, 8 → 208
207	a) How many boys have died?<br>b) And how many girls have died?<br>IF NONE, RECORD '00'.	a) BOYS DEAD . . . [ ][ ]<br>b) GIRLS DEAD . . . [ ][ ]	
208	SUM ANSWERS TO 203, 205, AND 207, AND ENTER TOTAL. IF NONE, RECORD '00'.	TOTAL CHILDREN . . . [ ][ ]	
209	CHECK 208:<br>HAS HAD MORE THAN ONE CHILD [ ] ↓<br>HAS HAD ONLY ONE CHILD [ ] → 211<br>HAS NOT HAD ANY CHILDREN [ ] → 301		211<br>301
210	Did all of the children you have fathered have the same biological mother?	YES . . . 1<br>NO . . . 2	
211	CHECK 208:<br>HAS HAD MORE THAN ONE CHILD [ ] ↓ a) How old were you when your first child was born?<br>HAS HAD ONLY ONE CHILD [ ] ↓ b) How old were you when your child was born?	AGE IN YEARS . . . [ ][ ]	
212	CHECK 203 AND 205:<br>AT LEAST ONE LIVING CHILD [ ] ↓<br>NO LIVING CHILDREN [ ] → 301		301

SECTION 2. REPRODUCTION

NO.	QUESTIONS AND FILTERS	CODING CATEGORIES	SKIP
213	CHECK 203 AND 205:<br>MORE THAN ONE LIVING CHILD ☐ / ONLY ONE LIVING CHILD ☐<br>a) How old is your youngest child? / b) How old is your child?	AGE IN YEARS . . . . [ ][ ]	
214	CHECK 213:<br>(YOUNGEST) CHILD IS AGE 0-2 YEARS ☐ / (YOUNGEST) CHILD IS AGE 3 YEARS OR OLDER ☐		→ 301
215	CHECK 203 AND 205:<br>MORE THAN ONE LIVING CHILD ☐ / ONLY ONE LIVING CHILD ☐<br>a) What is the name of your youngest child? / b) What is the name of your child?	____________________<br>(NAME OF (YOUNGEST) CHILD)	
216	When (NAME)'s mother was pregnant with (NAME), did she have any antenatal check-ups?	YES . . . . 1<br>NO . . . . 2<br>DON'T KNOW 8	NO, DON'T KNOW → 218
217	Were you ever present during any of those antenatal check-ups?	PRESENT . . . . 1<br>NOT PRESENT 2	NOT PRESENT → 218
217A	Were you offered a test for HIV by the health provider during any of the antenatal check-ups?	YES . . . . 1<br>NO 2	NO → 218
217B	I don’t want to know the results, but were you tested for HIV at that time?	YES . . . . 1<br>NO 2	
218	Was (NAME) born in a hospital or health facility?	HOSPITAL/HEALTH FACILITY . . . . 1<br>OTHER 2	
219	When a child has diarrhea, how much should he or she be given to drink: more than usual, about the same as usual, less than usual, or nothing to drink at all?	MORE THAN USUAL . . . . 1<br>ABOUT THE SAME . . . . 2<br>LESS THAN USUAL . . . . 3<br>NOTHING TO DRINK . . . . 4<br>DON'T KNOW 8	

SECTION 3. CONTRACEPTION

301	Now I would like to talk about family planning - the various ways or methods that a couple can use to delay or avoid a pregnancy. Have you ever heard of (METHOD)?	
01	Female Sterilization.<br>PROBE: Women can have an operation to avoid having any more children.	YES . . . . . 1<br>NO 2
02	Male Sterilization.<br>PROBE: Men can have an operation to avoid having any more children.	YES . . . . . 1<br>NO 2
03	IUD.<br>PROBE: Women can have a loop or coil placed inside them by a doctor or a nurse which can prevent pregnancy for one or more years.	YES . . . . . 1<br>NO 2
04	Injectables.<br>PROBE: Women can have an injection by a health provider that stops them from becoming pregnant for one or more months.	YES . . . . . 1<br>NO 2
05	Implants.<br>PROBE: Women can have one or more small rods placed in their upper arm by a doctor or nurse which can prevent pregnancy for one or more years.	YES . . . . . 1<br>NO 2
06	Pill.<br>PROBE: Women can take a pill every day to avoid becoming pregnant.	YES . . . . . 1<br>NO 2
07	Condom.<br>PROBE: Men can put a rubber sheath on their penis before sexual intercourse.	YES . . . . . 1<br>NO 2
08	Female Condom.<br>PROBE: Women can place a sheath in their vagina before sexual intercourse.	YES . . . . . 1<br>NO 2
09	Emergency Contraception.<br>PROBE: As an emergency measure, within three days after they have unprotected sexual intercourse, women can take special pills to prevent pregnancy.	YES . . . . . 1<br>NO 2
10	Standard Days Method.<br>PROBE: A woman uses a string of colored beads to know the days she can get pregnant. On the days she can get pregnant, she uses a condom or does not have sexual intercourse.	YES . . . . . 1<br>NO 2
11	Lactational Amenorrhea Method (LAM).<br>PROBE: Up to six months after childbirth, before the menstrual period has returned, women use a method requiring frequent breastfeeding day and night.	YES . . . . . 1<br>NO 2
12	Rhythm Method.<br>PROBE: To avoid pregnancy, women do not have sexual intercourse on the days of the month they think they can get pregnant.	YES . . . . . 1<br>NO 2
13	Withdrawal.<br>PROBE: Men can be careful and pull out before climax.	YES . . . . . 1<br>NO 2
14	Have you heard of any other ways or methods that women or men can use to avoid pregnancy?	YES, MODERN METHOD<br>__________ 1<br>(SPECIFY)<br>YES, TRADITIONAL METHOD<br>__________ 2<br>(SPECIFY)<br>NO 3

SECTION 3. CONTRACEPTION

NO.	QUESTIONS AND FILTERS	CODING CATEGORIES	SKIP
302	In the last few months have you:<br>a) Heard about family planning on the radio?<br>b) Seen anything about family planning on the television?<br>c) Read about family planning in a newspaper or magazine?<br>d) Received a voice or text message about family planning on a mobile phone?<br>e) Read about family planning on the internet/website?<br>f) Read about family planning on a poster?<br>g) Read about family planning on clothing (i.e. cap. chitenji, t-shirt)?<br>h) Heard about family planning in a drama?	YES NO<br>a) RADIO . . . . . 1 2<br>b) TELEVISION . . . . . 1 2<br>c) NEWSPAPER OR MAGAZINE . . . . . 1 2<br>d) MOBILE PHONE . . . . . 1 2<br>e) INTERNET/WEBSITE . . . . . 1 2<br>f) POSTER . . . . . 1 2<br>g) CLOTHING . . . . . 1 2<br>h) DRAMA 1 2	
303	In the last few months, have you discussed family planning with a health worker or health professional?	YES . . . . . 1<br>NO 2	
304	Now I would like to ask you about a woman's risk of pregnancy. From one menstrual period to the next, are there certain days when a woman is more likely to become pregnant when she has sexual relations?	YES . . . . . 1<br>NO . . . . . 2<br>DON'T KNOW 8	NO, DON'T KNOW → 306
305	Is this time just before her period begins, during her period, right after her period has ended, or halfway between two periods?	JUST BEFORE HER PERIOD BEGIN‹ . . . . . 1<br>DURING HER PERIOD . . . . . 2<br>RIGHT AFTER HER PERIOD HAS ENDE . . . . . 3<br>HALFWAY BETWEEN TWO PERIOD . . . . . 4<br>OTHER ______ 6<br>(SPECIFY)<br>DON'T KNOW 8	
306	After the birth of a child, can a woman become pregnant before her menstrual period has returned?	YES . . . . . 1<br>NO . . . . . 2<br>DON'T KNOW 8	
307	I will now read you some statements about contraception. Please tell me if you agree or disagree with each one.<br>a) Contraception is a woman's concern and a man should not have to worry about it.<br>b) Women who use contraception may become promiscuous.	AGREE / DIS-AGREE / DK<br>a) CONTRACEPTION WOMAN'S CONCERN 1 2 8<br>b) WOMEN MAY BECOME PROMISCUOUS 1 2 8	

SECTION 4. MARRIAGE AND SEXUAL ACTIVITY

NO.	QUESTIONS AND FILTERS	CODING CATEGORIES	SKIP
401	Are you currently married or living together with a woman as if married?	YES, CURRENTLY MARRIED . . . . . 1<br>YES, LIVING WITH A WOMAN . . . . . 2<br>NO, NOT IN UNION 3	1, 2 → 404
402	Have you ever been married or lived together with a woman as if married?	YES, FORMERLY MARRIED . . . . . 1<br>YES, LIVED WITH A WOMAN . . . . . 2<br>NO 3	3 → 413
403	What is your marital status now: are you widowed, divorced, or separated?	WIDOWED . . . . . 1<br>DIVORCED . . . . . 2<br>SEPARATED 3	→ 410
404	Is your (wife/partner) living with you now or is she staying elsewhere?	LIVING WITH HIM . . . . . 1<br>STAYING ELSEWHERE 2	
405	Do you have other wives or do you live with other women as if married?	YES (MORE THAN ONE WIFE) . . . . . 1<br>NO (ONLY ONE WIFE) 2	2 → 407
406	Altogether, how many wives or live-in partners do you have?	TOTAL NUMBER OF WIVES AND LIVE-IN PARTNERS . . . . . [ ][ ]	
407	CHECK 405:<br>ONE WIFE/ PARTNER ☐ ↓ | MORE THAN ONE WIFE/ PARTNER ☐ ↓<br>a) Please tell me the name of (your wife/the woman you are living with as if married). | b) Please tell me the name of each of your wives or each woman you are living with as if married.<br>RECORD THE NAME AND THE LINE NUMBER FROM THE HOUSEHOLD QUESTIONNAIRE FOR EACH WIFE AND LIVE-IN PARTNER.<br>IF A WOMAN IS NOT LISTED IN THE HOUSEHOLD, RECORD '00'.	NAME | LINE NUMBER | 408 How old was (NAME) on her last birthday? AGE<br>_____ | [ ][ ] | [ ][ ]<br>_____ | [ ][ ] | [ ][ ]<br>_____ | [ ][ ] | [ ][ ]<br>_____ | [ ][ ] | [ ][ ]	
408	ASK 408 FOR EACH PERSON.		
409	CHECK 407:<br>ONE WIFE/ PARTNER ☐ ↓ MORE THAN ONE WIFE/ PARTNER ☐		MORE THAN ONE WIFE/PARTNER → 411
410	Have you been married or lived with a woman only once or more than once?	MORE THAN ONCE . . . . . 1<br>ONLY ONCE 2	
411	CHECK 405 AND 410:<br>BOTH ARE CODE '2' ☐ ↓ | OTHER ☐ ↓<br>a) In what month and year did you start living with your (wife/partner)? | b) Now I would like to ask about your first (wife/partner). In what month and year did you start living with her?	MONTH . . . . . [ ][ ]<br>DON'T KNOW MONTH . . . . . 98<br>YEAR . . . . . [ ][ ][ ][ ]<br>DON'T KNOW YEAR 9998	YEAR → 413
412	How old were you when you first started living with her?	AGE . . . . . [ ][ ]	

SECTION 4. MARRIAGE AND SEXUAL ACTIVITY

NO.	QUESTIONS AND FILTERS	CODING CATEGORIES	SKIP
413	**CHECK FOR PRESENCE OF OTHERS. BEFORE CONTINUING, MAKE EVERY EFFORT TO ENSURE PRIVACY.**		
414	I would like to ask some questions about sexual activity in order to gain a better understanding of some important life issues. Let me assure you again that your answers are completely confidential and will not be told to anyone. If we should come to any question that you don't want to answer, just let me know and we will go to the next question.<br><br>How old were you when you had sexual intercourse for the very first time?	NEVER HAD SEXUAL INTERCOURSE . . . . 00<br><br>AGE IN YEARS . . . . [ ][ ]	00 → 501
415	Now I would like to ask you about your recent sexual activity. When was the last time you had sexual intercourse?<br><br>IF LESS THAN 12 MONTHS, ANSWER MUST BE RECORDED IN DAYS, WEEKS OR MONTHS. IF 12 MONTHS (ONE YEAR) OR MORE, ANSWER MUST BE RECORDED IN YEARS.	DAYS AGO . . . . 1 [ ][ ]<br>WEEKS AGO . . . . 2 [ ][ ]<br>MONTHS AGO . . . . 3 [ ][ ]<br>YEARS AGO . . . . 4 [ ][ ]	1–3 → 417<br>4 → 427

		LAST SEXUAL PARTNER	SECOND-TO-LAST SEXUAL PARTNER	THIRD-TO-LAST SEXUAL PARTNER
416	When was the last time you had sexual intercourse with this person?		DAYS AGO . . 1<br>WEEKS AGO . . 2<br>MONTHS AGO . . 3	DAYS AGO . . 1<br>WEEKS AGO . . 2<br>MONTHS AGO . . 3
417	The last time you had sexual intercourse with this person, was a condom used?	YES . . . . 1<br>NO . . . . 2<br>(SKIP TO 419)	YES . . . . 1<br>NO . . . . 2<br>(SKIP TO 419)	YES . . . . 1<br>NO . . . . 2<br>(SKIP TO 419)
418	Was a male or female condom used every time you had sexual intercourse with this person in the last 12 months?	YES . . . . 1<br>NO 2	YES . . . . 1<br>NO 2	YES . . . . 1<br>NO 2
419	What was your relationship to this person with whom you had sexual intercourse?<br><br>IF GIRLFRIEND: Were you living together as if married?<br><br>IF YES, RECORD '2'.<br>IF NO, RECORD '3'.	WIFE . . . . 1<br>LIVE-IN PARTNER . . . . . 2<br>GIRLFRIEND NOT LIVING WITH RESPONDENT . . . . . 3<br>CASUAL ACQUAINTANCE . . 4<br>CLIENT/SEX WORKER . . 5<br>OTHER ________ 6<br>(SPECIFY)	WIFE . . . . 1<br>LIVE-IN PARTNER . . . . . 2<br>GIRLFRIEND NOT LIVING WITH RESPONDENT . . . . . 3<br>CASUAL ACQUAINTANCE . . 4<br>CLIENT/SEX WORKER . . 5<br>OTHER ________ 6<br>(SPECIFY)	WIFE . . . . 1<br>LIVE-IN PARTNER . . . . . 2<br>GIRLFRIEND NOT LIVING WITH RESPONDENT . . . . . 3<br>CASUAL ACQUAINTANCE . . 4<br>CLIENT/SEX WORKER . . 5<br>OTHER ________ 6<br>(SPECIFY)
420	How long ago did you first have sexual intercourse with this person?	DAYS AGO . . 1<br>WEEKS AGO . . 2<br>MONTHS AGO . . 3<br>YEARS AGO . . 4	DAYS AGO . . 1<br>WEEKS AGO . . 2<br>MONTHS AGO . . 3<br>YEARS AGO . . 4	DAYS AGO . . 1<br>WEEKS AGO . . 2<br>MONTHS AGO . . 3<br>YEARS AGO . . 4
421	How many times during the last 12 months did you have sexual intercourse with this person?<br>IF NON-NUMERIC ANSWER, PROBE TO GET AN ESTIMATE. IF NUMBER OF TIMES IS 95 OR MORE, RECORD '95'.	NUMBER OF TIMES	NUMBER OF TIMES	NUMBER OF TIMES . . . . .
422	How old is this person?	AGE OF PARTNER<br><br>DON'T KNOW 98	AGE OF PARTNER<br><br>DON'T KNOW 98	AGE OF PARTNER<br><br>DON'T KNOW 98
423	Apart from this person, have you had sexual intercourse with any other person in the last 12 months?	YES . . . . 1<br>(GO BACK TO 416 IN NEXT COLUMN)<br>NO . . . . 2<br>(SKIP TO 425)	YES . . . . 1<br>(GO BACK TO 416 IN NEXT COLUMN)<br>NO . . . . 2<br>(SKIP TO 425)	
424	In total, with how many different people have you had sexual intercourse in the last 12 months?<br>IF NON-NUMERIC ANSWER, PROBE TO GET AN ESTIMATE. IF NUMBER OF PARTNERS IS 95 OR MORE, RECORD '95'.			NUMBER OF PARTNERS LAST 12 MONTHS . .<br><br>DON'T KNOW 98

SECTION 4. MARRIAGE AND SEXUAL ACTIVITY

NO.	QUESTIONS AND FILTERS	CODING CATEGORIES	SKIP
425	CHECK 419 (ALL COLUMNS):<br>AT LEAST ONE PARTNER IS A SEX WORKER ☐ ↓	NO PARTNERS ARE SEX WORKERS ☐	→ 427
426	CHECK 419 AND 417 (ALL COLUMNS):<br>CONDOM USED WITH EVERY SEX WORKER ☐	OTHER ☐	→ 430<br>→ 431
427	In the last 12 months, did you pay anyone in exchange for having sexual intercourse?	YES . . . 1<br>NO . . . 2	1 → 429
428	Have you ever paid anyone in exchange for having sexual intercourse?	YES . . . 1<br>NO . . . 2	→ 431
429	The last time you paid someone in exchange for having sexual intercourse, was a condom used?	YES . . . 1<br>NO . . . 2	2 → 431
430	Was a condom used during sexual intercourse every time you paid someone in exchange for having sexual intercourse in the last 12 months?	YES . . . 1<br>NO . . . 2<br>DON'T KNOW . . . 8	
431	In the past 12 months have you given any gifts or other goods in order to have sex or to become sexually involved with anyone?	YES . . . 1<br>NO . . . 2	1 → 433
432	Have you ever given any gifts or other goods in order to have sex or to become sexually involved with anyone?	YES . . . 1<br>NO . . . 2	
433	In total, with how many different people have you had sexual intercourse in your lifetime?<br><br>IF NON-NUMERIC ANSWER, PROBE TO GET AN ESTIMATE. IF NUMBER OF PARTNERS IS 95 OR MORE, RECORD '95'.	NUMBER OF PARTNERS IN LIFETIME . . . ☐☐<br><br>DON'T KNOW . . . 98	
434	CHECK 417: MOST RECENT PARTNER (FIRST COLUMN)<br>CONDOM USED ☐ ↓	NOT ASKED ☐<br>NO CONDOM USED ☐	→ 438<br>→ 438
435	You told me that a condom was used the last time you had sex. What is the brand name of the condom used at that time?<br><br>IF BRAND NOT KNOWN, ASK TO SEE THE PACKAGE.	CHISHANGO . . . 01<br>MANYUCHI . . . 02<br>SILVERTOUCH . . . 03<br>CARE(FEMALE CONDOM) . . . 04<br>PUBLIC SECTOR CONDOMS . . . 05<br><br>OTHER ________ 96<br>(SPECIFY)<br>DON'T KNOW . . . 98	

NO.	QUESTIONS AND FILTERS	CODING CATEGORIES	SKIP
436	From where did you obtain the condom the last time?<br><br>PROBE TO IDENTIFY TYPE OF SOURCE.<br><br>IF UNABLE TO DETERMINE IF PUBLIC OR PRIVATE SECTOR, WRITE THE NAME OF THE PLACE.<br><br>__________<br>(NAME OF PLACE)	**PUBLIC SECTOR**<br>GOVERNMENT HOSPITAL . . . 11<br>GOVERNMENT HEALTH CENTER . . . 12<br>GOVERNMENT HEALTH POST/ OUTREACH . . . 13<br>MOBILE CLINIC . . . 14<br>HSA . . . 15<br>CBDA/DOOR TO DOOR . . . 16<br>OTHER PUBLIC SECTOR<br>__________ 17<br>(SPECIFY)<br><br>**CHAM/MISSION**<br>HOSPITAL . . . 21<br>HEALTH CENTER . . . 22<br>MOBILE CLINIC . . . 23<br>DOOR TO DOOR . . . 24<br><br>**PRIVATE MEDICAL SECTOR**<br>PRIVATE HOSPITAL/CLINIC . . . 31<br>PHARMACY . . . 32<br>PRIVATE DOCTOR . . . 33<br>MOBILE CLINIC . . . 34<br>CBDA/DOOR TC . . . 35<br>OTHER PRIVATE MEDICAL SECTOR<br>__________ 36<br>(SPECIFY)<br><br>**BLM** . . . 41<br><br>**MACRO** . . . 51<br><br>**YOUTH DROP IN CENTRE** . . . 61<br><br>**OTHER SOURCE**<br>SHOP . . . 71<br>CHURCH . . . 72<br>FRIEND/RELATIVE . . . 73<br>CONDOMISED CAMPAIGNS . . . 74<br><br>OTHER __________ 96<br>(SPECIFY)<br>DON'T KNOW . . . 98	
437	The last time you had sex did you or your partner use any method other than a condom to avoid or prevent a pregnancy?	YES . . . 1<br>NO . . . 2<br>DON'T KNOW . . . 8	1 → 439<br>2, 8 → 440
438	The last time you had sex did you or your partner use any method to avoid or prevent a pregnancy?	YES . . . 1<br>NO . . . 2<br>DON'T KNOW . . . 8	2, 8 → 440
439	What method did you or your partner use?<br><br>PROBE: Did you or your partner use any other method to prevent pregnancy?<br><br>RECORD ALL MENTIONED.	FEMALE STERILIZATION . . . A<br>MALE STERILIZATION . . . B<br>IUD . . . C<br>INJECTABLES . . . D<br>IMPLANTS . . . E<br>PILL . . . F<br>CONDOM . . . G<br>FEMALE CONDOM . . . H<br>EMERGENCY CONTRACEPTION . . . I<br>STANDARD DAYS METHOD . . . J<br>LACTATIONAL AMENORRHEA METHOD . . . K<br>RHYTHM METHOD . . . L<br>WITHDRAWAL . . . M<br>OTHER MODERN METHOD . . . X<br>OTHER TRADITIONAL METHOD . . . Y	→ 501
440	Do you know of a place where you can obtain a method of family planning?	YES . . . 1<br>NO . . . 2	

SECTION 5. FERTILITY PREFERENCES

NO.	QUESTIONS AND FILTERS	CODING CATEGORIES	SKIP
501	CHECK 401:<br>CURRENTLY MARRIED OR LIVING WITH A PARTNER ☐ ↓<br>NOT CURRENTLY MARRIED AND NOT LIVING WITH A PARTNER ☐ → 514		514
502	CHECK 439:<br>MAN NOT STERILIZED ☐ ↓<br>MAN STERILIZED ☐ → 514		514
503	CHECK 407:<br>ONE WIFE/ PARTNER ☐ ↓<br>MORE THAN ONE WIFE/ PARTNER ☐ → 509		509
504	Is your (wife/partner) currently pregnant?	YES . . . . . 1<br>NO . . . . . 2<br>DON'T KNOW 8	 2, 8 → 507
505	Now I have some questions about the future. After the child you and your (wife/partner) are expecting now, would you like to have another child, or would you prefer not to have any more children?	HAVE ANOTHER CHILD . . . . . 1<br>NO MORE . . . . . 2<br>UNDECIDED/DON'T KNOW 8	 2, 8 → 514
506	After the birth of the child you are expecting now, how long would you like to wait before the birth of another child?	MONTHS . . . . . 1 ☐☐<br>YEARS . . . . . 2 ☐☐<br>SOON/NOW . . . . . 993<br>OTHER ______ 996<br>(SPECIFY)<br>DON'T KNOW 998	→ 514
507	CHECK 208:<br>HAS FATHERED CHILDREN ☐ ↓<br>a) Now I have some questions about the future. Would you like to have another child, or would you prefer not to have any more children?<br>HAS NOT FATHERED CHILDREN ☐ ↓<br>b) Now I have some questions about the future. Would you like to have a child, or would you prefer not to have any children?	HAVE (A/ANOTHER) CHILD . . . . . 1<br>NO MORE/NONE . . . . . 2<br>SAYS COUPLE CAN'T GET PREGNANT . . . . . 3<br>WIFE/PARTNER STERILIZED . . . . . 4<br>UNDECIDED/DON'T KNOW 8	 2, 3, 4, 8 → 514
508	CHECK 208:<br>HAS FATHERED CHILDREN ☐ ↓<br>a) How long would you like to wait from now before the birth of another child?<br>HAS NOT FATHERED CHILDREN ☐ ↓<br>b) How long would you like to wait from now before the birth of a child?	MONTHS . . . . . 1 ☐☐<br>YEARS . . . . . 2 ☐☐<br>SOON/NOW . . . . . 993<br>SAYS COUPLE CAN'T GET PREGNANT . . . . . 994<br>OTHER ______ 996<br>(SPECIFY)<br>DON'T KNOW 998	→ 514
509	Are any of your (wives/partners) currently pregnant?	YES . . . . . 1<br>NO . . . . . 2<br>DON'T KNOW 8	 2, 8 → 512

SECTION 5. FERTILITY PREFERENCES

NO.	QUESTIONS AND FILTERS	CODING CATEGORIES	SKIP
510	Now I have some questions about the future. After the (child/children) you and your (wives/partners) are expecting now, would you like to have another child, or would you prefer not to have any more children?	HAVE ANOTHER CHILD . . . . . 1<br>NO MORE . . . . . 2<br>UNDECIDED/DON'T KNOW 8	2, 8 → 514
511	After the birth of the child you are expecting now, how long would you like to wait before the birth of another child?	MONTHS . . . . . 1 [ ][ ]<br>YEARS . . . . . 2 [ ][ ]<br>SOON/NOW . . . . . 993<br>OTHER ______ 996<br>(SPECIFY)<br>DON'T KNOW 998	→ 514
512	CHECK 208:<br>HAS FATHERED CHILDREN [ ] / HAS NOT FATHERED CHILDREN [ ]<br>a) Now I have some questions about the future. Would you like to have another child, or would you prefer not to have any more children?<br>b) Now I have some questions about the future. Would you like to have a child, or would you prefer not to have any children?	HAVE (A/ANOTHER) CHILD . . . . . 1<br>NO MORE/NONE . . . . . 2<br>SAYS COUPLE CAN'T GET PREGNANT . . . . . 3<br>(WIFE/WIVES/PARTNER(S)) STERILIZED . . . . . 4<br>UNDECIDED/DON'T KNOW 8	2, 3, 4, 8 → 514
513	CHECK 208:<br>HAS FATHERED CHILDREN [ ] / HAS NOT FATHERED CHILDREN [ ]<br>a) How long would you like to wait from now before the birth of another child?<br>b) How long would you like to wait from now before the birth of a child?	MONTHS . . . . . 1 [ ][ ]<br>YEARS . . . . . 2 [ ][ ]<br>SOON/NOW . . . . . 993<br>SAYS COUPLE CAN'T GET PREGNANT . . . . . 994<br>OTHER ______ 996<br>(SPECIFY)<br>DON'T KNOW 998	
514	CHECK 203 AND 205:<br>HAS LIVING CHILDREN [ ] / NO LIVING CHILDREN [ ]<br>a) If you could go back to the time you did not have any children and could choose exactly the number of children to have in your whole life, how many would that be?<br>b) If you could choose exactly the number of children to have in your whole life, how many would that be?<br>PROBE FOR A NUMERIC RESPONSE.	NONE . . . . . 00<br>NUMBER . . . . . [ ][ ]<br>OTHER 96<br>______<br>(SPECIFY)	00 → 601<br>96 → 601
515	How many of these children would you like to be boys, how many would you like to be girls and for how many would it not matter if it's a boy or a girl?	BOYS / GIRLS / EITHER<br>NUMBER . . [ ][ ] [ ][ ] [ ][ ]<br>OTHER ______ 96<br>(SPECIFY)	

SECTION 6. EMPLOYMENT AND GENDER ROLES

NO.	QUESTIONS AND FILTERS	CODING CATEGORIES	SKIP
601	Have you done any work in the last seven days?	YES . . . . . 1<br>NO 2	1 → 604
602	Although you did not work in the last seven days, do you have any job or business from which you were absent for leave, illness, vacation, or any other such reason?	YES . . . . . 1<br>NO 2	1 → 604
603	Have you done any work in the last 12 months?	YES . . . . . 1<br>NO 2	2 → 607
604	What is your occupation? That is, what kind of work do you mainly do?	________<br>________ [ ][ ]<br>________	
605	Do you usually work throughout the year, or do you work seasonally, or only once in a while?	THROUGHOUT THE YEAR . . . . . 1<br>SEASONALLY/PART OF THE YEAR . . . . . 2<br>ONCE IN A WHILE 3	
606	Are you paid in cash or kind for this work or are you not paid at all?	CASH ONLY . . . . . 1<br>CASH AND KIND . . . . . 2<br>IN KIND ONLY . . . . . 3<br>NOT PAID 4	
607	CHECK 401:<br>CURRENTLY MARRIED OR LIVING WITH A PARTNER [ ] ↓<br>NOT CURRENTLY MARRIED AND NOT LIVING WITH A PARTNER [ ] →		→ 612
608	CHECK 606:<br>CODE '1' OR '2' CIRCLED [ ] ↓<br>OTHER [ ] →		→ 610
609	Who usually decides how the money you earn will be used: you, your (wife/partner), or you and your (wife/partner) jointly?	RESPONDENT . . . . . 1<br>WIFE/PARTNER . . . . . 2<br>RESPONDENT AND WIFE/PARTNER JOINTLY . . 3<br><br>OTHER ________ 6<br>(SPECIFY)	
610	Who usually makes decisions about health care for yourself: you, your (wife/partner), you and your (wife/partner) jointly, or someone else?	RESPONDENT . . . . . 1<br>WIFE/PARTNER . . . . . 2<br>RESPONDENT AND WIFE/PARTNER JOINTLY . . 3<br>SOMEONE ELSE . . . . . 4<br>OTHER 6	
611	Who usually makes decisions about making major household purchases?	RESPONDENT . . . . . 1<br>WIFE/PARTNER . . . . . 2<br>RESPONDENT AND WIFE/PARTNER JOINTLY . . 3<br>SOMEONE ELSE . . . . . 4<br><br>OTHER ________ 6<br>(SPECIFY)	

SECTION 6. EMPLOYMENT AND GENDER ROLES

NO.	QUESTIONS AND FILTERS	CODING CATEGORIES	SKIP
612	Do you own this or any other house either alone or jointly with someone else?	ALONE ONLY . . . . 1<br>JOINTLY ONLY . . . . 2<br>BOTH ALONE AND JOINTLY . . . . 3<br>DOES NOT OWN 4	 <br><br>→ 615
613	Do you have a title deed for any house you own?	YES . . . . 1<br>NO . . . . 2<br>DON'T KNOW 8	 2, 8 → 615
614	Is your name on the title deed?	YES . . . . 1<br>NO . . . . 2<br>DON'T KNOW 8	
615	Do you own any agricultural or non-agricultural land either alone or jointly with someone else?	ALONE ONLY . . . . 1<br>JOINTLY ONLY . . . . 2<br>BOTH ALONE AND JOINTLY . . . . 3<br>DOES NOT OWN 4	 <br><br>→ 618
616	Do you have a title deed for any land you own?	YES . . . . 1<br>NO . . . . 2<br>DON'T KNOW 8	 2, 8 → 618
617	Is your name on the title deed?	YES . . . . 1<br>NO . . . . 2<br>DON'T KNOW 8	
618	In your opinion, is a husband justified in hitting or beating his wife in the following situations:<br><br>a) If she goes out without telling him?<br>b) If she neglects the children?<br>c) If she argues with him?<br>d) If she refuses to have sex with him?<br>e) If she does not properly cook the food?	YES NO DK<br>a) GOES OUT . . . . 1 2 8<br>b) NEGLECTS CHILDREN . . 1 2 8<br>c) ARGUES . . . . 1 2 8<br>d) REFUSES SEX . . . . 1 2 8<br>e) FOOD 1 2 8	

SECTION 7. HIV/AIDS

NO.	QUESTIONS AND FILTERS	CODING CATEGORIES	SKIP
701	Now I would like to talk about something else. Have you ever heard of HIV or AIDS?	YES . . . . 1<br>NO 2	 → 727
702	HIV is the virus that can lead to AIDS. Can people reduce their chance of getting HIV by having just one uninfected sex partner who has no other sex partners?	YES . . . . 1<br>NO . . . . 2<br>DON'T KNOW 8	
703	Can people get HIV from mosquito bites?	YES . . . . 1<br>NO . . . . 2<br>DON'T KNOW 8	
704	Can people reduce their chance of getting HIV by using a condom every time they have sex?	YES . . . . 1<br>NO . . . . 2<br>DON'T KNOW 8	
704A	Can people reduce their chance of getting the AIDS virus by not having sexual intercourse at all?	YES . . . . 1<br>NO . . . . 2<br>DON'T KNOW 8	
705	Can people get HIV by sharing food with a person who has HIV?	YES . . . . 1<br>NO . . . . 2<br>DON'T KNOW 8	
706	Can people get HIV because of witchcraft or other supernatural means?	YES . . . . 1<br>NO . . . . 2<br>DON'T KNOW 8	
707	Is it possible for a healthy-looking person to have HIV?	YES . . . . 1<br>NO . . . . 2<br>DON'T KNOW 8	
708	Can HIV be transmitted from a mother to her baby:<br><br>a) During pregnancy?<br>b) During delivery?<br>c) By breastfeeding?	YES NO DK<br><br>a) DURING PREGNANCY . . 1 2 8<br>b) DURING DELIVERY . . . . 1 2 8<br>c) BREASTFEEDING 1 2 8	
709	CHECK 708:<br>AT LEAST ONE 'YES' ☐ ↓	OTHER ☐ →	711
710	Are there any special drugs that a doctor or a nurse can give to a woman infected with HIV to reduce the risk of transmission to the baby?	YES . . . . 1<br>NO . . . . 2<br>DON'T KNOW 8	
711	**CHECK FOR PRESENCE OF OTHERS. BEFORE CONTINUING, MAKE EVERY EFFORT TO ENSURE PRIVACY.**		
712	I don't want to know the results, but have you ever been tested for HIV?	YES . . . . 1<br>NO 2	 → 716
713	How many months ago was your most recent HIV test?	MONTHS AGO . . . . ☐☐<br><br>TWO OR MORE YEARS 95	

NO.	QUESTIONS AND FILTERS	CODING CATEGORIES	SKIP
714	I don't want to know the results, but did you get the results of the test?	YES . . . 1<br>NO . . . 2	
714A	The last time you had the test, did you yourself ask for the test, was it offered to you and you accepted, or was it required by the health provider?	TEST REQUESTED BY THE RESPONDENT . . . 1<br>TEST OFFERED BY THE HEALTH PROVIDER . . 2<br>TEST REQUIRED BY THE HEALTH PROVIDER . . 3	
715	Where was the test done?<br><br>PROBE TO IDENTIFY THE TYPE OF SOURCE.<br><br>IF UNABLE TO DETERMINE IF PUBLIC OR PRIVATE SECTOR, WRITE THE NAME OF THE PLACE.<br><br>__________<br>(NAME OF PLACE)	**PUBLIC SECTOR**<br>GOVERNMENT HOSPITAL . . . 11<br>GOVERNMENT HEALTH CENTER . . . 12<br>GOVERNMENT HEALTH POST/ OUTREACH . . . 13<br>HSA . . . 14<br>DOOR TO DOOR . . . 15<br>OTHER PUBLIC SECTOR<br>__________ 16<br>(SPECIFY)<br><br>**CHAM/MISSION**<br>HOSPITAL . . . 21<br>HEALTH CENTER . . . 22<br>MOBILE CLINIC . . . 23<br>DOOR TO DOOR . . . 24<br><br>**PRIVATE MEDICAL SECTOR**<br>PRIVATE HOSPITAL/CLINIC/ PRIVATE DOCTOR . . . 31<br>PHARMACY . . . 32<br>OTHER PRIVATE MEDICAL SECTOR<br>__________ 36<br>(SPECIFY)<br><br>**BLM** . . . 41<br><br>**MACRO** . . . 51<br><br>**OTHER SOURCE**<br>HOME . . . 61<br>WORKPLACE . . . 62<br>CORRECTIONAL FACILITY . . . 63<br><br>OTHER __________ 96<br>(SPECIFY)	→ 718
716	Do you know of a place where people can go to get an HIV test?	YES . . . 1<br>NO . . . 2	 → 718
717	Where is that?<br><br>Any other place?<br><br>PROBE TO IDENTIFY THE TYPE OF SOURCE.<br><br>IF UNABLE TO DETERMINE IF PUBLIC OR PRIVATE SECTOR, WRITE THE NAME OF THE PLACE.<br><br>__________<br>(NAME OF PLACE)	**PUBLIC SECTOR**<br>GOVERNMENT HOSPITAL . . . A<br>GOVERNMENT HEALTH CENTER . . . B<br>GOVERNMENT HEALTH POST/ OUTREACH . . . C<br>HSA . . . D<br>DOOR TO DOOR . . . E<br>OTHER PUBLIC SECTOR<br>__________ F<br>(SPECIFY)<br><br>**CHAM/MISSION**<br>HOSPITAL . . . G<br>HEALTH CENTER . . . H<br>MOBILE CLINIC . . . I<br>DOOR TO DOOR . . . J<br><br>**PRIVATE MEDICAL SECTOR**<br>PRIVATE HOSPITAL/CLINIC/ PRIVATE DOCTOR . . . K<br>PHARMACY . . . L<br>OTHER PRIVATE MEDICAL SECTOR<br>__________ M<br>(SPECIFY)<br><br>**BLM** . . . N<br><br>**MACRO** . . . O<br><br>**OTHER SOURCE**<br>HOME . . . P<br>WORKPLACE . . . Q<br>CORRECTIONAL FACILITY . . . R<br><br>OTHER __________ X<br>(SPECIFY)	
718	Have you heard of test kits people can use to test themselves for HIV?	YES . . . 1<br>NO . . . 2	 → 720
719	Have you ever tested yourself for HIV using a self-test kit?	YES . . . 1<br>NO . . . 2	

NO.	QUESTIONS AND FILTERS	CODING CATEGORIES	SKIP
720	Would you buy fresh vegetables from a shopkeeper or vendor if you knew that this person had HIV?	YES . . . 1<br>NO . . . 2<br>DON'T KNOW/NOT SURE/DEPENDS . . . 8	
721	Do you think children living with HIV should be allowed to attend school with children who do not have HIV?	YES . . . 1<br>NO . . . 2<br>DON'T KNOW/NOT SURE/DEPENDS . . . 8	
722	Do you think people hesitate to take an HIV test because they are afraid of how other people will react if the test result is positive for HIV?	YES . . . 1<br>NO . . . 2<br>DON'T KNOW/NOT SURE/DEPENDS . . . 8	
723	Do people talk badly about people living with HIV, or who are thought to be living with HIV?	YES . . . 1<br>NO . . . 2<br>DON'T KNOW/NOT SURE/DEPENDS . . . 8	
724	Do people living with HIV, or thought to be living with HIV, lose the respect of other people?	YES . . . 1<br>NO . . . 2<br>DON'T KNOW/NOT SURE/DEPENDS . . . 8	
725	Do you agree or disagree with the following statement: I would be ashamed if someone in my family had HIV.	AGREE . . . 1<br>DISAGREE . . . 2<br>DON'T KNOW/NOT SURE/DEPENDS . . . 8	
726	Do you fear that you could get HIV if you come into contact with the saliva of a person living with HIV?	YES . . . 1<br>NO . . . 2<br>SAYS HE HAS HIV . . . 3<br>DON'T KNOW/NOT SURE/DEPENDS . . . 8	
727	CHECK 701:<br>HEARD ABOUT HIV OR AIDS ☐ / NOT HEARD ABOUT HIV OR AIDS ☐<br>a) Apart from HIV, have you heard about other infections that can be transmitted through sexual contact?<br>b) Have you heard about infections that can be transmitted through sexual contact?	YES . . . 1<br>NO . . . 2	
728	CHECK 414:<br>HAS HAD SEXUAL INTERCOURSE ☐ / NEVER HAD SEXUAL INTERCOURSE ☐		736
729	CHECK 727: HEARD ABOUT OTHER SEXUALLY TRANSMITTED INFECTIONS?<br>YES ☐ / NO ☐		731
730	Now I would like to ask you some questions about your health in the last 12 months. During the last 12 months, have you had a disease which you got through sexual contact?	YES . . . 1<br>NO . . . 2<br>DON'T KNOW . . . 8	
731	Sometimes men experience an abnormal discharge from their penis. During the last 12 months, have you had an abnormal discharge from your penis?	YES . . . 1<br>NO . . . 2<br>DON'T KNOW . . . 8	
732	Sometimes men have a sore or ulcer near their penis. During the last 12 months, have you had a sore or ulcer on or near your penis?	YES . . . 1<br>NO . . . 2<br>DON'T KNOW . . . 8	

NO.	QUESTIONS AND FILTERS	CODING CATEGORIES	SKIP
733	CHECK 730, 731 AND 732:<br>HAS HAD AN INFECTION (ANY 'YES') ☐ ↓<br>HAS NOT HAD AN INFECTION OR DOES NOT KNOW ☐		→ 736
734	The last time you had (PROBLEM FROM 730/731/732), did you seek any kind of advice or treatment?	YES . . . . . 1<br>NO 2	 → 736
735	Where did you go?<br><br>Any other place?<br><br>PROBE TO IDENTIFY THE TYPE OF SOURCE.<br><br>IF UNABLE TO DETERMINE IF PUBLIC OR PRIVATE SECTOR, WRITE THE NAME OF THE PLACE.<br><br>__________________<br>(NAME OF PLACE)	**PUBLIC SECTOR**<br>GOVERNMENT HOSPITAL . . . . . A<br>GOVERNMENT HEALTH CENTER . . . . . B<br>GOVERNMENT HEALTH POST/ OUTREACH . . . . . C<br>HSA . . . . . D<br>DOOR TO DOOR . . . . . E<br>OTHER PUBLIC SECTOR<br>______________ F<br>(SPECIFY)<br><br>**CHAM/MISSION**<br>HOSPITAL . . . . . G<br>HEALTH CENTER . . . . . H<br>MOBILE CLINIC . . . . . I<br>DOOR TO DOOR . . . . . J<br><br>**PRIVATE MEDICAL SECTOR**<br>PRIVATE HOSPITAL/CLINIC/ PRIVATE DOCTOR . . . . . K<br>PHARMACY . . . . . L<br>OTHER PRIVATE MEDICAL SECTOR<br>______________ M<br>(SPECIFY)<br><br>**BLM** . . . . . N<br><br>**MACRO** . . . . . O<br><br>**OTHER SOURCE**<br>HOME . . . . . P<br>WORKPLACE . . . . . Q<br>CORRECTIONAL FACILITY . . . . . R<br><br>OTHER ______________ X<br>(SPECIFY)	
736	If a wife knows her husband has a disease that she can get during sexual intercourse, is she justified in asking that they use a condom when they have sex?	YES . . . . . 1<br>NO . . . . . 2<br>DON'T KNOW 8	
737	Is a wife justified in refusing to have sex with her husband when she knows he has sex with other women?	YES . . . . . 1<br>NO . . . . . 2<br>DON'T KNOW 8	

SECTION 8. OTHER HEALTH ISSUES

NO.	QUESTIONS AND FILTERS	CODING CATEGORIES	SKIP
801	Some men are circumcised, that is, the foreskin is completely removed from the penis. Are you circumcised?	YES . . . 1<br>NO . . . 2<br>DON'T KNOW . . . 8	 NO, DON'T KNOW → 805
802	How old were you when you got circumcised?	AGE IN COMPLETED YEARS . . . . [ ][ ]<br>DURING CHILDHOOD (<5 YEARS) . . . 95<br>DON'T KNOW . . . 98	
803	Who did the circumcision?	TRADITIONAL PRACTITIONER/FAMILY/FRIENI . . 1<br>HEALTH WORKER/PROFESSIONAL . . . 2<br>OTHER . . . 3<br>DON'T KNOW . . . 8	
804	Where was it done?	HEALTH FACILITY . . . 1<br>HOME OF A HEALTH WORKER/PROFESSION/ . . 2<br>CIRCUMCISION DONE AT HOME . . . 3<br>INITIATION CEREMONY . . . 4<br>OTHER HOME/PLACE . . . 5<br>DON'T KNOW . . . 8	
805	Now I would like to ask you some other questions relating to health matters. Have you had an injection for any reason in the last 12 months?<br><br>IF YES: How many injections have you had?<br><br>IF NUMBER OF INJECTIONS IS 90 OR MORE, OR DAILY FOR 3 MONTHS OR MORE, RECORD '90'. IF NON-NUMERIC ANSWER, PROBE TO GET AN ESTIMATE.	NUMBER OF INJECTIONS . . . . [ ][ ]<br><br>NONE . . . 00	 <br>NONE → 808
806	Among these injections, how many were administered by a doctor, a nurse, a pharmacist, a dentist, or any other health worker?<br><br>IF NUMBER OF INJECTIONS IS 90 OR MORE, OR DAILY FOR 3 MONTHS OR MORE, RECORD '90'. IF NON-NUMERIC ANSWER, PROBE TO GET AN ESTIMATE.	NUMBER OF INJECTIONS . . . . [ ][ ]<br><br>NONE . . . 00	 <br>NONE → 808
807	The last time you got an injection from a health worker, did he/she take the syringe and needle from a new, unopened package?	YES . . . 1<br>NO . . . 2<br>DON'T KNOW . . . 8	
808	Do you currently smoke tobacco every day, some days, or not at all?	EVERY DAY . . . 1<br>SOME DAYS . . . 2<br>NOT AT ALL . . . 3	EVERY DAY → 811<br><br>NOT AT ALL → 810
809	In the past, have you smoked tobacco every day?	YES . . . 1<br>NO . . . 2	YES, NO → 812
810	In the past, have you ever smoked tobacco every day, some days, or not at all?	EVERY DAY . . . 1<br>SOME DAYS . . . 2<br>NOT AT ALL . . . 3	EVERY DAY, SOME DAYS, NOT AT ALL → 813

SECTION 8. OTHER HEALTH ISSUES

NO.	QUESTIONS AND FILTERS	CODING CATEGORIES	SKIP
811	On average, how many of the following products do you currently smoke each day? Also, let me know if you use the product, but not every day.<br><br>IF RESPONDENT REPORTS USING THE PRODUCT BUT NOT EVERY DAY, RECORD '888'. IF THE PRODUCT IS NOT USED AT ALL, RECORD '000'.	NUMBER DAILY	
	a) Manufactured cigarettes?	a) MANUFACTURED CIGARETTES . . . . [ ][ ][ ]	→ 813
	b) Hand-rolled cigarettes?	b) HAND-ROLLED CIGARETTES . . . . [ ][ ][ ]	
	c) Pipes full of tobacco?	c) PIPES FULL OF TOBACCO . . . . [ ][ ][ ]	
	d) Cigars, cheroots, or cigarillos?	d) CIGARS, CHEROOTS, OR CIGARILLOS . . . . . [ ][ ][ ]	
	e) Number of water pipe sessions?	e) NUMBER OF WATER PIPE SESSIONS . . . . . [ ][ ][ ]	
	f) Any others? ________ (SPECIFY)	f) OTHERS . . . . [ ][ ][ ]	
812	On average, how many of the following products do you currently smoke each week? Also, let me know if you use the product, but not every week.<br><br>IF RESPONDENT REPORTS USING THE PRODUCT BUT NOT EVERY WEEK, RECORD '888'. IF THE PRODUCT IS NOT USED AT ALL, RECORD '000'.	NUMBER WEEKLY	
	a) Manufactured cigarettes?	a) MANUFACTURED CIGARETTES . . . . [ ][ ][ ]	
	b) Hand-rolled cigarettes?	b) HAND-ROLLED CIGARETTES . . . . [ ][ ][ ]	
	c) Pipes full of tobacco?	c) PIPES FULL OF TOBACCO . . . . [ ][ ][ ]	
	d) Cigars, cheroots, or cigarillos?	d) CIGARS, CHEROOTS, OR CIGARILLOS . . . . . [ ][ ][ ]	
	e) Number of water pipe sessions?	e) NUMBER OF WATER PIPE SESSIONS . . . . . [ ][ ][ ]	
	f) Any others? ________ (SPECIFY)	f) OTHERS . . . . [ ][ ][ ]	
813	Do you currently use smokeless tobacco every day, some days, or not at all?	EVERY DAY . . . . 1<br>SOME DAYS . . . . 2<br>NOT AT ALL 3	 → 815<br>→ 816
814	On average, how many times a day do you use the following products? Also, let me know if you use the product, but not every day.<br><br>IF RESPONDENT REPORTS USING THE PRODUCT BUT NOT EVERY DAY, RECORD '888'. IF THE PRODUCT IS NOT USED AT ALL, RECORD '000'.	TIMES DAILY	
	a) Snuff, by mouth?	a) SNUFF, BY MOUTH . . . . [ ][ ][ ]	→ 816
	b) Snuff, by nose?	b) SNUFF, BY NOSE . . . . [ ][ ][ ]	
	c) Chewing tobacco?	c) CHEWING TOBACCO . . . . . [ ][ ][ ]	
	d) Any others? ________ (SPECIFY)	d) ANY OTHERS . . . . [ ][ ][ ]	

NO.	QUESTIONS AND FILTERS	CODING CATEGORIES	SKIP
815	On average, how many times a week do you use the following products? Also, let me know if you use the product, but not every week.<br><br>IF RESPONDENT REPORTS USING THE PRODUCT BUT NOT EVERY WEEK, RECORD '888'. IF THE PRODUCT IS NOT USED AT ALL, RECORD '000'.<br>a) Snuff, by mouth?<br>b) Snuff, by nose?<br>c) Chewing tobacco?<br>d) Any others?<br>_______________ (SPECIFY)	TIMES WEEKLY<br>a) SNUFF, BY MOUTH . . . . [ ][ ][ ]<br>b) SNUFF, BY NOSE . . . . [ ][ ][ ]<br>c) CHEWING TOBACCO . . . . . [ ][ ][ ]<br>d) ANY OTHERS . . . . [ ][ ][ ]	
816	Are you covered by any health insurance?	YES . . . . 1<br>NO 2	 → 818
817	What type of health insurance are you covered by?<br><br>RECORD ALL MENTIONED.	HEALTH INSURANCE THROUGH EMPLOYER . . . . A<br>OTHER PRIVATELY PURCHASED COMMERCIAL HEALTH INSURANCE . . . . . B<br><br>OTHER _______________ X<br>(SPECIFY)	
818	Have you ever heard of an illness called tuberculosis or TB?	YES . . . . 1<br>NO 2	 → 822
819	How does tuberculosis spread from one person to another?<br><br>PROBE: Any other ways?<br>RECORD ALL MENTIONED.	THROUGH THE AIR WHEN COUGHING OR SNEEZING . . . . A<br>THROUGH SHARING UTENSILS . . . . . B<br>THROUGH TOUCHING A PERSON WITH TE . . . . . C<br>THROUGH FOOD . . . . D<br>THROUGH SEXUAL CONTACT . . . . . . E<br>THROUGH MOSQUITO BITES . . . . . . F<br><br>OTHER _______________ X<br>(SPECIFY)<br>DON'T KNOW . . . . Z	
820	Can tuberculosis be cured?	YES . . . . 1<br>NO . . . . 2<br>DON'T KNOW 8	
821	If a member of your family got tuberculosis, would you want it to remain a secret or not?	YES, REMAIN A SECRET . . . . 1<br>NO . . . . 2<br>DON'T KNOW/NOT SURE/DEPENDS 8	
822	RECORD THE TIME.	HOURS . . . . [ ][ ]<br>MINUTES . . . . [ ][ ]	

INTERVIEWER'S OBSERVATIONS

TO BE FILLED IN AFTER COMPLETING INTERVIEW

COMMENTS ABOUT INTERVIEW:

COMMENTS ON SPECIFIC QUESTIONS:

ANY OTHER COMMENTS:

SUPERVISOR'S OBSERVATIONS

EDITOR'S OBSERVATIONS

2015-2016 MALAWI DEMOGRAPHIC AND HEALTH SURVEY
FIELDWORKER QUESTIONNAIRE

MALAWI GOVERNEMNT
NATIONAL STATISTICAL OFFICE

LANGUAGE OF QUESTIONNAIRE ENGLISH

NO.	QUESTIONS AND FILTERS	CODING CATEGORIES	SKIP
100	What is your name?	NAME ______	
101	RECORD INTERVIEWER/EDITOR/SUPERVISOR NUMBER	NUMBER . . . . [ ][ ][ ][ ]	

INSTRUCTIONS

We are collecting information on the DHS field staff. Please fill in the information below. The information will be part of the survey data files. Your name will not be in the data files; your information will remain anonymous. If there is any question you do not want to answer you may skip it and go to the next question.

NO.	QUESTIONS AND FILTERS	CODING CATEGORIES	SKIP
102	In what [REGION] do you live?	NOTHERN . . . . 01<br>CENTRAL . . . . 02<br>SOUTHERN 03	
103	Do you live in a city, town, or rural area?	CITY . . . . 1<br>TOWN . . . . 2<br>RURAL 3	
104	How old are you?<br>RECORD AGE IN COMPLETED YEARS.	AGE . . . . [ ][ ]	
105	Are you male or female?	MALE . . . . 1<br>FEMALE 2	
106	What is your current marital status?	CURRENTLY MARRIED . . . . 1<br>LIVING WITH A MAN/WOMAN . . . . 2<br>WIDOWED . . . . 3<br>DIVORCED . . . . 4<br>SEPARATED . . . . 5<br>NEVER MARRIED OR LIVED WITH A MAN/WOMAN 6	
107	How many living children do you have?<br>INCLUDE ONLY CHILDREN WHO ARE YOUR BIOLOGICAL CHILDREN.	LIVING CHILDREN . . . . [ ][ ]	
108	Have you ever had a child who died?	YES . . . . 1<br>NO 2	
109	What is the highest level of school you attended: primary, secondary, or higher?	PRIMARY . . . . 1<br>SECONDARY . . . . 2<br>HIGHER 3	
110	What is the highest [GRADE/FORM/YEAR] you completed at that level?<br>IF COMPLETED LESS THAN ONE YEAR AT THAT LEVEL, RECORD '00'.	[GRADE/FORM/YEAR] . . . . [ ][ ]	

NO.	QUESTIONS AND FILTERS	CODING CATEGORIES	SKIP
111	What is your religion?	CATHOLIC . . . 01<br>CCAP . . . 02<br>ANGLICAN . . . 03<br>SEVENTH DAY ADVENT./BAPTIS . . . 04<br>OTHER CHRISTIAN . . . 05<br>MUSLIM . . . 06<br><br>NO RELIGION . . . 95<br><br>OTHER ______ 96<br>(SPECIFY)	
112	What is your ethnicity?	CHEWA . . . 01<br>TUMBUKA . . . 02<br>LOMWE . . . 03<br>TONGA . . . 04<br>YAO . . . 05<br>SENA . . . 06<br>NKHONDE . . . 07<br>NGONI . . . 08<br><br>OTHER ______<br>(SPECIFY) 96	
113	What is your mother tongue/native language (language spoken at home growing up)?	CHICHEWA . . . 01<br>TUMBUKA . . . 02<br><br>OTHER ______ 96<br>(SPECIFY)	
114	What other languages can you speak?<br><br>RECORD ALL OTHER LANGUAGES YOU CAN SPEAK.	ENGLISH . . . A<br>CHICHEWA . . . B<br>TUMBUKA . . . C<br><br>OTHER ______ X<br>(SPECIFY)<br>NO OTHER LANGUAGE . . . Y	
115	Have you ever worked on a DHS survey prior to this one?	YES . . . 1<br>NO . . . 2	
116	Have you ever worked on any other survey prior to this one (not a DHS)?	YES . . . 1<br>NO . . . 2	
117	Were you already working for NSO at the time you were employed to work on this DHS?	YES . . . 1<br>NO . . . 3	 → 119
118	Are you a permanent or temporary employee of NSO?	PERMANENT . . . 1<br>TEMPORARY . . . 2	
119	If you have comments, please write them here.		
